# A metabolism-related 4-lncRNA prognostic signature and corresponding mechanisms in intrahepatic cholangiocarcinoma

**DOI:** 10.1186/s12885-021-08322-5

**Published:** 2021-05-25

**Authors:** Wenbo Zou, Zizheng Wang, Fei Wang, Lincheng Li, Rong Liu, Minggen Hu

**Affiliations:** 1 Medical School of Chinese PLA, Beijing, China; 2 Faculty of Hepato-Pancreato-Biliary Surgery, Chinese PLA General Hospital, No.28 Fuxing Road, Haidian District, Beijing, 100853 China; 3 Institute of Hepatobiliary Surgery of Chinese PLA, Beijing, China; 4 Key Laboratory of Digital Hepetobiliary Surgery, PLA, Beijing, China

**Keywords:** Long non-coding RNA, Intrahepatic cholangiocarcinoma, Overall survival, Signature, Nomogram

## Abstract

**Background:**

Long non-coding RNA (lncRNA) plays a critical role in the malignant progression of intrahepatic cholangiocarcinoma (iCCA). This study aimed to establish a 4-lncRNA prognostic signature and explore corresponding potential mechanisms in patients with iCCA.

**Methods:**

The original lncRNA-seq and clinical data were collected from the TCGA and GEO databases. Overlapping and differentially expressed lncRNAs (DE-lncRNAs) were further identified from transcriptome data. Univariate regression analysis was performed to screen survival-related DE-lncRNAs, which were further selected to develop an optimal signature to predict prognosis using multivariate regression analysis. The Kaplan-Meier survival curve visualized the discrimination of the signature on overall survival (OS). The area under the curve (AUC) and C-index were used to verify the predictive accuracy of the signature. Combined with clinical data, multivariate survival analysis was used to reveal the independent predictive capability of the signature. In addition, a prognostic nomogram was constructed. Finally, the common target genes of 4 lncRNAs were predicted by the co-expression method, and the corresponding functions were annotated by GO and KEGG enrichment analysis. Gene set enrichment analysis (GSEA) was also performed to explore the potential mechanism of the signature. Quantitative real-time PCR was used to evaluated the expression of 4 lncRNAs in an independent cohort.

**Results:**

We identified and constructed a 4-lncRNA (AC138430.1, AGAP2-AS1, AP001783.1, and AP005233.2) prognostic signature using regression analysis, and it had the capability to independently predict prognosis. The AUCs were 0.952, 0.909, and 0.882 at 1, 2, and 3 years, respectively, and the C-index was 0.808, which showed good predictive capability. Subsequently, combined with clinical data, we constructed a nomogram with good clinical application. Finally, 252 target genes of all four lncRNAs were identified by the co-expression method, and functional enrichment analysis showed that the signature was strongly correlated with metabolism-related mechanisms in tumourigenesis. The same results were also validated via GSEA.

**Conclusion:**

We demonstrated that a metabolism-related 4-lncRNA prognostic signature could be a novel biomarker and deeply explored the target genes and potential mechanism. This study will provide a promising therapeutic strategy for patients with intrahepatic cholangiocarcinoma.

**Supplementary Information:**

The online version contains supplementary material available at 10.1186/s12885-021-08322-5.

## Introduction

Intrahepatic cholangiocarcinoma (iCCA) is a malignant hepatobiliary tumour originating from the intrahepatic bile ducts and accounts for 10–20% of all bile duct malignancies [1]. Over the past few decades, the incidence of iCCA has been reported to be steadily increasing in most of the world, and it frequently occurs in patients with underlying liver disease [1, 2]. Because of its high malignancy, hidden nature, and aggressive nature, iCCA lacks obvious symptoms in the early stages, which makes diagnosis and treatment difficult for clinicians [2, 3]. Although the prognosis of iCCA patients has improved in recent years with the enhancement of surgical techniques, the application of adjuvant therapies, and the gradual promotion of emerging immunotherapies and targeted therapies for specific mutation sites [4, 5], the long-term prognosis of iCCA patients still needs to be strengthened. Therefore, there is an urgent need to explore new prognostic indicators and reveal potential mechanisms to better understand disease progression.

lncRNAs are greater than 200 bp in length and do not have a protein-coding function [6]. With the increasing exploration and development of cancerous molecular pathology, numerous studies have proven that lncRNAs are functional RNAs that can play a corresponding role similar to that of oncogenes or tumour suppressor genes [7] and are not, as previously thought, “transcriptional noise” (non-functional RNA). Recently, researchers have become very interested in their role in tumour invasion, metastasis, and signalling pathways, and previous studies have demonstrated the involvement of lncRNAs as key molecules in the malignant biology of various cancers [8–10]. At present, some studies have described the relevant roles of fractional lncRNAs in cholangiocarcinoma [11–13], but the role of more lncRNAs in the malignant progression of intrahepatic cholangiocarcinoma still needs to be further identified and analysed.

In this study, we identified and constructed a metabolism-related lncRNA prognostic signature and incorporated it into the nomogram for OS prediction. The relevant target genes of the signature and their potential mechanisms were also explored. The findings may provide new prognostic biomarkers that reveal a novel perspective for the individualized treatment of patients with iCCA.

## Materials and methods

### Data obtaining and pre-processing

The CHOL RNA-seq dataset and clinical data were downloaded from the TCGA database (https://portal.gdc.cancer.gov/). Another dataset, GSE107943, was collected from the GEO database (https://www.ncbi.nlm.nih.gov/geo/). Then, the lncRNA matrix was annotated and extracted from the RNA-seq dataset using the “Perl” language and “GenomicTools” package.

### Differential expression analysis

The differentially expressed genes and DE-lncRNAs were analysed by using the “edgeR” package with the threshold FDR < 0.05 coupled with |log2foldchange(FC)| > 2 in two datasets [14]. Overlapping DE-lncRNAs were extracted and exhibited using the “Venn” package [15], and the heatmap and volcano map were plotted to illustrate the up- (red) and downregulated (green) DE-lncRNAs using the “ggplot2” package.

### Construction and validation of the prognostic signature

The survival-related DE-lncRNAs were screened using univariate regression analysis and were further incorporated into the multivariable regression analysis to obtain independent prognostic DE-lncRNAs for constructing a prognostic signature. *p* < 0.05 was deemed the threshold. The coefficients and expression levels of DE-lncRNAs were obtained to calculate the risk score. Based on the median risk score, we divided all patients into high- and low-risk groups. The Kaplan-Meier (KM) survival curve described the predictive power of indicators using the “survival” and “survminer” packages. The AUC of the receiver operating characteristic (ROC) curve and C-index were applied to verify the predictive accuracy using the “timeROC” and “rms” packages. The clinical value of the signature was analysed. To reveal the potential mechanism of the prognostic signature, we performed gene set enrichment analysis (GSEA).

### Validation of lncRNAs using quantitative real-time PCR

Tumour tissues with pathological diagnosis of intrahepatic cholangiocarcinoma and normal tissues were prospective collected from the Chinese PLA general Hospital. qRT-PCR was performed to validate the gene expression changes of all four lncRNAs. TRIzol reagent (Ambion) was used to extracted total RNA; NanoPhotometer® C40 Touch (IMPLEN) was used to assess the RNA purity based on the ratio of OD260/280 and 260/230; Eppendorf Mastercycler® was used to perform reverse transcription of qualified RNA to single-stranded complementary DNA according to the manufacturer’s instructions; QuanStudio™ 5 Real-Time PCR instrument was used to implement real-time quantification; β-actin was used as internal reference; finally, we recorded cycle threshold (Ct) and calculated the relative expression of four lncRNAs using the 2^−ΔΔCt^ method. Primers sequences of four lncRNAs and β-actin were shown in Supplementary Table S1.

### Co-expression prediction of target genes and functional annotation

We calculated the Pearson’s correlation coefficients between four DE-lncRNA expression profiles and protein-coding genes (PCGs) to determine the co-expression relationship, where PCGs with |Pearson’s correlation coefficient| > 0.5 were considered lncRNA-associated PCGs. Then, we took the intersection of PCGs and differentially expressed genes, and the overlapping PCGs were further functionally annotated using Gene Ontology (GO) and Kyoto Encyclopedia of Genes and Genomes (KEGG) analysis in the “clusterProfiler” package [16].

### Development and assessment of the nomogram

To reveal the independent prognostic value of the signature, univariate and multivariate regression analyses were implemented on the signature and clinical data involving age, sex, race, T stage, N stage, M stage, and AJCC stage. Based on all independently predictive variables, we constructed a prediction nomogram using the “rms” and “foreign” packages. The predictivity of the nomogram was validated using AUC and calibration curves.

### Statistical analysis

R version 4.0.2 software and its resource packages were used for statistical analysis and related visualization graphics. Pearson’s correlation coefficients were calculated between the expression profiles of four DE-lncRNAs and PCGs to determine the co-expression relationship among them, and |coef| > 0.5 was considered to have relevance. Principal component analysis (PCA) was implemented to efficiently downscale high-dimensional lncRNA sequencing data to improve the ability for data identification and was implemented to analyse the capability to distinguish patients with high or low risk based on a risk model, differentially expressed lncRNAs, and whole lncRNA sequences. The function of the prognostic signature was annotated by GSEA. Statistical significance of all statistical tests implemented in this study was determined as two-sided *p* < 0.05.

## Results

### Differential expression analysis

**Fig. 1:**
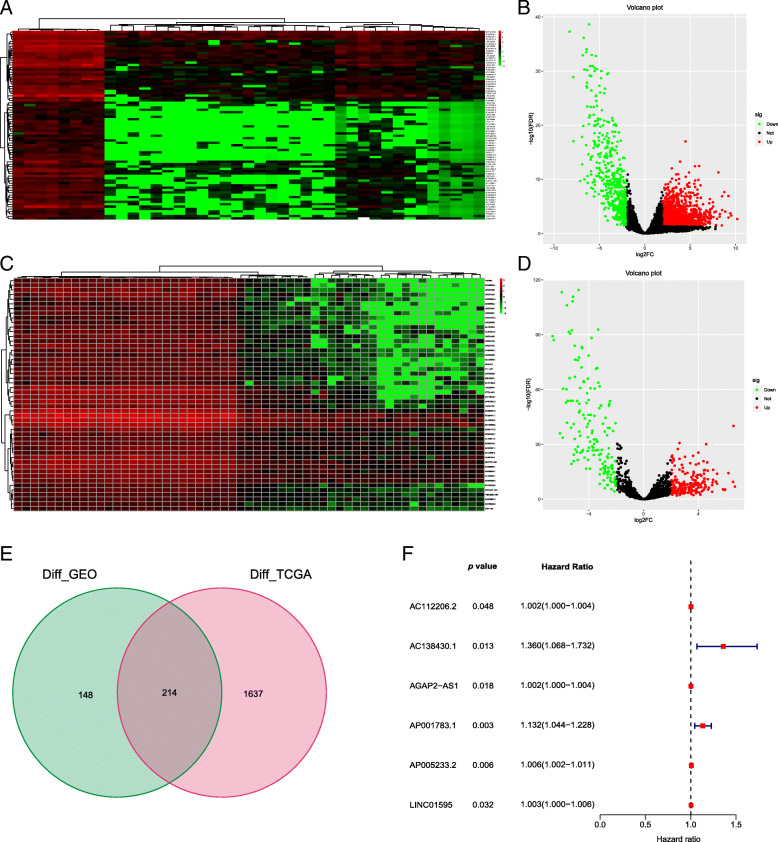
Differentially expressed analysis. **a-b** Heatmap and volcano plot in the TCGA dataset. **c-d** Heatmap and volcano plot in the GEO dataset. **e** Venn diagram of the differentially expressed lncRNA intersection. **f** Survival-related lncRNAs were obtained using univariate regression analysis

Meeting the screening criteria, a total of 1851 DE-lncRNAs were identified from the TCGA dataset, of which 1334 were upregulated and 518 were downregulated (Fig. 1a-b), while a total of 362 DE-lncRNAs were identified from the GEO dataset, of which 197 were upregulated and 165 were downregulated (Fig. 1c-d). Finally, 214 overlapping DE-lncRNAs were extracted from the intersection of DE-lncRNAs (Fig. 1e).

### Construction and validation of the prognostic signature

Univariate Cox regression analysis showed that six lncRNAs were significantly related to OS in all DE-lncRNAs (*p* < 0.05, Fig. 1f). Subsequently, an optimal prognostic signature involving 4 lncRNAs (AC138430.1, AGAP2-AS1, AP001783.1, and AP005233.2) was constructed using multivariate Cox regression analysis, of which the lncRNAs AGAP2-AS1 and AP005233.2 were upregulated, whereas the lncRNAs AC138430.1 and AP001783.1 were downregulated in tumour tissues. The risk coefficients suggested that all 4 lncRNAs were risk factors for iCCA with |coef| > 0 (Table 1).

**Table 1:** The Four lncRNAs were identified using the multivariate analysis

LncRNA	Coefficient	HR	95% Confidence interval	*p* value
AC138430.1	0.459	1.582	1.143–2.190	0.006
AGAP2-AS1	0.004	1.004	1.002–1.006	0.001
AP001783.1	0.096	1.101	0.997–1.216	0.058
AP005233.2	0.010	1.010	1.004–1.016	0.001

*HR* Hazard Ratio

**Fig. 2:**
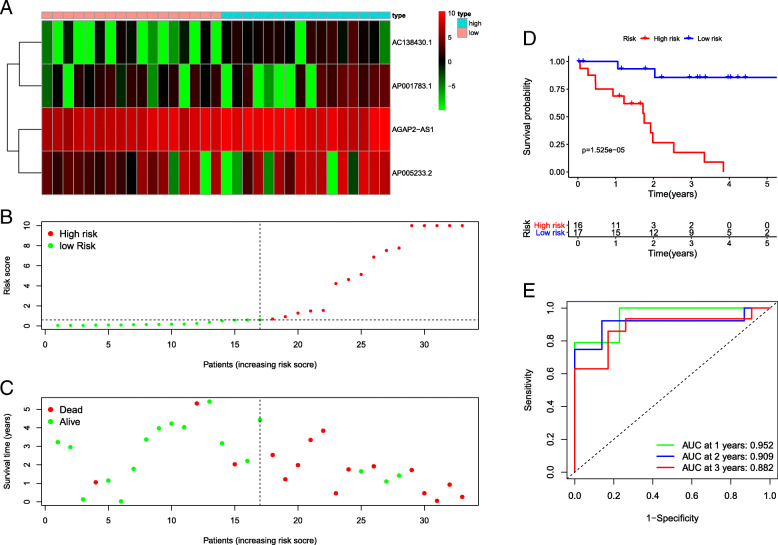
**a-c** Survival condition plots and heatmap of 4 IRGs. **d** Kaplan-Meier survival curve. **e** Time-dependent ROC curves for predicting OS at 1, 2, and 3 years

**Fig. 3:**
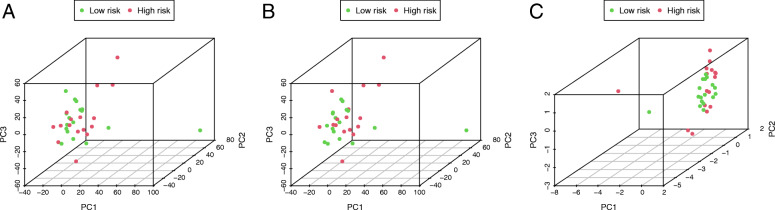
Principal components analysis. **a-c** Distribution of high and low risk based on all lncRNAs, differentially expressed lncRNAs, and the risk model

We extracted the coefficients and expression of 4 lncRNAs and calculated risk scores with the following equation: risk score = (0.459) *AC138430.1 + (0.004) *AGAP2-AS1 + (0.096) *AP001783.1 + (0.010) *AP005233.2. Depending on the median risk score, we divided all patients into high- and low-risk groups (*n* = 16 and 17, respectively). Corresponding survival condition plots showed that the high-risk group had higher mortality than the low-risk group (Fig. 2a-b), and the related gene expression patterns of four lncRNAs in the two risk groups are shown in Fig. 2c. The Kaplan-Meier survival curve revealed that patients with high risk scores had a significantly shorter OS (Fig. 2d). The AUCs were 0.952, 0.909, and 0.882 at 1, 2, and 3 years, respectively (Fig. 2e), and the C-index was 0.808, which demonstrated that this signature performed well as a predictor of prognosis. The PCA results showed a clear distribution of high and low risk on both sides based on the risk model, which could well classify patients into high- and low-risk groups compared to the other two approaches (Fig. 3a-c). qRT-PCR analysis showed the similar trends of four lncRNAs expression compared to differentially expression analysis (Supplementary Figure S1), which revealed all four lncRNAs were differentially expressed between the tumour and normal tissue in an independent cohort and may involve in the tumorigenesis of iCCA.

### Co-expression prediction and functional enrichment analysis

**Fig. 4:**
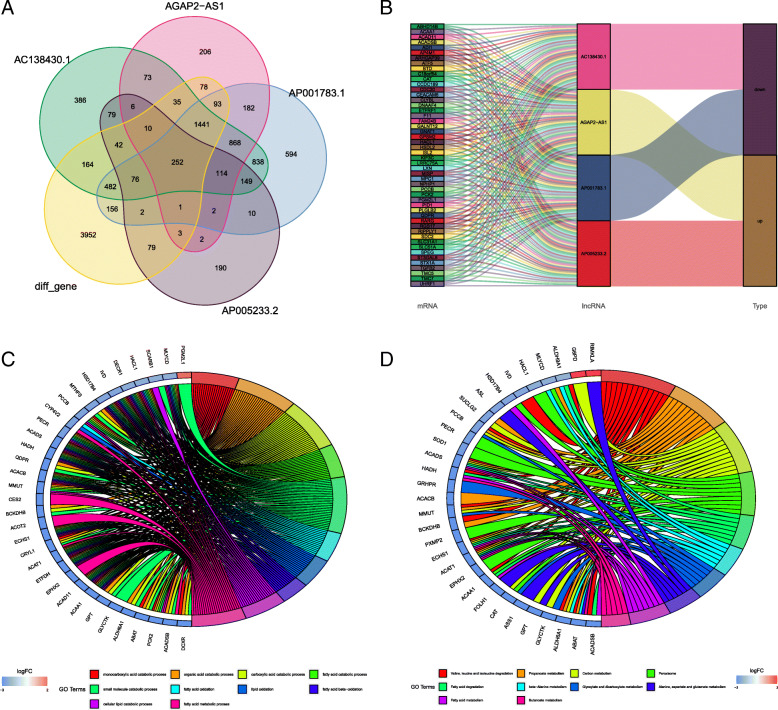
**a** Venn diagram of the intersection of co-expressed genes. **b** Association of mRNAs with lncRNAs and regulated types. **c** GO enrichment analysis. **d** KEGG pathway enrichment analysis

We used the co-expression method to predict the highly correlated PCGs of four DE-lncRNAs. A total of 252 differentially expressed PCGs were identified (Fig. 4a), which had a high correlation with all four lncRNAs (coef> 0.5, *p* < 0.05). Alluvial plot of PCGs illustrating associations with lncRNAs and survival state (Fig. 4b). The list of differentially expressed genes and PCGs is shown in Supplementary Tables S2–3. The GO and KEGG analyses showed that the function of PCGs was highly enriched in metabolism-related processes (Fig. 4b-c). The results showed that the four DE-lncRNAs were extensively involved in the metabolism-related processes of tumourigenesis.

### Gene set enrichment analysis

**Fig. 5:**
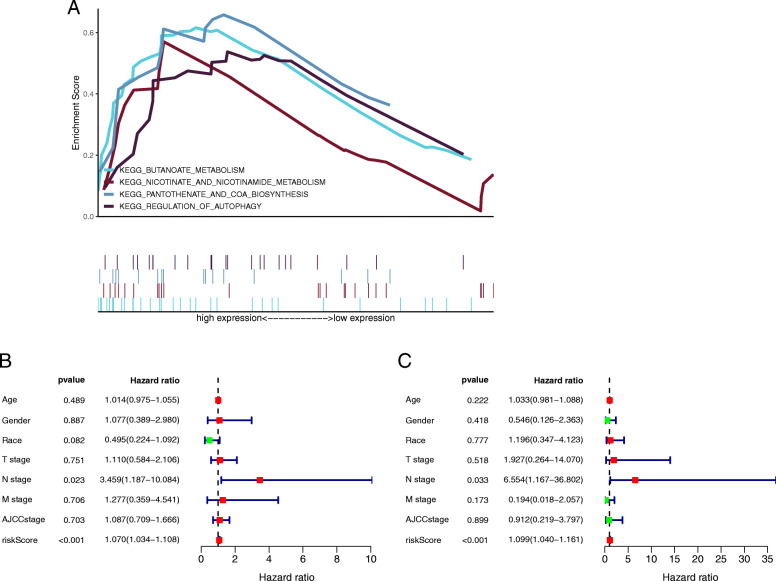
**a** Four crucially enriched pathways were displayed by using gene set enrichment analysis. **b-c** Forest plot of univariate and multivariate regression analyses

Based on these results, the prognostic signature has been shown to have good predictive power for OS. To further explore the potential mechanisms of the prognostic signature, we performed GSEA in the high-risk group. As expected, the enriched results showed that the signature had a strong correlation with metabolism in tumourigenesis (*p* < 0.05), which was similar to the pathways of co-expression target genes. The key enrichment pathways of the prognostic signature were “nicotinate and nicotinamide metabolism”, “butanoate metabolism”, “regulation of autophagy”, and “pantothenate and COA biosynthesis” (Fig. 5a). The detailed GSEA results are shown in Supplementary Figure S2.

### Independent prognosis analysis

The clinical data containing 33 patients with follow-up were collected from the TCGA database, as shown in Table 2. This prognostic signature was verified to be an independent prognostic indicator using Cox regression analyses, as shown in Fig. 5b-c, and the risk score and N stage were significantly correlated with the OS of patients in both univariate and multivariate regression analyses (*p* < 0.05). The results demonstrated that they had independent predictability.

**Table 2:** Clinicopathologic characteristics of patients with intrahepatic cholangiocarcinoma

Variables	N (33)	%
Age	66 (57)	
Sex		
Female	19	0.58
Male	14	0.42
Race		
Asian	3	0.09
Black or African American	2	0.06
White	28	0.85
T stage		
T1	18	0.55
T2	10	0.30
T3	5	0.15
N stage		
N0	25	0.76
N1&NX	8	0.24
M stage		
M0	27	0.82
M1&MX	6	0.18
AJCC stage		
I	18	0.55
II	9	0.27
III	1	0.03
IV	5	0.15
Status		
Alive	17	0.52
Dead	16	0.48
Survival time	1.92 (1.10)	

### Construction and verification of the nomogram

**Fig. 6:**
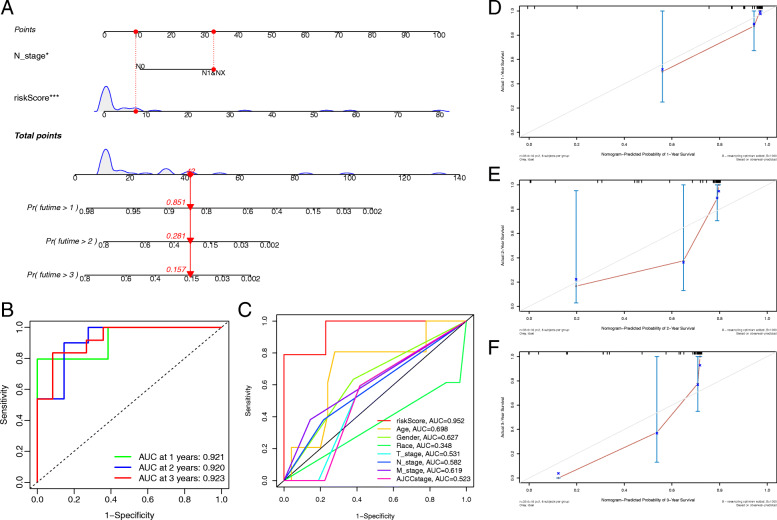
**a** Nomogram for predicting OS at 1, 2, and 3 years. **b** Time-dependent ROC curves of nomogram prediction at 1, 3, and 5 years. **c** The AUC for the risk score and clinical features. **d-f** Calibration curves showing the probability of 1-, 2-, and 3-year OS between the nomogram prediction and practical observation in the TCGA dataset

Next, the above independent predictors were integrated into a nomogram for predicting 1-, 2-, and 3-year survival rates (Fig. 6a). Based on the nomogram score, the 1-, 2-, and 3-year survival rates of patients could be well predicted according to their nomogram scores. The AUCs at 1, 2, and 3 years were 0.912, 0.920, and 0.923, respectively, in the nomogram prediction (Fig. 6b). The multivariable ROC curve demonstrated that the risk score had the best predictive capability (Fig. 6c). In addition, the calibration curves showed good agreement between the predicted survival rate and the actual survival rate at 1, 2, and 3 years (Fig. 6d-f). All the results suggested that the nomogram had good predictive accuracy and clinical application prospects.

## Discussion

Like most malignant tumours, the diagnosis and prognosis of patients with intrahepatic cholangiocarcinoma remain relatively poor, even though various treatments have been gradually improved and optimally applied [1]. In recent years, exploring tumour biomarkers has become an increasingly popular field in tumour therapy, which has greatly aroused the interest of researchers [5]. Meanwhile, with the extensive development of sequencing technology and the deepening of transcriptomics, lncRNAs have gradually entered the perspective of researchers [6, 17, 18]. Recently, many studies have demonstrated that lncRNAs are not “transcriptional noise”, as originally thought, but that they play roles as oncogenes or tumour suppressor genes [7] and act as competing endogenous RNAs (ceRNAs) to regulate key target genes in tumour development [19, 20]. Previous studies have identified a variety of lncRNAs involved in tumour metabolism, autophagy, and immune-related processes [21–24], while many researchers have developed various predictive models based on prognosis-related lncRNAs for application [25–27]. We are very interested in pathways related to tumour metabolism and would like to develop a predictive model for intrahepatic cholangiocarcinoma. Zhang et al.’s study identified a ceRNA regulation network containing 25 lncRNAs that were highly involved in bone metabolism-related biological processes and corresponding pathways [28]. Huang et al. also identified 9 lncRNAs that may be mainly involved in metabolism-related pathways of lung squamous cell carcinoma [29]. However, fewer lncRNAs have been developed for metabolism-related processes in intrahepatic cholangiocarcinoma, and the identification of more prognostic lncRNAs and related models is urgently needed to provide novel therapeutic targets in intrahepatic cholangiocarcinoma.

In this study, four survival-related and metabolism-related lncRNAs (AC138430.1, AGAP2-AS1, AP001783.1, and AP005233.2) were identified for prognosis prediction in patients with intrahepatic cholangiocarcinoma based on transcriptome data from public databases, and were verified in an independent cohort via quantitative real-time PCR. It shows these four lncRNAs may highly involve in the tumorigenesis of iCCA.

Recently, lncRNA AGAP2-AS1 (AGAP2 antisense RNA 1) has been reported to play crucial roles in some tumours, such as colorectal cancer [30], pancreatic cancer [31], and breast cancer [32]. It is extensively involved in important processes of tumourigenesis and development and participates in the expression process of target genes as a ceRNA. However, to our knowledge, high-quality studies to explore the potential mechanism of AGAP2-AS1 in intrahepatic cholangiocarcinoma are lacking. Hence, studying the correlation of AGAP2-AS1 with tumourigenesis in intrahepatic cholangiocarcinoma is significantly meaningful. The lncRNA AP005233.2 has only been identified as a key lncRNA in pathway crosstalk of lung adenocarcinoma [33]. Consequently, the mechanism of AP005233.2 in intrahepatic cholangiocarcinoma or other tumours also needs further investigation. Notably, no study has explored the mechanisms of lncRNA AC138430.1 and AP001783.1 in tumours. Therefore, their pivotal roles in intrahepatic cholangiocarcinoma are also worth revealing in future studies for the first time. In addition, according to whole genome sequencing, we identified the potential PCGs of the four lncRNAs using the co-expression method, and these expression states of PCGs may be upregulated or downregulated via the lncRNA-mRNA regulation network. Next, we performed GO and KEGG analyses to reveal a potential mechanism. The results showed that these four lncRNAs were extensively involved in metabolic processes. Previous studies have demonstrated that metabolism is highly related to tumourigenesis and metastasis of intrahepatic cholangiocarcinoma [34, 35], such as the fatty acid synthesis pathway [36]. Zhang et al. revealed that fatty acid-related metabolism plays a crucial role in the tumourigenesis of intrahepatic cholangiocarcinoma [37]. Based on four metabolism-related lncRNAs, we developed a prognostic signature with independent predictive capability using Cox regression analysis. The AUCs were 0.952, 0.909, and 0.882 at 1, 2, and 3 years, respectively, and the C-index was 0.808, which showed that our prognostic signature had good predictive accuracy. The multi-ROC curve showed that the signature had better predictive capability than other clinicopathologic characteristics, such as AJCC stage, so we could predict the patient’s prognosis using this signature. The PCA results showed that the signature including four lncRNAs could well distinguish high- and low-risk patients with intrahepatic cholangiocarcinoma to guide clinical grouping. Meanwhile, as we expected, GSEA also revealed that the signature containing four lncRNAs was highly related to metabolic processes, such as the nicotinate and nicotinamide metabolism pathway; therefore, these lncRNAs would have key effectiveness in tumour metabolism. Finally, we combined the two independent prognostic factors (N stage and risk score) to construct a nomogram for clinical application. The corresponding results also indicated that this nomogram had good discrimination capability. The calibration curves showed that there were good discriminative and calibration capabilities for this nomogram.

In addition, some limitations must also be noted in this study. First, all the data in our study were obtained from public databases to perform the retrospective analysis, and selection bias is inevitable. Secondly, our study included the number of prospective samples may be insufficient, thus, further large prospective cohort studies must be implemented to confirm the effectiveness of our prognostic signature. Then, due to the different sequencing methods in the TCGA and GEO databases, the standardization process was difficult to unify in our study, so we only completed the differential expression analysis for screening the overlapping DE-lncRNAs. But notably, our study validated the differential expression of these four DE-lncRNAs in an independent cohort using qRT-PCR, so them could be considered as a valuable predictive factor.

## Conclusion

We identified and validated a 4-lncRNA (AC138430.1, AGAP2-AS1, AP001783.1, and AP005233.2) prognostic signature that had a good predictive capability for prognosis in patients with intrahepatic cholangiocarcinoma. Meanwhile, the signature was highly related to metabolic pathways, which helps researchers deeply understand the correlation of metabolism with tumourigenesis. Hopefully, it will provide a new perspective for exploring biomarkers in the tumour metabolic microenvironment of intrahepatic cholangiocarcinoma.

## Supplementary Information

**Additional file 1: Supplementary Fig. S1.** A-D The results of quantitative real-time PCR showed that relative expression level of 4 key lncRNAs between tumor and normal tissue. * *P* < 0.05; ** *P* < 0.01; *** *P* < 0.001. **Supplementary Fig. S2** A-D Four detailed gene set enrichment pathways in high-risk group. **Table S1.** Primers used for quantitative real-time PCR. **Table S2.** Correlation analasisi of protein coding gene with each lncRNA. **Table S3.** Differentially expressed protein coding gene.

## Data Availability

The datasets downloaded for supporting the results of this article are publicly available at the TCGA (https://portal.gdc.cancer.gov/) and GEO (https://www.ncbi.nlm.nih.gov/geo/).

## Abbreviations

AJCC: American Joint Committee on Cancer
AUC: The area under curve
OS: Overall survival
GEO: Gene Expression Omnibus
GO: Gene Ontology
iCCA: Intrahepatic cholangiocarcinoma
KEGG: Kyoto Encyclopedia of Genes and Genomes
ROC: Receiver operating characteristic
TCGA: The Cancer Genome Atlas
PCGs: Protein-coding genes
PCA: Principal component analysis
GSEA: Gene set enrichment analysis
