# Supplementary material for: A metabolism-related 4-lncRNA prognostic signature and corresponding mechanisms in intrahepatic cholangiocarcinoma
Source: BMC Cancer. 2021 May 25;21:608. doi: 10.1186/s12885-021-08322-5 (PMC8152356; doi:10.1186/s12885-021-08322-5)
Supplement: Supplementary file 1 — Additional file 1: Supplementary Fig. S1. A-D The results of quantitative real-time PCR showed that relative expression level of 4 key lncRNAs between tumor and normal tissue. * P < 0.05; ** P < 0.01; *** P < 0.001. Supplementary Fig. S2 A-D Four detailed gene set enrichment pathways in high-risk group. Table S1. Primers used for quantitative real-time PCR. Table S2. Correlation analasisi of protein coding gene with each lncRNA. Table S3. Differentially expressed protein coding gene. [file 12885_2021_8322_MOESM1_ESM.docx]

Supplementary Materials

**Title:**

A Metabolism-related 4-lncRNA prognostic signature and corresponding mechanisms in intrahepatic cholangiocarcinoma

**Running Title:**

A lncRNA-prognostic signature in intrahepatic cholangiocarcinoma

**Authors:**

Wenbo Zou^1,2,3,4†^, Zizheng Wang^2,3,4†^, Fei wang^2,3,4†^, Lincheng li^2,3,4^, Rong Liu ^2,3,4*^, Minggen Hu^2,3,4*^

**Affiliations:**

1.Medical School of Chinese PLA

2.Faculty of Hepato-Pancreato-Biliary Surgery, Chinese PLA General Hospital, Beijing, China.

3.Institute of Hepatobiliary Surgery of Chinese PLA, Beijing, China.

4.Key Laboratory of Digital Hepetobiliary Surgery, PLA, Beijing, China.

**^†^Wenbo Zou, Zizheng Wang, and Fei Wang contributed equally to this work**

**Corresponding author:**

*Prof. Minggen Hu, Faculty of Hepato-Pancreato-Biliary Surgery, Chinese PLA General Hospital, No.28 Fuxing Road, Haidian District, 100853, Beijing, China. E-mail: [hmg301@126.com](mailto:hmg301@126.com)

*Prof. Rong Liu, Faculty of Hepato-Pancreato-Biliary Surgery, Chinese PLA General Hospital, No.28 Fuxing Road, Haidian District, 100853, Beijing, China. E-mail: [liurong301@126.com](mailto:liurong301@126.com)

**Supplementary Figure S1**


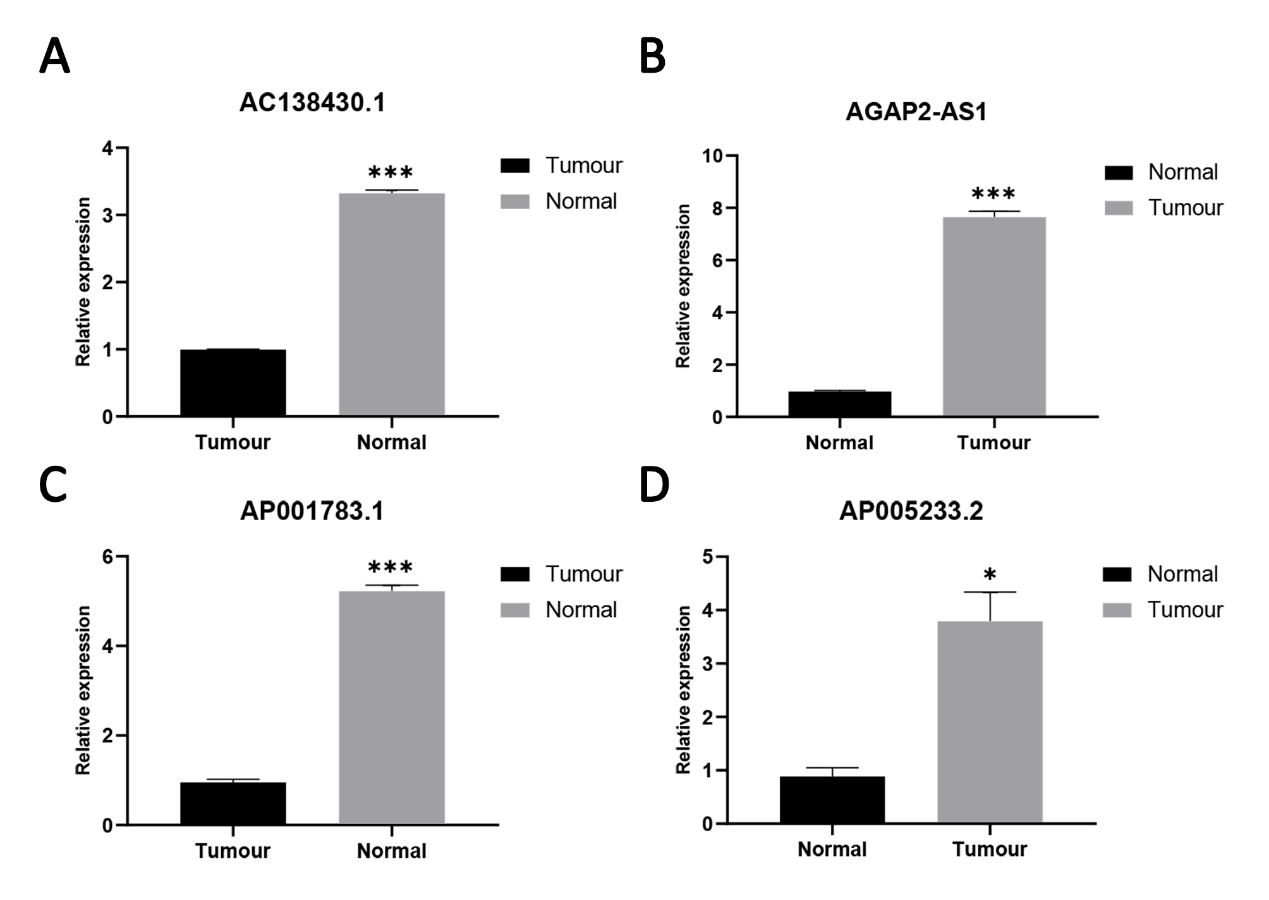


Supplementary Fig.S1 A-D The results of quantitative real-time PCR showed that relative expression level of 4 key lncRNAs between tumor and normal tissue. * *P* < 0.05; ** *P* < 0.01; *** *P* < 0.001.

**Supplementary Figure S2**


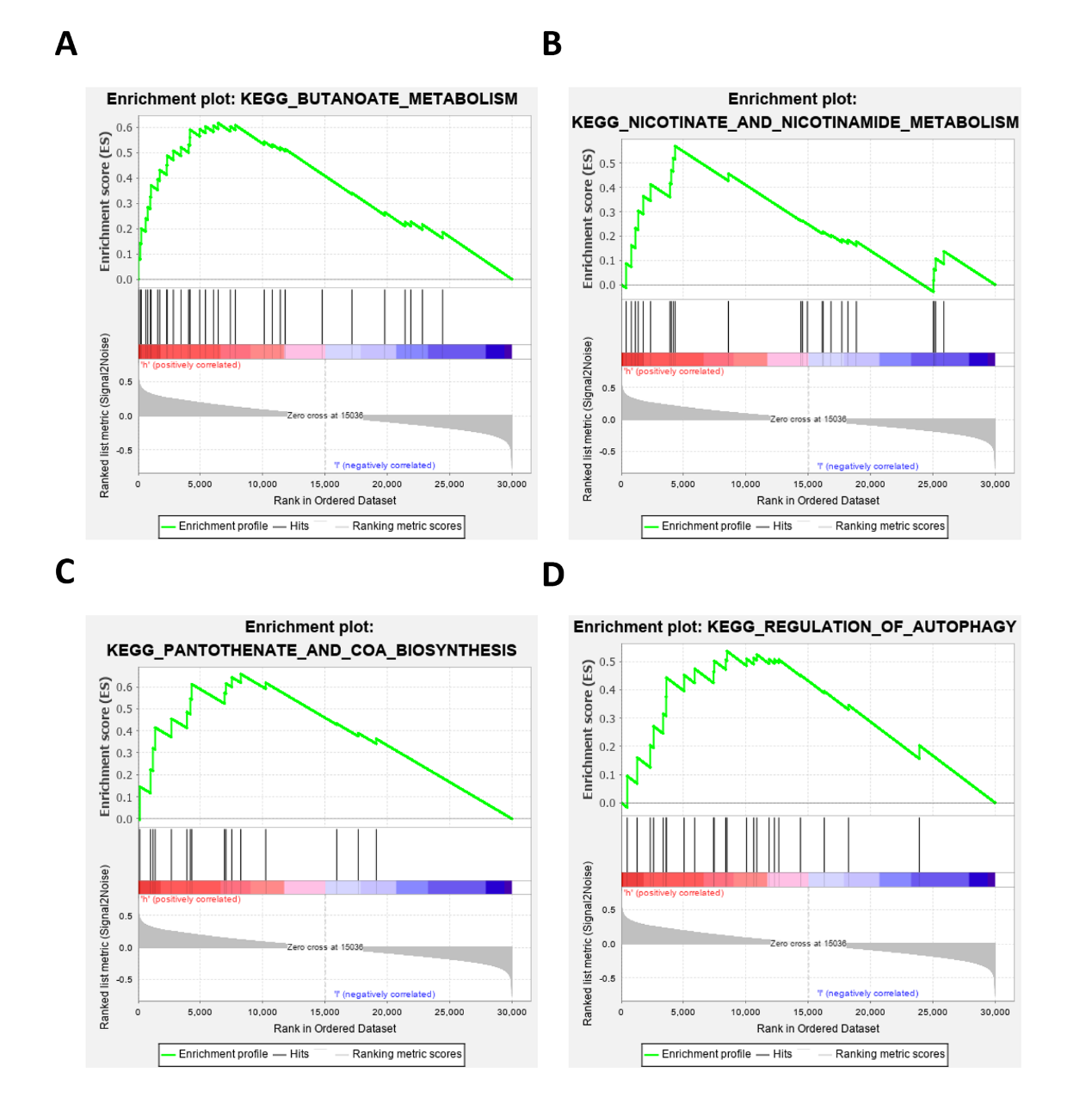


Supplementary Fig.S2 A-D Four detailed gene set enrichment pathways in high-risk group.

**Supplementary Table S1**

| **Table S1 Primers used for quantitative real-time PCR** | | |
| --- | --- | --- |
| **GeneName** | **Direction** | **Sequences (5′–3′)** |
| AGAP2-AS1 | Forward | CACAGCATTGCAGACTGGCTA |
| AGAP2-AS1 | Reverse | CCCTGCTTGAACTCTGTCCCTTA |
| AC138430.1 | Forward | TGGACCTCATCATCGCTCTTCAG |
| AC138430.1 | Reverse | CAACCAGGGAAGGTGAGAGCATA |
| AP001783.1 | Forward | TTCCGGACCTGGGATTCTGAAA |
| AP001783.1 | Reverse | GAACCAGCAACACAGATGTTCCC |
| AP005233.2 | Forward | GAGAATTGTGCCTGCCCAGC |
| AP005233.2 | Reverse | TCCCCACACTCCACAAACCC |
| β-actin | Forward | CATGTACGTTGCTATCCAGGC |
| β-actin | Reverse | CTCCTTAATGTCACGCACGAT |

**Supplementary Table S2**

**Table S2 correlation analasisi of protein coding gene with each lncRNA**

| **AGAP2-AS1** | | | | |
| --- | --- | --- | --- | --- |
| **lncRNA** | **gene** | **geneType** | **cor** | **pvalue** |
| AGAP2-AS1 | SPACA6 | protein_coding | 0.53 | 0.000358062 |
| AGAP2-AS1 | NAGS | protein_coding | -0.52 | 0.000564338 |
| AGAP2-AS1 | CEACAM6 | protein_coding | 0.56 | 0.000129802 |
| AGAP2-AS1 | SH3BGRL2 | protein_coding | -0.51 | 0.000670915 |
| AGAP2-AS1 | TRIM24 | protein_coding | -0.56 | 0.000127658 |
| AGAP2-AS1 | NUF2 | protein_coding | 0.63 | 8.45E-06 |
| AGAP2-AS1 | HSDL2 | protein_coding | -0.64 | 8.18E-06 |
| AGAP2-AS1 | FGGY | protein_coding | -0.55 | 0.000181732 |
| AGAP2-AS1 | ADRA2B | protein_coding | -0.69 | 7.28E-07 |
| AGAP2-AS1 | KRT80 | protein_coding | 0.58 | 6.49E-05 |
| AGAP2-AS1 | POLA2 | protein_coding | 0.70 | 3.06E-07 |
| AGAP2-AS1 | SEC14L6 | protein_coding | 0.54 | 0.000304287 |
| AGAP2-AS1 | ALDH2 | protein_coding | -0.59 | 5.69E-05 |
| AGAP2-AS1 | HSD17B13 | protein_coding | -0.59 | 4.70E-05 |
| AGAP2-AS1 | KLF9 | protein_coding | -0.52 | 0.000568977 |
| AGAP2-AS1 | AZGP1 | protein_coding | -0.51 | 0.000663687 |
| AGAP2-AS1 | EPS8L3 | protein_coding | 0.67 | 1.74E-06 |
| AGAP2-AS1 | MYEF2 | protein_coding | 0.67 | 1.73E-06 |
| AGAP2-AS1 | NR1I3 | protein_coding | -0.63 | 1.02E-05 |
| AGAP2-AS1 | NSUN5 | protein_coding | 0.57 | 0.000100362 |
| AGAP2-AS1 | ABHD11 | protein_coding | 0.58 | 8.20E-05 |
| AGAP2-AS1 | GDF2 | protein_coding | -0.58 | 7.18E-05 |
| AGAP2-AS1 | TMC5 | protein_coding | 0.52 | 0.000534146 |
| AGAP2-AS1 | UPB1 | protein_coding | -0.57 | 0.00011238 |
| AGAP2-AS1 | ORC6 | protein_coding | 0.77 | 3.87E-09 |
| AGAP2-AS1 | ALAS1 | protein_coding | -0.68 | 1.25E-06 |
| AGAP2-AS1 | METTL14 | protein_coding | -0.53 | 0.000331493 |
| AGAP2-AS1 | STAMBP | protein_coding | 0.55 | 0.000223151 |
| AGAP2-AS1 | DCK | protein_coding | 0.51 | 0.000757334 |
| AGAP2-AS1 | CIT | protein_coding | 0.56 | 0.000128573 |
| AGAP2-AS1 | NEK2 | protein_coding | 0.67 | 2.00E-06 |
| AGAP2-AS1 | DAND5 | protein_coding | 0.60 | 3.93E-05 |
| AGAP2-AS1 | RAP2C | protein_coding | -0.63 | 1.01E-05 |
| AGAP2-AS1 | ADORA2A | protein_coding | -0.57 | 0.000108292 |
| AGAP2-AS1 | SLC25A44 | protein_coding | -0.64 | 6.30E-06 |
| AGAP2-AS1 | RHPN1 | protein_coding | 0.61 | 2.00E-05 |
| AGAP2-AS1 | TRPC5 | protein_coding | -0.60 | 2.92E-05 |
| AGAP2-AS1 | RABL6 | protein_coding | 0.62 | 1.46E-05 |
| AGAP2-AS1 | C14orf93 | protein_coding | 0.57 | 0.000101473 |
| AGAP2-AS1 | RNPEPL1 | protein_coding | 0.64 | 5.70E-06 |
| AGAP2-AS1 | ZNF680 | protein_coding | -0.66 | 2.91E-06 |
| AGAP2-AS1 | NCAM2 | protein_coding | -0.60 | 3.52E-05 |
| AGAP2-AS1 | DGKH | protein_coding | 0.52 | 0.000431405 |
| AGAP2-AS1 | EGR1 | protein_coding | -0.56 | 0.000158959 |
| AGAP2-AS1 | NDOR1 | protein_coding | 0.56 | 0.000123784 |
| AGAP2-AS1 | DAB1 | protein_coding | -0.58 | 6.24E-05 |
| AGAP2-AS1 | S100PBP | protein_coding | 0.53 | 0.000385739 |
| AGAP2-AS1 | F11 | protein_coding | -0.58 | 6.74E-05 |
| AGAP2-AS1 | HMGCL | protein_coding | -0.58 | 6.75E-05 |
| AGAP2-AS1 | EPM2AIP1 | protein_coding | -0.59 | 4.92E-05 |
| AGAP2-AS1 | AFM | protein_coding | -0.60 | 3.86E-05 |
| AGAP2-AS1 | SMAGP | protein_coding | 0.65 | 4.35E-06 |
| AGAP2-AS1 | DRD1 | protein_coding | -0.60 | 2.89E-05 |
| AGAP2-AS1 | NDUFAF6 | protein_coding | -0.51 | 0.000620224 |
| AGAP2-AS1 | DLGAP5 | protein_coding | 0.69 | 5.85E-07 |
| AGAP2-AS1 | PRKAG2 | protein_coding | -0.61 | 1.92E-05 |
| AGAP2-AS1 | ABHD14B | protein_coding | -0.64 | 6.37E-06 |
| AGAP2-AS1 | TMC7 | protein_coding | 0.51 | 0.000629007 |
| AGAP2-AS1 | PROB1 | protein_coding | 0.58 | 7.07E-05 |
| AGAP2-AS1 | HSDL1 | protein_coding | 0.58 | 8.25E-05 |
| AGAP2-AS1 | GREB1 | protein_coding | -0.51 | 0.000598609 |
| AGAP2-AS1 | BTD | protein_coding | -0.61 | 2.18E-05 |
| AGAP2-AS1 | BMS1 | protein_coding | 0.55 | 0.000198646 |
| AGAP2-AS1 | DLG3 | protein_coding | 0.69 | 4.97E-07 |
| AGAP2-AS1 | DCTN2 | protein_coding | 0.76 | 7.15E-09 |
| AGAP2-AS1 | ESS2 | protein_coding | 0.59 | 4.58E-05 |
| AGAP2-AS1 | C12orf49 | protein_coding | 0.55 | 0.000178382 |
| AGAP2-AS1 | GLTP | protein_coding | 0.54 | 0.000278923 |
| AGAP2-AS1 | ZNF579 | protein_coding | 0.52 | 0.000483176 |
| AGAP2-AS1 | SESN1 | protein_coding | -0.52 | 0.000522895 |
| AGAP2-AS1 | ERFE | protein_coding | 0.56 | 0.000144795 |
| AGAP2-AS1 | HDGFL1 | protein_coding | 0.56 | 0.000121537 |
| AGAP2-AS1 | DKK1 | protein_coding | 0.54 | 0.00027953 |
| AGAP2-AS1 | MAN2B2 | protein_coding | -0.52 | 0.000543775 |
| AGAP2-AS1 | GAL3ST4 | protein_coding | 0.64 | 6.86E-06 |
| AGAP2-AS1 | TMEM65 | protein_coding | 0.52 | 0.000512052 |
| AGAP2-AS1 | PORCN | protein_coding | 0.58 | 6.64E-05 |
| AGAP2-AS1 | ITCH | protein_coding | -0.68 | 1.10E-06 |
| AGAP2-AS1 | ABCD4 | protein_coding | -0.57 | 8.69E-05 |
| AGAP2-AS1 | ARF3 | protein_coding | 0.59 | 5.90E-05 |
| AGAP2-AS1 | GNG13 | protein_coding | 0.51 | 0.000709624 |
| AGAP2-AS1 | TM6SF2 | protein_coding | -0.53 | 0.000411404 |
| AGAP2-AS1 | EPHA6 | protein_coding | 0.53 | 0.000416361 |
| AGAP2-AS1 | ICA1L | protein_coding | 0.71 | 1.55E-07 |
| AGAP2-AS1 | SLC9A3R2 | protein_coding | -0.63 | 1.12E-05 |
| AGAP2-AS1 | CLYBL | protein_coding | -0.57 | 0.000117094 |
| AGAP2-AS1 | BHLHE41 | protein_coding | 0.54 | 0.000279247 |
| AGAP2-AS1 | TSPAN17 | protein_coding | 0.65 | 4.60E-06 |
| AGAP2-AS1 | CMTM3 | protein_coding | 0.67 | 1.62E-06 |
| AGAP2-AS1 | DZANK1 | protein_coding | 0.63 | 1.24E-05 |
| AGAP2-AS1 | DNM1L | protein_coding | 0.52 | 0.000526178 |
| AGAP2-AS1 | ORM2 | protein_coding | -0.62 | 1.34E-05 |
| AGAP2-AS1 | NBPF12 | protein_coding | -0.59 | 4.54E-05 |
| AGAP2-AS1 | H3C4 | protein_coding | 0.51 | 0.000665089 |
| AGAP2-AS1 | CDCA7L | protein_coding | 0.53 | 0.000418096 |
| AGAP2-AS1 | ORM1 | protein_coding | -0.56 | 0.00012696 |
| AGAP2-AS1 | ITIH2 | protein_coding | -0.57 | 0.000107133 |
| AGAP2-AS1 | RREB1 | protein_coding | -0.63 | 1.15E-05 |
| AGAP2-AS1 | SHF | protein_coding | -0.52 | 0.000516185 |
| AGAP2-AS1 | TMEM123 | protein_coding | -0.56 | 0.000125282 |
| AGAP2-AS1 | ARID3C | protein_coding | -0.64 | 6.81E-06 |
| AGAP2-AS1 | C3orf80 | protein_coding | 0.50 | 0.000795993 |
| AGAP2-AS1 | DTYMK | protein_coding | 0.67 | 1.86E-06 |
| AGAP2-AS1 | CYTH2 | protein_coding | 0.58 | 8.35E-05 |
| AGAP2-AS1 | H2BU1 | protein_coding | 0.66 | 2.79E-06 |
| AGAP2-AS1 | STX17 | protein_coding | -0.58 | 7.89E-05 |
| AGAP2-AS1 | BMT2 | protein_coding | 0.51 | 0.000591523 |
| AGAP2-AS1 | AP1M2 | protein_coding | 0.55 | 0.000218598 |
| AGAP2-AS1 | TINAGL1 | protein_coding | 0.65 | 4.53E-06 |
| AGAP2-AS1 | MT1F | protein_coding | -0.57 | 8.80E-05 |
| AGAP2-AS1 | DPCD | protein_coding | 0.60 | 3.57E-05 |
| AGAP2-AS1 | KLHL15 | protein_coding | -0.66 | 2.95E-06 |
| AGAP2-AS1 | GAB2 | protein_coding | 0.50 | 0.000824602 |
| AGAP2-AS1 | TOP2A | protein_coding | 0.70 | 4.22E-07 |
| AGAP2-AS1 | YBX3 | protein_coding | 0.55 | 0.000218983 |
| AGAP2-AS1 | GNAT1 | protein_coding | -0.59 | 5.07E-05 |
| AGAP2-AS1 | UGT2B10 | protein_coding | -0.56 | 0.000127779 |
| AGAP2-AS1 | DKC1 | protein_coding | 0.53 | 0.000375028 |
| AGAP2-AS1 | YKT6 | protein_coding | 0.70 | 3.26E-07 |
| AGAP2-AS1 | MTHFD1 | protein_coding | -0.64 | 7.81E-06 |
| AGAP2-AS1 | SSB | protein_coding | 0.61 | 2.50E-05 |
| AGAP2-AS1 | ERMP1 | protein_coding | 0.54 | 0.000244238 |
| AGAP2-AS1 | TGFB2 | protein_coding | 0.56 | 0.000147532 |
| AGAP2-AS1 | FAM126B | protein_coding | -0.61 | 2.52E-05 |
| AGAP2-AS1 | MTFR2 | protein_coding | 0.71 | 1.75E-07 |
| AGAP2-AS1 | USP18 | protein_coding | -0.51 | 0.000667304 |
| AGAP2-AS1 | ACADSB | protein_coding | -0.68 | 8.29E-07 |
| AGAP2-AS1 | ENO3 | protein_coding | -0.57 | 0.00011683 |
| AGAP2-AS1 | SLC25A18 | protein_coding | -0.63 | 1.13E-05 |
| AGAP2-AS1 | KMO | protein_coding | -0.70 | 4.17E-07 |
| AGAP2-AS1 | HGF | protein_coding | -0.58 | 6.25E-05 |
| AGAP2-AS1 | SNED1 | protein_coding | -0.58 | 7.42E-05 |
| AGAP2-AS1 | DTD1 | protein_coding | 0.75 | 1.22E-08 |
| AGAP2-AS1 | NUP93 | protein_coding | 0.65 | 5.07E-06 |
| AGAP2-AS1 | MT1A | protein_coding | -0.59 | 5.71E-05 |
| AGAP2-AS1 | ATP1A1 | protein_coding | 0.58 | 6.17E-05 |
| AGAP2-AS1 | HSF2BP | protein_coding | 0.58 | 7.96E-05 |
| AGAP2-AS1 | AMT | protein_coding | -0.67 | 2.08E-06 |
| AGAP2-AS1 | MASP2 | protein_coding | -0.63 | 1.11E-05 |
| AGAP2-AS1 | CYP3A4 | protein_coding | -0.58 | 7.44E-05 |
| AGAP2-AS1 | GPRIN1 | protein_coding | 0.70 | 3.24E-07 |
| AGAP2-AS1 | STAT2 | protein_coding | -0.50 | 0.000831717 |
| AGAP2-AS1 | AC002316.1 | protein_coding | 0.65 | 3.90E-06 |
| AGAP2-AS1 | NPHP1 | protein_coding | 0.53 | 0.000366729 |
| AGAP2-AS1 | TXNRD2 | protein_coding | -0.57 | 0.00010401 |
| AGAP2-AS1 | SMYD5 | protein_coding | 0.70 | 4.02E-07 |
| AGAP2-AS1 | FRMD5 | protein_coding | 0.66 | 2.40E-06 |
| AGAP2-AS1 | ZNF14 | protein_coding | 0.52 | 0.000577066 |
| AGAP2-AS1 | AP3M2 | protein_coding | 0.58 | 6.42E-05 |
| AGAP2-AS1 | PLCB3 | protein_coding | 0.64 | 6.16E-06 |
| AGAP2-AS1 | CCNG1 | protein_coding | -0.56 | 0.000155213 |
| AGAP2-AS1 | RIPPLY1 | protein_coding | -0.52 | 0.000486445 |
| AGAP2-AS1 | BUB1B | protein_coding | 0.73 | 5.82E-08 |
| AGAP2-AS1 | RARG | protein_coding | 0.66 | 2.18E-06 |
| AGAP2-AS1 | C4orf3 | protein_coding | -0.53 | 0.000430951 |
| AGAP2-AS1 | SIRT5 | protein_coding | -0.53 | 0.000351725 |
| AGAP2-AS1 | FAM189B | protein_coding | 0.51 | 0.000739938 |
| AGAP2-AS1 | CFHR1 | protein_coding | -0.57 | 0.000119602 |
| AGAP2-AS1 | NTN3 | protein_coding | -0.66 | 2.17E-06 |
| AGAP2-AS1 | AGBL5 | protein_coding | 0.51 | 0.000645091 |
| AGAP2-AS1 | PAOX | protein_coding | -0.55 | 0.000186399 |
| AGAP2-AS1 | CPT2 | protein_coding | -0.61 | 2.02E-05 |
| AGAP2-AS1 | APPL2 | protein_coding | 0.56 | 0.00012608 |
| AGAP2-AS1 | VPS35 | protein_coding | 0.67 | 1.37E-06 |
| AGAP2-AS1 | CHTF18 | protein_coding | 0.59 | 5.01E-05 |
| AGAP2-AS1 | CYB561D1 | protein_coding | 0.59 | 5.13E-05 |
| AGAP2-AS1 | PGLYRP2 | protein_coding | -0.60 | 2.96E-05 |
| AGAP2-AS1 | HPD | protein_coding | -0.59 | 5.41E-05 |
| AGAP2-AS1 | HAUS1 | protein_coding | 0.68 | 8.44E-07 |
| AGAP2-AS1 | HAO2 | protein_coding | -0.59 | 4.30E-05 |
| AGAP2-AS1 | OASL | protein_coding | -0.53 | 0.00041653 |
| AGAP2-AS1 | NUGGC | protein_coding | -0.66 | 2.21E-06 |
| AGAP2-AS1 | MACROH2A1 | protein_coding | 0.68 | 1.25E-06 |
| AGAP2-AS1 | SEC14L3 | protein_coding | -0.68 | 1.09E-06 |
| AGAP2-AS1 | PCGF1 | protein_coding | 0.53 | 0.000425092 |
| AGAP2-AS1 | CHMP1A | protein_coding | 0.65 | 3.56E-06 |
| AGAP2-AS1 | E2F1 | protein_coding | 0.73 | 7.68E-08 |
| AGAP2-AS1 | ADGRF4 | protein_coding | 0.61 | 2.17E-05 |
| AGAP2-AS1 | RECQL4 | protein_coding | 0.66 | 2.50E-06 |
| AGAP2-AS1 | CXorf38 | protein_coding | 0.52 | 0.000560657 |
| AGAP2-AS1 | STX1A | protein_coding | 0.57 | 9.34E-05 |
| AGAP2-AS1 | STX16 | protein_coding | 0.54 | 0.000281086 |
| AGAP2-AS1 | PRR7 | protein_coding | 0.59 | 4.93E-05 |
| AGAP2-AS1 | HBS1L | protein_coding | -0.54 | 0.000251995 |
| AGAP2-AS1 | TMEM53 | protein_coding | -0.55 | 0.000218505 |
| AGAP2-AS1 | PEX3 | protein_coding | -0.60 | 3.29E-05 |
| AGAP2-AS1 | STX3 | protein_coding | 0.52 | 0.000511217 |
| AGAP2-AS1 | RAB41 | protein_coding | 0.64 | 5.55E-06 |
| AGAP2-AS1 | MRPS31 | protein_coding | -0.54 | 0.000239812 |
| AGAP2-AS1 | MNS1 | protein_coding | 0.61 | 2.45E-05 |
| AGAP2-AS1 | PFDN1 | protein_coding | 0.61 | 2.55E-05 |
| AGAP2-AS1 | ENPP7 | protein_coding | -0.53 | 0.000358433 |
| AGAP2-AS1 | ACOT12 | protein_coding | -0.59 | 5.55E-05 |
| AGAP2-AS1 | EHHADH | protein_coding | -0.64 | 8.37E-06 |
| AGAP2-AS1 | IKZF5 | protein_coding | -0.50 | 0.00084439 |
| AGAP2-AS1 | SFI1 | protein_coding | 0.57 | 0.000112166 |
| AGAP2-AS1 | LOXL2 | protein_coding | 0.61 | 2.03E-05 |
| AGAP2-AS1 | KCNJ11 | protein_coding | 0.52 | 0.000441783 |
| AGAP2-AS1 | UBE2Q2 | protein_coding | 0.63 | 1.24E-05 |
| AGAP2-AS1 | KCNQ3 | protein_coding | 0.65 | 4.04E-06 |
| AGAP2-AS1 | SLC13A3 | protein_coding | -0.69 | 5.14E-07 |
| AGAP2-AS1 | ZNF22 | protein_coding | -0.60 | 4.02E-05 |
| AGAP2-AS1 | SLC16A3 | protein_coding | 0.62 | 1.72E-05 |
| AGAP2-AS1 | SLC16A13 | protein_coding | -0.62 | 1.43E-05 |
| AGAP2-AS1 | TPX2 | protein_coding | 0.63 | 1.13E-05 |
| AGAP2-AS1 | TSC22D2 | protein_coding | -0.59 | 5.41E-05 |
| AGAP2-AS1 | OMA1 | protein_coding | -0.56 | 0.000135266 |
| AGAP2-AS1 | ATF5 | protein_coding | -0.64 | 6.06E-06 |
| AGAP2-AS1 | SULT2B1 | protein_coding | 0.58 | 7.14E-05 |
| AGAP2-AS1 | EPHA1 | protein_coding | -0.57 | 0.000114231 |
| AGAP2-AS1 | LAP3 | protein_coding | -0.66 | 2.73E-06 |
| AGAP2-AS1 | SYPL2 | protein_coding | -0.58 | 6.44E-05 |
| AGAP2-AS1 | MFGE8 | protein_coding | 0.58 | 6.43E-05 |
| AGAP2-AS1 | SLC25A47 | protein_coding | -0.54 | 0.00029978 |
| AGAP2-AS1 | ABHD13 | protein_coding | -0.56 | 0.000153204 |
| AGAP2-AS1 | E2F2 | protein_coding | 0.60 | 4.08E-05 |
| AGAP2-AS1 | SLC38A4 | protein_coding | -0.57 | 0.000102018 |
| AGAP2-AS1 | CDC20 | protein_coding | 0.71 | 1.85E-07 |
| AGAP2-AS1 | PACS1 | protein_coding | 0.61 | 2.00E-05 |
| AGAP2-AS1 | PNCK | protein_coding | 0.57 | 0.000108081 |
| AGAP2-AS1 | TESC | protein_coding | 0.66 | 3.40E-06 |
| AGAP2-AS1 | ZNF385B | protein_coding | -0.55 | 0.000209551 |
| AGAP2-AS1 | TSKU | protein_coding | -0.60 | 2.95E-05 |
| AGAP2-AS1 | SPRED3 | protein_coding | 0.71 | 1.61E-07 |
| AGAP2-AS1 | IBTK | protein_coding | -0.60 | 4.12E-05 |
| AGAP2-AS1 | ADI1 | protein_coding | -0.64 | 6.09E-06 |
| AGAP2-AS1 | SBK3 | protein_coding | 0.55 | 0.000196355 |
| AGAP2-AS1 | EIF5 | protein_coding | -0.59 | 4.38E-05 |
| AGAP2-AS1 | MELTF | protein_coding | 0.62 | 1.73E-05 |
| AGAP2-AS1 | XKR6 | protein_coding | 0.66 | 2.78E-06 |
| AGAP2-AS1 | SLCO2B1 | protein_coding | -0.61 | 2.12E-05 |
| AGAP2-AS1 | ORMDL2 | protein_coding | 0.50 | 0.000776205 |
| AGAP2-AS1 | STX6 | protein_coding | 0.52 | 0.000519717 |
| AGAP2-AS1 | ETNK2 | protein_coding | -0.66 | 2.99E-06 |
| AGAP2-AS1 | CCDC9B | protein_coding | 0.74 | 4.05E-08 |
| AGAP2-AS1 | ST3GAL6 | protein_coding | -0.57 | 0.000104883 |
| AGAP2-AS1 | ZBTB21 | protein_coding | -0.54 | 0.000250437 |
| AGAP2-AS1 | CYP2J2 | protein_coding | -0.53 | 0.00037296 |
| AGAP2-AS1 | BSG | protein_coding | 0.56 | 0.000135606 |
| AGAP2-AS1 | C6orf132 | protein_coding | 0.56 | 0.00016758 |
| AGAP2-AS1 | AKR1D1 | protein_coding | -0.63 | 9.08E-06 |
| AGAP2-AS1 | ZNF630 | protein_coding | 0.50 | 0.000800725 |
| AGAP2-AS1 | LHFPL2 | protein_coding | 0.53 | 0.000393813 |
| AGAP2-AS1 | PCCB | protein_coding | -0.57 | 9.46E-05 |
| AGAP2-AS1 | UHRF1 | protein_coding | 0.68 | 1.03E-06 |
| AGAP2-AS1 | SHANK3 | protein_coding | -0.56 | 0.000151707 |
| AGAP2-AS1 | RAB8A | protein_coding | -0.51 | 0.000654163 |
| AGAP2-AS1 | PSG9 | protein_coding | 0.53 | 0.000394836 |
| AGAP2-AS1 | TPRKB | protein_coding | 0.58 | 6.08E-05 |
| AGAP2-AS1 | MAP1LC3A | protein_coding | -0.56 | 0.000130207 |
| AGAP2-AS1 | MNX1 | protein_coding | 0.63 | 1.05E-05 |
| AGAP2-AS1 | HSD3B2 | protein_coding | -0.54 | 0.000266314 |
| AGAP2-AS1 | RFWD3 | protein_coding | 0.64 | 7.60E-06 |
| AGAP2-AS1 | CHST10 | protein_coding | 0.67 | 1.54E-06 |
| AGAP2-AS1 | CYP2C18 | protein_coding | -0.56 | 0.000129379 |
| AGAP2-AS1 | MINDY4 | protein_coding | 0.54 | 0.000245634 |
| AGAP2-AS1 | KCTD17 | protein_coding | 0.75 | 1.79E-08 |
| AGAP2-AS1 | KIF3C | protein_coding | 0.57 | 9.63E-05 |
| AGAP2-AS1 | ATP10A | protein_coding | 0.58 | 7.44E-05 |
| AGAP2-AS1 | CDC37 | protein_coding | 0.53 | 0.000378551 |
| AGAP2-AS1 | KIAA1522 | protein_coding | 0.52 | 0.000530599 |
| AGAP2-AS1 | NAA40 | protein_coding | 0.69 | 7.81E-07 |
| AGAP2-AS1 | FTSJ1 | protein_coding | 0.63 | 1.13E-05 |
| AGAP2-AS1 | EEF1A2 | protein_coding | 0.58 | 8.29E-05 |
| AGAP2-AS1 | PTK7 | protein_coding | 0.58 | 8.30E-05 |
| AGAP2-AS1 | CCDC34 | protein_coding | 0.76 | 1.10E-08 |
| AGAP2-AS1 | RDH5 | protein_coding | -0.52 | 0.000453552 |
| AGAP2-AS1 | PUS3 | protein_coding | -0.62 | 1.64E-05 |
| AGAP2-AS1 | COG3 | protein_coding | -0.58 | 8.49E-05 |
| AGAP2-AS1 | CCNF | protein_coding | 0.61 | 2.65E-05 |
| AGAP2-AS1 | CITED2 | protein_coding | -0.52 | 0.000545978 |
| AGAP2-AS1 | HEPACAM | protein_coding | -0.69 | 6.96E-07 |
| AGAP2-AS1 | HACL1 | protein_coding | -0.64 | 5.50E-06 |
| AGAP2-AS1 | LXN | protein_coding | 0.59 | 5.34E-05 |
| AGAP2-AS1 | PCLAF | protein_coding | 0.69 | 6.90E-07 |
| AGAP2-AS1 | IL6R | protein_coding | -0.67 | 1.50E-06 |
| AGAP2-AS1 | ENPP3 | protein_coding | -0.65 | 3.63E-06 |
| AGAP2-AS1 | SLC38A1 | protein_coding | 0.61 | 2.82E-05 |
| AGAP2-AS1 | MTSS2 | protein_coding | -0.61 | 2.54E-05 |
| AGAP2-AS1 | DNAJC9 | protein_coding | 0.59 | 5.20E-05 |
| AGAP2-AS1 | TCTN2 | protein_coding | 0.68 | 1.30E-06 |
| AGAP2-AS1 | ETFRF1 | protein_coding | -0.69 | 6.25E-07 |
| AGAP2-AS1 | DAPK3 | protein_coding | 0.60 | 2.98E-05 |
| AGAP2-AS1 | RCAN1 | protein_coding | -0.53 | 0.00031968 |
| AGAP2-AS1 | TRO | protein_coding | -0.58 | 8.49E-05 |
| AGAP2-AS1 | SEPHS2 | protein_coding | -0.52 | 0.000503829 |
| AGAP2-AS1 | SRSF5 | protein_coding | -0.66 | 2.59E-06 |
| AGAP2-AS1 | HSD17B6 | protein_coding | -0.58 | 7.71E-05 |
| AGAP2-AS1 | KRT10 | protein_coding | 0.52 | 0.000479297 |
| AGAP2-AS1 | GCDH | protein_coding | -0.65 | 4.29E-06 |
| AGAP2-AS1 | SPEG | protein_coding | 0.59 | 5.00E-05 |
| AGAP2-AS1 | MT-ND2 | protein_coding | -0.66 | 2.90E-06 |
| AGAP2-AS1 | PIEZO1 | protein_coding | 0.53 | 0.000382171 |
| AGAP2-AS1 | C12orf4 | protein_coding | 0.57 | 0.000103048 |
| AGAP2-AS1 | XYLB | protein_coding | -0.72 | 1.43E-07 |
| AGAP2-AS1 | GPR84 | protein_coding | 0.56 | 0.000139888 |
| AGAP2-AS1 | MT-ND1 | protein_coding | -0.68 | 1.20E-06 |
| AGAP2-AS1 | ZP1 | protein_coding | 0.56 | 0.000133663 |
| AGAP2-AS1 | DNAAF4 | protein_coding | 0.64 | 6.93E-06 |
| AGAP2-AS1 | FAM98B | protein_coding | 0.61 | 2.14E-05 |
| AGAP2-AS1 | MFSD2A | protein_coding | -0.60 | 3.67E-05 |
| AGAP2-AS1 | MAPKBP1 | protein_coding | 0.55 | 0.000200697 |
| AGAP2-AS1 | NANOS3 | protein_coding | 0.64 | 7.02E-06 |
| AGAP2-AS1 | CENPW | protein_coding | 0.61 | 2.67E-05 |
| AGAP2-AS1 | GPSM2 | protein_coding | 0.62 | 1.45E-05 |
| AGAP2-AS1 | KCTD2 | protein_coding | 0.70 | 3.30E-07 |
| AGAP2-AS1 | RASD2 | protein_coding | 0.62 | 1.49E-05 |
| AGAP2-AS1 | KBTBD6 | protein_coding | -0.53 | 0.000389105 |
| AGAP2-AS1 | B3GNT9 | protein_coding | 0.60 | 3.71E-05 |
| AGAP2-AS1 | C9orf72 | protein_coding | -0.53 | 0.000362885 |
| AGAP2-AS1 | USP54 | protein_coding | 0.50 | 0.000853207 |
| AGAP2-AS1 | DECR2 | protein_coding | -0.51 | 0.000637839 |
| AGAP2-AS1 | SSTR1 | protein_coding | -0.53 | 0.000401685 |
| AGAP2-AS1 | NXPH4 | protein_coding | 0.62 | 1.56E-05 |
| AGAP2-AS1 | PARP9 | protein_coding | -0.61 | 2.38E-05 |
| AGAP2-AS1 | CMTM4 | protein_coding | 0.57 | 9.33E-05 |
| AGAP2-AS1 | ITGB1BP1 | protein_coding | 0.65 | 5.22E-06 |
| AGAP2-AS1 | ORC2 | protein_coding | 0.51 | 0.000742996 |
| AGAP2-AS1 | AC024592.3 | protein_coding | -0.62 | 1.70E-05 |
| AGAP2-AS1 | FAAP24 | protein_coding | 0.74 | 3.07E-08 |
| AGAP2-AS1 | OLFML1 | protein_coding | -0.51 | 0.00063736 |
| AGAP2-AS1 | TLCD5 | protein_coding | 0.73 | 7.57E-08 |
| AGAP2-AS1 | GPD1 | protein_coding | -0.64 | 7.27E-06 |
| AGAP2-AS1 | LYRM1 | protein_coding | -0.58 | 8.26E-05 |
| AGAP2-AS1 | MAB21L3 | protein_coding | -0.50 | 0.000769782 |
| AGAP2-AS1 | SPOCK1 | protein_coding | 0.59 | 4.49E-05 |
| AGAP2-AS1 | DCTN5 | protein_coding | 0.64 | 5.90E-06 |
| AGAP2-AS1 | ZNF282 | protein_coding | 0.60 | 2.98E-05 |
| AGAP2-AS1 | KNOP1 | protein_coding | 0.64 | 6.76E-06 |
| AGAP2-AS1 | IQSEC1 | protein_coding | -0.54 | 0.000293663 |
| AGAP2-AS1 | ADAM28 | protein_coding | 0.60 | 3.86E-05 |
| AGAP2-AS1 | TTF2 | protein_coding | 0.63 | 1.11E-05 |
| AGAP2-AS1 | RASGRF1 | protein_coding | 0.56 | 0.000135229 |
| AGAP2-AS1 | IQGAP3 | protein_coding | 0.64 | 6.48E-06 |
| AGAP2-AS1 | CAT | protein_coding | -0.70 | 3.72E-07 |
| AGAP2-AS1 | C7 | protein_coding | -0.57 | 9.93E-05 |
| AGAP2-AS1 | COLGALT1 | protein_coding | 0.62 | 1.36E-05 |
| AGAP2-AS1 | P4HTM | protein_coding | 0.56 | 0.000164625 |
| AGAP2-AS1 | HMMR | protein_coding | 0.58 | 6.69E-05 |
| AGAP2-AS1 | TMEM151A | protein_coding | 0.60 | 4.04E-05 |
| AGAP2-AS1 | GSTA2 | protein_coding | -0.55 | 0.000177514 |
| AGAP2-AS1 | ZNF684 | protein_coding | -0.52 | 0.000439019 |
| AGAP2-AS1 | DNAL4 | protein_coding | 0.64 | 7.79E-06 |
| AGAP2-AS1 | AANAT | protein_coding | 0.51 | 0.000721142 |
| AGAP2-AS1 | PHF21A | protein_coding | 0.60 | 2.89E-05 |
| AGAP2-AS1 | ZNF711 | protein_coding | 0.54 | 0.000244957 |
| AGAP2-AS1 | XAGE3 | protein_coding | -0.69 | 7.57E-07 |
| AGAP2-AS1 | MRO | protein_coding | -0.64 | 7.96E-06 |
| AGAP2-AS1 | CAMK2N2 | protein_coding | 0.61 | 2.62E-05 |
| AGAP2-AS1 | SLC31A1 | protein_coding | -0.58 | 7.48E-05 |
| AGAP2-AS1 | ARHGAP22 | protein_coding | 0.64 | 5.87E-06 |
| AGAP2-AS1 | BPIFB2 | protein_coding | -0.62 | 1.59E-05 |
| AGAP2-AS1 | SERPINA11 | protein_coding | -0.56 | 0.000122288 |
| AGAP2-AS1 | PRSS51 | protein_coding | -0.52 | 0.000455878 |
| AGAP2-AS1 | CNBP | protein_coding | -0.62 | 1.83E-05 |
| AGAP2-AS1 | NTN1 | protein_coding | -0.63 | 1.25E-05 |
| AGAP2-AS1 | LOXL1 | protein_coding | 0.56 | 0.000141481 |
| AGAP2-AS1 | XG | protein_coding | -0.67 | 1.68E-06 |
| AGAP2-AS1 | SLC51A | protein_coding | -0.64 | 6.69E-06 |
| AGAP2-AS1 | MAP3K10 | protein_coding | 0.53 | 0.000421907 |
| AGAP2-AS1 | ISL2 | protein_coding | 0.55 | 0.000208378 |
| AGAP2-AS1 | MAT1A | protein_coding | -0.59 | 5.86E-05 |
| AGAP2-AS1 | FAM184A | protein_coding | -0.56 | 0.000134606 |
| AGAP2-AS1 | TONSL | protein_coding | 0.62 | 1.56E-05 |
| AGAP2-AS1 | ZNF142 | protein_coding | 0.57 | 8.83E-05 |
| AGAP2-AS1 | PHF8 | protein_coding | -0.53 | 0.000412285 |
| AGAP2-AS1 | PLPBP | protein_coding | -0.62 | 1.83E-05 |
| AGAP2-AS1 | VIM | protein_coding | 0.53 | 0.00040738 |
| AGAP2-AS1 | CDH23 | protein_coding | -0.74 | 4.04E-08 |
| AGAP2-AS1 | BSPRY | protein_coding | 0.51 | 0.000617593 |
| AGAP2-AS1 | CCDC157 | protein_coding | 0.52 | 0.000450791 |
| AGAP2-AS1 | MAPK12 | protein_coding | 0.67 | 1.97E-06 |
| AGAP2-AS1 | SHKBP1 | protein_coding | 0.65 | 5.23E-06 |
| AGAP2-AS1 | SLC26A2 | protein_coding | 0.50 | 0.000826603 |
| AGAP2-AS1 | CAMK2B | protein_coding | -0.51 | 0.000756586 |
| AGAP2-AS1 | PLEKHN1 | protein_coding | 0.68 | 1.09E-06 |
| AGAP2-AS1 | RABEP2 | protein_coding | 0.51 | 0.000714698 |
| AGAP2-AS1 | SFR1 | protein_coding | 0.52 | 0.00047357 |
| AGAP2-AS1 | PID1 | protein_coding | -0.61 | 2.65E-05 |
| AGAP2-AS1 | CYP7A1 | protein_coding | -0.65 | 5.39E-06 |
| AGAP2-AS1 | AKR1B1 | protein_coding | 0.74 | 4.11E-08 |
| AGAP2-AS1 | ACSM5 | protein_coding | -0.65 | 4.24E-06 |
| AGAP2-AS1 | PRR32 | protein_coding | -0.58 | 6.36E-05 |
| AGAP2-AS1 | MTCL1 | protein_coding | 0.56 | 0.000159599 |
| AGAP2-AS1 | CETN2 | protein_coding | 0.56 | 0.000144571 |
| AGAP2-AS1 | LGI4 | protein_coding | -0.56 | 0.000153207 |
| AGAP2-AS1 | HOXB9 | protein_coding | 0.53 | 0.000422276 |
| AGAP2-AS1 | UBALD1 | protein_coding | -0.51 | 0.000740627 |
| AGAP2-AS1 | ERLIN1 | protein_coding | -0.57 | 0.000107214 |
| AGAP2-AS1 | DHRS3 | protein_coding | -0.52 | 0.000479029 |
| AGAP2-AS1 | NUP107 | protein_coding | 0.54 | 0.000269051 |
| AGAP2-AS1 | TMOD1 | protein_coding | -0.55 | 0.000198795 |
| AGAP2-AS1 | SLC12A9 | protein_coding | 0.55 | 0.000195837 |
| AGAP2-AS1 | ADIPOR2 | protein_coding | -0.53 | 0.000365004 |
| AGAP2-AS1 | MCRS1 | protein_coding | 0.62 | 1.37E-05 |
| AGAP2-AS1 | ZFAND5 | protein_coding | -0.63 | 1.04E-05 |
| AGAP2-AS1 | TSPAN3 | protein_coding | 0.51 | 0.000750325 |
| AGAP2-AS1 | LRRC37B | protein_coding | 0.62 | 1.40E-05 |
| AGAP2-AS1 | HERC5 | protein_coding | -0.52 | 0.00051184 |
| AGAP2-AS1 | STARD3 | protein_coding | 0.57 | 9.55E-05 |
| AGAP2-AS1 | C8A | protein_coding | -0.52 | 0.000512919 |
| AGAP2-AS1 | PDE9A | protein_coding | 0.57 | 0.000117394 |
| AGAP2-AS1 | MAPK3 | protein_coding | 0.58 | 7.24E-05 |
| AGAP2-AS1 | ACD | protein_coding | 0.67 | 1.53E-06 |
| AGAP2-AS1 | S100A3 | protein_coding | 0.67 | 1.82E-06 |
| AGAP2-AS1 | MDN1 | protein_coding | -0.71 | 1.71E-07 |
| AGAP2-AS1 | GLT1D1 | protein_coding | -0.68 | 9.34E-07 |
| AGAP2-AS1 | LOXL4 | protein_coding | 0.51 | 0.000754779 |
| AGAP2-AS1 | ALDH3A2 | protein_coding | -0.63 | 1.20E-05 |
| AGAP2-AS1 | VIPR1 | protein_coding | -0.68 | 9.23E-07 |
| AGAP2-AS1 | CCT6A | protein_coding | 0.53 | 0.000420831 |
| AGAP2-AS1 | RAB19 | protein_coding | 0.55 | 0.000212515 |
| AGAP2-AS1 | GALNT12 | protein_coding | 0.52 | 0.000519784 |
| AGAP2-AS1 | PSKH1 | protein_coding | 0.55 | 0.000170662 |
| AGAP2-AS1 | MAPK13 | protein_coding | 0.52 | 0.000504314 |
| AGAP2-AS1 | DUOX2 | protein_coding | 0.53 | 0.000414621 |
| AGAP2-AS1 | KANSL2 | protein_coding | 0.58 | 7.85E-05 |
| AGAP2-AS1 | MSMO1 | protein_coding | -0.63 | 1.09E-05 |
| AGAP2-AS1 | C15orf65 | protein_coding | 0.59 | 4.24E-05 |
| AGAP2-AS1 | TDRD10 | protein_coding | -0.59 | 5.68E-05 |
| AGAP2-AS1 | C3orf85 | protein_coding | -0.66 | 2.72E-06 |
| AGAP2-AS1 | ATP13A1 | protein_coding | 0.59 | 5.83E-05 |
| AGAP2-AS1 | AACS | protein_coding | 0.71 | 1.82E-07 |
| AGAP2-AS1 | MT1E | protein_coding | -0.59 | 5.24E-05 |
| AGAP2-AS1 | MMP11 | protein_coding | 0.71 | 1.58E-07 |
| AGAP2-AS1 | DPP3 | protein_coding | 0.62 | 1.50E-05 |
| AGAP2-AS1 | EXTL1 | protein_coding | 0.52 | 0.000533669 |
| AGAP2-AS1 | RAD51D | protein_coding | 0.66 | 3.03E-06 |
| AGAP2-AS1 | MND1 | protein_coding | 0.66 | 2.96E-06 |
| AGAP2-AS1 | TMEM237 | protein_coding | 0.63 | 1.22E-05 |
| AGAP2-AS1 | ANLN | protein_coding | 0.71 | 2.55E-07 |
| AGAP2-AS1 | TUBA1B | protein_coding | 0.59 | 5.31E-05 |
| AGAP2-AS1 | MTMR10 | protein_coding | -0.66 | 3.22E-06 |
| AGAP2-AS1 | GPN1 | protein_coding | 0.66 | 3.01E-06 |
| AGAP2-AS1 | SERINC5 | protein_coding | -0.55 | 0.000221941 |
| AGAP2-AS1 | KLC4 | protein_coding | -0.55 | 0.000184132 |
| AGAP2-AS1 | CTDNEP1 | protein_coding | 0.65 | 3.68E-06 |
| AGAP2-AS1 | TPD52L2 | protein_coding | 0.71 | 1.71E-07 |
| AGAP2-AS1 | BZW2 | protein_coding | 0.51 | 0.000734745 |
| AGAP2-AS1 | CXCL12 | protein_coding | -0.57 | 0.00011745 |
| AGAP2-AS1 | MISP | protein_coding | 0.53 | 0.000324064 |
| AGAP2-AS1 | TRIM45 | protein_coding | 0.62 | 1.58E-05 |
| AGAP2-AS1 | KIF15 | protein_coding | 0.68 | 1.22E-06 |
| AGAP2-AS1 | FGFR1 | protein_coding | 0.50 | 0.000857358 |
| AGAP2-AS1 | MRTFA | protein_coding | 0.62 | 1.37E-05 |
| AGAP2-AS1 | CDC6 | protein_coding | 0.71 | 2.44E-07 |
| AGAP2-AS1 | LPAR2 | protein_coding | 0.67 | 1.49E-06 |
| AGAP2-AS1 | PLPP3 | protein_coding | -0.55 | 0.000210308 |
| AGAP2-AS1 | PLGLB2 | protein_coding | -0.60 | 3.41E-05 |
| AGAP2-AS1 | PDE12 | protein_coding | -0.60 | 2.87E-05 |
| AGAP2-AS1 | ECM2 | protein_coding | -0.66 | 2.55E-06 |
| AGAP2-AS1 | ABCC11 | protein_coding | -0.51 | 0.000602801 |
| AGAP2-AS1 | ITIH3 | protein_coding | -0.62 | 1.81E-05 |
| AGAP2-AS1 | RNF24 | protein_coding | 0.62 | 1.51E-05 |
| AGAP2-AS1 | MFSD12 | protein_coding | 0.51 | 0.000661054 |
| AGAP2-AS1 | FNDC5 | protein_coding | -0.69 | 5.44E-07 |
| AGAP2-AS1 | STIP1 | protein_coding | 0.61 | 2.05E-05 |
| AGAP2-AS1 | GDI1 | protein_coding | 0.61 | 2.38E-05 |
| AGAP2-AS1 | PCK2 | protein_coding | -0.65 | 4.60E-06 |
| AGAP2-AS1 | ADGRE5 | protein_coding | 0.52 | 0.000439263 |
| AGAP2-AS1 | OSBP2 | protein_coding | 0.65 | 3.58E-06 |
| AGAP2-AS1 | AS3MT | protein_coding | -0.51 | 0.000636319 |
| AGAP2-AS1 | SLC25A52 | protein_coding | -0.67 | 1.58E-06 |
| AGAP2-AS1 | GPR35 | protein_coding | 0.56 | 0.000158229 |
| AGAP2-AS1 | OVCH1 | protein_coding | -0.57 | 9.19E-05 |
| AGAP2-AS1 | ESPL1 | protein_coding | 0.54 | 0.000317666 |
| AGAP2-AS1 | NIT2 | protein_coding | -0.51 | 0.000748873 |
| AGAP2-AS1 | UBE2T | protein_coding | 0.69 | 5.12E-07 |
| AGAP2-AS1 | NUP37 | protein_coding | 0.63 | 8.88E-06 |
| AGAP2-AS1 | TAS2R60 | protein_coding | -0.58 | 6.31E-05 |
| AGAP2-AS1 | CCNB2 | protein_coding | 0.73 | 6.04E-08 |
| AGAP2-AS1 | UBE2Z | protein_coding | 0.74 | 4.50E-08 |
| AGAP2-AS1 | SLC16A10 | protein_coding | -0.61 | 2.24E-05 |
| AGAP2-AS1 | UNC45A | protein_coding | 0.67 | 1.96E-06 |
| AGAP2-AS1 | CDK16 | protein_coding | 0.68 | 1.13E-06 |
| AGAP2-AS1 | ASB4 | protein_coding | -0.54 | 0.000269165 |
| AGAP2-AS1 | SMARCAL1 | protein_coding | 0.65 | 4.93E-06 |
| AGAP2-AS1 | ARHGAP35 | protein_coding | -0.56 | 0.000132275 |
| AGAP2-AS1 | CRYAA | protein_coding | -0.62 | 1.44E-05 |
| AGAP2-AS1 | BIK | protein_coding | 0.52 | 0.000512876 |
| AGAP2-AS1 | TTF1 | protein_coding | 0.62 | 1.47E-05 |
| AGAP2-AS1 | UGT1A4 | protein_coding | -0.61 | 2.70E-05 |
| AGAP2-AS1 | CALB2 | protein_coding | 0.53 | 0.000321709 |
| AGAP2-AS1 | C3orf52 | protein_coding | 0.60 | 3.29E-05 |
| AGAP2-AS1 | NUP205 | protein_coding | 0.50 | 0.000849265 |
| AGAP2-AS1 | SAA4 | protein_coding | -0.50 | 0.000848733 |
| AGAP2-AS1 | ANKRD11 | protein_coding | 0.59 | 5.96E-05 |
| AGAP2-AS1 | PALM2AKAP2 | protein_coding | -0.51 | 0.000704539 |
| AGAP2-AS1 | CHRM2 | protein_coding | -0.59 | 5.24E-05 |
| AGAP2-AS1 | PLAAT2 | protein_coding | -0.65 | 3.78E-06 |
| AGAP2-AS1 | ELMO3 | protein_coding | 0.61 | 1.96E-05 |
| AGAP2-AS1 | PDE5A | protein_coding | 0.53 | 0.000399868 |
| AGAP2-AS1 | PPP1R37 | protein_coding | 0.59 | 5.45E-05 |
| AGAP2-AS1 | ACAD8 | protein_coding | -0.52 | 0.000499395 |
| AGAP2-AS1 | ABHD10 | protein_coding | -0.51 | 0.000711118 |
| AGAP2-AS1 | SPSB2 | protein_coding | 0.54 | 0.000304273 |
| AGAP2-AS1 | CFHR5 | protein_coding | -0.57 | 9.52E-05 |
| AGAP2-AS1 | ARHGAP27 | protein_coding | 0.55 | 0.000177785 |
| AGAP2-AS1 | SPRYD4 | protein_coding | -0.64 | 5.88E-06 |
| AGAP2-AS1 | SKI | protein_coding | -0.53 | 0.000347624 |
| AGAP2-AS1 | ACSL5 | protein_coding | -0.57 | 9.86E-05 |
| AGAP2-AS1 | PPP1CC | protein_coding | 0.56 | 0.000168027 |
| AGAP2-AS1 | C16orf74 | protein_coding | 0.51 | 0.000609164 |
| AGAP2-AS1 | ARID4A | protein_coding | -0.52 | 0.000520291 |
| AGAP2-AS1 | SLC25A30 | protein_coding | -0.50 | 0.000818463 |
| AGAP2-AS1 | ERG28 | protein_coding | -0.54 | 0.000270267 |
| AGAP2-AS1 | C4BPA | protein_coding | -0.59 | 5.60E-05 |
| AGAP2-AS1 | RASGRF2 | protein_coding | 0.53 | 0.000362922 |
| AGAP2-AS1 | KIF23 | protein_coding | 0.74 | 3.25E-08 |
| AGAP2-AS1 | GAREM2 | protein_coding | 0.55 | 0.000209065 |
| AGAP2-AS1 | GOLGA6A | protein_coding | -0.63 | 8.79E-06 |
| AGAP2-AS1 | OVOL2 | protein_coding | 0.61 | 2.24E-05 |
| AGAP2-AS1 | IL6ST | protein_coding | -0.51 | 0.000640367 |
| AGAP2-AS1 | POLA1 | protein_coding | 0.61 | 2.25E-05 |
| AGAP2-AS1 | CNOT6 | protein_coding | 0.57 | 9.44E-05 |
| AGAP2-AS1 | TIPIN | protein_coding | 0.52 | 0.000522095 |
| AGAP2-AS1 | NCDN | protein_coding | 0.56 | 0.000145234 |
| AGAP2-AS1 | CLSPN | protein_coding | 0.57 | 0.000111686 |
| AGAP2-AS1 | GLUD2 | protein_coding | -0.52 | 0.00057566 |
| AGAP2-AS1 | GLYATL3 | protein_coding | -0.67 | 1.95E-06 |
| AGAP2-AS1 | HNRNPUL1 | protein_coding | 0.52 | 0.000450201 |
| AGAP2-AS1 | TRPM8 | protein_coding | -0.60 | 4.10E-05 |
| AGAP2-AS1 | HSD17B14 | protein_coding | -0.52 | 0.000545163 |
| AGAP2-AS1 | ARMC6 | protein_coding | -0.50 | 0.000820489 |
| AGAP2-AS1 | CCNB1 | protein_coding | 0.70 | 2.98E-07 |
| AGAP2-AS1 | FOXO6 | protein_coding | 0.59 | 4.98E-05 |
| AGAP2-AS1 | DYNLRB1 | protein_coding | 0.52 | 0.000504178 |
| AGAP2-AS1 | MRPL39 | protein_coding | -0.52 | 0.000441907 |
| AGAP2-AS1 | RFPL1 | protein_coding | -0.57 | 0.000114673 |
| AGAP2-AS1 | SEPTIN2 | protein_coding | 0.62 | 1.78E-05 |
| AGAP2-AS1 | SAPCD2 | protein_coding | 0.65 | 3.78E-06 |
| AGAP2-AS1 | FAM155B | protein_coding | 0.60 | 3.60E-05 |
| AGAP2-AS1 | TCOF1 | protein_coding | 0.69 | 5.48E-07 |
| AGAP2-AS1 | PAQR5 | protein_coding | 0.51 | 0.000657129 |
| AGAP2-AS1 | RSPH14 | protein_coding | 0.58 | 8.27E-05 |
| AGAP2-AS1 | COL5A1 | protein_coding | 0.52 | 0.000575374 |
| AGAP2-AS1 | DCLRE1B | protein_coding | 0.54 | 0.000310332 |
| AGAP2-AS1 | ECT2 | protein_coding | 0.65 | 4.61E-06 |
| AGAP2-AS1 | GBP7 | protein_coding | -0.62 | 1.38E-05 |
| AGAP2-AS1 | USH1C | protein_coding | 0.62 | 1.34E-05 |
| AGAP2-AS1 | SNRK | protein_coding | -0.59 | 5.19E-05 |
| AGAP2-AS1 | PTPN20 | protein_coding | -0.50 | 0.000822374 |
| AGAP2-AS1 | CPOX | protein_coding | -0.51 | 0.000602498 |
| AGAP2-AS1 | DBF4B | protein_coding | 0.67 | 2.00E-06 |
| AGAP2-AS1 | ZNF273 | protein_coding | 0.56 | 0.0001275 |
| AGAP2-AS1 | FAM160B1 | protein_coding | -0.66 | 3.06E-06 |
| AGAP2-AS1 | CROCC | protein_coding | 0.56 | 0.000155451 |
| AGAP2-AS1 | HNRNPUL2 | protein_coding | 0.69 | 5.84E-07 |
| AGAP2-AS1 | PCNX3 | protein_coding | 0.67 | 1.48E-06 |
| AGAP2-AS1 | LRG1 | protein_coding | -0.52 | 0.000453406 |
| AGAP2-AS1 | ZDHHC7 | protein_coding | 0.67 | 1.83E-06 |
| AGAP2-AS1 | RASGEF1B | protein_coding | -0.68 | 8.69E-07 |
| AGAP2-AS1 | NCAPD2 | protein_coding | 0.74 | 2.49E-08 |
| AGAP2-AS1 | KANK1 | protein_coding | -0.62 | 1.63E-05 |
| AGAP2-AS1 | HDAC7 | protein_coding | 0.67 | 1.85E-06 |
| AGAP2-AS1 | MYO19 | protein_coding | 0.61 | 2.30E-05 |
| AGAP2-AS1 | NRG4 | protein_coding | -0.61 | 2.10E-05 |
| AGAP2-AS1 | NKPD1 | protein_coding | 0.56 | 0.000160743 |
| AGAP2-AS1 | RANBP3L | protein_coding | -0.69 | 6.79E-07 |
| AGAP2-AS1 | SFXN5 | protein_coding | -0.54 | 0.000293069 |
| AGAP2-AS1 | UBB | protein_coding | -0.53 | 0.00040507 |
| AGAP2-AS1 | FAM24B | protein_coding | 0.59 | 4.86E-05 |
| AGAP2-AS1 | C18orf54 | protein_coding | 0.71 | 1.68E-07 |
| AGAP2-AS1 | ARL6IP1 | protein_coding | -0.52 | 0.000546675 |
| AGAP2-AS1 | AP4M1 | protein_coding | 0.60 | 3.55E-05 |
| AGAP2-AS1 | MRPS25 | protein_coding | -0.51 | 0.000764634 |
| AGAP2-AS1 | ZG16 | protein_coding | -0.64 | 5.93E-06 |
| AGAP2-AS1 | LRRC75A | protein_coding | 0.51 | 0.000736671 |
| AGAP2-AS1 | IRX5 | protein_coding | 0.53 | 0.0003533 |
| AGAP2-AS1 | CAPN2 | protein_coding | 0.60 | 3.55E-05 |
| AGAP2-AS1 | CRHBP | protein_coding | -0.58 | 6.25E-05 |
| AGAP2-AS1 | CHST14 | protein_coding | 0.70 | 3.88E-07 |
| AGAP2-AS1 | SH3BP2 | protein_coding | -0.58 | 8.49E-05 |
| AGAP2-AS1 | DNM1 | protein_coding | 0.61 | 2.66E-05 |
| AGAP2-AS1 | CRYBG2 | protein_coding | 0.53 | 0.000365317 |
| AGAP2-AS1 | PRR12 | protein_coding | 0.51 | 0.00063618 |
| AGAP2-AS1 | FTL | protein_coding | -0.51 | 0.000610062 |
| AGAP2-AS1 | LYPD1 | protein_coding | 0.68 | 1.06E-06 |
| AGAP2-AS1 | TMEM164 | protein_coding | 0.61 | 2.49E-05 |
| AGAP2-AS1 | SEL1L | protein_coding | -0.51 | 0.000624779 |
| AGAP2-AS1 | EXOC6B | protein_coding | 0.63 | 9.01E-06 |
| AGAP2-AS1 | MLLT11 | protein_coding | 0.52 | 0.000552377 |
| AGAP2-AS1 | GPR180 | protein_coding | -0.54 | 0.000284023 |
| AGAP2-AS1 | WDR47 | protein_coding | 0.51 | 0.000680126 |
| AGAP2-AS1 | GTF3C1 | protein_coding | 0.58 | 7.29E-05 |
| AGAP2-AS1 | NIT1 | protein_coding | -0.58 | 7.81E-05 |
| AGAP2-AS1 | PHYHD1 | protein_coding | -0.60 | 3.93E-05 |
| AGAP2-AS1 | KIAA2012 | protein_coding | -0.54 | 0.000277525 |
| AGAP2-AS1 | EEF1E1 | protein_coding | 0.51 | 0.000579465 |
| AGAP2-AS1 | MPC1 | protein_coding | -0.62 | 1.72E-05 |
| AGAP2-AS1 | ASPDH | protein_coding | -0.64 | 6.06E-06 |
| AGAP2-AS1 | CCDC189 | protein_coding | 0.61 | 2.33E-05 |
| AGAP2-AS1 | CYP2E1 | protein_coding | -0.61 | 2.80E-05 |
| AGAP2-AS1 | CERS1 | protein_coding | 0.54 | 0.00031556 |
| AGAP2-AS1 | GPRIN2 | protein_coding | 0.50 | 0.000828585 |
| AGAP2-AS1 | POLQ | protein_coding | 0.65 | 4.56E-06 |
| AGAP2-AS1 | BPI | protein_coding | -0.50 | 0.000844211 |
| AGAP2-AS1 | NPW | protein_coding | -0.55 | 0.000187413 |
| AGAP2-AS1 | SDC2 | protein_coding | -0.56 | 0.000133427 |
| AGAP2-AS1 | NUTF2 | protein_coding | 0.57 | 9.49E-05 |
| AGAP2-AS1 | GABBR1 | protein_coding | 0.57 | 0.000102631 |
| AGAP2-AS1 | HCN3 | protein_coding | -0.67 | 1.85E-06 |
| AGAP2-AS1 | PER2 | protein_coding | -0.51 | 0.000652643 |
| AGAP2-AS1 | RGPD8 | protein_coding | -0.57 | 9.00E-05 |
| AGAP2-AS1 | MUC12 | protein_coding | 0.53 | 0.00039695 |
| AGAP2-AS1 | PPM1G | protein_coding | 0.71 | 1.63E-07 |
| AGAP2-AS1 | QDPR | protein_coding | -0.64 | 8.35E-06 |
| AGAP2-AS1 | NAT1 | protein_coding | -0.68 | 9.37E-07 |
| AGAP2-AS1 | RGS17 | protein_coding | 0.62 | 1.56E-05 |
| AGAP2-AS1 | SCARA5 | protein_coding | -0.54 | 0.000266705 |
| AGAP2-AS1 | SCUBE1 | protein_coding | -0.57 | 9.02E-05 |
| AGAP2-AS1 | ATP13A2 | protein_coding | 0.70 | 2.84E-07 |
| AGAP2-AS1 | COBLL1 | protein_coding | -0.64 | 6.55E-06 |
| AGAP2-AS1 | LRRC46 | protein_coding | 0.57 | 0.000119082 |
| AGAP2-AS1 | APOF | protein_coding | -0.59 | 4.76E-05 |
| AGAP2-AS1 | PDE4A | protein_coding | 0.65 | 5.31E-06 |
| AGAP2-AS1 | SHLD2 | protein_coding | -0.54 | 0.000252002 |
| AGAP2-AS1 | DTX3 | protein_coding | 0.58 | 6.62E-05 |
| AGAP2-AS1 | KIAA1211L | protein_coding | 0.51 | 0.000591173 |
| AGAP2-AS1 | IFT80 | protein_coding | 0.58 | 6.08E-05 |
| AGAP2-AS1 | XRCC4 | protein_coding | 0.69 | 5.11E-07 |
| AGAP2-AS1 | NIPAL3 | protein_coding | 0.57 | 0.000103365 |
| AGAP2-AS1 | NUDT1 | protein_coding | 0.73 | 4.90E-08 |
| AGAP2-AS1 | BLVRA | protein_coding | 0.71 | 2.12E-07 |
| AGAP2-AS1 | HMCN2 | protein_coding | -0.58 | 6.97E-05 |
| AGAP2-AS1 | UPF3B | protein_coding | 0.64 | 6.04E-06 |
| AGAP2-AS1 | ANKRD22 | protein_coding | 0.53 | 0.00032891 |
| AGAP2-AS1 | FTCDNL1 | protein_coding | -0.58 | 6.31E-05 |
| AGAP2-AS1 | ACKR2 | protein_coding | -0.64 | 5.50E-06 |
| AGAP2-AS1 | SPX | protein_coding | -0.63 | 1.23E-05 |
| AGAP2-AS1 | PGM2L1 | protein_coding | 0.54 | 0.000247088 |
| AGAP2-AS1 | CDC42BPG | protein_coding | 0.64 | 8.20E-06 |
| AGAP2-AS1 | ARL5B | protein_coding | -0.51 | 0.000672069 |
| AGAP2-AS1 | NPM1 | protein_coding | 0.54 | 0.000275812 |
| AGAP2-AS1 | DTNBP1 | protein_coding | 0.53 | 0.00040993 |
| AGAP2-AS1 | NMD3 | protein_coding | -0.56 | 0.000151523 |
| AGAP2-AS1 | CACNG4 | protein_coding | 0.51 | 0.000595323 |
| AGAP2-AS1 | C1S | protein_coding | -0.57 | 9.43E-05 |
| AGAP2-AS1 | VWCE | protein_coding | -0.60 | 3.26E-05 |
| AGAP2-AS1 | NKAIN1 | protein_coding | 0.51 | 0.000649649 |
| AGAP2-AS1 | GALT | protein_coding | -0.60 | 2.87E-05 |
| AGAP2-AS1 | LARP1B | protein_coding | -0.59 | 5.20E-05 |
| AGAP2-AS1 | SRRM3 | protein_coding | 0.68 | 1.05E-06 |
| AGAP2-AS1 | AC006254.1 | protein_coding | -0.66 | 3.22E-06 |
| AGAP2-AS1 | TESMIN | protein_coding | 0.69 | 5.29E-07 |
| AGAP2-AS1 | CATSPER1 | protein_coding | 0.59 | 4.94E-05 |
| AGAP2-AS1 | FAM8A1 | protein_coding | -0.64 | 6.66E-06 |
| AGAP2-AS1 | ST7 | protein_coding | -0.59 | 4.72E-05 |
| AGAP2-AS1 | ACAA1 | protein_coding | -0.69 | 6.76E-07 |
| AGAP2-AS1 | ACAD11 | protein_coding | -0.68 | 1.14E-06 |
| AGAP2-AS1 | FAM122A | protein_coding | -0.60 | 3.26E-05 |
| AGAP2-AS1 | SHMT1 | protein_coding | -0.68 | 1.15E-06 |
| AGAP2-AS1 | ARHGAP39 | protein_coding | 0.50 | 0.000820458 |
| AGAP2-AS1 | TGFA | protein_coding | 0.54 | 0.000280718 |
| AGAP2-AS1 | FZD2 | protein_coding | 0.57 | 0.000100639 |
| AGAP2-AS1 | DENND6B | protein_coding | 0.58 | 7.13E-05 |
| AGAP2-AS1 | NFIA | protein_coding | -0.51 | 0.000679663 |
| AGAP2-AS1 | CCNO | protein_coding | 0.76 | 1.14E-08 |
| AGAP2-AS1 | KIF18A | protein_coding | 0.69 | 5.88E-07 |
| AGAP2-AS1 | MLYCD | protein_coding | -0.53 | 0.000326513 |
| AGAP2-AS1 | KPNA2 | protein_coding | 0.54 | 0.000279932 |
| AGAP2-AS1 | TMEM52 | protein_coding | -0.51 | 0.000597012 |
| AGAP2-AS1 | SELENOO | protein_coding | -0.54 | 0.000237695 |
| AGAP2-AS1 | PEX11A | protein_coding | -0.63 | 8.40E-06 |
| AGAP2-AS1 | PDRG1 | protein_coding | 0.55 | 0.000203062 |
| AGAP2-AS1 | SH3D21 | protein_coding | 0.55 | 0.000211004 |
| AGAP2-AS1 | MAD2L1 | protein_coding | 0.73 | 5.48E-08 |
| AGAP2-AS1 | CYB561 | protein_coding | 0.55 | 0.000189041 |
| AGAP2-AS1 | NRM | protein_coding | 0.60 | 3.40E-05 |
| AGAP2-AS1 | NRSN2 | protein_coding | 0.63 | 8.42E-06 |
| AGAP2-AS1 | AUTS2 | protein_coding | -0.55 | 0.00022163 |
| AGAP2-AS1 | FBXO7 | protein_coding | -0.52 | 0.000536183 |
| AGAP2-AS1 | BCKDHB | protein_coding | -0.62 | 1.62E-05 |
| AGAP2-AS1 | COL18A1 | protein_coding | -0.58 | 6.53E-05 |
| AGAP2-AS1 | KCNJ8 | protein_coding | -0.63 | 8.55E-06 |
| AGAP2-AS1 | LMTK3 | protein_coding | 0.61 | 2.23E-05 |
| AGAP2-AS1 | NT5DC2 | protein_coding | 0.63 | 1.13E-05 |
| AGAP2-AS1 | DMWD | protein_coding | 0.63 | 1.16E-05 |
| AGAP2-AS1 | GRIN1 | protein_coding | 0.61 | 2.72E-05 |
| AGAP2-AS1 | TRMT10A | protein_coding | -0.51 | 0.000579097 |
| AGAP2-AS1 | FAM122B | protein_coding | 0.59 | 5.06E-05 |
| AGAP2-AS1 | PRPSAP1 | protein_coding | -0.53 | 0.000376397 |
| AGAP2-AS1 | CCL23 | protein_coding | -0.58 | 7.46E-05 |
| AGAP2-AS1 | PI15 | protein_coding | 0.56 | 0.000164279 |
| AGAP2-AS1 | CST2 | protein_coding | 0.56 | 0.000133213 |
| AGAP2-AS1 | SLITRK3 | protein_coding | -0.51 | 0.000633875 |
| AGAP2-AS1 | AC021066.1 | protein_coding | 0.59 | 5.73E-05 |
| AGAP2-AS1 | MKRN2OS | protein_coding | 0.64 | 6.78E-06 |
| AGAP2-AS1 | GGN | protein_coding | 0.64 | 5.72E-06 |
| AGAP2-AS1 | DYNLL1 | protein_coding | 0.56 | 0.000130069 |
| AGAP2-AS1 | SLC4A3 | protein_coding | 0.63 | 1.25E-05 |
| AGAP2-AS1 | SIPA1L3 | protein_coding | 0.63 | 8.60E-06 |
| AGAP2-AS1 | CERS5 | protein_coding | 0.73 | 5.01E-08 |
| AGAP2-AS1 | COQ8A | protein_coding | -0.53 | 0.000431217 |
| AGAP2-AS1 | NCAPG2 | protein_coding | 0.56 | 0.000150648 |
| AGAP2-AS1 | ARMC9 | protein_coding | 0.67 | 2.09E-06 |
| AGAP2-AS1 | RBBP7 | protein_coding | 0.54 | 0.00024366 |
| AGAP2-AS1 | TMEM165 | protein_coding | 0.62 | 1.63E-05 |
| AGAP2-AS1 | EPS15L1 | protein_coding | 0.56 | 0.00015229 |
| AGAP2-AS1 | TYMS | protein_coding | 0.61 | 2.60E-05 |
| AGAP2-AS1 | ADGRG1 | protein_coding | 0.64 | 6.34E-06 |
| AGAP2-AS1 | LEMD2 | protein_coding | 0.58 | 7.20E-05 |
| AGAP2-AS1 | CHMP4B | protein_coding | 0.60 | 3.36E-05 |
| AGAP2-AS1 | CD1D | protein_coding | -0.54 | 0.000290039 |
| AGAP2-AS1 | AGRN | protein_coding | 0.58 | 8.12E-05 |
| AGAP2-AS1 | PRKAR2A | protein_coding | -0.51 | 0.000619985 |
| AGAP2-AS1 | GABRD | protein_coding | 0.60 | 4.08E-05 |
| AGAP2-AS1 | PNRC1 | protein_coding | -0.52 | 0.000553975 |
| AGAP2-AS1 | ANKS6 | protein_coding | 0.64 | 8.14E-06 |
| AGAP2-AS1 | NAP1L1 | protein_coding | 0.62 | 1.84E-05 |
| AGAP2-AS1 | METTL7A | protein_coding | -0.60 | 4.09E-05 |
| AGAP2-AS1 | SART3 | protein_coding | 0.61 | 2.37E-05 |
| AGAP2-AS1 | ACADS | protein_coding | -0.51 | 0.000708742 |
| AGAP2-AS1 | ATIC | protein_coding | 0.60 | 4.06E-05 |
| AGAP2-AS1 | CCNL1 | protein_coding | -0.64 | 5.82E-06 |
| AGAP2-AS1 | CDK5R2 | protein_coding | 0.52 | 0.000440691 |
| AGAP2-AS1 | MAPK11 | protein_coding | 0.58 | 6.67E-05 |
| AGAP2-AS1 | TCP10L | protein_coding | -0.63 | 9.12E-06 |
| AGAP2-AS1 | DHDH | protein_coding | 0.52 | 0.000513065 |
| AGAP2-AS1 | F12 | protein_coding | -0.51 | 0.000629099 |
| AGAP2-AS1 | SLC17A2 | protein_coding | -0.63 | 8.48E-06 |
| AGAP2-AS1 | RTL4 | protein_coding | -0.62 | 1.83E-05 |
| AGAP2-AS1 | ANKRD18B | protein_coding | 0.52 | 0.00047277 |
| AGAP2-AS1 | ARPC1B | protein_coding | 0.64 | 8.06E-06 |
| AGAP2-AS1 | FKBP1A | protein_coding | 0.60 | 2.87E-05 |
| AGAP2-AS1 | CNIH2 | protein_coding | 0.62 | 1.44E-05 |
| AGAP2-AS1 | PFKM | protein_coding | 0.67 | 1.48E-06 |
| AGAP2-AS1 | TBX15 | protein_coding | -0.51 | 0.000710551 |
| AGAP2-AS1 | PYGB | protein_coding | 0.63 | 9.42E-06 |
| AGAP2-AS1 | ABCD3 | protein_coding | -0.55 | 0.000217126 |
| AGAP2-AS1 | DMBX1 | protein_coding | 0.69 | 5.29E-07 |
| AGAP2-AS1 | AOX1 | protein_coding | -0.57 | 0.000107983 |
| AGAP2-AS1 | DDX60L | protein_coding | -0.56 | 0.000145535 |
| AGAP2-AS1 | KIAA0930 | protein_coding | 0.53 | 0.000409923 |
| AGAP2-AS1 | TP53I11 | protein_coding | 0.52 | 0.000472181 |
| AGAP2-AS1 | CYP2A7 | protein_coding | -0.63 | 1.08E-05 |
| AGAP2-AS1 | AHSG | protein_coding | -0.53 | 0.000333312 |
| AGAP2-AS1 | SSBP4 | protein_coding | 0.53 | 0.000337294 |
| AGAP2-AS1 | DALRD3 | protein_coding | 0.54 | 0.000295976 |
| AGAP2-AS1 | RMND5A | protein_coding | -0.59 | 5.32E-05 |
| AGAP2-AS1 | NDST3 | protein_coding | -0.57 | 0.0001022 |
| AGAP2-AS1 | PIP4K2B | protein_coding | 0.72 | 1.00E-07 |
| AGAP2-AS1 | TMEM54 | protein_coding | 0.59 | 5.42E-05 |
| AGAP2-AS1 | SELENON | protein_coding | 0.58 | 6.34E-05 |
| AGAP2-AS1 | WDYHV1 | protein_coding | 0.53 | 0.000372215 |
| AGAP2-AS1 | EFHC1 | protein_coding | 0.53 | 0.000357986 |
| AGAP2-AS1 | SMARCA4 | protein_coding | 0.63 | 8.60E-06 |
| AGAP2-AS1 | CYP27A1 | protein_coding | -0.66 | 3.18E-06 |
| AGAP2-AS1 | GAS8 | protein_coding | 0.59 | 5.38E-05 |
| AGAP2-AS1 | ZFP1 | protein_coding | -0.56 | 0.000164644 |
| AGAP2-AS1 | CXorf66 | protein_coding | -0.66 | 2.73E-06 |
| AGAP2-AS1 | SLC44A2 | protein_coding | 0.54 | 0.000314863 |
| AGAP2-AS1 | CA11 | protein_coding | 0.50 | 0.00077795 |
| AGAP2-AS1 | SLC6A2 | protein_coding | -0.58 | 6.93E-05 |
| AGAP2-AS1 | P3H4 | protein_coding | 0.77 | 5.82E-09 |
| AGAP2-AS1 | SLC2A9 | protein_coding | -0.58 | 6.69E-05 |
| AGAP2-AS1 | FUT11 | protein_coding | 0.53 | 0.000376841 |
| AGAP2-AS1 | TSSK6 | protein_coding | 0.63 | 1.20E-05 |
| AGAP2-AS1 | SLC25A42 | protein_coding | -0.62 | 1.32E-05 |
| AGAP2-AS1 | MPV17 | protein_coding | 0.74 | 3.79E-08 |
| AGAP2-AS1 | HDAC6 | protein_coding | -0.65 | 3.76E-06 |
| AGAP2-AS1 | MAIP1 | protein_coding | -0.54 | 0.000242885 |
| AGAP2-AS1 | SMLR1 | protein_coding | -0.54 | 0.000256156 |
| AGAP2-AS1 | NEDD1 | protein_coding | 0.52 | 0.000500883 |
| AGAP2-AS1 | FCN3 | protein_coding | -0.52 | 0.000495999 |
| AGAP2-AS1 | REEP4 | protein_coding | 0.71 | 1.91E-07 |
| AGAP2-AS1 | ACACB | protein_coding | -0.57 | 9.64E-05 |
| AGAP2-AS1 | RIDA | protein_coding | -0.64 | 6.57E-06 |
| AGAP2-AS1 | RUNDC1 | protein_coding | 0.69 | 4.87E-07 |
| AGAP2-AS1 | ABCA10 | protein_coding | -0.56 | 0.000138353 |
| AGAP2-AS1 | YWHAZ | protein_coding | 0.52 | 0.000510163 |
| AGAP2-AS1 | EGFL6 | protein_coding | 0.51 | 0.000681691 |
| AGAP2-AS1 | AP1G2 | protein_coding | 0.59 | 5.89E-05 |
| AGAP2-AS1 | STIL | protein_coding | 0.59 | 5.09E-05 |
| AGAP2-AS1 | PRDM12 | protein_coding | 0.53 | 0.000335422 |
| AGAP2-AS1 | GPD2 | protein_coding | 0.54 | 0.000300083 |
| AGAP2-AS1 | KLHL32 | protein_coding | -0.58 | 7.24E-05 |
| AGAP2-AS1 | RMDN3 | protein_coding | -0.56 | 0.000160075 |
| AGAP2-AS1 | CYP4V2 | protein_coding | -0.58 | 6.09E-05 |
| AGAP2-AS1 | PUS10 | protein_coding | -0.68 | 1.23E-06 |
| AGAP2-AS1 | YWHAQ | protein_coding | 0.51 | 0.000640712 |
| AGAP2-AS1 | SLC23A2 | protein_coding | -0.65 | 3.85E-06 |
| AGAP2-AS1 | DDX39A | protein_coding | 0.63 | 1.22E-05 |
| AGAP2-AS1 | DHRS4 | protein_coding | -0.56 | 0.000154586 |
| AGAP2-AS1 | CEP89 | protein_coding | 0.69 | 6.01E-07 |
| AGAP2-AS1 | F13B | protein_coding | -0.53 | 0.000365617 |
| AGAP2-AS1 | TMBIM6 | protein_coding | -0.54 | 0.000272924 |
| AGAP2-AS1 | CETP | protein_coding | -0.60 | 3.90E-05 |
| AGAP2-AS1 | TFCP2 | protein_coding | 0.52 | 0.000534518 |
| AGAP2-AS1 | ILF3 | protein_coding | 0.65 | 3.48E-06 |
| AGAP2-AS1 | ASIC1 | protein_coding | 0.57 | 9.43E-05 |
| AGAP2-AS1 | CYTH3 | protein_coding | 0.53 | 0.000365053 |
| AGAP2-AS1 | PRRX2 | protein_coding | 0.52 | 0.000508728 |
| AGAP2-AS1 | PGS1 | protein_coding | 0.67 | 1.66E-06 |
| AGAP2-AS1 | HNRNPA1P48 | protein_coding | 0.50 | 0.000835556 |
| AGAP2-AS1 | INSIG1 | protein_coding | -0.69 | 6.62E-07 |
| AGAP2-AS1 | GNB1L | protein_coding | 0.61 | 2.67E-05 |
| AGAP2-AS1 | GPT2 | protein_coding | -0.55 | 0.00019103 |
| AGAP2-AS1 | MMP24 | protein_coding | 0.62 | 1.70E-05 |
| AGAP2-AS1 | PITX1 | protein_coding | 0.63 | 1.01E-05 |
| AGAP2-AS1 | CCDC73 | protein_coding | -0.52 | 0.000578354 |
| AGAP2-AS1 | TEDC2 | protein_coding | 0.69 | 6.47E-07 |
| AGAP2-AS1 | SMAD3 | protein_coding | 0.60 | 3.68E-05 |
| AGAP2-AS1 | KIF18B | protein_coding | 0.72 | 1.05E-07 |
| AGAP2-AS1 | ASGR1 | protein_coding | -0.50 | 0.000771246 |
| AGAP2-AS1 | POLR3K | protein_coding | 0.51 | 0.000633807 |
| AGAP2-AS1 | CIDEB | protein_coding | -0.57 | 8.59E-05 |
| AGAP2-AS1 | MKRN1 | protein_coding | 0.52 | 0.000449843 |
| AGAP2-AS1 | AGXT | protein_coding | -0.55 | 0.000209274 |
| AGAP2-AS1 | RAD9A | protein_coding | 0.52 | 0.000525034 |
| AGAP2-AS1 | FIBP | protein_coding | 0.64 | 5.70E-06 |
| AGAP2-AS1 | SLC5A7 | protein_coding | -0.54 | 0.000250672 |
| AGAP2-AS1 | RFXANK | protein_coding | 0.55 | 0.000221428 |
| AGAP2-AS1 | TDRD15 | protein_coding | -0.65 | 3.69E-06 |
| AGAP2-AS1 | FMO3 | protein_coding | -0.63 | 8.58E-06 |
| AGAP2-AS1 | EPHX1 | protein_coding | -0.65 | 4.95E-06 |
| AGAP2-AS1 | DNMT1 | protein_coding | 0.63 | 1.15E-05 |
| AGAP2-AS1 | SLC22A10 | protein_coding | -0.57 | 0.000109616 |
| AGAP2-AS1 | CCDC148 | protein_coding | 0.51 | 0.000632816 |
| AGAP2-AS1 | ITLN2 | protein_coding | -0.54 | 0.000285741 |
| AGAP2-AS1 | LIPC | protein_coding | -0.53 | 0.000347464 |
| AGAP2-AS1 | RUFY1 | protein_coding | 0.53 | 0.000328698 |
| AGAP2-AS1 | CNTLN | protein_coding | -0.59 | 4.35E-05 |
| AGAP2-AS1 | TAF1D | protein_coding | 0.52 | 0.000440939 |
| AGAP2-AS1 | ADRA1A | protein_coding | -0.67 | 1.46E-06 |
| AGAP2-AS1 | FANCG | protein_coding | 0.69 | 7.68E-07 |
| AGAP2-AS1 | LONRF1 | protein_coding | -0.54 | 0.000261248 |
| AGAP2-AS1 | TMEM44 | protein_coding | 0.61 | 2.53E-05 |
| AGAP2-AS1 | ABHD14A-ACY1 | protein_coding | -0.53 | 0.000402228 |
| AGAP2-AS1 | ARSF | protein_coding | -0.65 | 4.16E-06 |
| AGAP2-AS1 | GPSM1 | protein_coding | 0.65 | 3.96E-06 |
| AGAP2-AS1 | SRCAP | protein_coding | 0.59 | 4.57E-05 |
| AGAP2-AS1 | RNF34 | protein_coding | 0.50 | 0.000849584 |
| AGAP2-AS1 | ZCCHC2 | protein_coding | -0.60 | 3.19E-05 |
| AGAP2-AS1 | DMD | protein_coding | -0.60 | 3.08E-05 |
| AGAP2-AS1 | ELOVL1 | protein_coding | 0.59 | 5.18E-05 |
| AGAP2-AS1 | CD160 | protein_coding | -0.57 | 0.00010267 |
| AGAP2-AS1 | NCK2 | protein_coding | 0.73 | 5.29E-08 |
| AGAP2-AS1 | TMEM240 | protein_coding | 0.55 | 0.000188351 |
| AGAP2-AS1 | SLC35D1 | protein_coding | -0.66 | 2.57E-06 |
| AGAP2-AS1 | SLC43A1 | protein_coding | -0.54 | 0.000288843 |
| AGAP2-AS1 | C5orf30 | protein_coding | 0.65 | 3.71E-06 |
| AGAP2-AS1 | AP2B1 | protein_coding | 0.64 | 5.92E-06 |
| AGAP2-AS1 | ABCA9 | protein_coding | -0.55 | 0.000194163 |
| AGAP2-AS1 | NCOR2 | protein_coding | 0.59 | 4.24E-05 |
| AGAP2-AS1 | SPATS2 | protein_coding | 0.72 | 1.35E-07 |
| AGAP2-AS1 | RMDN2 | protein_coding | -0.67 | 1.59E-06 |
| AGAP2-AS1 | SNAP25 | protein_coding | 0.57 | 9.08E-05 |
| AGAP2-AS1 | HIGD1C | protein_coding | -0.58 | 7.16E-05 |
| AGAP2-AS1 | SLC28A2 | protein_coding | -0.53 | 0.000324569 |
| AGAP2-AS1 | RPUSD1 | protein_coding | 0.54 | 0.000248295 |
| AGAP2-AS1 | SMCO4 | protein_coding | -0.56 | 0.000135983 |
| AGAP2-AS1 | S1PR1 | protein_coding | -0.53 | 0.000410363 |
| AGAP2-AS1 | LLGL1 | protein_coding | 0.67 | 1.57E-06 |
| AGAP2-AS1 | SMG9 | protein_coding | 0.71 | 1.83E-07 |
| AGAP2-AS1 | C12orf10 | protein_coding | 0.56 | 0.000151253 |
| AGAP2-AS1 | NLGN1 | protein_coding | 0.58 | 6.20E-05 |
| AGAP2-AS1 | DNASE1 | protein_coding | 0.53 | 0.000405337 |
| AGAP2-AS1 | GRHPR | protein_coding | -0.62 | 1.58E-05 |
| AGAP2-AS1 | SAP30BP | protein_coding | 0.56 | 0.000129138 |
| AGAP2-AS1 | A2M | protein_coding | -0.68 | 8.10E-07 |
| AGAP2-AS1 | RXRA | protein_coding | -0.65 | 4.94E-06 |
| AGAP2-AS1 | LHFPL5 | protein_coding | 0.56 | 0.000138066 |
| AGAP2-AS1 | ANKRD27 | protein_coding | 0.59 | 5.83E-05 |
| AGAP2-AS1 | SERINC1 | protein_coding | -0.51 | 0.000599333 |
| AGAP2-AS1 | ADH6 | protein_coding | -0.55 | 0.000224872 |
| AGAP2-AS1 | DNASE1L2 | protein_coding | 0.57 | 0.000109973 |
| AGAP2-AS1 | MITD1 | protein_coding | 0.53 | 0.000393617 |
| AGAP2-AS1 | FMR1 | protein_coding | -0.60 | 3.67E-05 |
| AGAP2-AS1 | APOH | protein_coding | -0.55 | 0.000202453 |
| AGAP2-AS1 | RGN | protein_coding | -0.65 | 4.82E-06 |
| AGAP2-AS1 | KLHL2 | protein_coding | -0.50 | 0.000827112 |
| AGAP2-AS1 | CSRNP1 | protein_coding | -0.53 | 0.000326352 |
| AGAP2-AS1 | NRBF2 | protein_coding | -0.59 | 4.67E-05 |
| AGAP2-AS1 | GUSB | protein_coding | -0.51 | 0.000751968 |
| AGAP2-AS1 | CASTOR3 | protein_coding | 0.64 | 6.40E-06 |
| AGAP2-AS1 | ASPHD1 | protein_coding | 0.77 | 5.31E-09 |
| AGAP2-AS1 | FKBP9 | protein_coding | 0.62 | 1.32E-05 |
| AGAP2-AS1 | PC | protein_coding | -0.53 | 0.00037125 |
| AGAP2-AS1 | CNST | protein_coding | -0.52 | 0.000470307 |
| AGAP2-AS1 | C1RL | protein_coding | -0.59 | 5.27E-05 |
| AGAP2-AS1 | OLFML2B | protein_coding | 0.57 | 0.000118389 |
| AGAP2-AS1 | MFAP1 | protein_coding | 0.62 | 1.88E-05 |
| AGAP2-AS1 | PTGFRN | protein_coding | 0.52 | 0.000437092 |
| AGAP2-AS1 | HAL | protein_coding | -0.57 | 0.000112657 |
| AGAP2-AS1 | CYP3A43 | protein_coding | -0.70 | 4.41E-07 |
| AGAP2-AS1 | UBXN11 | protein_coding | 0.52 | 0.000551778 |
| AGAP2-AS1 | PGRMC1 | protein_coding | -0.62 | 1.38E-05 |
| AGAP2-AS1 | CD36 | protein_coding | -0.65 | 3.50E-06 |
| AGAP2-AS1 | XRCC1 | protein_coding | 0.61 | 2.74E-05 |
| AGAP2-AS1 | NR2C2AP | protein_coding | 0.67 | 1.63E-06 |
| AGAP2-AS1 | FBXO6 | protein_coding | -0.58 | 8.32E-05 |
| AGAP2-AS1 | CKMT1A | protein_coding | 0.59 | 5.22E-05 |
| AGAP2-AS1 | ZNF213 | protein_coding | 0.60 | 2.89E-05 |
| AGAP2-AS1 | MARCKS | protein_coding | 0.57 | 0.000120603 |
| AGAP2-AS1 | GIT1 | protein_coding | 0.78 | 1.48E-09 |
| AGAP2-AS1 | EPHA10 | protein_coding | 0.57 | 0.000111918 |
| AGAP2-AS1 | GSK3A | protein_coding | 0.58 | 7.59E-05 |
| AGAP2-AS1 | MYBL2 | protein_coding | 0.69 | 6.43E-07 |
| AGAP2-AS1 | STRN4 | protein_coding | 0.60 | 3.25E-05 |
| AGAP2-AS1 | FBXW9 | protein_coding | 0.62 | 1.77E-05 |
| AGAP2-AS1 | DEPDC1 | protein_coding | 0.64 | 6.53E-06 |
| AGAP2-AS1 | PLOD3 | protein_coding | 0.51 | 0.000728223 |
| AGAP2-AS1 | PDSS2 | protein_coding | -0.69 | 6.72E-07 |
| AGAP2-AS1 | DNAJC7 | protein_coding | 0.60 | 3.58E-05 |
| AGAP2-AS1 | CDNF | protein_coding | -0.59 | 5.64E-05 |
| AGAP2-AS1 | ATRAID | protein_coding | 0.67 | 2.00E-06 |
| AGAP2-AS1 | PAX8 | protein_coding | 0.56 | 0.000147211 |
| AGAP2-AS1 | GLUD1 | protein_coding | -0.65 | 4.84E-06 |
| AGAP2-AS1 | LIFR | protein_coding | -0.56 | 0.00014086 |
| AGAP2-AS1 | CDCA2 | protein_coding | 0.69 | 6.60E-07 |
| AGAP2-AS1 | SEC62 | protein_coding | -0.56 | 0.00012149 |
| AGAP2-AS1 | ATP11C | protein_coding | -0.69 | 6.07E-07 |
| AGAP2-AS1 | GRAMD1B | protein_coding | 0.67 | 2.08E-06 |
| AGAP2-AS1 | OSBPL5 | protein_coding | 0.57 | 9.03E-05 |
| AGAP2-AS1 | IQCD | protein_coding | 0.60 | 3.17E-05 |
| AGAP2-AS1 | MBNL3 | protein_coding | -0.52 | 0.000489049 |
| AGAP2-AS1 | PCCA | protein_coding | -0.52 | 0.00057201 |
| AGAP2-AS1 | PPP2R1A | protein_coding | 0.57 | 9.86E-05 |
| AGAP2-AS1 | TEX9 | protein_coding | 0.51 | 0.000632084 |
| AGAP2-AS1 | ZG16B | protein_coding | 0.56 | 0.000149712 |
| AGAP2-AS1 | BLM | protein_coding | 0.67 | 1.78E-06 |
| AGAP2-AS1 | GPHN | protein_coding | -0.58 | 6.91E-05 |
| AGAP2-AS1 | ITIH1 | protein_coding | -0.61 | 2.30E-05 |
| AGAP2-AS1 | DEFB132 | protein_coding | -0.56 | 0.00016197 |
| AGAP2-AS1 | WNK3 | protein_coding | -0.59 | 5.51E-05 |
| AGAP2-AS1 | PLCE1 | protein_coding | 0.56 | 0.000146969 |
| AGAP2-AS1 | TMEM51 | protein_coding | 0.72 | 1.19E-07 |
| AGAP2-AS1 | NSD2 | protein_coding | 0.51 | 0.000742882 |
| AGAP2-AS1 | SMOX | protein_coding | 0.64 | 5.80E-06 |
| AGAP2-AS1 | EZH1 | protein_coding | 0.53 | 0.000393314 |
| AGAP2-AS1 | EIF3D | protein_coding | 0.68 | 1.10E-06 |
| AGAP2-AS1 | XPO6 | protein_coding | 0.54 | 0.000293918 |
| AGAP2-AS1 | LASP1 | protein_coding | 0.50 | 0.000789333 |
| AGAP2-AS1 | C20orf144 | protein_coding | 0.56 | 0.000128377 |
| AGAP2-AS1 | CIPC | protein_coding | -0.57 | 9.02E-05 |
| AGAP2-AS1 | SHFL | protein_coding | -0.64 | 7.05E-06 |
| AGAP2-AS1 | BAX | protein_coding | 0.51 | 0.000720294 |
| AGAP2-AS1 | CLIP2 | protein_coding | 0.75 | 1.39E-08 |
| AGAP2-AS1 | DECR1 | protein_coding | -0.61 | 2.41E-05 |
| AGAP2-AS1 | DPF2 | protein_coding | 0.67 | 1.54E-06 |
| AGAP2-AS1 | CBX1 | protein_coding | 0.73 | 7.28E-08 |
| AGAP2-AS1 | RAPGEF4 | protein_coding | -0.63 | 1.25E-05 |
| AGAP2-AS1 | MYL6B | protein_coding | 0.62 | 1.83E-05 |
| AGAP2-AS1 | VASP | protein_coding | 0.57 | 0.000104508 |
| AGAP2-AS1 | MSRB1 | protein_coding | -0.55 | 0.000181474 |
| AGAP2-AS1 | ABCA3 | protein_coding | 0.61 | 2.65E-05 |
| AGAP2-AS1 | KIAA1841 | protein_coding | 0.76 | 6.90E-09 |
| AGAP2-AS1 | MRE11 | protein_coding | 0.64 | 7.33E-06 |
| AGAP2-AS1 | PRSS22 | protein_coding | 0.53 | 0.000373684 |
| AGAP2-AS1 | MTA2 | protein_coding | 0.58 | 6.56E-05 |
| AGAP2-AS1 | PHTF1 | protein_coding | 0.56 | 0.000146597 |
| AGAP2-AS1 | CST4 | protein_coding | 0.55 | 0.000207249 |
| AGAP2-AS1 | AL845331.2 | protein_coding | -0.61 | 2.68E-05 |
| AGAP2-AS1 | CLHC1 | protein_coding | 0.60 | 4.05E-05 |
| AGAP2-AS1 | SH3GLB2 | protein_coding | 0.52 | 0.000540304 |
| AGAP2-AS1 | ENHO | protein_coding | -0.59 | 4.98E-05 |
| AGAP2-AS1 | RBP5 | protein_coding | -0.73 | 4.80E-08 |
| AGAP2-AS1 | MATN3 | protein_coding | 0.61 | 2.35E-05 |
| AGAP2-AS1 | AKT3 | protein_coding | 0.61 | 2.25E-05 |
| AGAP2-AS1 | PRKD2 | protein_coding | 0.56 | 0.000151746 |
| AGAP2-AS1 | MCCC2 | protein_coding | -0.57 | 0.000107272 |
| AGAP2-AS1 | CKMT1B | protein_coding | 0.53 | 0.000350052 |
| AGAP2-AS1 | PDZD7 | protein_coding | 0.65 | 3.85E-06 |
| AGAP2-AS1 | EVA1A | protein_coding | -0.56 | 0.000165648 |
| AGAP2-AS1 | F9 | protein_coding | -0.56 | 0.000156859 |
| AGAP2-AS1 | SARDH | protein_coding | -0.65 | 3.76E-06 |
| AGAP2-AS1 | CDCA8 | protein_coding | 0.66 | 3.30E-06 |
| AGAP2-AS1 | GJC1 | protein_coding | 0.61 | 2.81E-05 |
| AGAP2-AS1 | DYRK1B | protein_coding | 0.62 | 1.43E-05 |
| AGAP2-AS1 | MFAP3L | protein_coding | -0.71 | 1.92E-07 |
| AGAP2-AS1 | CCDC25 | protein_coding | -0.51 | 0.000625535 |
| AGAP2-AS1 | ACY3 | protein_coding | -0.51 | 0.000605682 |
| AGAP2-AS1 | SIRT7 | protein_coding | 0.54 | 0.000240631 |
| AGAP2-AS1 | GGCX | protein_coding | -0.60 | 3.10E-05 |
| AGAP2-AS1 | RORC | protein_coding | -0.54 | 0.000234129 |
| AGAP2-AS1 | ABHD1 | protein_coding | -0.58 | 7.07E-05 |
| AGAP2-AS1 | ELL2 | protein_coding | -0.56 | 0.00014981 |
| AGAP2-AS1 | MKI67 | protein_coding | 0.71 | 1.62E-07 |
| AGAP2-AS1 | TRPC1 | protein_coding | 0.65 | 4.91E-06 |
| AGAP2-AS1 | BPHL | protein_coding | -0.57 | 0.00010934 |
| AGAP2-AS1 | DHX38 | protein_coding | 0.51 | 0.000698529 |
| AGAP2-AS1 | TEAD2 | protein_coding | 0.56 | 0.000150863 |
| AGAP2-AS1 | TMEM132A | protein_coding | 0.76 | 6.55E-09 |
| AGAP2-AS1 | CPN2 | protein_coding | -0.56 | 0.000123873 |
| AGAP2-AS1 | TNFAIP8L1 | protein_coding | -0.51 | 0.000681906 |
| AGAP2-AS1 | BBS1 | protein_coding | 0.54 | 0.000303567 |
| AGAP2-AS1 | TMEM109 | protein_coding | 0.64 | 7.57E-06 |
| AGAP2-AS1 | IGFBP4 | protein_coding | -0.55 | 0.000215529 |
| AGAP2-AS1 | TIMP1 | protein_coding | 0.54 | 0.000291274 |
| AGAP2-AS1 | CHST12 | protein_coding | 0.50 | 0.000850305 |
| AGAP2-AS1 | HIF1AN | protein_coding | 0.57 | 0.000113743 |
| AGAP2-AS1 | MYD88 | protein_coding | -0.62 | 1.50E-05 |
| AGAP2-AS1 | MVP | protein_coding | 0.68 | 9.88E-07 |
| AGAP2-AS1 | ARNT2 | protein_coding | 0.58 | 7.18E-05 |
| AGAP2-AS1 | P2RX3 | protein_coding | -0.64 | 8.08E-06 |
| AGAP2-AS1 | FBLIM1 | protein_coding | 0.62 | 1.88E-05 |
| AGAP2-AS1 | SERPINB1 | protein_coding | 0.56 | 0.000129364 |
| AGAP2-AS1 | GPIHBP1 | protein_coding | -0.61 | 1.97E-05 |
| AGAP2-AS1 | DNAAF3 | protein_coding | 0.56 | 0.000143868 |
| AGAP2-AS1 | HID1 | protein_coding | 0.56 | 0.000125925 |
| AGAP2-AS1 | PTGR1 | protein_coding | -0.54 | 0.00026758 |
| AGAP2-AS1 | RAF1 | protein_coding | -0.60 | 2.93E-05 |
| AGAP2-AS1 | TAX1BP3 | protein_coding | 0.72 | 1.48E-07 |
| AGAP2-AS1 | ARL4D | protein_coding | -0.51 | 0.000769501 |
| AGAP2-AS1 | SAMD4B | protein_coding | 0.52 | 0.000492166 |
| AGAP2-AS1 | DGAT2 | protein_coding | -0.65 | 4.55E-06 |
| AGAP2-AS1 | IGFALS | protein_coding | -0.59 | 5.19E-05 |
| AGAP2-AS1 | DDA1 | protein_coding | 0.67 | 1.53E-06 |
| AGAP2-AS1 | FAR1 | protein_coding | 0.50 | 0.000851171 |
| AGAP2-AS1 | OTC | protein_coding | -0.60 | 3.65E-05 |
| AGAP2-AS1 | XYLT2 | protein_coding | 0.64 | 6.95E-06 |
| AGAP2-AS1 | HROB | protein_coding | 0.68 | 1.17E-06 |
| AGAP2-AS1 | RUVBL1 | protein_coding | 0.50 | 0.000800911 |
| AGAP2-AS1 | RAB3IP | protein_coding | 0.59 | 5.20E-05 |
| AGAP2-AS1 | SORD | protein_coding | -0.60 | 2.99E-05 |
| AGAP2-AS1 | GCK | protein_coding | -0.57 | 9.15E-05 |
| AGAP2-AS1 | INTS6 | protein_coding | -0.57 | 9.71E-05 |
| AGAP2-AS1 | ADH5 | protein_coding | -0.63 | 1.06E-05 |
| AGAP2-AS1 | CSNK1G1 | protein_coding | 0.58 | 8.54E-05 |
| AGAP2-AS1 | GPR88 | protein_coding | -0.62 | 1.64E-05 |
| AGAP2-AS1 | CACNB2 | protein_coding | -0.62 | 1.33E-05 |
| AGAP2-AS1 | TMEM184B | protein_coding | 0.70 | 2.79E-07 |
| AGAP2-AS1 | CEP152 | protein_coding | 0.65 | 4.00E-06 |
| AGAP2-AS1 | SLC25A14 | protein_coding | 0.58 | 7.30E-05 |
| AGAP2-AS1 | CBX8 | protein_coding | 0.57 | 0.000120341 |
| AGAP2-AS1 | UBP1 | protein_coding | -0.50 | 0.000770813 |
| AGAP2-AS1 | CYP4F2 | protein_coding | -0.55 | 0.000198664 |
| AGAP2-AS1 | RAB40A | protein_coding | 0.63 | 8.87E-06 |
| AGAP2-AS1 | TSPAN10 | protein_coding | 0.54 | 0.000292138 |
| AGAP2-AS1 | ACY1 | protein_coding | -0.64 | 8.11E-06 |
| AGAP2-AS1 | HNRNPA1 | protein_coding | 0.60 | 3.10E-05 |
| AGAP2-AS1 | SENP3 | protein_coding | 0.56 | 0.000139884 |
| AGAP2-AS1 | MFAP2 | protein_coding | 0.60 | 3.09E-05 |
| AGAP2-AS1 | SH3BGRL3 | protein_coding | 0.53 | 0.000386316 |
| AGAP2-AS1 | PANO1 | protein_coding | 0.53 | 0.000339171 |
| AGAP2-AS1 | PRXL2A | protein_coding | -0.66 | 2.38E-06 |
| AGAP2-AS1 | CAMTA1 | protein_coding | -0.54 | 0.000276073 |
| AGAP2-AS1 | HLF | protein_coding | -0.58 | 7.25E-05 |
| AGAP2-AS1 | TOR4A | protein_coding | 0.61 | 1.92E-05 |
| AGAP2-AS1 | AURKB | protein_coding | 0.70 | 4.21E-07 |
| AGAP2-AS1 | MBLAC2 | protein_coding | -0.55 | 0.000179348 |
| AGAP2-AS1 | POC5 | protein_coding | 0.57 | 9.75E-05 |
| AGAP2-AS1 | ZNF341 | protein_coding | 0.58 | 7.08E-05 |
| AGAP2-AS1 | PIK3R1 | protein_coding | -0.65 | 4.02E-06 |
| AGAP2-AS1 | TUT7 | protein_coding | -0.54 | 0.000264454 |
| AGAP2-AS1 | ADAM9 | protein_coding | 0.60 | 3.43E-05 |
| AGAP2-AS1 | MYO7A | protein_coding | -0.53 | 0.000395394 |
| AGAP2-AS1 | PACS2 | protein_coding | 0.61 | 2.07E-05 |
| AGAP2-AS1 | TRIM16 | protein_coding | 0.62 | 1.75E-05 |
| AGAP2-AS1 | MRAP | protein_coding | -0.53 | 0.00038424 |
| AGAP2-AS1 | ATP6V1E1 | protein_coding | 0.52 | 0.00053701 |
| AGAP2-AS1 | RPGRIP1L | protein_coding | 0.61 | 2.63E-05 |
| AGAP2-AS1 | NAT14 | protein_coding | 0.63 | 8.40E-06 |
| AGAP2-AS1 | CEP131 | protein_coding | 0.71 | 1.68E-07 |
| AGAP2-AS1 | CHMP3 | protein_coding | 0.71 | 2.22E-07 |
| AGAP2-AS1 | PEX14 | protein_coding | -0.51 | 0.000689438 |
| AGAP2-AS1 | PLIN3 | protein_coding | 0.51 | 0.000656431 |
| AGAP2-AS1 | LCE2D | protein_coding | -0.55 | 0.00018924 |
| AGAP2-AS1 | HPR | protein_coding | -0.54 | 0.000249513 |
| AGAP2-AS1 | NPHP4 | protein_coding | 0.58 | 8.46E-05 |
| AGAP2-AS1 | ASS1 | protein_coding | -0.55 | 0.000196875 |
| AGAP2-AS1 | MED15 | protein_coding | 0.67 | 1.69E-06 |
| AGAP2-AS1 | DRAM1 | protein_coding | 0.51 | 0.00071111 |
| AGAP2-AS1 | CDT1 | protein_coding | 0.75 | 2.19E-08 |
| AGAP2-AS1 | PNMA1 | protein_coding | 0.58 | 7.89E-05 |
| AGAP2-AS1 | HEMGN | protein_coding | -0.55 | 0.000184845 |
| AGAP2-AS1 | RIMKLA | protein_coding | 0.64 | 7.17E-06 |
| AGAP2-AS1 | RAI1 | protein_coding | 0.62 | 1.48E-05 |
| AGAP2-AS1 | S100A2 | protein_coding | 0.54 | 0.000303127 |
| AGAP2-AS1 | IGF2BP2 | protein_coding | 0.64 | 7.46E-06 |
| AGAP2-AS1 | TMED5 | protein_coding | -0.52 | 0.000565915 |
| AGAP2-AS1 | EIF4EBP3 | protein_coding | -0.52 | 0.000508396 |
| AGAP2-AS1 | NEIL3 | protein_coding | 0.56 | 0.00013207 |
| AGAP2-AS1 | ZBTB45 | protein_coding | 0.61 | 2.79E-05 |
| AGAP2-AS1 | NAT9 | protein_coding | 0.56 | 0.000137294 |
| AGAP2-AS1 | KIFC2 | protein_coding | 0.51 | 0.000698186 |
| AGAP2-AS1 | PIPOX | protein_coding | -0.63 | 1.08E-05 |
| AGAP2-AS1 | SNAPC2 | protein_coding | 0.72 | 9.81E-08 |
| AGAP2-AS1 | BRD3OS | protein_coding | 0.58 | 7.58E-05 |
| AGAP2-AS1 | CYP4F12 | protein_coding | -0.68 | 1.31E-06 |
| AGAP2-AS1 | REPS1 | protein_coding | -0.56 | 0.000132256 |
| AGAP2-AS1 | PHF19 | protein_coding | 0.75 | 2.09E-08 |
| AGAP2-AS1 | FAM171A2 | protein_coding | 0.53 | 0.000333733 |
| AGAP2-AS1 | IL13RA1 | protein_coding | -0.55 | 0.000199385 |
| AGAP2-AS1 | B4GALNT1 | protein_coding | 0.60 | 3.28E-05 |
| AGAP2-AS1 | BMP10 | protein_coding | -0.64 | 7.28E-06 |
| AGAP2-AS1 | CYP4F11 | protein_coding | -0.54 | 0.000234669 |
| AGAP2-AS1 | CST1 | protein_coding | 0.57 | 8.84E-05 |
| AGAP2-AS1 | GLRX | protein_coding | -0.50 | 0.000789009 |
| AGAP2-AS1 | BTBD10 | protein_coding | 0.57 | 8.62E-05 |
| AGAP2-AS1 | AC005041.1 | protein_coding | 0.71 | 1.56E-07 |
| AGAP2-AS1 | CCDC43 | protein_coding | 0.73 | 6.86E-08 |
| AGAP2-AS1 | ABHD12 | protein_coding | 0.56 | 0.000165877 |
| AGAP2-AS1 | ADAMTSL4 | protein_coding | -0.53 | 0.000346794 |
| AGAP2-AS1 | CFHR2 | protein_coding | -0.64 | 6.82E-06 |
| AGAP2-AS1 | PON1 | protein_coding | -0.55 | 0.000205375 |
| AGAP2-AS1 | STXBP2 | protein_coding | 0.52 | 0.00047858 |
| AGAP2-AS1 | PPP4R2 | protein_coding | -0.64 | 6.07E-06 |
| AGAP2-AS1 | MUC13 | protein_coding | 0.51 | 0.000735685 |
| AGAP2-AS1 | PPP1R13L | protein_coding | 0.60 | 3.28E-05 |
| AGAP2-AS1 | DMGDH | protein_coding | -0.61 | 1.92E-05 |
| AGAP2-AS1 | RAN | protein_coding | 0.59 | 4.21E-05 |
| AGAP2-AS1 | UAP1L1 | protein_coding | 0.51 | 0.000638518 |
| AGAP2-AS1 | STC2 | protein_coding | 0.56 | 0.000153494 |
| AGAP2-AS1 | UTP18 | protein_coding | 0.65 | 3.75E-06 |
| AGAP2-AS1 | TP73 | protein_coding | 0.67 | 1.61E-06 |
| AGAP2-AS1 | GPR162 | protein_coding | -0.50 | 0.000812414 |
| AGAP2-AS1 | CYP4F22 | protein_coding | -0.64 | 5.72E-06 |
| AGAP2-AS1 | MED25 | protein_coding | 0.53 | 0.000421377 |
| AGAP2-AS1 | PVALB | protein_coding | -0.58 | 8.53E-05 |
| AGAP2-AS1 | EPB41L4B | protein_coding | -0.54 | 0.00025628 |
| AGAP2-AS1 | PEMT | protein_coding | -0.60 | 3.18E-05 |
| AGAP2-AS1 | FUOM | protein_coding | -0.54 | 0.000245028 |
| AGAP2-AS1 | CERCAM | protein_coding | 0.66 | 2.44E-06 |
| AGAP2-AS1 | NME2 | protein_coding | 0.62 | 1.62E-05 |
| AGAP2-AS1 | TIGD5 | protein_coding | 0.63 | 9.48E-06 |
| AGAP2-AS1 | UGT1A3 | protein_coding | -0.58 | 6.20E-05 |
| AGAP2-AS1 | CTNNA3 | protein_coding | -0.66 | 2.24E-06 |
| AGAP2-AS1 | NEUROD6 | protein_coding | -0.60 | 3.35E-05 |
| AGAP2-AS1 | FITM1 | protein_coding | -0.61 | 2.46E-05 |
| AGAP2-AS1 | EIF1B | protein_coding | -0.54 | 0.000293997 |
| AGAP2-AS1 | NABP2 | protein_coding | 0.66 | 2.68E-06 |
| AGAP2-AS1 | IFT140 | protein_coding | 0.56 | 0.000128495 |
| AGAP2-AS1 | TPPP2 | protein_coding | -0.65 | 3.92E-06 |
| AGAP2-AS1 | TRIB1 | protein_coding | -0.62 | 1.72E-05 |
| AGAP2-AS1 | GINS1 | protein_coding | 0.71 | 2.35E-07 |
| AGAP2-AS1 | IFT52 | protein_coding | 0.67 | 1.46E-06 |
| AGAP2-AS1 | SS18L1 | protein_coding | -0.69 | 6.65E-07 |
| AGAP2-AS1 | CASP2 | protein_coding | 0.52 | 0.000470908 |
| AGAP2-AS1 | CIAPIN1 | protein_coding | 0.56 | 0.000130492 |
| AGAP2-AS1 | ZNF174 | protein_coding | 0.51 | 0.000647245 |
| AGAP2-AS1 | SMIM19 | protein_coding | -0.53 | 0.000389117 |
| AGAP2-AS1 | AKR1A1 | protein_coding | -0.57 | 0.000108511 |
| AGAP2-AS1 | ABCC6 | protein_coding | -0.57 | 0.000113003 |
| AGAP2-AS1 | DNAJC16 | protein_coding | -0.68 | 1.23E-06 |
| AGAP2-AS1 | UCK2 | protein_coding | 0.57 | 9.33E-05 |
| AGAP2-AS1 | BICC1 | protein_coding | 0.60 | 3.83E-05 |
| AGAP2-AS1 | PLCZ1 | protein_coding | -0.54 | 0.000267005 |
| AGAP2-AS1 | CECR2 | protein_coding | -0.60 | 2.89E-05 |
| AGAP2-AS1 | EVPLL | protein_coding | -0.64 | 6.77E-06 |
| AGAP2-AS1 | HGS | protein_coding | 0.57 | 9.07E-05 |
| AGAP2-AS1 | SYNGR3 | protein_coding | 0.58 | 8.11E-05 |
| AGAP2-AS1 | FLII | protein_coding | 0.55 | 0.000179196 |
| AGAP2-AS1 | TRIP13 | protein_coding | 0.73 | 4.99E-08 |
| AGAP2-AS1 | INAVA | protein_coding | 0.59 | 4.60E-05 |
| AGAP2-AS1 | SCAMP5 | protein_coding | 0.57 | 0.000111984 |
| AGAP2-AS1 | TCEA3 | protein_coding | -0.59 | 4.52E-05 |
| AGAP2-AS1 | TP53I3 | protein_coding | 0.51 | 0.000681427 |
| AGAP2-AS1 | CFL2 | protein_coding | -0.62 | 1.43E-05 |
| AGAP2-AS1 | GPM6A | protein_coding | -0.54 | 0.000236813 |
| AGAP2-AS1 | FAM81A | protein_coding | 0.64 | 6.34E-06 |
| AGAP2-AS1 | SFSWAP | protein_coding | 0.58 | 8.03E-05 |
| AGAP2-AS1 | CRPPA | protein_coding | -0.59 | 4.80E-05 |
| AGAP2-AS1 | LMNB2 | protein_coding | 0.73 | 7.96E-08 |
| AGAP2-AS1 | ATP1B2 | protein_coding | -0.59 | 5.04E-05 |
| AGAP2-AS1 | ANO4 | protein_coding | 0.60 | 2.86E-05 |
| AGAP2-AS1 | GPR3 | protein_coding | 0.55 | 0.000215091 |
| AGAP2-AS1 | SLC52A2 | protein_coding | 0.70 | 4.56E-07 |
| AGAP2-AS1 | KDM7A | protein_coding | -0.64 | 5.54E-06 |
| AGAP2-AS1 | HJV | protein_coding | -0.64 | 6.20E-06 |
| AGAP2-AS1 | TMEM158 | protein_coding | 0.52 | 0.000462219 |
| AGAP2-AS1 | XRCC6 | protein_coding | 0.56 | 0.00012901 |
| AGAP2-AS1 | SKA3 | protein_coding | 0.68 | 9.16E-07 |
| AGAP2-AS1 | SLC10A3 | protein_coding | 0.66 | 2.73E-06 |
| AGAP2-AS1 | NOS1 | protein_coding | -0.60 | 3.91E-05 |
| AGAP2-AS1 | RIC8A | protein_coding | 0.57 | 8.96E-05 |
| AGAP2-AS1 | TPMT | protein_coding | -0.57 | 9.46E-05 |
| AGAP2-AS1 | FSIP1 | protein_coding | 0.54 | 0.000262625 |
| AGAP2-AS1 | HARS2 | protein_coding | 0.52 | 0.000559443 |
| AGAP2-AS1 | SPNS2 | protein_coding | 0.52 | 0.000448122 |
| AGAP2-AS1 | THY1 | protein_coding | 0.61 | 2.82E-05 |
| AGAP2-AS1 | CPNE2 | protein_coding | 0.58 | 8.14E-05 |
| AGAP2-AS1 | SLCO1B3-SLCO1B7 | protein_coding | -0.63 | 8.75E-06 |
| AGAP2-AS1 | HP | protein_coding | -0.50 | 0.000792259 |
| AGAP2-AS1 | C21orf58 | protein_coding | 0.57 | 9.85E-05 |
| AGAP2-AS1 | SSRP1 | protein_coding | 0.61 | 2.23E-05 |
| AGAP2-AS1 | ACTL10 | protein_coding | 0.69 | 6.99E-07 |
| AGAP2-AS1 | GFRA1 | protein_coding | -0.72 | 1.47E-07 |
| AGAP2-AS1 | ZNF330 | protein_coding | -0.57 | 9.33E-05 |
| AGAP2-AS1 | AL139011.2 | protein_coding | -0.67 | 1.73E-06 |
| AGAP2-AS1 | TMEM170B | protein_coding | -0.56 | 0.000136214 |
| AGAP2-AS1 | ADM5 | protein_coding | 0.58 | 7.76E-05 |
| AGAP2-AS1 | SMIM8 | protein_coding | -0.51 | 0.000671868 |
| AGAP2-AS1 | TK1 | protein_coding | 0.68 | 9.69E-07 |
| AGAP2-AS1 | PLAU | protein_coding | 0.54 | 0.000243507 |
| AGAP2-AS1 | APOL6 | protein_coding | -0.66 | 3.38E-06 |
| AGAP2-AS1 | THRA | protein_coding | 0.60 | 3.17E-05 |
| AGAP2-AS1 | CD151 | protein_coding | 0.63 | 1.27E-05 |
| AGAP2-AS1 | CLEC4G | protein_coding | -0.63 | 9.03E-06 |
| AGAP2-AS1 | CDC37L1 | protein_coding | -0.71 | 2.47E-07 |
| AGAP2-AS1 | LRP1 | protein_coding | -0.66 | 3.18E-06 |
| AGAP2-AS1 | FANK1 | protein_coding | 0.54 | 0.000276602 |
| AGAP2-AS1 | HEY2 | protein_coding | -0.61 | 2.71E-05 |
| AGAP2-AS1 | COL4A1 | protein_coding | 0.50 | 0.000808843 |
| AGAP2-AS1 | FAM49B | protein_coding | 0.53 | 0.000418422 |
| AGAP2-AS1 | DEGS2 | protein_coding | 0.53 | 0.000424055 |
| AGAP2-AS1 | CENPE | protein_coding | 0.63 | 1.25E-05 |
| AGAP2-AS1 | KIF11 | protein_coding | 0.69 | 6.99E-07 |
| AGAP2-AS1 | HMGCS1 | protein_coding | -0.52 | 0.000487983 |
| AGAP2-AS1 | MUS81 | protein_coding | 0.51 | 0.00062595 |
| AGAP2-AS1 | SLC9A5 | protein_coding | 0.75 | 1.22E-08 |
| AGAP2-AS1 | GLTPD2 | protein_coding | -0.54 | 0.00029081 |
| AGAP2-AS1 | EXO1 | protein_coding | 0.65 | 4.75E-06 |
| AGAP2-AS1 | RAVER2 | protein_coding | 0.56 | 0.000155968 |
| AGAP2-AS1 | TCTEX1D1 | protein_coding | -0.66 | 2.32E-06 |
| AGAP2-AS1 | KRTAP5-1 | protein_coding | 0.53 | 0.000410887 |
| AGAP2-AS1 | PTMS | protein_coding | -0.53 | 0.000401062 |
| AGAP2-AS1 | AMHR2 | protein_coding | -0.53 | 0.000345224 |
| AGAP2-AS1 | CENPM | protein_coding | 0.69 | 6.55E-07 |
| AGAP2-AS1 | STMN3 | protein_coding | 0.62 | 1.65E-05 |
| AGAP2-AS1 | SMPD2 | protein_coding | 0.54 | 0.000252844 |
| AGAP2-AS1 | ARHGEF39 | protein_coding | 0.65 | 3.95E-06 |
| AGAP2-AS1 | KLF12 | protein_coding | -0.61 | 2.05E-05 |
| AGAP2-AS1 | MAN2B1 | protein_coding | 0.51 | 0.000579456 |
| AGAP2-AS1 | IQCC | protein_coding | 0.58 | 8.30E-05 |
| AGAP2-AS1 | LDLRAP1 | protein_coding | -0.56 | 0.000167497 |
| AGAP2-AS1 | ACOT2 | protein_coding | -0.65 | 4.41E-06 |
| AGAP2-AS1 | STAB2 | protein_coding | -0.63 | 1.05E-05 |
| AGAP2-AS1 | NARS2 | protein_coding | -0.57 | 9.20E-05 |
| AGAP2-AS1 | C15orf39 | protein_coding | 0.57 | 0.000106569 |
| AGAP2-AS1 | ABR | protein_coding | 0.75 | 2.11E-08 |
| AGAP2-AS1 | ZGPAT | protein_coding | -0.57 | 9.51E-05 |
| AGAP2-AS1 | MT-CO2 | protein_coding | -0.50 | 0.000829023 |
| AGAP2-AS1 | SH2D3A | protein_coding | 0.73 | 7.90E-08 |
| AGAP2-AS1 | MEI4 | protein_coding | -0.56 | 0.000158972 |
| AGAP2-AS1 | MGAT5 | protein_coding | 0.58 | 7.28E-05 |
| AGAP2-AS1 | STARD3NL | protein_coding | 0.66 | 2.16E-06 |
| AGAP2-AS1 | CREB5 | protein_coding | 0.55 | 0.000232565 |
| AGAP2-AS1 | GAL3ST1 | protein_coding | 0.53 | 0.000394128 |
| AGAP2-AS1 | ATAD5 | protein_coding | 0.58 | 6.04E-05 |
| AGAP2-AS1 | PDE4DIP | protein_coding | -0.59 | 4.18E-05 |
| AGAP2-AS1 | CWH43 | protein_coding | -0.54 | 0.000267389 |
| AGAP2-AS1 | KSR2 | protein_coding | 0.66 | 2.23E-06 |
| AGAP2-AS1 | C19orf54 | protein_coding | 0.56 | 0.000135623 |
| AGAP2-AS1 | PTPN14 | protein_coding | 0.60 | 3.76E-05 |
| AGAP2-AS1 | ARHGAP1 | protein_coding | 0.50 | 0.000820008 |
| AGAP2-AS1 | SLCO1A2 | protein_coding | -0.50 | 0.000860545 |
| AGAP2-AS1 | SMAD2 | protein_coding | 0.60 | 3.28E-05 |
| AGAP2-AS1 | SEC63 | protein_coding | -0.55 | 0.000190469 |
| AGAP2-AS1 | MTBP | protein_coding | 0.52 | 0.000542447 |
| AGAP2-AS1 | GINS4 | protein_coding | 0.60 | 2.91E-05 |
| AGAP2-AS1 | RAB25 | protein_coding | 0.59 | 4.95E-05 |
| AGAP2-AS1 | MT-CYB | protein_coding | -0.64 | 6.25E-06 |
| AGAP2-AS1 | SAT2 | protein_coding | -0.51 | 0.000627455 |
| AGAP2-AS1 | NECAB2 | protein_coding | -0.53 | 0.000342701 |
| AGAP2-AS1 | FBXO40 | protein_coding | -0.70 | 3.28E-07 |
| AGAP2-AS1 | ZNF431 | protein_coding | 0.68 | 9.76E-07 |
| AGAP2-AS1 | MT-ATP6 | protein_coding | -0.59 | 4.53E-05 |
| AGAP2-AS1 | TENT5A | protein_coding | -0.56 | 0.000128915 |
| AGAP2-AS1 | KIF25 | protein_coding | -0.53 | 0.000360303 |
| AGAP2-AS1 | RDH11 | protein_coding | -0.51 | 0.000715335 |
| AGAP2-AS1 | H2AW | protein_coding | 0.68 | 1.27E-06 |
| AGAP2-AS1 | GLYATL1 | protein_coding | -0.58 | 6.49E-05 |
| AGAP2-AS1 | RNF39 | protein_coding | 0.54 | 0.000318034 |
| AGAP2-AS1 | CFAP45 | protein_coding | 0.52 | 0.000527012 |
| AGAP2-AS1 | CYP4F3 | protein_coding | -0.53 | 0.000342075 |
| AGAP2-AS1 | NDRG3 | protein_coding | 0.66 | 2.32E-06 |
| AGAP2-AS1 | KCTD7 | protein_coding | 0.51 | 0.000594914 |
| AGAP2-AS1 | ANGPTL6 | protein_coding | -0.64 | 7.73E-06 |
| AGAP2-AS1 | GINS2 | protein_coding | 0.68 | 1.02E-06 |
| AGAP2-AS1 | DLGAP2 | protein_coding | -0.63 | 8.99E-06 |
| AGAP2-AS1 | UBE2A | protein_coding | 0.67 | 1.70E-06 |
| AGAP2-AS1 | ARL16 | protein_coding | 0.54 | 0.000261981 |
| AGAP2-AS1 | NT5C3A | protein_coding | 0.60 | 4.12E-05 |
| AGAP2-AS1 | TRIM65 | protein_coding | 0.67 | 1.38E-06 |
| AGAP2-AS1 | TLR4 | protein_coding | -0.53 | 0.000429635 |
| AGAP2-AS1 | FANCD2 | protein_coding | 0.62 | 1.31E-05 |
| AGAP2-AS1 | MLXIPL | protein_coding | -0.55 | 0.000173234 |
| AGAP2-AS1 | SERPINA10 | protein_coding | -0.51 | 0.000636107 |
| AGAP2-AS1 | CCDC6 | protein_coding | 0.54 | 0.000290586 |
| AGAP2-AS1 | PEX19 | protein_coding | -0.69 | 7.35E-07 |
| AGAP2-AS1 | NRIP1 | protein_coding | -0.55 | 0.000215161 |
| AGAP2-AS1 | TES | protein_coding | 0.70 | 3.62E-07 |
| AGAP2-AS1 | COA1 | protein_coding | 0.57 | 0.000101208 |
| AGAP2-AS1 | CIP2A | protein_coding | 0.62 | 1.64E-05 |
| AGAP2-AS1 | SLC25A32 | protein_coding | -0.52 | 0.000542897 |
| AGAP2-AS1 | TTK | protein_coding | 0.65 | 3.53E-06 |
| AGAP2-AS1 | SPDL1 | protein_coding | 0.69 | 6.07E-07 |
| AGAP2-AS1 | EVX1 | protein_coding | 0.52 | 0.000457081 |
| AGAP2-AS1 | SERPINB6 | protein_coding | 0.53 | 0.00039599 |
| AGAP2-AS1 | UBE2M | protein_coding | 0.56 | 0.000154665 |
| AGAP2-AS1 | ALDH9A1 | protein_coding | -0.74 | 4.13E-08 |
| AGAP2-AS1 | PSME3IP1 | protein_coding | 0.61 | 2.33E-05 |
| AGAP2-AS1 | CLEC1B | protein_coding | -0.60 | 3.10E-05 |
| AGAP2-AS1 | COX19 | protein_coding | 0.56 | 0.00013602 |
| AGAP2-AS1 | FAHD2A | protein_coding | -0.56 | 0.000156073 |
| AGAP2-AS1 | RCC2 | protein_coding | 0.68 | 8.68E-07 |
| AGAP2-AS1 | INCENP | protein_coding | 0.62 | 1.65E-05 |
| AGAP2-AS1 | ST14 | protein_coding | 0.66 | 3.05E-06 |
| AGAP2-AS1 | ZSWIM1 | protein_coding | 0.55 | 0.000218777 |
| AGAP2-AS1 | TANC2 | protein_coding | 0.60 | 4.03E-05 |
| AGAP2-AS1 | ENPP1 | protein_coding | -0.59 | 5.01E-05 |
| AGAP2-AS1 | TCHP | protein_coding | 0.51 | 0.000690312 |
| AGAP2-AS1 | CPS1 | protein_coding | -0.58 | 6.85E-05 |
| AGAP2-AS1 | SLCO1B7 | protein_coding | -0.63 | 1.13E-05 |
| AGAP2-AS1 | MTA3 | protein_coding | 0.69 | 4.96E-07 |
| AGAP2-AS1 | TMCO3 | protein_coding | 0.55 | 0.000212808 |
| AGAP2-AS1 | MAP3K12 | protein_coding | 0.69 | 7.87E-07 |
| AGAP2-AS1 | GRAMD2A | protein_coding | 0.51 | 0.000653217 |
| AGAP2-AS1 | C6orf201 | protein_coding | -0.58 | 8.07E-05 |
| AGAP2-AS1 | PLVAP | protein_coding | 0.54 | 0.000298386 |
| AGAP2-AS1 | DCUN1D5 | protein_coding | 0.50 | 0.000801429 |
| AGAP2-AS1 | PZP | protein_coding | -0.56 | 0.000124171 |
| AGAP2-AS1 | LCMT1 | protein_coding | 0.68 | 9.27E-07 |
| AGAP2-AS1 | COL9A1 | protein_coding | 0.60 | 3.20E-05 |
| AGAP2-AS1 | 43892 | protein_coding | -0.57 | 8.70E-05 |
| AGAP2-AS1 | DSG2 | protein_coding | 0.53 | 0.000424314 |
| AGAP2-AS1 | SLC47A1 | protein_coding | -0.65 | 3.94E-06 |
| AGAP2-AS1 | MROH2B | protein_coding | -0.57 | 0.000101241 |
| AGAP2-AS1 | FARSA | protein_coding | 0.52 | 0.000521693 |
| AGAP2-AS1 | NR1H4 | protein_coding | -0.51 | 0.000684056 |
| AGAP2-AS1 | DROSHA | protein_coding | 0.57 | 0.000118671 |
| AGAP2-AS1 | NUP85 | protein_coding | 0.56 | 0.000142502 |
| AGAP2-AS1 | SULT2A1 | protein_coding | -0.53 | 0.000412246 |
| AGAP2-AS1 | MPDZ | protein_coding | -0.60 | 3.47E-05 |
| AGAP2-AS1 | OXSM | protein_coding | -0.54 | 0.000284152 |
| AGAP2-AS1 | ZFP41 | protein_coding | 0.58 | 6.80E-05 |
| AGAP2-AS1 | WDR62 | protein_coding | 0.64 | 5.76E-06 |
| AGAP2-AS1 | COPS7B | protein_coding | 0.63 | 1.04E-05 |
| AGAP2-AS1 | C11orf71 | protein_coding | -0.55 | 0.000214389 |
| AGAP2-AS1 | RILPL1 | protein_coding | 0.51 | 0.000600725 |
| AGAP2-AS1 | FBXO41 | protein_coding | 0.55 | 0.000193644 |
| AGAP2-AS1 | FMN1 | protein_coding | 0.58 | 8.23E-05 |
| AGAP2-AS1 | TMEM106C | protein_coding | 0.73 | 7.90E-08 |
| AGAP2-AS1 | HPX | protein_coding | -0.61 | 2.63E-05 |
| AGAP2-AS1 | NFE2L3 | protein_coding | 0.55 | 0.000175151 |
| AGAP2-AS1 | KCTD10 | protein_coding | 0.69 | 4.84E-07 |
| AGAP2-AS1 | DHRS4L2 | protein_coding | -0.52 | 0.000438977 |
| AGAP2-AS1 | ATXN7L3 | protein_coding | 0.72 | 1.09E-07 |
| AGAP2-AS1 | SLC19A2 | protein_coding | -0.55 | 0.000171229 |
| AGAP2-AS1 | TRADD | protein_coding | 0.53 | 0.000339742 |
| AGAP2-AS1 | TRAPPC4 | protein_coding | 0.51 | 0.000582841 |
| AGAP2-AS1 | ZSCAN20 | protein_coding | 0.54 | 0.00025591 |
| AGAP2-AS1 | CCDC97 | protein_coding | 0.54 | 0.000277456 |
| AGAP2-AS1 | FZD4 | protein_coding | -0.51 | 0.000665081 |
| AGAP2-AS1 | ATN1 | protein_coding | 0.54 | 0.000233538 |
| AGAP2-AS1 | HADH | protein_coding | -0.63 | 1.04E-05 |
| AGAP2-AS1 | RBM4 | protein_coding | 0.51 | 0.000586561 |
| AGAP2-AS1 | CDH15 | protein_coding | -0.52 | 0.000555577 |
| AGAP2-AS1 | MIF4GD | protein_coding | 0.65 | 5.12E-06 |
| AGAP2-AS1 | MCM7 | protein_coding | 0.73 | 6.08E-08 |
| AGAP2-AS1 | CAPNS1 | protein_coding | 0.63 | 1.09E-05 |
| AGAP2-AS1 | TTC39B | protein_coding | -0.56 | 0.000155498 |
| AGAP2-AS1 | FCGBP | protein_coding | 0.53 | 0.000365714 |
| AGAP2-AS1 | MELK | protein_coding | 0.72 | 1.33E-07 |
| AGAP2-AS1 | RNF165 | protein_coding | -0.65 | 4.98E-06 |
| AGAP2-AS1 | GPR146 | protein_coding | -0.67 | 1.76E-06 |
| AGAP2-AS1 | MT1HL1 | protein_coding | -0.53 | 0.000388879 |
| AGAP2-AS1 | FAM131C | protein_coding | 0.54 | 0.000289537 |
| AGAP2-AS1 | ZNF429 | protein_coding | -0.59 | 5.05E-05 |
| AGAP2-AS1 | SERPINA12 | protein_coding | -0.56 | 0.000135884 |
| AGAP2-AS1 | DHX8 | protein_coding | 0.69 | 7.33E-07 |
| AGAP2-AS1 | REEP2 | protein_coding | 0.63 | 1.03E-05 |
| AGAP2-AS1 | SLC13A5 | protein_coding | -0.51 | 0.00065101 |
| AGAP2-AS1 | SHD | protein_coding | -0.62 | 1.71E-05 |
| AGAP2-AS1 | RALA | protein_coding | 0.65 | 4.38E-06 |
| AGAP2-AS1 | THRB | protein_coding | -0.56 | 0.000163836 |
| AGAP2-AS1 | DPF1 | protein_coding | 0.78 | 1.90E-09 |
| AGAP2-AS1 | ADK | protein_coding | -0.56 | 0.000122286 |
| AGAP2-AS1 | CLU | protein_coding | -0.63 | 8.45E-06 |
| AGAP2-AS1 | DEPDC1B | protein_coding | 0.72 | 1.32E-07 |
| AGAP2-AS1 | SRPX2 | protein_coding | 0.53 | 0.000424955 |
| AGAP2-AS1 | ADAMTS12 | protein_coding | 0.52 | 0.000493612 |
| AGAP2-AS1 | CCDC86 | protein_coding | 0.67 | 1.81E-06 |
| AGAP2-AS1 | C19orf12 | protein_coding | -0.72 | 1.10E-07 |
| AGAP2-AS1 | PNPO | protein_coding | -0.55 | 0.000210658 |
| AGAP2-AS1 | ECHDC2 | protein_coding | -0.66 | 3.02E-06 |
| AGAP2-AS1 | UNC5A | protein_coding | 0.60 | 2.92E-05 |
| AGAP2-AS1 | ABCC9 | protein_coding | -0.67 | 1.83E-06 |
| AGAP2-AS1 | ASCL5 | protein_coding | 0.55 | 0.000188595 |
| AGAP2-AS1 | MOB3A | protein_coding | 0.60 | 3.47E-05 |
| AGAP2-AS1 | LRRC49 | protein_coding | 0.69 | 5.82E-07 |
| AGAP2-AS1 | GTF3C2 | protein_coding | 0.64 | 7.85E-06 |
| AGAP2-AS1 | MFSD10 | protein_coding | 0.63 | 1.01E-05 |
| AGAP2-AS1 | HK1 | protein_coding | 0.52 | 0.000541719 |
| AGAP2-AS1 | PHYKPL | protein_coding | -0.51 | 0.000713697 |
| AGAP2-AS1 | ZNF275 | protein_coding | -0.71 | 2.05E-07 |
| AGAP2-AS1 | HOOK2 | protein_coding | 0.53 | 0.000408305 |
| AGAP2-AS1 | ACOT13 | protein_coding | -0.64 | 6.07E-06 |
| AGAP2-AS1 | NEU4 | protein_coding | -0.61 | 2.01E-05 |
| AGAP2-AS1 | C4BPB | protein_coding | -0.57 | 9.48E-05 |
| AGAP2-AS1 | PDLIM7 | protein_coding | 0.68 | 1.06E-06 |
| AGAP2-AS1 | LDB2 | protein_coding | -0.53 | 0.000428358 |
| AGAP2-AS1 | INO80E | protein_coding | 0.52 | 0.000441682 |
| AGAP2-AS1 | YWHAB | protein_coding | 0.64 | 6.72E-06 |
| AGAP2-AS1 | RYBP | protein_coding | -0.60 | 3.89E-05 |
| AGAP2-AS1 | CDC7 | protein_coding | 0.64 | 6.15E-06 |
| AGAP2-AS1 | ZDHHC1 | protein_coding | 0.61 | 2.60E-05 |
| AGAP2-AS1 | BCAR1 | protein_coding | 0.56 | 0.000121051 |
| AGAP2-AS1 | FKBP10 | protein_coding | 0.67 | 1.73E-06 |
| AGAP2-AS1 | COLCA2 | protein_coding | 0.63 | 1.14E-05 |
| AGAP2-AS1 | CA9 | protein_coding | 0.53 | 0.000431181 |
| AGAP2-AS1 | SLC38A3 | protein_coding | -0.53 | 0.000325394 |
| AGAP2-AS1 | SNTB1 | protein_coding | -0.55 | 0.000216606 |
| AGAP2-AS1 | TRIM59 | protein_coding | 0.65 | 4.44E-06 |
| AGAP2-AS1 | RPP14 | protein_coding | -0.61 | 2.41E-05 |
| AGAP2-AS1 | ZNF207 | protein_coding | 0.53 | 0.000372335 |
| AGAP2-AS1 | MEAK7 | protein_coding | 0.72 | 1.21E-07 |
| AGAP2-AS1 | MT-CO1 | protein_coding | -0.52 | 0.000483937 |
| AGAP2-AS1 | SEPSECS | protein_coding | -0.60 | 3.35E-05 |
| AGAP2-AS1 | MYLK2 | protein_coding | 0.67 | 1.41E-06 |
| AGAP2-AS1 | ADH1C | protein_coding | -0.51 | 0.000628548 |
| AGAP2-AS1 | ARG1 | protein_coding | -0.58 | 8.17E-05 |
| AGAP2-AS1 | BEST3 | protein_coding | 0.59 | 4.85E-05 |
| AGAP2-AS1 | SLC6A1 | protein_coding | -0.64 | 7.82E-06 |
| AGAP2-AS1 | CTH | protein_coding | -0.62 | 1.31E-05 |
| AGAP2-AS1 | UBL4A | protein_coding | 0.53 | 0.00040645 |
| AGAP2-AS1 | CTBP2 | protein_coding | 0.67 | 1.80E-06 |
| AGAP2-AS1 | ELFN1 | protein_coding | -0.61 | 1.94E-05 |
| AGAP2-AS1 | SHISA4 | protein_coding | 0.54 | 0.000314029 |
| AGAP2-AS1 | SPAG1 | protein_coding | 0.51 | 0.000644998 |
| AGAP2-AS1 | NUP62 | protein_coding | 0.55 | 0.000171945 |
| AGAP2-AS1 | RAB38 | protein_coding | 0.54 | 0.000257739 |
| AGAP2-AS1 | UNC119B | protein_coding | 0.63 | 1.26E-05 |
| AGAP2-AS1 | RD3L | protein_coding | -0.66 | 3.07E-06 |
| AGAP2-AS1 | CYP4A22 | protein_coding | -0.60 | 3.80E-05 |
| AGAP2-AS1 | CYP3A7 | protein_coding | -0.54 | 0.000275463 |
| AGAP2-AS1 | DDN | protein_coding | 0.70 | 3.10E-07 |
| AGAP2-AS1 | UIMC1 | protein_coding | 0.62 | 1.64E-05 |
| AGAP2-AS1 | RBMX2 | protein_coding | 0.62 | 1.63E-05 |
| AGAP2-AS1 | NOL4L | protein_coding | 0.50 | 0.000794229 |
| AGAP2-AS1 | MSRA | protein_coding | -0.56 | 0.000163343 |
| AGAP2-AS1 | MICAL1 | protein_coding | 0.60 | 4.02E-05 |
| AGAP2-AS1 | TMUB2 | protein_coding | 0.64 | 6.07E-06 |
| AGAP2-AS1 | SLC39A14 | protein_coding | -0.55 | 0.00022649 |
| AGAP2-AS1 | RGPD3 | protein_coding | -0.53 | 0.000372227 |
| AGAP2-AS1 | MARS1 | protein_coding | 0.50 | 0.000857342 |
| AGAP2-AS1 | HMGCR | protein_coding | -0.51 | 0.000719 |
| AGAP2-AS1 | INPP5F | protein_coding | 0.55 | 0.00018646 |
| AGAP2-AS1 | PKMYT1 | protein_coding | 0.71 | 1.93E-07 |
| AGAP2-AS1 | DHX40 | protein_coding | 0.55 | 0.000229781 |
| AGAP2-AS1 | STEAP4 | protein_coding | -0.58 | 6.01E-05 |
| AGAP2-AS1 | N4BP2L1 | protein_coding | -0.72 | 1.21E-07 |
| AGAP2-AS1 | SOGA1 | protein_coding | 0.69 | 5.80E-07 |
| AGAP2-AS1 | KCNK12 | protein_coding | 0.62 | 1.64E-05 |
| AGAP2-AS1 | ZP3 | protein_coding | 0.58 | 8.28E-05 |
| AGAP2-AS1 | BCAS4 | protein_coding | 0.62 | 1.81E-05 |
| AGAP2-AS1 | ASB9 | protein_coding | -0.52 | 0.00056413 |
| AGAP2-AS1 | RAD54L | protein_coding | 0.70 | 3.25E-07 |
| AGAP2-AS1 | ACOT9 | protein_coding | 0.70 | 3.67E-07 |
| AGAP2-AS1 | SLC7A2 | protein_coding | -0.67 | 1.60E-06 |
| AGAP2-AS1 | ORC1 | protein_coding | 0.69 | 5.88E-07 |
| AGAP2-AS1 | FAM13A | protein_coding | -0.56 | 0.000131741 |
| AGAP2-AS1 | PSORS1C1 | protein_coding | 0.69 | 7.05E-07 |
| AGAP2-AS1 | PADI2 | protein_coding | 0.51 | 0.000588867 |
| AGAP2-AS1 | OSBPL11 | protein_coding | -0.58 | 6.20E-05 |
| AGAP2-AS1 | CHRNB2 | protein_coding | 0.52 | 0.000545539 |
| AGAP2-AS1 | RTL8C | protein_coding | 0.71 | 2.55E-07 |
| AGAP2-AS1 | ZFP90 | protein_coding | 0.51 | 0.000761923 |
| AGAP2-AS1 | TMEM145 | protein_coding | 0.54 | 0.000290728 |
| AGAP2-AS1 | FBXO43 | protein_coding | 0.55 | 0.000230362 |
| AGAP2-AS1 | TRIM36 | protein_coding | 0.52 | 0.000455791 |
| AGAP2-AS1 | TNFRSF11A | protein_coding | 0.63 | 1.22E-05 |
| AGAP2-AS1 | SPATA17 | protein_coding | 0.58 | 7.18E-05 |
| AGAP2-AS1 | MT-ND4L | protein_coding | -0.61 | 2.23E-05 |
| AGAP2-AS1 | ACLY | protein_coding | 0.60 | 3.51E-05 |
| AGAP2-AS1 | NIPSNAP3A | protein_coding | -0.52 | 0.000435374 |
| AGAP2-AS1 | CYP2A13 | protein_coding | -0.60 | 2.85E-05 |
| AGAP2-AS1 | TMSB15A | protein_coding | 0.51 | 0.000752956 |
| AGAP2-AS1 | LYSMD3 | protein_coding | -0.56 | 0.000157068 |
| AGAP2-AS1 | PLIN1 | protein_coding | -0.64 | 6.99E-06 |
| AGAP2-AS1 | ECHS1 | protein_coding | -0.60 | 3.37E-05 |
| AGAP2-AS1 | ACSL1 | protein_coding | -0.68 | 1.01E-06 |
| AGAP2-AS1 | TPBG | protein_coding | 0.56 | 0.000140091 |
| AGAP2-AS1 | FZD1 | protein_coding | 0.51 | 0.000716025 |
| AGAP2-AS1 | RGSL1 | protein_coding | -0.59 | 5.91E-05 |
| AGAP2-AS1 | ISOC1 | protein_coding | -0.66 | 2.90E-06 |
| AGAP2-AS1 | SLC10A1 | protein_coding | -0.57 | 9.66E-05 |
| AGAP2-AS1 | ALOXE3 | protein_coding | 0.67 | 2.07E-06 |
| AGAP2-AS1 | NR1D2 | protein_coding | -0.62 | 1.89E-05 |
| AGAP2-AS1 | MT1G | protein_coding | -0.62 | 1.89E-05 |
| AGAP2-AS1 | DUSP18 | protein_coding | 0.60 | 3.72E-05 |
| AGAP2-AS1 | PCNA | protein_coding | 0.64 | 5.91E-06 |
| AGAP2-AS1 | UROC1 | protein_coding | -0.64 | 7.89E-06 |
| AGAP2-AS1 | C8B | protein_coding | -0.55 | 0.000202669 |
| AGAP2-AS1 | IL1RL2 | protein_coding | -0.54 | 0.000278194 |
| AGAP2-AS1 | PLCD3 | protein_coding | 0.72 | 1.02E-07 |
| AGAP2-AS1 | ZBTB12 | protein_coding | 0.60 | 3.24E-05 |
| AGAP2-AS1 | AC010531.1 | protein_coding | -0.55 | 0.00021329 |
| AGAP2-AS1 | CLSTN1 | protein_coding | 0.54 | 0.000242178 |
| AGAP2-AS1 | ACOT4 | protein_coding | -0.65 | 5.10E-06 |
| AGAP2-AS1 | PANK1 | protein_coding | -0.62 | 1.62E-05 |
| AGAP2-AS1 | MRGBP | protein_coding | 0.57 | 0.000118454 |
| AGAP2-AS1 | PFKFB1 | protein_coding | -0.66 | 2.74E-06 |
| AGAP2-AS1 | KMT5C | protein_coding | 0.55 | 0.000205185 |
| AGAP2-AS1 | TPRG1 | protein_coding | -0.66 | 2.89E-06 |
| AGAP2-AS1 | APMAP | protein_coding | -0.53 | 0.000337645 |
| AGAP2-AS1 | EAF1 | protein_coding | -0.50 | 0.000813509 |
| AGAP2-AS1 | MMP14 | protein_coding | 0.68 | 1.27E-06 |
| AGAP2-AS1 | BOP1 | protein_coding | 0.55 | 0.000222916 |
| AGAP2-AS1 | LAMB1 | protein_coding | 0.61 | 1.97E-05 |
| AGAP2-AS1 | MTHFS | protein_coding | -0.54 | 0.000250117 |
| AGAP2-AS1 | NIBAN2 | protein_coding | 0.58 | 6.45E-05 |
| AGAP2-AS1 | ESPN | protein_coding | -0.52 | 0.000438568 |
| AGAP2-AS1 | COL25A1 | protein_coding | -0.59 | 4.87E-05 |
| AGAP2-AS1 | RXRG | protein_coding | -0.51 | 0.0005883 |
| AGAP2-AS1 | PDE1C | protein_coding | 0.62 | 1.63E-05 |
| AGAP2-AS1 | PLIN5 | protein_coding | -0.60 | 3.26E-05 |
| AGAP2-AS1 | JPT1 | protein_coding | 0.59 | 5.33E-05 |
| AGAP2-AS1 | LOXL3 | protein_coding | 0.55 | 0.000216272 |
| AGAP2-AS1 | C11orf54 | protein_coding | -0.58 | 7.12E-05 |
| AGAP2-AS1 | LIMK1 | protein_coding | 0.70 | 3.08E-07 |
| AGAP2-AS1 | CEP104 | protein_coding | -0.56 | 0.000135756 |
| AGAP2-AS1 | CD58 | protein_coding | 0.60 | 3.55E-05 |
| AGAP2-AS1 | UBALD2 | protein_coding | 0.62 | 1.31E-05 |
| AGAP2-AS1 | UGT1A1 | protein_coding | -0.56 | 0.000131451 |
| AGAP2-AS1 | SLC35F2 | protein_coding | 0.59 | 5.32E-05 |
| AGAP2-AS1 | RAPH1 | protein_coding | -0.50 | 0.000771267 |
| AGAP2-AS1 | ABCC2 | protein_coding | -0.55 | 0.000218088 |
| AGAP2-AS1 | CUEDC1 | protein_coding | 0.69 | 5.64E-07 |
| AGAP2-AS1 | AC023055.1 | protein_coding | 0.51 | 0.000728746 |
| AGAP2-AS1 | CTSV | protein_coding | 0.68 | 1.22E-06 |
| AGAP2-AS1 | TNFSF9 | protein_coding | 0.54 | 0.000241412 |
| AGAP2-AS1 | SFXN3 | protein_coding | 0.72 | 1.49E-07 |
| AGAP2-AS1 | DLX4 | protein_coding | 0.59 | 4.70E-05 |
| AGAP2-AS1 | TTL | protein_coding | 0.54 | 0.000286497 |
| AGAP2-AS1 | POLR2G | protein_coding | 0.67 | 1.59E-06 |
| AGAP2-AS1 | PKLR | protein_coding | -0.56 | 0.000127294 |
| AGAP2-AS1 | KIAA0753 | protein_coding | 0.53 | 0.000330664 |
| AGAP2-AS1 | GSTA1 | protein_coding | -0.54 | 0.000286307 |
| AGAP2-AS1 | B3GALT4 | protein_coding | 0.57 | 0.00011636 |
| AGAP2-AS1 | C2CD4C | protein_coding | 0.55 | 0.000225182 |
| AGAP2-AS1 | HMGA2 | protein_coding | 0.56 | 0.000138107 |
| AGAP2-AS1 | USP38 | protein_coding | -0.57 | 0.000111028 |
| AGAP2-AS1 | SERTM2 | protein_coding | -0.63 | 1.24E-05 |
| AGAP2-AS1 | NHLRC3 | protein_coding | -0.58 | 8.04E-05 |
| AGAP2-AS1 | CFL1 | protein_coding | 0.66 | 2.84E-06 |
| AGAP2-AS1 | DOCK4 | protein_coding | -0.52 | 0.000535479 |
| AGAP2-AS1 | OLFML2A | protein_coding | 0.62 | 1.71E-05 |
| AGAP2-AS1 | JAG1 | protein_coding | 0.58 | 8.12E-05 |
| AGAP2-AS1 | U2AF2 | protein_coding | 0.58 | 6.13E-05 |
| AGAP2-AS1 | LAMB3 | protein_coding | 0.57 | 0.000110168 |
| AGAP2-AS1 | TMEM220 | protein_coding | -0.65 | 3.98E-06 |
| AGAP2-AS1 | PRMT2 | protein_coding | 0.60 | 3.58E-05 |
| AGAP2-AS1 | CLVS1 | protein_coding | 0.53 | 0.000385106 |
| AGAP2-AS1 | F8 | protein_coding | -0.54 | 0.000287179 |
| AGAP2-AS1 | SLC6A8 | protein_coding | 0.54 | 0.000273796 |
| AGAP2-AS1 | COX6A2 | protein_coding | -0.56 | 0.000122974 |
| AGAP2-AS1 | ABCG2 | protein_coding | -0.69 | 6.26E-07 |
| AGAP2-AS1 | CENPK | protein_coding | 0.68 | 1.20E-06 |
| AGAP2-AS1 | ALAD | protein_coding | -0.58 | 6.94E-05 |
| AGAP2-AS1 | PTP4A3 | protein_coding | 0.63 | 9.86E-06 |
| AGAP2-AS1 | POLD3 | protein_coding | 0.55 | 0.000203864 |
| AGAP2-AS1 | KNTC1 | protein_coding | 0.65 | 5.24E-06 |
| AGAP2-AS1 | DDT | protein_coding | -0.56 | 0.000152527 |
| AGAP2-AS1 | TMEM254 | protein_coding | -0.57 | 9.39E-05 |
| AGAP2-AS1 | GHR | protein_coding | -0.54 | 0.000300321 |
| AGAP2-AS1 | KCTD13 | protein_coding | 0.64 | 7.90E-06 |
| AGAP2-AS1 | EDIL3 | protein_coding | 0.51 | 0.000627269 |
| AGAP2-AS1 | PRKCG | protein_coding | 0.53 | 0.000375041 |
| AGAP2-AS1 | TBC1D10B | protein_coding | 0.75 | 1.23E-08 |
| AGAP2-AS1 | MCEE | protein_coding | -0.63 | 1.16E-05 |
| AGAP2-AS1 | ZNF296 | protein_coding | 0.56 | 0.000145958 |
| AGAP2-AS1 | SELENOP | protein_coding | -0.71 | 1.99E-07 |
| AGAP2-AS1 | STIMATE-MUSTN1 | protein_coding | -0.57 | 0.00010121 |
| AGAP2-AS1 | RRM2 | protein_coding | 0.66 | 2.29E-06 |
| AGAP2-AS1 | BBC3 | protein_coding | 0.51 | 0.000580488 |
| AGAP2-AS1 | VPS37C | protein_coding | 0.70 | 2.83E-07 |
| AGAP2-AS1 | CLCF1 | protein_coding | 0.51 | 0.000617062 |
| AGAP2-AS1 | TCEAL9 | protein_coding | 0.57 | 9.55E-05 |
| AGAP2-AS1 | USH2A | protein_coding | -0.73 | 7.96E-08 |
| AGAP2-AS1 | NPEPPS | protein_coding | 0.59 | 4.69E-05 |
| AGAP2-AS1 | S100A11 | protein_coding | 0.63 | 1.09E-05 |
| AGAP2-AS1 | HTRA3 | protein_coding | 0.57 | 9.36E-05 |
| AGAP2-AS1 | GALNT10 | protein_coding | 0.69 | 7.46E-07 |
| AGAP2-AS1 | CCDC93 | protein_coding | 0.60 | 3.68E-05 |
| AGAP2-AS1 | MRPS28 | protein_coding | -0.59 | 5.23E-05 |
| AGAP2-AS1 | ALOX12B | protein_coding | 0.54 | 0.000295504 |
| AGAP2-AS1 | ERCC3 | protein_coding | 0.61 | 2.02E-05 |
| AGAP2-AS1 | PFKP | protein_coding | 0.76 | 6.83E-09 |
| AGAP2-AS1 | DNMT3L | protein_coding | -0.68 | 1.01E-06 |
| AGAP2-AS1 | B4GALNT2 | protein_coding | 0.65 | 3.62E-06 |
| AGAP2-AS1 | PPM1A | protein_coding | -0.61 | 2.68E-05 |
| AGAP2-AS1 | ACAT2 | protein_coding | -0.59 | 4.77E-05 |
| AGAP2-AS1 | CEP112 | protein_coding | 0.66 | 2.17E-06 |
| AGAP2-AS1 | ZNF319 | protein_coding | 0.50 | 0.000834199 |
| AGAP2-AS1 | C8orf33 | protein_coding | 0.52 | 0.000532834 |
| AGAP2-AS1 | EDEM1 | protein_coding | -0.56 | 0.000167556 |
| AGAP2-AS1 | ACR | protein_coding | -0.58 | 8.30E-05 |
| AGAP2-AS1 | MFSD8 | protein_coding | -0.52 | 0.000451225 |
| AGAP2-AS1 | C9 | protein_coding | -0.50 | 0.000834593 |
| AGAP2-AS1 | TGDS | protein_coding | -0.61 | 2.68E-05 |
| AGAP2-AS1 | SGK3 | protein_coding | -0.52 | 0.000553354 |
| AGAP2-AS1 | CPEB4 | protein_coding | -0.50 | 0.000803387 |
| AGAP2-AS1 | GALNT6 | protein_coding | 0.51 | 0.000674442 |
| AGAP2-AS1 | BICD1 | protein_coding | 0.61 | 2.10E-05 |
| AGAP2-AS1 | RIN1 | protein_coding | 0.68 | 1.03E-06 |
| AGAP2-AS1 | ANKRD46 | protein_coding | -0.64 | 7.36E-06 |
| AGAP2-AS1 | APOC3 | protein_coding | -0.50 | 0.000772983 |
| AGAP2-AS1 | SAMD1 | protein_coding | 0.55 | 0.000200303 |
| AGAP2-AS1 | RAB35 | protein_coding | 0.55 | 0.000222114 |
| AGAP2-AS1 | ERMARD | protein_coding | -0.51 | 0.000742419 |
| AGAP2-AS1 | BTNL9 | protein_coding | -0.58 | 7.05E-05 |
| AGAP2-AS1 | MADD | protein_coding | 0.56 | 0.000129949 |
| AGAP2-AS1 | NAAA | protein_coding | -0.56 | 0.000125253 |
| AGAP2-AS1 | CD248 | protein_coding | 0.51 | 0.000640784 |
| AGAP2-AS1 | MAF1 | protein_coding | 0.54 | 0.000239497 |
| AGAP2-AS1 | TRMT6 | protein_coding | 0.55 | 0.000208194 |
| AGAP2-AS1 | WDHD1 | protein_coding | 0.65 | 4.03E-06 |
| AGAP2-AS1 | MT-ND5 | protein_coding | -0.65 | 4.48E-06 |
| AGAP2-AS1 | EHMT2 | protein_coding | 0.52 | 0.000544313 |
| AGAP2-AS1 | ARMCX6 | protein_coding | 0.61 | 2.62E-05 |
| AGAP2-AS1 | UNC93A | protein_coding | -0.58 | 6.37E-05 |
| AGAP2-AS1 | CERS4 | protein_coding | -0.51 | 0.000766233 |
| AGAP2-AS1 | PELI3 | protein_coding | 0.54 | 0.000284954 |
| AGAP2-AS1 | UNC13D | protein_coding | 0.55 | 0.000229493 |
| AGAP2-AS1 | CDPF1 | protein_coding | 0.56 | 0.000161181 |
| AGAP2-AS1 | GALNT16 | protein_coding | -0.53 | 0.000336715 |
| AGAP2-AS1 | PRKD1 | protein_coding | 0.62 | 1.44E-05 |
| AGAP2-AS1 | SLC26A1 | protein_coding | -0.70 | 3.24E-07 |
| AGAP2-AS1 | ARRDC4 | protein_coding | -0.56 | 0.000157552 |
| AGAP2-AS1 | KHK | protein_coding | -0.61 | 2.21E-05 |
| AGAP2-AS1 | TIMM22 | protein_coding | 0.56 | 0.000154222 |
| AGAP2-AS1 | SF3B3 | protein_coding | 0.59 | 4.26E-05 |
| AGAP2-AS1 | PPP1R3G | protein_coding | -0.51 | 0.000726448 |
| AGAP2-AS1 | HPS5 | protein_coding | -0.58 | 8.30E-05 |
| AGAP2-AS1 | SYCE1L | protein_coding | 0.63 | 8.90E-06 |
| AGAP2-AS1 | CYBA | protein_coding | 0.55 | 0.000199814 |
| AGAP2-AS1 | SLC39A10 | protein_coding | 0.58 | 6.91E-05 |
| AGAP2-AS1 | MPP2 | protein_coding | 0.58 | 6.02E-05 |
| AGAP2-AS1 | GNPNAT1 | protein_coding | -0.53 | 0.000429714 |
| AGAP2-AS1 | HKDC1 | protein_coding | 0.59 | 5.36E-05 |
| AGAP2-AS1 | CEND1 | protein_coding | 0.54 | 0.000275919 |
| AGAP2-AS1 | HES7 | protein_coding | 0.50 | 0.00084198 |
| AGAP2-AS1 | CANT1 | protein_coding | 0.68 | 1.29E-06 |
| AGAP2-AS1 | C1orf115 | protein_coding | -0.70 | 3.87E-07 |
| AGAP2-AS1 | RAB11FIP5 | protein_coding | 0.61 | 2.07E-05 |
| AGAP2-AS1 | AGL | protein_coding | -0.67 | 1.92E-06 |
| AGAP2-AS1 | GYS2 | protein_coding | -0.63 | 1.02E-05 |
| AGAP2-AS1 | ZADH2 | protein_coding | -0.55 | 0.000215665 |
| AGAP2-AS1 | TMEM190 | protein_coding | 0.67 | 1.36E-06 |
| AGAP2-AS1 | SYDE1 | protein_coding | 0.52 | 0.000524185 |
| AGAP2-AS1 | IL11 | protein_coding | 0.76 | 1.08E-08 |
| AGAP2-AS1 | SEC16B | protein_coding | -0.53 | 0.000328066 |
| AGAP2-AS1 | ETS2 | protein_coding | -0.62 | 1.92E-05 |
| AGAP2-AS1 | DGAT1 | protein_coding | -0.58 | 6.59E-05 |
| AGAP2-AS1 | SYT7 | protein_coding | -0.56 | 0.000151986 |
| AGAP2-AS1 | FGD1 | protein_coding | 0.54 | 0.000275999 |
| AGAP2-AS1 | C1orf167 | protein_coding | -0.55 | 0.000226458 |
| AGAP2-AS1 | PHEX | protein_coding | 0.66 | 3.12E-06 |
| AGAP2-AS1 | RHBDF2 | protein_coding | 0.66 | 2.45E-06 |
| AGAP2-AS1 | TCP10L2 | protein_coding | -0.69 | 5.80E-07 |
| AGAP2-AS1 | PTMA | protein_coding | 0.58 | 7.70E-05 |
| AGAP2-AS1 | IGSF3 | protein_coding | 0.61 | 1.97E-05 |
| AGAP2-AS1 | ORAI2 | protein_coding | 0.67 | 1.53E-06 |
| AGAP2-AS1 | EZH2 | protein_coding | 0.68 | 9.46E-07 |
| AGAP2-AS1 | NKX2-8 | protein_coding | 0.62 | 1.91E-05 |
| AGAP2-AS1 | FBP1 | protein_coding | -0.66 | 3.05E-06 |
| AGAP2-AS1 | APOBEC3C | protein_coding | 0.50 | 0.000850134 |
| AGAP2-AS1 | PLEKHB2 | protein_coding | 0.54 | 0.000293245 |
| AGAP2-AS1 | LIN7C | protein_coding | -0.61 | 2.45E-05 |
| AGAP2-AS1 | ALYREF | protein_coding | 0.64 | 6.01E-06 |
| AGAP2-AS1 | RBCK1 | protein_coding | 0.54 | 0.000287523 |
| AGAP2-AS1 | CPSF3 | protein_coding | 0.53 | 0.000379426 |
| AGAP2-AS1 | LECT2 | protein_coding | -0.55 | 0.000218107 |
| AGAP2-AS1 | LHB | protein_coding | 0.65 | 3.99E-06 |
| AGAP2-AS1 | NPM3 | protein_coding | 0.56 | 0.000164509 |
| AGAP2-AS1 | ARHGAP33 | protein_coding | 0.56 | 0.000143079 |
| AGAP2-AS1 | LRRFIP2 | protein_coding | -0.58 | 7.84E-05 |
| AGAP2-AS1 | OTX1 | protein_coding | 0.58 | 6.48E-05 |
| AGAP2-AS1 | FNDC10 | protein_coding | 0.68 | 1.02E-06 |
| AGAP2-AS1 | UBL7 | protein_coding | 0.56 | 0.000157096 |
| AGAP2-AS1 | RHOV | protein_coding | 0.58 | 8.11E-05 |
| AGAP2-AS1 | KANK4 | protein_coding | -0.58 | 6.96E-05 |
| AGAP2-AS1 | CCNJL | protein_coding | 0.61 | 2.52E-05 |
| AGAP2-AS1 | APOA1 | protein_coding | -0.50 | 0.000855544 |
| AGAP2-AS1 | PARPBP | protein_coding | 0.73 | 4.92E-08 |
| AGAP2-AS1 | AC068580.4 | protein_coding | 0.59 | 5.01E-05 |
| AGAP2-AS1 | SLC4A11 | protein_coding | 0.53 | 0.000406153 |
| AGAP2-AS1 | ADRB2 | protein_coding | -0.60 | 3.96E-05 |
| AGAP2-AS1 | C11orf80 | protein_coding | 0.68 | 1.15E-06 |
| AGAP2-AS1 | HAMP | protein_coding | -0.58 | 6.99E-05 |
| AGAP2-AS1 | ZNF654 | protein_coding | -0.57 | 0.000118607 |
| AGAP2-AS1 | FLNA | protein_coding | 0.65 | 4.59E-06 |
| AGAP2-AS1 | VIP | protein_coding | -0.52 | 0.000545823 |
| AGAP2-AS1 | DPP4 | protein_coding | -0.55 | 0.000209263 |
| AGAP2-AS1 | CARNMT1 | protein_coding | -0.54 | 0.000281714 |
| AGAP2-AS1 | RASEF | protein_coding | 0.55 | 0.00019481 |
| AGAP2-AS1 | CENPO | protein_coding | 0.74 | 3.30E-08 |
| AGAP2-AS1 | LARS1 | protein_coding | 0.56 | 0.000124241 |
| AGAP2-AS1 | MTM1 | protein_coding | -0.64 | 5.76E-06 |
| AGAP2-AS1 | SMOC1 | protein_coding | -0.50 | 0.00080924 |
| AGAP2-AS1 | CYP1A2 | protein_coding | -0.60 | 4.04E-05 |
| AGAP2-AS1 | NT5DC1 | protein_coding | -0.58 | 6.70E-05 |
| AGAP2-AS1 | CHRD | protein_coding | -0.51 | 0.000683994 |
| AGAP2-AS1 | IGFBP3 | protein_coding | -0.51 | 0.000655229 |
| AGAP2-AS1 | SLC25A6 | protein_coding | 0.53 | 0.000354722 |
| AGAP2-AS1 | IRAK1 | protein_coding | 0.63 | 1.10E-05 |
| AGAP2-AS1 | FAM171B | protein_coding | 0.63 | 9.92E-06 |
| AGAP2-AS1 | SAMD5 | protein_coding | -0.51 | 0.000737223 |
| AGAP2-AS1 | ATL2 | protein_coding | -0.56 | 0.000140034 |
| AGAP2-AS1 | MFSD5 | protein_coding | 0.68 | 1.10E-06 |
| AGAP2-AS1 | CMBL | protein_coding | -0.55 | 0.000203834 |
| AGAP2-AS1 | APOC4-APOC2 | protein_coding | -0.64 | 7.42E-06 |
| AGAP2-AS1 | TLK2 | protein_coding | 0.52 | 0.000486735 |
| AGAP2-AS1 | TAT | protein_coding | -0.55 | 0.000182345 |
| AGAP2-AS1 | ABHD6 | protein_coding | -0.55 | 0.000216046 |
| AGAP2-AS1 | GREM2 | protein_coding | -0.60 | 3.39E-05 |
| AGAP2-AS1 | ANKRD52 | protein_coding | 0.58 | 7.73E-05 |
| AGAP2-AS1 | APOE | protein_coding | -0.63 | 1.22E-05 |
| AGAP2-AS1 | LRFN4 | protein_coding | 0.63 | 8.47E-06 |
| AGAP2-AS1 | SPECC1 | protein_coding | 0.70 | 4.73E-07 |
| AGAP2-AS1 | RCN2 | protein_coding | 0.67 | 1.75E-06 |
| AGAP2-AS1 | TRIM47 | protein_coding | 0.61 | 2.64E-05 |
| AGAP2-AS1 | RNF146 | protein_coding | -0.53 | 0.000373757 |
| AGAP2-AS1 | FUNDC1 | protein_coding | 0.63 | 1.07E-05 |
| AGAP2-AS1 | MLIP | protein_coding | -0.57 | 9.09E-05 |
| AGAP2-AS1 | TUBG1 | protein_coding | 0.56 | 0.000162427 |
| AGAP2-AS1 | TTC9C | protein_coding | 0.68 | 8.80E-07 |
| AGAP2-AS1 | PKN1 | protein_coding | 0.65 | 5.40E-06 |
| AGAP2-AS1 | LONRF3 | protein_coding | -0.52 | 0.000496549 |
| AGAP2-AS1 | CACNB3 | protein_coding | 0.68 | 1.21E-06 |
| AGAP2-AS1 | RAD51C | protein_coding | 0.53 | 0.000402764 |
| AGAP2-AS1 | ANG | protein_coding | -0.63 | 8.45E-06 |
| AGAP2-AS1 | GPER1 | protein_coding | -0.73 | 6.43E-08 |
| AGAP2-AS1 | MARK2 | protein_coding | 0.65 | 4.62E-06 |
| AGAP2-AS1 | ANXA11 | protein_coding | 0.59 | 5.54E-05 |
| AGAP2-AS1 | NXNL2 | protein_coding | 0.77 | 4.07E-09 |
| AGAP2-AS1 | ADAMTS9 | protein_coding | 0.54 | 0.000280902 |
| AGAP2-AS1 | FCHSD1 | protein_coding | 0.60 | 3.04E-05 |
| AGAP2-AS1 | SPINT1 | protein_coding | 0.75 | 1.75E-08 |
| AGAP2-AS1 | TICRR | protein_coding | 0.69 | 7.63E-07 |
| AGAP2-AS1 | TTC26 | protein_coding | 0.54 | 0.000270361 |
| AGAP2-AS1 | WNT9A | protein_coding | 0.55 | 0.000212525 |
| AGAP2-AS1 | PBK | protein_coding | 0.67 | 1.42E-06 |
| AGAP2-AS1 | CA5A | protein_coding | -0.66 | 2.44E-06 |
| AGAP2-AS1 | SPPL2A | protein_coding | -0.56 | 0.000165752 |
| AGAP2-AS1 | RALY | protein_coding | 0.72 | 1.41E-07 |
| AGAP2-AS1 | ADH1B | protein_coding | -0.67 | 1.77E-06 |
| AGAP2-AS1 | PCIF1 | protein_coding | 0.51 | 0.000706506 |
| AGAP2-AS1 | TMEM120B | protein_coding | 0.50 | 0.000849381 |
| AGAP2-AS1 | CD5L | protein_coding | -0.56 | 0.000145487 |
| AGAP2-AS1 | ALDOB | protein_coding | -0.58 | 7.41E-05 |
| AGAP2-AS1 | SCARB1 | protein_coding | -0.50 | 0.000815443 |
| AGAP2-AS1 | GTSE1 | protein_coding | 0.67 | 1.51E-06 |
| AGAP2-AS1 | IGFL4 | protein_coding | 0.53 | 0.000374694 |
| AGAP2-AS1 | FBRS | protein_coding | 0.60 | 4.14E-05 |
| AGAP2-AS1 | ERCC4 | protein_coding | -0.53 | 0.000337843 |
| AGAP2-AS1 | DZIP1L | protein_coding | 0.52 | 0.000471068 |
| AGAP2-AS1 | PES1 | protein_coding | 0.59 | 4.64E-05 |
| AGAP2-AS1 | NXPH1 | protein_coding | -0.50 | 0.000855839 |
| AGAP2-AS1 | NARF | protein_coding | 0.52 | 0.000550732 |
| AGAP2-AS1 | GALP | protein_coding | -0.65 | 3.62E-06 |
| AGAP2-AS1 | ARFGEF3 | protein_coding | 0.59 | 4.95E-05 |
| AGAP2-AS1 | GPR155 | protein_coding | -0.54 | 0.000318249 |
| AGAP2-AS1 | ADCY10 | protein_coding | -0.67 | 2.04E-06 |
| AGAP2-AS1 | ST6GALNAC4 | protein_coding | 0.70 | 4.52E-07 |
| AGAP2-AS1 | CAPN10 | protein_coding | 0.62 | 1.62E-05 |
| AGAP2-AS1 | ACTG1 | protein_coding | 0.60 | 3.72E-05 |
| AGAP2-AS1 | FAH | protein_coding | -0.52 | 0.000484201 |
| AGAP2-AS1 | STK32C | protein_coding | 0.56 | 0.000149271 |
| AGAP2-AS1 | ADCY3 | protein_coding | 0.62 | 1.51E-05 |
| AGAP2-AS1 | MCOLN3 | protein_coding | 0.59 | 5.76E-05 |
| AGAP2-AS1 | GOLGA8M | protein_coding | -0.55 | 0.000232753 |
| AGAP2-AS1 | LAMC2 | protein_coding | 0.62 | 1.41E-05 |
| AGAP2-AS1 | AFMID | protein_coding | -0.65 | 3.96E-06 |
| AGAP2-AS1 | SEC23A | protein_coding | -0.55 | 0.000169441 |
| AGAP2-AS1 | TNFRSF12A | protein_coding | 0.52 | 0.00056637 |
| AGAP2-AS1 | FXYD1 | protein_coding | -0.65 | 4.76E-06 |
| AGAP2-AS1 | TDGF1 | protein_coding | -0.68 | 8.10E-07 |
| AGAP2-AS1 | CKLF-CMTM1 | protein_coding | 0.68 | 8.66E-07 |
| AGAP2-AS1 | PEG3 | protein_coding | -0.59 | 5.87E-05 |
| AGAP2-AS1 | HCFC1 | protein_coding | 0.60 | 2.94E-05 |
| AGAP2-AS1 | ASPA | protein_coding | -0.57 | 9.66E-05 |
| AGAP2-AS1 | NRARP | protein_coding | 0.51 | 0.000607028 |
| AGAP2-AS1 | KLHDC2 | protein_coding | -0.51 | 0.000621932 |
| AGAP2-AS1 | GRN | protein_coding | 0.68 | 8.31E-07 |
| AGAP2-AS1 | PSORS1C2 | protein_coding | 0.61 | 2.32E-05 |
| AGAP2-AS1 | NF2 | protein_coding | 0.63 | 1.09E-05 |
| AGAP2-AS1 | KRT15 | protein_coding | 0.56 | 0.000134668 |
| AGAP2-AS1 | ZCCHC12 | protein_coding | 0.51 | 0.000717485 |
| AGAP2-AS1 | STAT3 | protein_coding | -0.52 | 0.000472232 |
| AGAP2-AS1 | SIAH2 | protein_coding | -0.58 | 7.63E-05 |
| AGAP2-AS1 | NKIRAS1 | protein_coding | -0.62 | 1.90E-05 |
| AGAP2-AS1 | ATP1B3 | protein_coding | 0.70 | 3.23E-07 |
| AGAP2-AS1 | DVL2 | protein_coding | 0.56 | 0.000151127 |
| AGAP2-AS1 | PDXP | protein_coding | -0.54 | 0.000247436 |
| AGAP2-AS1 | CENPH | protein_coding | 0.77 | 5.91E-09 |
| AGAP2-AS1 | APOC4 | protein_coding | -0.68 | 1.00E-06 |
| AGAP2-AS1 | MTMR2 | protein_coding | 0.67 | 1.75E-06 |
| AGAP2-AS1 | RNF121 | protein_coding | 0.55 | 0.000210327 |
| AGAP2-AS1 | MASTL | protein_coding | 0.57 | 0.000103508 |
| AGAP2-AS1 | MST1 | protein_coding | -0.60 | 2.88E-05 |
| AGAP2-AS1 | BUB1 | protein_coding | 0.72 | 1.00E-07 |
| AGAP2-AS1 | IST1 | protein_coding | 0.62 | 1.62E-05 |
| AGAP2-AS1 | CHD3 | protein_coding | 0.54 | 0.000265013 |
| AGAP2-AS1 | MT-ATP8 | protein_coding | -0.54 | 0.000286879 |
| AGAP2-AS1 | ANGPTL8 | protein_coding | -0.54 | 0.000256377 |
| AGAP2-AS1 | USP19 | protein_coding | -0.51 | 0.000712691 |
| AGAP2-AS1 | IFT22 | protein_coding | 0.64 | 6.12E-06 |
| AGAP2-AS1 | PDZD11 | protein_coding | 0.66 | 2.80E-06 |
| AGAP2-AS1 | SEC14L2 | protein_coding | -0.55 | 0.000192576 |
| AGAP2-AS1 | AVPI1 | protein_coding | -0.59 | 5.69E-05 |
| AGAP2-AS1 | RHOC | protein_coding | 0.60 | 3.24E-05 |
| AGAP2-AS1 | APOA5 | protein_coding | -0.53 | 0.000371192 |
| AGAP2-AS1 | PAK4 | protein_coding | 0.54 | 0.000270969 |
| AGAP2-AS1 | KCTD6 | protein_coding | -0.52 | 0.000534108 |
| AGAP2-AS1 | PLA2G5 | protein_coding | -0.52 | 0.000517498 |
| AGAP2-AS1 | MED10 | protein_coding | 0.52 | 0.000505614 |
| AGAP2-AS1 | SPP2 | protein_coding | -0.56 | 0.000121389 |
| AGAP2-AS1 | PRXL2B | protein_coding | 0.54 | 0.000247065 |
| AGAP2-AS1 | COL26A1 | protein_coding | -0.66 | 2.32E-06 |
| AGAP2-AS1 | SYNGR2 | protein_coding | 0.58 | 7.32E-05 |
| AGAP2-AS1 | FERMT2 | protein_coding | -0.57 | 0.000102137 |
| AGAP2-AS1 | CRBN | protein_coding | -0.63 | 9.77E-06 |
| AGAP2-AS1 | CKLF | protein_coding | 0.60 | 4.15E-05 |
| AGAP2-AS1 | USP15 | protein_coding | -0.65 | 4.50E-06 |
| AGAP2-AS1 | INTS9 | protein_coding | 0.52 | 0.000543508 |
| AGAP2-AS1 | ECI2 | protein_coding | -0.54 | 0.00031464 |
| AGAP2-AS1 | ALDOA | protein_coding | 0.71 | 1.71E-07 |
| AGAP2-AS1 | SOX7 | protein_coding | -0.53 | 0.000348351 |
| AGAP2-AS1 | TRAIP | protein_coding | 0.69 | 6.92E-07 |
| AGAP2-AS1 | LRCOL1 | protein_coding | -0.55 | 0.000176368 |
| AGAP2-AS1 | DBNDD2 | protein_coding | 0.71 | 1.97E-07 |
| AGAP2-AS1 | HSPB9 | protein_coding | -0.66 | 2.32E-06 |
| AGAP2-AS1 | ELOA | protein_coding | -0.59 | 4.17E-05 |
| AGAP2-AS1 | MIER2 | protein_coding | 0.69 | 5.80E-07 |
| AGAP2-AS1 | TTC39A | protein_coding | 0.56 | 0.000165631 |
| AGAP2-AS1 | ARPC2 | protein_coding | 0.60 | 2.95E-05 |
| AGAP2-AS1 | HSBP1 | protein_coding | 0.52 | 0.000535936 |
| AGAP2-AS1 | RNF223 | protein_coding | 0.54 | 0.000288049 |
| AGAP2-AS1 | HSPB1 | protein_coding | 0.59 | 4.94E-05 |
| AGAP2-AS1 | LEPR | protein_coding | -0.53 | 0.000374614 |
| AGAP2-AS1 | ZDHHC19 | protein_coding | -0.51 | 0.000665303 |
| AGAP2-AS1 | TF | protein_coding | -0.56 | 0.000155238 |
| AGAP2-AS1 | MTHFD2 | protein_coding | 0.51 | 0.000585631 |
| AGAP2-AS1 | PIK3R4 | protein_coding | -0.59 | 5.48E-05 |
| AGAP2-AS1 | MRPS23 | protein_coding | 0.63 | 9.84E-06 |
| AGAP2-AS1 | NEO1 | protein_coding | 0.61 | 2.00E-05 |
| AGAP2-AS1 | TTLL2 | protein_coding | -0.64 | 6.06E-06 |
| AGAP2-AS1 | RORA | protein_coding | -0.61 | 2.08E-05 |
| AGAP2-AS1 | RPS6KA4 | protein_coding | 0.66 | 2.49E-06 |
| AGAP2-AS1 | HRG | protein_coding | -0.54 | 0.00030009 |
| AGAP2-AS1 | GSDME | protein_coding | 0.53 | 0.000354561 |
| AGAP2-AS1 | SPTAN1 | protein_coding | 0.61 | 2.64E-05 |
| AGAP2-AS1 | PPP1R14B | protein_coding | 0.61 | 2.25E-05 |
| AGAP2-AS1 | KPNB1 | protein_coding | 0.70 | 3.60E-07 |
| AGAP2-AS1 | GNAO1 | protein_coding | -0.54 | 0.000291658 |
| AGAP2-AS1 | TYRP1 | protein_coding | 0.51 | 0.00058758 |
| AGAP2-AS1 | NQO2 | protein_coding | -0.57 | 0.000118739 |
| AGAP2-AS1 | VPS33B | protein_coding | 0.58 | 6.36E-05 |
| AGAP2-AS1 | NMUR1 | protein_coding | -0.54 | 0.000287983 |
| AGAP2-AS1 | CCNA2 | protein_coding | 0.67 | 1.94E-06 |
| AGAP2-AS1 | RPL17 | protein_coding | 0.57 | 8.99E-05 |
| AGAP2-AS1 | CD300LG | protein_coding | -0.57 | 0.00010751 |
| AGAP2-AS1 | C15orf48 | protein_coding | 0.51 | 0.000659433 |
| AGAP2-AS1 | C8orf82 | protein_coding | -0.60 | 3.43E-05 |
| AGAP2-AS1 | GPAM | protein_coding | -0.59 | 5.03E-05 |
| AGAP2-AS1 | NLGN3 | protein_coding | 0.55 | 0.000223319 |
| AGAP2-AS1 | PLEKHB1 | protein_coding | 0.60 | 3.45E-05 |
| AGAP2-AS1 | CYB5A | protein_coding | -0.64 | 7.49E-06 |
| AGAP2-AS1 | IMPDH1 | protein_coding | 0.59 | 5.08E-05 |
| AGAP2-AS1 | TRPM7 | protein_coding | -0.55 | 0.000217019 |
| AGAP2-AS1 | IQGAP1 | protein_coding | 0.55 | 0.000182114 |
| AGAP2-AS1 | CCZ1 | protein_coding | 0.54 | 0.000254718 |
| AGAP2-AS1 | MAPRE1 | protein_coding | 0.63 | 8.48E-06 |
| AGAP2-AS1 | MCF2L2 | protein_coding | 0.52 | 0.000545073 |
| AGAP2-AS1 | ZNF385A | protein_coding | 0.58 | 6.31E-05 |
| AGAP2-AS1 | DHODH | protein_coding | -0.64 | 6.91E-06 |
| AGAP2-AS1 | DLEC1 | protein_coding | -0.53 | 0.00037529 |
| AGAP2-AS1 | SLC25A20 | protein_coding | -0.68 | 1.21E-06 |
| AGAP2-AS1 | TCF3 | protein_coding | 0.65 | 4.26E-06 |
| AGAP2-AS1 | CDA | protein_coding | -0.54 | 0.000306665 |
| AGAP2-AS1 | LONP2 | protein_coding | -0.55 | 0.00018178 |
| AGAP2-AS1 | SLFN13 | protein_coding | 0.62 | 1.74E-05 |
| AGAP2-AS1 | DCXR | protein_coding | -0.61 | 1.98E-05 |
| AGAP2-AS1 | BRMS1 | protein_coding | 0.59 | 5.64E-05 |
| AGAP2-AS1 | TMEM159 | protein_coding | 0.55 | 0.000230757 |
| AGAP2-AS1 | SFXN1 | protein_coding | -0.60 | 3.66E-05 |
| AGAP2-AS1 | DBN1 | protein_coding | 0.67 | 2.07E-06 |
| AGAP2-AS1 | PPDPF | protein_coding | 0.52 | 0.000485518 |
| AGAP2-AS1 | NELFE | protein_coding | 0.57 | 0.000111075 |
| AGAP2-AS1 | INPPL1 | protein_coding | 0.51 | 0.000624753 |
| AGAP2-AS1 | PHLDA2 | protein_coding | 0.54 | 0.000314898 |
| AGAP2-AS1 | ANKS3 | protein_coding | 0.62 | 1.61E-05 |
| AGAP2-AS1 | PRC1 | protein_coding | 0.71 | 1.58E-07 |
| AGAP2-AS1 | STIMATE | protein_coding | -0.62 | 1.74E-05 |
| AGAP2-AS1 | GCH1 | protein_coding | -0.69 | 6.96E-07 |
| AGAP2-AS1 | PLK4 | protein_coding | 0.69 | 5.36E-07 |
| AGAP2-AS1 | B3GNT3 | protein_coding | 0.71 | 2.00E-07 |
| AGAP2-AS1 | CNOT3 | protein_coding | 0.52 | 0.000501381 |
| AGAP2-AS1 | MOGAT1 | protein_coding | -0.61 | 2.67E-05 |
| AGAP2-AS1 | MAN1A1 | protein_coding | -0.74 | 3.49E-08 |
| AGAP2-AS1 | PRDX6 | protein_coding | -0.56 | 0.00015947 |
| AGAP2-AS1 | SOAT2 | protein_coding | -0.50 | 0.000849794 |
| AGAP2-AS1 | PKP2 | protein_coding | -0.60 | 3.04E-05 |
| AGAP2-AS1 | OTUB1 | protein_coding | 0.62 | 1.83E-05 |
| AGAP2-AS1 | CCNE1 | protein_coding | 0.72 | 1.18E-07 |
| AGAP2-AS1 | TTLL1 | protein_coding | 0.67 | 2.14E-06 |
| AGAP2-AS1 | CLIC1 | protein_coding | 0.66 | 2.59E-06 |
| AGAP2-AS1 | PXMP2 | protein_coding | -0.52 | 0.000532264 |
| AGAP2-AS1 | CNKSR2 | protein_coding | -0.51 | 0.000671571 |
| AGAP2-AS1 | ZNF580 | protein_coding | 0.55 | 0.00016999 |
| AGAP2-AS1 | CPNE9 | protein_coding | 0.54 | 0.000311952 |
| AGAP2-AS1 | SLC6A12 | protein_coding | -0.62 | 1.88E-05 |
| AGAP2-AS1 | RASSF7 | protein_coding | 0.55 | 0.000225631 |
| AGAP2-AS1 | MED27 | protein_coding | 0.52 | 0.000554718 |
| AGAP2-AS1 | VOPP1 | protein_coding | 0.60 | 3.23E-05 |
| AGAP2-AS1 | AKR1C4 | protein_coding | -0.57 | 0.000103309 |
| AGAP2-AS1 | C11orf97 | protein_coding | -0.51 | 0.000737997 |
| AGAP2-AS1 | CTXN1 | protein_coding | 0.62 | 1.90E-05 |
| AGAP2-AS1 | DNAJB2 | protein_coding | 0.60 | 2.96E-05 |
| AGAP2-AS1 | OXNAD1 | protein_coding | -0.58 | 6.38E-05 |
| AGAP2-AS1 | CDHR5 | protein_coding | -0.59 | 5.43E-05 |
| AGAP2-AS1 | SEPTIN4 | protein_coding | -0.62 | 1.44E-05 |
| AGAP2-AS1 | DYNC1I2 | protein_coding | 0.52 | 0.000496774 |
| AGAP2-AS1 | UGT3A1 | protein_coding | -0.50 | 0.000783694 |
| AGAP2-AS1 | TBC1D22B | protein_coding | 0.55 | 0.000209767 |
| AGAP2-AS1 | PKM | protein_coding | 0.78 | 1.99E-09 |
| AGAP2-AS1 | PI4K2B | protein_coding | -0.65 | 3.95E-06 |
| AGAP2-AS1 | HMGCS2 | protein_coding | -0.55 | 0.00021414 |
| AGAP2-AS1 | ATP6V1FNB | protein_coding | 0.50 | 0.000846129 |
| AGAP2-AS1 | MCM3 | protein_coding | 0.64 | 8.33E-06 |
| AGAP2-AS1 | SLC25A16 | protein_coding | -0.58 | 7.55E-05 |
| AGAP2-AS1 | RBM3 | protein_coding | 0.61 | 1.94E-05 |
| AGAP2-AS1 | DMPK | protein_coding | 0.67 | 2.03E-06 |
| AGAP2-AS1 | RTN2 | protein_coding | 0.64 | 7.80E-06 |
| AGAP2-AS1 | PA2G4 | protein_coding | 0.50 | 0.000853798 |
| AGAP2-AS1 | CHAF1A | protein_coding | 0.72 | 1.41E-07 |
| AGAP2-AS1 | TP53INP1 | protein_coding | -0.56 | 0.000153641 |
| AGAP2-AS1 | TMEM17 | protein_coding | 0.56 | 0.000147932 |
| AGAP2-AS1 | CYP1A1 | protein_coding | -0.58 | 8.02E-05 |
| AGAP2-AS1 | PGAP4 | protein_coding | 0.58 | 8.12E-05 |
| AGAP2-AS1 | CEBPA | protein_coding | -0.62 | 1.81E-05 |
| AGAP2-AS1 | C12orf76 | protein_coding | 0.50 | 0.000779278 |
| AGAP2-AS1 | ZSWIM9 | protein_coding | 0.58 | 6.70E-05 |
| AGAP2-AS1 | CDC45 | protein_coding | 0.75 | 1.86E-08 |
| AGAP2-AS1 | F10 | protein_coding | -0.51 | 0.000619343 |
| AGAP2-AS1 | AURKA | protein_coding | 0.63 | 1.09E-05 |
| AGAP2-AS1 | TSPO | protein_coding | 0.67 | 1.46E-06 |
| AGAP2-AS1 | YEATS2 | protein_coding | 0.66 | 3.37E-06 |
| AGAP2-AS1 | ALDH7A1 | protein_coding | -0.59 | 5.13E-05 |
| AGAP2-AS1 | SLC35F3 | protein_coding | 0.66 | 2.65E-06 |
| AGAP2-AS1 | ANAPC7 | protein_coding | 0.52 | 0.000465922 |
| AGAP2-AS1 | DNMT3A | protein_coding | 0.54 | 0.000242899 |
| AGAP2-AS1 | VPS39 | protein_coding | 0.53 | 0.00034803 |
| AGAP2-AS1 | SAR1B | protein_coding | -0.63 | 1.19E-05 |
| AGAP2-AS1 | WDR73 | protein_coding | 0.56 | 0.000166527 |
| AGAP2-AS1 | MT1H | protein_coding | -0.58 | 6.23E-05 |
| AGAP2-AS1 | MLLT3 | protein_coding | 0.55 | 0.000214586 |
| AGAP2-AS1 | C5orf34 | protein_coding | 0.67 | 1.80E-06 |
| AGAP2-AS1 | SERPINF2 | protein_coding | -0.52 | 0.000480511 |
| AGAP2-AS1 | HAGHL | protein_coding | 0.66 | 2.38E-06 |
| AGAP2-AS1 | IDI1 | protein_coding | -0.66 | 3.45E-06 |
| AGAP2-AS1 | ABCC10 | protein_coding | 0.63 | 9.11E-06 |
| AGAP2-AS1 | LRRC23 | protein_coding | 0.61 | 2.73E-05 |
| AGAP2-AS1 | CYP2R1 | protein_coding | 0.57 | 0.0001106 |
| AGAP2-AS1 | KLF15 | protein_coding | -0.57 | 9.10E-05 |
| AGAP2-AS1 | N4BP3 | protein_coding | 0.54 | 0.000289703 |
| AGAP2-AS1 | RHCE | protein_coding | -0.53 | 0.000425794 |
| AGAP2-AS1 | LTBP3 | protein_coding | 0.53 | 0.000349866 |
| AGAP2-AS1 | ZNF365 | protein_coding | 0.56 | 0.00014881 |
| AGAP2-AS1 | VEPH1 | protein_coding | 0.54 | 0.000273218 |
| AGAP2-AS1 | RBMXL1 | protein_coding | -0.52 | 0.000556636 |
| AGAP2-AS1 | FOXM1 | protein_coding | 0.70 | 3.87E-07 |
| AGAP2-AS1 | KIF3A | protein_coding | 0.51 | 0.000593652 |
| AGAP2-AS1 | SSBP2 | protein_coding | 0.59 | 4.73E-05 |
| AGAP2-AS1 | PLPP2 | protein_coding | 0.70 | 3.25E-07 |
| AGAP2-AS1 | ADH1A | protein_coding | -0.57 | 9.47E-05 |
| AGAP2-AS1 | PROC | protein_coding | -0.58 | 7.06E-05 |
| AGAP2-AS1 | PKD1L3 | protein_coding | -0.53 | 0.000331766 |
| AGAP2-AS1 | CCDC137 | protein_coding | 0.71 | 2.14E-07 |
| AGAP2-AS1 | TRIP4 | protein_coding | 0.61 | 2.60E-05 |
| AGAP2-AS1 | GASK1A | protein_coding | -0.69 | 7.55E-07 |
| AGAP2-AS1 | KLHL29 | protein_coding | 0.57 | 0.00011945 |
| AGAP2-AS1 | FZD10 | protein_coding | 0.54 | 0.000237558 |
| AGAP2-AS1 | UTP20 | protein_coding | 0.53 | 0.000401635 |
| AGAP2-AS1 | CPEB3 | protein_coding | -0.63 | 1.06E-05 |
| AGAP2-AS1 | ZBED5 | protein_coding | 0.61 | 2.58E-05 |
| AGAP2-AS1 | SLC22A9 | protein_coding | -0.56 | 0.000126131 |
| AGAP2-AS1 | PLIN4 | protein_coding | -0.67 | 1.86E-06 |
| AGAP2-AS1 | ZDHHC24 | protein_coding | 0.73 | 8.37E-08 |
| AGAP2-AS1 | AMH | protein_coding | 0.51 | 0.000599209 |
| AGAP2-AS1 | PPID | protein_coding | -0.56 | 0.000153223 |
| AGAP2-AS1 | A1BG | protein_coding | -0.57 | 9.95E-05 |
| AGAP2-AS1 | GNAZ | protein_coding | 0.57 | 9.49E-05 |
| AGAP2-AS1 | CES1 | protein_coding | -0.59 | 5.30E-05 |
| AGAP2-AS1 | UPP2 | protein_coding | -0.56 | 0.000160465 |
| AGAP2-AS1 | CDK1 | protein_coding | 0.73 | 4.81E-08 |
| AGAP2-AS1 | TENM1 | protein_coding | -0.70 | 3.55E-07 |
| AGAP2-AS1 | INHBC | protein_coding | -0.64 | 8.00E-06 |
| AGAP2-AS1 | CCDC127 | protein_coding | 0.50 | 0.000859982 |
| AGAP2-AS1 | ALDH1L1 | protein_coding | -0.59 | 5.63E-05 |
| AGAP2-AS1 | MEP1B | protein_coding | -0.67 | 1.99E-06 |
| AGAP2-AS1 | PLIN2 | protein_coding | -0.61 | 2.16E-05 |
| AGAP2-AS1 | GRIA3 | protein_coding | -0.58 | 8.30E-05 |
| AGAP2-AS1 | ADGRB2 | protein_coding | 0.78 | 1.94E-09 |
| AGAP2-AS1 | PDGFA | protein_coding | 0.52 | 0.000446067 |
| AGAP2-AS1 | PRR15L | protein_coding | 0.55 | 0.000229692 |
| AGAP2-AS1 | ZNF563 | protein_coding | -0.51 | 0.000647078 |
| AGAP2-AS1 | COLEC10 | protein_coding | -0.53 | 0.000372805 |
| AGAP2-AS1 | EXTL3 | protein_coding | 0.56 | 0.000136675 |
| AGAP2-AS1 | PDXDC1 | protein_coding | -0.54 | 0.000272808 |
| AGAP2-AS1 | CALML3 | protein_coding | -0.64 | 7.25E-06 |
| AGAP2-AS1 | PARP14 | protein_coding | -0.50 | 0.000838555 |
| AGAP2-AS1 | INHA | protein_coding | 0.52 | 0.000573669 |
| AGAP2-AS1 | ATP6AP1 | protein_coding | 0.58 | 7.59E-05 |
| AGAP2-AS1 | MACC1 | protein_coding | 0.52 | 0.000542371 |
| AGAP2-AS1 | TAB2 | protein_coding | -0.52 | 0.000452819 |
| AGAP2-AS1 | HSD17B8 | protein_coding | -0.62 | 1.57E-05 |
| AGAP2-AS1 | SNPH | protein_coding | 0.58 | 6.85E-05 |
| AGAP2-AS1 | RNF44 | protein_coding | 0.52 | 0.000466005 |
| AGAP2-AS1 | ETAA1 | protein_coding | 0.67 | 1.50E-06 |
| AGAP2-AS1 | PCBD1 | protein_coding | -0.54 | 0.000268056 |
| AGAP2-AS1 | NPTX1 | protein_coding | 0.58 | 6.92E-05 |
| AGAP2-AS1 | SLC2A14 | protein_coding | -0.53 | 0.000401096 |
| AGAP2-AS1 | MOCS1 | protein_coding | -0.55 | 0.000176752 |
| AGAP2-AS1 | MYRIP | protein_coding | -0.66 | 2.21E-06 |
| AGAP2-AS1 | SLC27A5 | protein_coding | -0.63 | 8.78E-06 |
| AGAP2-AS1 | AL583836.1 | protein_coding | -0.65 | 5.10E-06 |
| AGAP2-AS1 | ATPAF1 | protein_coding | -0.52 | 0.00044367 |
| AGAP2-AS1 | KLB | protein_coding | -0.52 | 0.000535639 |
| AGAP2-AS1 | EPB41L5 | protein_coding | -0.55 | 0.000185074 |
| AGAP2-AS1 | CHN2 | protein_coding | -0.59 | 5.64E-05 |
| AGAP2-AS1 | HAAO | protein_coding | -0.51 | 0.000603678 |
| AGAP2-AS1 | AFF4 | protein_coding | -0.52 | 0.000545147 |
| AGAP2-AS1 | MROH2A | protein_coding | -0.58 | 8.56E-05 |
| AGAP2-AS1 | CPSF6 | protein_coding | 0.51 | 0.000655706 |
| AGAP2-AS1 | TEAD4 | protein_coding | 0.71 | 2.38E-07 |
| AGAP2-AS1 | NLGN4X | protein_coding | -0.50 | 0.000797269 |
| AGAP2-AS1 | C1QL4 | protein_coding | 0.55 | 0.000217448 |
| AGAP2-AS1 | MOGAT2 | protein_coding | -0.58 | 6.45E-05 |
| AGAP2-AS1 | NMRK1 | protein_coding | -0.53 | 0.00036499 |
| AGAP2-AS1 | ZFAND4 | protein_coding | -0.54 | 0.000302251 |
| AGAP2-AS1 | TSEN54 | protein_coding | 0.67 | 1.70E-06 |
| AGAP2-AS1 | STK11IP | protein_coding | 0.68 | 1.23E-06 |
| AGAP2-AS1 | RFC4 | protein_coding | 0.65 | 3.57E-06 |
| AGAP2-AS1 | MRPL32 | protein_coding | -0.53 | 0.000336047 |
| AGAP2-AS1 | AL136295.3 | protein_coding | -0.62 | 1.71E-05 |
| AGAP2-AS1 | PDE2A | protein_coding | -0.51 | 0.000653019 |
| AGAP2-AS1 | GCGR | protein_coding | -0.56 | 0.000134293 |
| AGAP2-AS1 | B9D2 | protein_coding | 0.56 | 0.000135777 |
| AGAP2-AS1 | PPP1R35 | protein_coding | 0.52 | 0.000460296 |
| AGAP2-AS1 | GLB1L2 | protein_coding | 0.60 | 3.35E-05 |
| AGAP2-AS1 | NR1I2 | protein_coding | -0.62 | 1.66E-05 |
| AGAP2-AS1 | BCHE | protein_coding | -0.55 | 0.000228294 |
| AGAP2-AS1 | LENG9 | protein_coding | 0.53 | 0.000392436 |
| AGAP2-AS1 | POF1B | protein_coding | 0.55 | 0.000223915 |
| AGAP2-AS1 | OIP5 | protein_coding | 0.66 | 2.75E-06 |
| AGAP2-AS1 | ACOT6 | protein_coding | -0.64 | 7.10E-06 |
| AGAP2-AS1 | PPARA | protein_coding | -0.73 | 4.65E-08 |
| AGAP2-AS1 | FSCN2 | protein_coding | 0.69 | 6.34E-07 |
| AGAP2-AS1 | HSD17B4 | protein_coding | -0.66 | 2.20E-06 |
| AGAP2-AS1 | AC022335.1 | protein_coding | -0.67 | 1.37E-06 |
| AGAP2-AS1 | FEN1 | protein_coding | 0.66 | 2.69E-06 |
| AGAP2-AS1 | ELF4 | protein_coding | 0.69 | 5.72E-07 |
| AGAP2-AS1 | AKAIN1 | protein_coding | -0.58 | 7.14E-05 |
| AGAP2-AS1 | EME1 | protein_coding | 0.74 | 2.93E-08 |
| AGAP2-AS1 | CBFA2T3 | protein_coding | -0.56 | 0.000142026 |
| AGAP2-AS1 | KIF19 | protein_coding | -0.54 | 0.000252557 |
| AGAP2-AS1 | WDR76 | protein_coding | 0.63 | 1.19E-05 |
| AGAP2-AS1 | DICER1 | protein_coding | -0.58 | 6.33E-05 |
| AGAP2-AS1 | WDR45B | protein_coding | 0.52 | 0.000520052 |
| AGAP2-AS1 | ZNF581 | protein_coding | 0.56 | 0.000136288 |
| AGAP2-AS1 | CRLS1 | protein_coding | -0.55 | 0.00021571 |
| AGAP2-AS1 | CES3 | protein_coding | -0.52 | 0.000532844 |
| AGAP2-AS1 | HASPIN | protein_coding | 0.55 | 0.000205262 |
| AGAP2-AS1 | DDR1 | protein_coding | 0.66 | 2.91E-06 |
| AGAP2-AS1 | DCAF16 | protein_coding | 0.52 | 0.000473898 |
| AGAP2-AS1 | CPNE7 | protein_coding | 0.62 | 1.83E-05 |
| AGAP2-AS1 | CABIN1 | protein_coding | 0.54 | 0.000312699 |
| AGAP2-AS1 | L3MBTL1 | protein_coding | 0.54 | 0.000290751 |
| AGAP2-AS1 | NOTUM | protein_coding | -0.51 | 0.000681996 |
| AGAP2-AS1 | DHX57 | protein_coding | 0.60 | 2.86E-05 |
| AGAP2-AS1 | MT-ND6 | protein_coding | -0.61 | 2.72E-05 |
| AGAP2-AS1 | GAD1 | protein_coding | 0.53 | 0.00039037 |
| AGAP2-AS1 | IDUA | protein_coding | 0.57 | 8.88E-05 |
| AGAP2-AS1 | DPPA4 | protein_coding | -0.57 | 9.41E-05 |
| AGAP2-AS1 | ADAMTS17 | protein_coding | -0.65 | 3.64E-06 |
| AGAP2-AS1 | ARHGEF25 | protein_coding | 0.67 | 1.54E-06 |
| AGAP2-AS1 | ETFBKMT | protein_coding | -0.62 | 1.39E-05 |
| AGAP2-AS1 | LIG4 | protein_coding | -0.51 | 0.000583925 |
| AGAP2-AS1 | GRID2IP | protein_coding | 0.64 | 6.69E-06 |
| AGAP2-AS1 | OSCP1 | protein_coding | 0.53 | 0.000404328 |
| AGAP2-AS1 | CD2BP2 | protein_coding | 0.68 | 8.68E-07 |
| AGAP2-AS1 | PRIMA1 | protein_coding | -0.50 | 0.000818583 |
| AGAP2-AS1 | AC092042.3 | protein_coding | -0.62 | 1.60E-05 |
| AGAP2-AS1 | RSPH1 | protein_coding | 0.51 | 0.000673125 |
| AGAP2-AS1 | MCM4 | protein_coding | 0.68 | 1.21E-06 |
| AGAP2-AS1 | SOX5 | protein_coding | -0.71 | 1.83E-07 |
| AGAP2-AS1 | GATM | protein_coding | -0.62 | 1.36E-05 |
| AGAP2-AS1 | ACYP1 | protein_coding | 0.60 | 3.04E-05 |
| AGAP2-AS1 | MTO1 | protein_coding | -0.55 | 0.000211587 |
| AGAP2-AS1 | PRDM4 | protein_coding | 0.57 | 0.000111472 |
| AGAP2-AS1 | DSN1 | protein_coding | 0.65 | 4.95E-06 |
| AGAP2-AS1 | DHX37 | protein_coding | 0.61 | 2.19E-05 |
| AGAP2-AS1 | HAO1 | protein_coding | -0.58 | 6.69E-05 |
| AGAP2-AS1 | CES5A | protein_coding | -0.68 | 8.71E-07 |
| AGAP2-AS1 | DEPDC7 | protein_coding | -0.56 | 0.000131785 |
| AGAP2-AS1 | ANXA6 | protein_coding | -0.56 | 0.00014092 |
| AGAP2-AS1 | RAB36 | protein_coding | 0.60 | 3.84E-05 |
| AGAP2-AS1 | TMEM101 | protein_coding | 0.69 | 7.22E-07 |
| AGAP2-AS1 | AKR1C8P | protein_coding | -0.57 | 9.72E-05 |
| AGAP2-AS1 | GHRHR | protein_coding | 0.57 | 9.16E-05 |
| AGAP2-AS1 | SLC30A1 | protein_coding | -0.68 | 9.91E-07 |
| AGAP2-AS1 | MTTP | protein_coding | -0.60 | 3.99E-05 |
| AGAP2-AS1 | RUSC1 | protein_coding | 0.53 | 0.000389876 |
| AGAP2-AS1 | NSMAF | protein_coding | 0.50 | 0.00083535 |
| AGAP2-AS1 | TBC1D22A | protein_coding | 0.59 | 4.81E-05 |
| AGAP2-AS1 | SRD5A2 | protein_coding | -0.65 | 5.12E-06 |
| AGAP2-AS1 | CENPA | protein_coding | 0.72 | 1.33E-07 |
| AGAP2-AS1 | MAP1A | protein_coding | 0.55 | 0.000230809 |
| AGAP2-AS1 | C4orf46 | protein_coding | 0.64 | 6.24E-06 |
| AGAP2-AS1 | COPS8 | protein_coding | 0.52 | 0.000486402 |
| AGAP2-AS1 | FANCA | protein_coding | 0.55 | 0.000194954 |
| AGAP2-AS1 | MOB1A | protein_coding | 0.50 | 0.000834901 |
| AGAP2-AS1 | GAS2L1 | protein_coding | 0.50 | 0.00079197 |
| AGAP2-AS1 | XRN1 | protein_coding | -0.57 | 0.000119606 |
| AGAP2-AS1 | ITLN1 | protein_coding | -0.60 | 2.87E-05 |
| AGAP2-AS1 | ATP6V1F | protein_coding | 0.67 | 2.03E-06 |
| AGAP2-AS1 | KBTBD11 | protein_coding | -0.59 | 5.72E-05 |
| AGAP2-AS1 | APOC1 | protein_coding | -0.60 | 2.93E-05 |
| AGAP2-AS1 | JHY | protein_coding | 0.54 | 0.000313857 |
| AGAP2-AS1 | NUMBL | protein_coding | 0.52 | 0.000484484 |
| AGAP2-AS1 | TMEM107 | protein_coding | 0.59 | 5.79E-05 |
| AGAP2-AS1 | POLR3GL | protein_coding | -0.51 | 0.000648425 |
| AGAP2-AS1 | FGF21 | protein_coding | -0.61 | 2.45E-05 |
| AGAP2-AS1 | MMAA | protein_coding | -0.71 | 1.88E-07 |
| AGAP2-AS1 | BRCA2 | protein_coding | 0.57 | 0.000101397 |
| AGAP2-AS1 | HECTD1 | protein_coding | -0.57 | 0.000116978 |
| AGAP2-AS1 | G6PC | protein_coding | -0.53 | 0.00041117 |
| AGAP2-AS1 | MCM10 | protein_coding | 0.66 | 2.28E-06 |
| AGAP2-AS1 | SKA2 | protein_coding | 0.58 | 7.29E-05 |
| AGAP2-AS1 | AP5Z1 | protein_coding | 0.54 | 0.000253659 |
| AGAP2-AS1 | TSPAN19 | protein_coding | 0.56 | 0.000125169 |
| AGAP2-AS1 | CHEK1 | protein_coding | 0.70 | 2.78E-07 |
| AGAP2-AS1 | SEC11A | protein_coding | 0.52 | 0.000432671 |
| AGAP2-AS1 | SCN11A | protein_coding | -0.57 | 0.000117361 |
| AGAP2-AS1 | LDLRAD4 | protein_coding | -0.64 | 7.01E-06 |
| AGAP2-AS1 | PNMA6A | protein_coding | -0.56 | 0.000157355 |
| AGAP2-AS1 | BARD1 | protein_coding | 0.68 | 1.27E-06 |
| AGAP2-AS1 | ABCA5 | protein_coding | -0.61 | 2.37E-05 |
| AGAP2-AS1 | CYP2C9 | protein_coding | -0.65 | 4.05E-06 |
| AGAP2-AS1 | CLEC4M | protein_coding | -0.62 | 1.55E-05 |
| AGAP2-AS1 | PIP5K1C | protein_coding | 0.72 | 1.13E-07 |
| AGAP2-AS1 | SEC61A2 | protein_coding | -0.52 | 0.000468555 |
| AGAP2-AS1 | ITIH5 | protein_coding | 0.54 | 0.000295119 |
| AGAP2-AS1 | SETD1A | protein_coding | 0.54 | 0.000263023 |
| AGAP2-AS1 | SLC9A1 | protein_coding | 0.56 | 0.000157448 |
| AGAP2-AS1 | DCTPP1 | protein_coding | 0.62 | 1.42E-05 |
| AGAP2-AS1 | G6PC3 | protein_coding | 0.73 | 6.90E-08 |
| AGAP2-AS1 | C2orf15 | protein_coding | 0.66 | 2.98E-06 |
| AGAP2-AS1 | ACOX3 | protein_coding | -0.51 | 0.000697953 |
| AGAP2-AS1 | MT-ND4 | protein_coding | -0.59 | 4.89E-05 |
| AGAP2-AS1 | AGXT2 | protein_coding | -0.53 | 0.000367693 |
| AGAP2-AS1 | TNNT1 | protein_coding | 0.62 | 1.80E-05 |
| AGAP2-AS1 | SLC22A1 | protein_coding | -0.60 | 3.39E-05 |
| AGAP2-AS1 | LRP10 | protein_coding | 0.61 | 2.53E-05 |
| AGAP2-AS1 | TAZ | protein_coding | 0.52 | 0.000527713 |
| AGAP2-AS1 | UBE2S | protein_coding | 0.73 | 8.29E-08 |
| AGAP2-AS1 | PRR19 | protein_coding | 0.57 | 9.32E-05 |
| AGAP2-AS1 | ZMYND12 | protein_coding | -0.58 | 8.25E-05 |
| AGAP2-AS1 | TENT5C | protein_coding | -0.54 | 0.000308928 |
| AGAP2-AS1 | DCT | protein_coding | -0.50 | 0.000812352 |
| AGAP2-AS1 | FOXN4 | protein_coding | -0.60 | 3.36E-05 |
| AGAP2-AS1 | B4GALNT4 | protein_coding | 0.58 | 8.53E-05 |
| AGAP2-AS1 | UFL1 | protein_coding | -0.54 | 0.000316039 |
| AGAP2-AS1 | IFT81 | protein_coding | 0.58 | 7.59E-05 |
| AGAP2-AS1 | NRF1 | protein_coding | 0.53 | 0.000346034 |
| AGAP2-AS1 | SLC25A13 | protein_coding | -0.65 | 5.07E-06 |
| AGAP2-AS1 | PCK1 | protein_coding | -0.51 | 0.000645236 |
| AGAP2-AS1 | CCL15-CCL14 | protein_coding | -0.63 | 9.47E-06 |
| AGAP2-AS1 | PCNX2 | protein_coding | 0.69 | 5.74E-07 |
| AGAP2-AS1 | CENPF | protein_coding | 0.64 | 6.35E-06 |
| AGAP2-AS1 | CNTFR | protein_coding | -0.64 | 5.87E-06 |
| AGAP2-AS1 | SYVN1 | protein_coding | -0.55 | 0.000210143 |
| AGAP2-AS1 | CEP41 | protein_coding | 0.62 | 1.65E-05 |
| AGAP2-AS1 | RECQL5 | protein_coding | 0.50 | 0.000844798 |
| AGAP2-AS1 | GK | protein_coding | -0.52 | 0.000566824 |
| AGAP2-AS1 | SHISA2 | protein_coding | 0.53 | 0.000406198 |
| AGAP2-AS1 | RNASEH2A | protein_coding | 0.67 | 1.88E-06 |
| AGAP2-AS1 | NPEPL1 | protein_coding | 0.55 | 0.000189698 |
| AGAP2-AS1 | ENO4 | protein_coding | 0.52 | 0.000440124 |
| AGAP2-AS1 | AAGAB | protein_coding | 0.67 | 1.78E-06 |
| AGAP2-AS1 | NCAPG | protein_coding | 0.71 | 2.36E-07 |
| AGAP2-AS1 | FBXL19 | protein_coding | 0.68 | 1.03E-06 |
| AGAP2-AS1 | RBMS2 | protein_coding | 0.61 | 1.95E-05 |
| AGAP2-AS1 | CHRNA5 | protein_coding | 0.65 | 4.84E-06 |
| AGAP2-AS1 | TMOD2 | protein_coding | 0.52 | 0.000535459 |
| AGAP2-AS1 | HBD | protein_coding | -0.54 | 0.000253707 |
| AGAP2-AS1 | CEACAM1 | protein_coding | -0.55 | 0.000176168 |
| AGAP2-AS1 | FAM9B | protein_coding | -0.51 | 0.000671189 |
| AGAP2-AS1 | UCN2 | protein_coding | 0.57 | 9.53E-05 |
| AGAP2-AS1 | CKAP2L | protein_coding | 0.62 | 1.72E-05 |
| AGAP2-AS1 | SIK2 | protein_coding | -0.58 | 7.26E-05 |
| AGAP2-AS1 | SMARCE1 | protein_coding | 0.57 | 8.67E-05 |
| AGAP2-AS1 | SFXN2 | protein_coding | -0.65 | 3.68E-06 |
| AGAP2-AS1 | MRPL53 | protein_coding | 0.52 | 0.000464389 |
| AGAP2-AS1 | 43891 | protein_coding | -0.72 | 1.40E-07 |
| AGAP2-AS1 | BAIAP2L2 | protein_coding | 0.66 | 2.17E-06 |
| AGAP2-AS1 | RNF123 | protein_coding | -0.58 | 7.76E-05 |
| AGAP2-AS1 | CYP8B1 | protein_coding | -0.56 | 0.000124553 |
| AGAP2-AS1 | SLC35A3 | protein_coding | -0.53 | 0.000344873 |
| AGAP2-AS1 | RNF227 | protein_coding | 0.53 | 0.0003724 |
| AGAP2-AS1 | FOXN3 | protein_coding | -0.73 | 8.24E-08 |
| AGAP2-AS1 | RABL2B | protein_coding | 0.57 | 9.81E-05 |
| AGAP2-AS1 | HMGN5 | protein_coding | -0.51 | 0.000728121 |
| AGAP2-AS1 | CENPI | protein_coding | 0.65 | 3.91E-06 |
| AGAP2-AS1 | TCAIM | protein_coding | -0.74 | 3.65E-08 |
| AGAP2-AS1 | LHPP | protein_coding | -0.58 | 8.51E-05 |
| AGAP2-AS1 | EIF4A3 | protein_coding | 0.71 | 1.71E-07 |
| AGAP2-AS1 | SEC24A | protein_coding | -0.60 | 3.32E-05 |
| AGAP2-AS1 | ASIC3 | protein_coding | 0.50 | 0.000836465 |
| AGAP2-AS1 | FOXS1 | protein_coding | 0.52 | 0.000498489 |
| AGAP2-AS1 | PALB2 | protein_coding | 0.55 | 0.000196188 |
| AGAP2-AS1 | OIT3 | protein_coding | -0.56 | 0.000142918 |
| AGAP2-AS1 | PNKP | protein_coding | 0.51 | 0.000627349 |
| AGAP2-AS1 | MMS19 | protein_coding | 0.51 | 0.000651828 |
| AGAP2-AS1 | CCDC152 | protein_coding | -0.65 | 4.13E-06 |
| AGAP2-AS1 | CRMP1 | protein_coding | 0.53 | 0.000341809 |
| AGAP2-AS1 | SF3A2 | protein_coding | 0.54 | 0.000315076 |
| AGAP2-AS1 | C2orf50 | protein_coding | 0.55 | 0.00022158 |
| AGAP2-AS1 | UBASH3B | protein_coding | 0.58 | 7.25E-05 |
| AGAP2-AS1 | KCND3 | protein_coding | -0.57 | 8.86E-05 |
| AGAP2-AS1 | C7orf31 | protein_coding | 0.54 | 0.000261192 |
| AGAP2-AS1 | HSD17B10 | protein_coding | -0.51 | 0.000684184 |
| AGAP2-AS1 | CYP4Z1 | protein_coding | -0.52 | 0.000562522 |
| AGAP2-AS1 | SAE1 | protein_coding | 0.61 | 2.40E-05 |
| AGAP2-AS1 | SLC25A25 | protein_coding | -0.54 | 0.000278704 |
| AGAP2-AS1 | LPCAT4 | protein_coding | 0.71 | 2.13E-07 |
| AGAP2-AS1 | LNP1 | protein_coding | -0.57 | 9.43E-05 |
| AGAP2-AS1 | AL121845.2 | protein_coding | -0.72 | 9.83E-08 |
| AGAP2-AS1 | CYP26A1 | protein_coding | -0.60 | 3.67E-05 |
| AGAP2-AS1 | CBS | protein_coding | -0.54 | 0.000270026 |
| AGAP2-AS1 | ABCB4 | protein_coding | -0.58 | 7.08E-05 |
| AGAP2-AS1 | DNASE1L3 | protein_coding | -0.58 | 8.07E-05 |
| AGAP2-AS1 | NUS1 | protein_coding | -0.58 | 7.90E-05 |
| AGAP2-AS1 | LYPD6B | protein_coding | 0.54 | 0.00027979 |
| AGAP2-AS1 | CDCA5 | protein_coding | 0.78 | 2.46E-09 |
| AGAP2-AS1 | GLS | protein_coding | 0.58 | 6.74E-05 |
| AGAP2-AS1 | TTLL13P | protein_coding | 0.59 | 4.47E-05 |
| AGAP2-AS1 | ACSS2 | protein_coding | -0.53 | 0.000363125 |
| AGAP2-AS1 | SPACA7 | protein_coding | -0.72 | 1.15E-07 |
| AGAP2-AS1 | NACC1 | protein_coding | 0.56 | 0.000127885 |
| AGAP2-AS1 | GRP | protein_coding | 0.55 | 0.000225597 |
| AGAP2-AS1 | IDO2 | protein_coding | -0.65 | 4.62E-06 |
| AGAP2-AS1 | SHE | protein_coding | -0.61 | 2.07E-05 |
| AGAP2-AS1 | DNAJC25 | protein_coding | -0.66 | 3.08E-06 |
| AGAP2-AS1 | RELL2 | protein_coding | 0.65 | 5.04E-06 |
| AGAP2-AS1 | CHAF1B | protein_coding | 0.74 | 4.39E-08 |
| AGAP2-AS1 | PCYOX1L | protein_coding | 0.69 | 5.20E-07 |
| AGAP2-AS1 | RHBDF1 | protein_coding | 0.59 | 5.45E-05 |
| AGAP2-AS1 | GFOD2 | protein_coding | -0.51 | 0.000719423 |
| AGAP2-AS1 | IVD | protein_coding | -0.62 | 1.47E-05 |
| AGAP2-AS1 | NAT2 | protein_coding | -0.54 | 0.000248245 |
| AGAP2-AS1 | PTTG1 | protein_coding | 0.70 | 3.84E-07 |
| AGAP2-AS1 | ZBED1 | protein_coding | -0.52 | 0.000521561 |
| AGAP2-AS1 | TMPRSS9 | protein_coding | -0.51 | 0.000672674 |
| AGAP2-AS1 | ATF7IP2 | protein_coding | -0.64 | 6.86E-06 |
| AGAP2-AS1 | SPC24 | protein_coding | 0.67 | 1.69E-06 |
| AGAP2-AS1 | ZCCHC24 | protein_coding | -0.60 | 3.86E-05 |
| AGAP2-AS1 | MFSD6 | protein_coding | 0.72 | 9.88E-08 |
| AGAP2-AS1 | NADK2 | protein_coding | -0.65 | 4.15E-06 |
| AGAP2-AS1 | THRSP | protein_coding | -0.54 | 0.000290432 |
| AGAP2-AS1 | HES2 | protein_coding | 0.52 | 0.000438633 |
| AGAP2-AS1 | PDPN | protein_coding | 0.52 | 0.000555564 |
| AGAP2-AS1 | PSMC3IP | protein_coding | 0.79 | 1.29E-09 |
| AGAP2-AS1 | DAO | protein_coding | -0.55 | 0.000209341 |
| AGAP2-AS1 | TTPA | protein_coding | -0.60 | 4.06E-05 |
| AGAP2-AS1 | FCGRT | protein_coding | -0.52 | 0.000534006 |
| AGAP2-AS1 | TTC39C | protein_coding | -0.59 | 4.90E-05 |
| AGAP2-AS1 | PPP1R15B | protein_coding | -0.62 | 1.75E-05 |
| AGAP2-AS1 | LAD1 | protein_coding | 0.56 | 0.000137782 |
| AGAP2-AS1 | KANSL1 | protein_coding | 0.54 | 0.000288551 |
| AGAP2-AS1 | ARL4C | protein_coding | 0.57 | 0.000108656 |
| AGAP2-AS1 | TSPYL1 | protein_coding | -0.59 | 4.31E-05 |
| AGAP2-AS1 | UBXN10 | protein_coding | -0.61 | 2.83E-05 |
| AGAP2-AS1 | BRIX1 | protein_coding | 0.56 | 0.000126255 |
| AGAP2-AS1 | NTF3 | protein_coding | -0.63 | 1.09E-05 |
| AGAP2-AS1 | NCS1 | protein_coding | 0.70 | 3.27E-07 |
| AGAP2-AS1 | MARCHF10 | protein_coding | 0.58 | 6.96E-05 |
| AGAP2-AS1 | RHNO1 | protein_coding | 0.71 | 1.62E-07 |
| AGAP2-AS1 | TMEM131L | protein_coding | -0.66 | 2.83E-06 |
| AGAP2-AS1 | SLC25A19 | protein_coding | 0.62 | 1.85E-05 |
| AGAP2-AS1 | C11orf49 | protein_coding | 0.74 | 3.78E-08 |
| AGAP2-AS1 | MICALL1 | protein_coding | 0.51 | 0.000682679 |
| AGAP2-AS1 | RCE1 | protein_coding | 0.68 | 9.80E-07 |
| AGAP2-AS1 | PSME3 | protein_coding | 0.58 | 8.10E-05 |
| AGAP2-AS1 | TMEM267 | protein_coding | 0.51 | 0.000625629 |
| AGAP2-AS1 | E2F8 | protein_coding | 0.70 | 3.64E-07 |
| AGAP2-AS1 | NOL12 | protein_coding | 0.52 | 0.000535207 |
| AGAP2-AS1 | IFT172 | protein_coding | 0.52 | 0.00048402 |
| AGAP2-AS1 | CPED1 | protein_coding | -0.61 | 2.44E-05 |
| AGAP2-AS1 | NANOS1 | protein_coding | 0.53 | 0.000319622 |
| AGAP2-AS1 | TMEM178B | protein_coding | 0.50 | 0.000785193 |
| AGAP2-AS1 | SPATC1L | protein_coding | 0.64 | 5.97E-06 |
| AGAP2-AS1 | SHBG | protein_coding | -0.51 | 0.000665569 |
| AGAP2-AS1 | NLRP14 | protein_coding | -0.54 | 0.00031408 |
| AGAP2-AS1 | PALMD | protein_coding | -0.57 | 0.000105662 |
| AGAP2-AS1 | PER3 | protein_coding | -0.61 | 2.76E-05 |
| AGAP2-AS1 | AC011498.4 | protein_coding | -0.57 | 0.000105154 |
| AGAP2-AS1 | MME | protein_coding | -0.54 | 0.000266299 |
| AGAP2-AS1 | SNX4 | protein_coding | -0.62 | 1.36E-05 |
| AGAP2-AS1 | SOX4 | protein_coding | 0.58 | 8.19E-05 |
| AGAP2-AS1 | IQCE | protein_coding | 0.65 | 4.51E-06 |
| AGAP2-AS1 | MT-ND3 | protein_coding | -0.56 | 0.000121276 |
| AGAP2-AS1 | CYP2A6 | protein_coding | -0.58 | 7.28E-05 |
| AGAP2-AS1 | SPPL3 | protein_coding | 0.54 | 0.000248446 |
| AGAP2-AS1 | GLS2 | protein_coding | -0.71 | 2.22E-07 |
| AGAP2-AS1 | SPDYC | protein_coding | -0.74 | 3.74E-08 |
| AGAP2-AS1 | DHTKD1 | protein_coding | -0.72 | 9.71E-08 |
| AGAP2-AS1 | BHMT | protein_coding | -0.56 | 0.000164549 |
| AGAP2-AS1 | ZNF714 | protein_coding | 0.50 | 0.00080733 |
| AGAP2-AS1 | CDKN2AIPNL | protein_coding | 0.51 | 0.000619145 |
| AGAP2-AS1 | TIMP2 | protein_coding | 0.58 | 8.32E-05 |
| AGAP2-AS1 | RNF144B | protein_coding | -0.65 | 4.13E-06 |
| AGAP2-AS1 | CRLF1 | protein_coding | 0.56 | 0.000140892 |
| AGAP2-AS1 | MAJIN | protein_coding | -0.52 | 0.000511046 |
| AGAP2-AS1 | ABCG5 | protein_coding | -0.54 | 0.000274585 |
| AGAP2-AS1 | GTF2F1 | protein_coding | 0.61 | 2.02E-05 |
| AGAP2-AS1 | BLMH | protein_coding | 0.57 | 8.63E-05 |
| AGAP2-AS1 | CCL14 | protein_coding | -0.69 | 7.59E-07 |
| AGAP2-AS1 | GLIS2 | protein_coding | 0.72 | 1.40E-07 |
| AGAP2-AS1 | PPCDC | protein_coding | 0.63 | 1.17E-05 |
| AGAP2-AS1 | CEP290 | protein_coding | 0.57 | 0.000108417 |
| AGAP2-AS1 | LGSN | protein_coding | -0.69 | 7.36E-07 |
| AGAP2-AS1 | CSAD | protein_coding | -0.72 | 1.24E-07 |
| AGAP2-AS1 | SIK3 | protein_coding | -0.61 | 2.35E-05 |
| AGAP2-AS1 | HGFAC | protein_coding | -0.62 | 1.63E-05 |
| AGAP2-AS1 | POLD1 | protein_coding | 0.61 | 2.22E-05 |
| AGAP2-AS1 | ABCG8 | protein_coding | -0.54 | 0.000264193 |
| AGAP2-AS1 | ABAT | protein_coding | -0.69 | 6.42E-07 |
| AGAP2-AS1 | HES4 | protein_coding | 0.69 | 7.21E-07 |
| AGAP2-AS1 | POMT2 | protein_coding | 0.64 | 6.36E-06 |
| AGAP2-AS1 | PRR36 | protein_coding | 0.51 | 0.000595501 |
| AGAP2-AS1 | MCU | protein_coding | 0.51 | 0.000697561 |
| AGAP2-AS1 | CFAP53 | protein_coding | 0.56 | 0.000126538 |
| AGAP2-AS1 | RUNDC3A | protein_coding | 0.51 | 0.000696047 |
| AGAP2-AS1 | GYS1 | protein_coding | 0.61 | 2.21E-05 |
| AGAP2-AS1 | UTP14A | protein_coding | 0.59 | 4.61E-05 |
| AGAP2-AS1 | PRKAB2 | protein_coding | -0.57 | 0.000105691 |
| AGAP2-AS1 | TMEM79 | protein_coding | 0.64 | 5.49E-06 |
| AGAP2-AS1 | CHAD | protein_coding | -0.58 | 6.27E-05 |
| AGAP2-AS1 | ZNF185 | protein_coding | 0.64 | 8.22E-06 |
| AGAP2-AS1 | UGP2 | protein_coding | -0.67 | 1.86E-06 |
| AGAP2-AS1 | ZBTB33 | protein_coding | -0.52 | 0.000499 |
| AGAP2-AS1 | CCT7 | protein_coding | 0.51 | 0.000678596 |
| AGAP2-AS1 | CES4A | protein_coding | -0.55 | 0.000228087 |
| AGAP2-AS1 | PPP4R1 | protein_coding | 0.58 | 8.03E-05 |
| AGAP2-AS1 | TRNP1 | protein_coding | 0.67 | 1.89E-06 |
| AGAP2-AS1 | TMCC2 | protein_coding | 0.51 | 0.000608006 |
| AGAP2-AS1 | MPP3 | protein_coding | 0.61 | 2.24E-05 |
| AGAP2-AS1 | TULP3 | protein_coding | 0.56 | 0.000152132 |
| AGAP2-AS1 | COL5A3 | protein_coding | -0.52 | 0.000478303 |
| AGAP2-AS1 | DNAAF2 | protein_coding | -0.54 | 0.000241494 |
| AGAP2-AS1 | TRIM55 | protein_coding | -0.52 | 0.000494222 |
| AGAP2-AS1 | ANO9 | protein_coding | 0.60 | 3.30E-05 |
| AGAP2-AS1 | ZNF430 | protein_coding | 0.61 | 2.34E-05 |
| AGAP2-AS1 | CP | protein_coding | -0.52 | 0.000464321 |
| AGAP2-AS1 | SPINT2 | protein_coding | 0.72 | 1.49E-07 |
| AGAP2-AS1 | ESR1 | protein_coding | -0.69 | 7.34E-07 |
| AGAP2-AS1 | PAQR4 | protein_coding | 0.67 | 1.52E-06 |
| AGAP2-AS1 | GADD45G | protein_coding | -0.54 | 0.000286563 |
| AGAP2-AS1 | ALDH6A1 | protein_coding | -0.68 | 1.22E-06 |
| AGAP2-AS1 | KIF5A | protein_coding | 0.58 | 8.47E-05 |
| AGAP2-AS1 | SMCO2 | protein_coding | 0.52 | 0.00046606 |
| AGAP2-AS1 | CAPG | protein_coding | 0.61 | 2.33E-05 |
| AGAP2-AS1 | MICU3 | protein_coding | -0.58 | 6.01E-05 |
| AGAP2-AS1 | SLC16A2 | protein_coding | -0.63 | 9.35E-06 |
| AGAP2-AS1 | KIAA1549 | protein_coding | 0.56 | 0.000148609 |
| AGAP2-AS1 | DAGLA | protein_coding | 0.63 | 1.12E-05 |
| AGAP2-AS1 | KATNB1 | protein_coding | 0.63 | 9.94E-06 |
| AGAP2-AS1 | BTG3 | protein_coding | 0.53 | 0.000322964 |
| AGAP2-AS1 | SRI | protein_coding | 0.52 | 0.000505834 |
| AGAP2-AS1 | CYP7B1 | protein_coding | -0.52 | 0.000484016 |
| AGAP2-AS1 | CD177 | protein_coding | 0.68 | 1.28E-06 |
| AGAP2-AS1 | HSD11B1 | protein_coding | -0.56 | 0.00016827 |
| AGAP2-AS1 | L2HGDH | protein_coding | -0.52 | 0.000466523 |
| AGAP2-AS1 | MBOAT2 | protein_coding | 0.66 | 3.44E-06 |
| AGAP2-AS1 | PANK3 | protein_coding | -0.68 | 1.26E-06 |
| AGAP2-AS1 | CTTN | protein_coding | 0.53 | 0.000341416 |
| AGAP2-AS1 | TMEM59L | protein_coding | 0.62 | 1.90E-05 |
| AGAP2-AS1 | NPTXR | protein_coding | 0.51 | 0.000672839 |
| AGAP2-AS1 | PBLD | protein_coding | -0.62 | 1.28E-05 |
| AGAP2-AS1 | RIBC2 | protein_coding | 0.65 | 4.37E-06 |
| AGAP2-AS1 | DNAJC5 | protein_coding | 0.65 | 3.79E-06 |
| AGAP2-AS1 | SLC7A1 | protein_coding | 0.59 | 5.33E-05 |
| AGAP2-AS1 | EI24 | protein_coding | -0.53 | 0.000332059 |
| AGAP2-AS1 | IFT57 | protein_coding | 0.58 | 6.89E-05 |
| AGAP2-AS1 | BACE2 | protein_coding | 0.55 | 0.000211007 |
| AGAP2-AS1 | PLA2G2A | protein_coding | -0.58 | 7.66E-05 |
| AGAP2-AS1 | SNF8 | protein_coding | 0.58 | 6.78E-05 |
| AGAP2-AS1 | SC5D | protein_coding | -0.67 | 1.47E-06 |
| AGAP2-AS1 | ZNF668 | protein_coding | 0.70 | 3.21E-07 |
| AGAP2-AS1 | EID2B | protein_coding | 0.61 | 2.84E-05 |
| AGAP2-AS1 | CHRNA4 | protein_coding | -0.63 | 8.75E-06 |
| AGAP2-AS1 | H2BC8 | protein_coding | 0.57 | 9.35E-05 |
| AGAP2-AS1 | CDKN3 | protein_coding | 0.61 | 2.07E-05 |
| AGAP2-AS1 | SLC28A1 | protein_coding | -0.53 | 0.00036581 |
| AGAP2-AS1 | BORCS7 | protein_coding | -0.57 | 0.000101039 |
| AGAP2-AS1 | SKA1 | protein_coding | 0.70 | 3.61E-07 |
| AGAP2-AS1 | DCAF11 | protein_coding | -0.69 | 6.04E-07 |
| AGAP2-AS1 | KIF14 | protein_coding | 0.54 | 0.000309213 |
| AGAP2-AS1 | IDH1 | protein_coding | -0.56 | 0.000134367 |
| AGAP2-AS1 | ADH7 | protein_coding | -0.72 | 1.05E-07 |
| AGAP2-AS1 | FRMD8 | protein_coding | 0.54 | 0.000251403 |
| AGAP2-AS1 | EDC3 | protein_coding | 0.61 | 2.31E-05 |
| AGAP2-AS1 | MAN1C1 | protein_coding | -0.62 | 1.66E-05 |
| AGAP2-AS1 | RAB3D | protein_coding | 0.66 | 3.37E-06 |
| AGAP2-AS1 | KIFC1 | protein_coding | 0.68 | 8.24E-07 |
| AGAP2-AS1 | MTSS1 | protein_coding | -0.77 | 5.75E-09 |
| AGAP2-AS1 | ACVR1C | protein_coding | -0.64 | 7.29E-06 |
| AGAP2-AS1 | SEMA4F | protein_coding | 0.68 | 9.13E-07 |
| AGAP2-AS1 | SENP1 | protein_coding | 0.54 | 0.000312414 |
| AGAP2-AS1 | CCL16 | protein_coding | -0.59 | 4.23E-05 |
| AGAP2-AS1 | SLC1A2 | protein_coding | -0.66 | 2.98E-06 |
| AGAP2-AS1 | STK39 | protein_coding | 0.70 | 3.07E-07 |
| AGAP2-AS1 | AAK1 | protein_coding | 0.53 | 0.000408192 |
| AGAP2-AS1 | PPP1R9B | protein_coding | 0.62 | 1.47E-05 |
| AGAP2-AS1 | CREG1 | protein_coding | -0.61 | 2.50E-05 |
| AGAP2-AS1 | SLC25A33 | protein_coding | -0.51 | 0.00062307 |
| AGAP2-AS1 | CFHR4 | protein_coding | -0.60 | 2.94E-05 |
| AGAP2-AS1 | NR3C2 | protein_coding | -0.55 | 0.000174237 |
| AGAP2-AS1 | PYGL | protein_coding | -0.55 | 0.000212713 |
| AGAP2-AS1 | LEF1 | protein_coding | 0.55 | 0.000232491 |
| AGAP2-AS1 | THNSL1 | protein_coding | -0.63 | 1.22E-05 |
| AGAP2-AS1 | IL3RA | protein_coding | 0.55 | 0.000180872 |
| AGAP2-AS1 | PMAIP1 | protein_coding | 0.53 | 0.000411015 |
| AGAP2-AS1 | TADA2A | protein_coding | 0.56 | 0.000163552 |
| AGAP2-AS1 | TMEM253 | protein_coding | 0.56 | 0.000126713 |
| AGAP2-AS1 | AQP11 | protein_coding | -0.63 | 1.10E-05 |
| AGAP2-AS1 | SYT13 | protein_coding | 0.54 | 0.000299148 |
| AGAP2-AS1 | MACO1 | protein_coding | -0.63 | 8.46E-06 |
| AGAP2-AS1 | PIF1 | protein_coding | 0.62 | 1.67E-05 |
| AGAP2-AS1 | FAM151A | protein_coding | -0.67 | 1.49E-06 |
| AGAP2-AS1 | KLF10 | protein_coding | -0.52 | 0.000568076 |
| AGAP2-AS1 | TLCD4 | protein_coding | -0.61 | 1.94E-05 |
| AGAP2-AS1 | OLFM2 | protein_coding | -0.50 | 0.000836099 |
| AGAP2-AS1 | BEX2 | protein_coding | 0.64 | 6.87E-06 |
| AGAP2-AS1 | C16orf96 | protein_coding | -0.62 | 1.78E-05 |
| AGAP2-AS1 | MN1 | protein_coding | 0.65 | 5.25E-06 |
| AGAP2-AS1 | ETFDH | protein_coding | -0.66 | 3.25E-06 |
| AGAP2-AS1 | AK8 | protein_coding | 0.65 | 4.84E-06 |
| AGAP2-AS1 | AADAT | protein_coding | -0.64 | 7.14E-06 |
| AGAP2-AS1 | OSBPL7 | protein_coding | 0.62 | 1.42E-05 |
| AGAP2-AS1 | C7orf61 | protein_coding | 0.57 | 9.67E-05 |
| AGAP2-AS1 | ABCC1 | protein_coding | 0.56 | 0.000126012 |
| AGAP2-AS1 | USP30 | protein_coding | -0.53 | 0.000337022 |
| AGAP2-AS1 | THADA | protein_coding | 0.59 | 5.36E-05 |
| AGAP2-AS1 | LRRC40 | protein_coding | -0.53 | 0.000352444 |
| AGAP2-AS1 | ZFHX4 | protein_coding | -0.62 | 1.82E-05 |
| AGAP2-AS1 | SNX12 | protein_coding | 0.57 | 9.59E-05 |
| AGAP2-AS1 | RAB24 | protein_coding | 0.52 | 0.000502519 |
| AGAP2-AS1 | IGF1 | protein_coding | -0.61 | 2.08E-05 |
| AGAP2-AS1 | EFHD1 | protein_coding | -0.56 | 0.000153511 |
| AGAP2-AS1 | MCM5 | protein_coding | 0.66 | 3.34E-06 |
| AGAP2-AS1 | KIF24 | protein_coding | 0.60 | 3.27E-05 |
| AGAP2-AS1 | AL163636.3 | protein_coding | -0.60 | 2.94E-05 |
| AGAP2-AS1 | KCNQ1 | protein_coding | 0.63 | 1.17E-05 |
| AGAP2-AS1 | PEPD | protein_coding | -0.53 | 0.000395577 |
| AGAP2-AS1 | NUDT16 | protein_coding | -0.57 | 0.000113964 |
| AGAP2-AS1 | CD3EAP | protein_coding | 0.51 | 0.000662354 |
| AGAP2-AS1 | RAD54L2 | protein_coding | -0.66 | 3.43E-06 |
| AGAP2-AS1 | TMEM163 | protein_coding | 0.73 | 6.11E-08 |
| AGAP2-AS1 | MCUB | protein_coding | 0.65 | 4.81E-06 |
| AGAP2-AS1 | NCAPH | protein_coding | 0.73 | 5.34E-08 |
| AGAP2-AS1 | MMUT | protein_coding | -0.65 | 4.44E-06 |
| AGAP2-AS1 | ZBED8 | protein_coding | 0.62 | 1.74E-05 |
| AGAP2-AS1 | HOXB6 | protein_coding | 0.51 | 0.000702813 |
| AGAP2-AS1 | ANKDD1A | protein_coding | 0.51 | 0.000668756 |
| AGAP2-AS1 | GNRH2 | protein_coding | -0.53 | 0.000383015 |
| AGAP2-AS1 | MC1R | protein_coding | 0.73 | 5.01E-08 |
| AGAP2-AS1 | DDX27 | protein_coding | 0.63 | 1.13E-05 |
| AGAP2-AS1 | SRPRA | protein_coding | -0.54 | 0.000234535 |
| AGAP2-AS1 | KRBA1 | protein_coding | 0.56 | 0.00012472 |
| AGAP2-AS1 | CCDC42 | protein_coding | -0.61 | 2.27E-05 |
| AGAP2-AS1 | ZKSCAN5 | protein_coding | 0.63 | 9.87E-06 |
| AGAP2-AS1 | PRSS16 | protein_coding | 0.62 | 1.44E-05 |
| AGAP2-AS1 | ERRFI1 | protein_coding | -0.51 | 0.000610494 |
| AGAP2-AS1 | BRICD5 | protein_coding | 0.52 | 0.000577229 |
| AGAP2-AS1 | POR | protein_coding | -0.64 | 8.22E-06 |
| AGAP2-AS1 | LRRC2 | protein_coding | -0.62 | 1.81E-05 |
| AGAP2-AS1 | NOL3 | protein_coding | 0.61 | 2.17E-05 |
| AGAP2-AS1 | TTC38 | protein_coding | -0.66 | 2.88E-06 |
| AGAP2-AS1 | EPS8L1 | protein_coding | 0.67 | 1.98E-06 |
| AGAP2-AS1 | CENPJ | protein_coding | 0.54 | 0.000289234 |
| AGAP2-AS1 | WDR54 | protein_coding | 0.76 | 8.86E-09 |
| AGAP2-AS1 | ABCA1 | protein_coding | -0.59 | 5.68E-05 |
| AGAP2-AS1 | MYT1 | protein_coding | 0.52 | 0.00049016 |
| AGAP2-AS1 | RNPS1 | protein_coding | 0.64 | 7.37E-06 |
| AGAP2-AS1 | FAM222B | protein_coding | 0.56 | 0.000121652 |
| AGAP2-AS1 | NFATC4 | protein_coding | 0.57 | 9.15E-05 |
| AGAP2-AS1 | PROZ | protein_coding | -0.53 | 0.000354806 |
| AGAP2-AS1 | GPR182 | protein_coding | -0.61 | 2.39E-05 |
| AGAP2-AS1 | ACBD5 | protein_coding | -0.53 | 0.000341122 |
| AGAP2-AS1 | RHOB | protein_coding | -0.60 | 4.13E-05 |
| AGAP2-AS1 | ABHD17B | protein_coding | -0.53 | 0.000347558 |
| AGAP2-AS1 | POLR1A | protein_coding | 0.62 | 1.36E-05 |
| AGAP2-AS1 | GPX8 | protein_coding | 0.59 | 4.81E-05 |
| AGAP2-AS1 | CCDC112 | protein_coding | 0.70 | 3.89E-07 |
| AGAP2-AS1 | NEK11 | protein_coding | 0.58 | 6.78E-05 |
| AGAP2-AS1 | DUSP4 | protein_coding | 0.52 | 0.000529244 |
| AGAP2-AS1 | SGPP2 | protein_coding | 0.57 | 0.000103463 |
| AGAP2-AS1 | TSPAN15 | protein_coding | 0.71 | 1.95E-07 |
| AGAP2-AS1 | XRCC2 | protein_coding | 0.61 | 2.14E-05 |
| AGAP2-AS1 | MOSMO | protein_coding | -0.54 | 0.000257537 |
| AGAP2-AS1 | NADK | protein_coding | -0.57 | 0.000106173 |
| AGAP2-AS1 | SLC6A6 | protein_coding | 0.58 | 7.85E-05 |
| AGAP2-AS1 | GCLC | protein_coding | -0.64 | 8.35E-06 |
| AGAP2-AS1 | MYL12A | protein_coding | 0.52 | 0.000448679 |
| AGAP2-AS1 | PACC1 | protein_coding | 0.72 | 1.47E-07 |
| AGAP2-AS1 | EIF4EBP2 | protein_coding | -0.58 | 6.07E-05 |
| AGAP2-AS1 | HAGH | protein_coding | -0.59 | 5.38E-05 |
| AGAP2-AS1 | MTMR3 | protein_coding | 0.52 | 0.000468145 |
| AGAP2-AS1 | GIPR | protein_coding | 0.68 | 8.83E-07 |
| AGAP2-AS1 | COL13A1 | protein_coding | 0.60 | 4.11E-05 |
| AGAP2-AS1 | SPATA12 | protein_coding | 0.62 | 1.35E-05 |
| AGAP2-AS1 | UBE2D1 | protein_coding | 0.53 | 0.000382318 |
| AGAP2-AS1 | DYSF | protein_coding | -0.52 | 0.000565984 |
| AGAP2-AS1 | GMNC | protein_coding | -0.59 | 4.91E-05 |
| AGAP2-AS1 | CTSO | protein_coding | -0.64 | 5.94E-06 |
| AGAP2-AS1 | VPS11 | protein_coding | 0.51 | 0.00066969 |
| AGAP2-AS1 | SCCPDH | protein_coding | -0.52 | 0.000519865 |
| AGAP2-AS1 | SUOX | protein_coding | -0.60 | 3.25E-05 |
| AGAP2-AS1 | PHYH | protein_coding | -0.67 | 1.57E-06 |
| AGAP2-AS1 | GRAMD1A | protein_coding | 0.52 | 0.00046341 |
| AGAP2-AS1 | H2AX | protein_coding | 0.70 | 3.14E-07 |
| AGAP2-AS1 | FAM111B | protein_coding | 0.70 | 4.52E-07 |
| AGAP2-AS1 | LEAP2 | protein_coding | -0.65 | 4.53E-06 |
| AGAP2-AS1 | B3GALNT1 | protein_coding | 0.55 | 0.000231078 |
| AGAP2-AS1 | UBXN1 | protein_coding | 0.54 | 0.000308032 |
| AGAP2-AS1 | DUT | protein_coding | 0.58 | 6.07E-05 |
| AGAP2-AS1 | PDK4 | protein_coding | -0.55 | 0.000227871 |
| AGAP2-AS1 | FANCF | protein_coding | 0.53 | 0.000344209 |
| AGAP2-AS1 | FBXL3 | protein_coding | -0.57 | 0.000106434 |
| AGAP2-AS1 | CAPN1 | protein_coding | 0.64 | 7.21E-06 |
| AGAP2-AS1 | HIBCH | protein_coding | -0.61 | 2.01E-05 |
| AGAP2-AS1 | ARHGAP11A | protein_coding | 0.61 | 2.11E-05 |
| AGAP2-AS1 | KCTD11 | protein_coding | 0.50 | 0.000853008 |
| AGAP2-AS1 | CDADC1 | protein_coding | -0.62 | 1.37E-05 |
| AGAP2-AS1 | NDC80 | protein_coding | 0.67 | 1.51E-06 |
| AGAP2-AS1 | SPRR3 | protein_coding | 0.52 | 0.000458521 |
| AGAP2-AS1 | EPCAM | protein_coding | 0.61 | 2.54E-05 |
| AGAP2-AS1 | ALDH8A1 | protein_coding | -0.52 | 0.00044825 |
| AGAP2-AS1 | APOL5 | protein_coding | -0.62 | 1.57E-05 |
| AGAP2-AS1 | ACOX1 | protein_coding | -0.67 | 1.57E-06 |
| AGAP2-AS1 | SMIM14 | protein_coding | -0.69 | 4.95E-07 |
| AGAP2-AS1 | TAGLN2 | protein_coding | 0.62 | 1.71E-05 |
| AGAP2-AS1 | CENPU | protein_coding | 0.70 | 3.27E-07 |
| AGAP2-AS1 | ENTPD5 | protein_coding | -0.69 | 5.71E-07 |
| AGAP2-AS1 | FAM72C | protein_coding | 0.61 | 2.00E-05 |
| AGAP2-AS1 | TSC22D4 | protein_coding | 0.55 | 0.000220398 |
| AGAP2-AS1 | FDX1 | protein_coding | -0.56 | 0.000164356 |
| AGAP2-AS1 | MMACHC | protein_coding | -0.61 | 2.55E-05 |
| AGAP2-AS1 | DMKN | protein_coding | 0.67 | 1.55E-06 |
| AGAP2-AS1 | IQGAP2 | protein_coding | -0.63 | 1.20E-05 |
| AGAP2-AS1 | MPPED1 | protein_coding | -0.62 | 1.55E-05 |
| AGAP2-AS1 | MPP4 | protein_coding | 0.53 | 0.000388742 |
| AGAP2-AS1 | RUNDC3B | protein_coding | -0.52 | 0.000458983 |
| AGAP2-AS1 | ADGRA3 | protein_coding | -0.50 | 0.000777209 |
| AGAP2-AS1 | GALM | protein_coding | -0.58 | 8.06E-05 |
| AGAP2-AS1 | WWP1 | protein_coding | -0.56 | 0.000144903 |
| AGAP2-AS1 | PLLP | protein_coding | 0.55 | 0.00021378 |
| AGAP2-AS1 | DBH | protein_coding | -0.64 | 7.98E-06 |
| AGAP2-AS1 | CLDN7 | protein_coding | 0.52 | 0.000490066 |
| AGAP2-AS1 | DCLRE1C | protein_coding | 0.55 | 0.00022482 |
| AGAP2-AS1 | NCOA4 | protein_coding | -0.62 | 1.39E-05 |
| AGAP2-AS1 | TLDC2 | protein_coding | 0.68 | 1.28E-06 |
| AGAP2-AS1 | VSIG10 | protein_coding | 0.51 | 0.000666828 |
| AGAP2-AS1 | SMARCD1 | protein_coding | 0.60 | 3.88E-05 |
| AGAP2-AS1 | KLC3 | protein_coding | 0.53 | 0.000354622 |
| AGAP2-AS1 | MZF1 | protein_coding | 0.54 | 0.000250604 |
| AGAP2-AS1 | GCA | protein_coding | 0.58 | 6.22E-05 |
| AGAP2-AS1 | BEX3 | protein_coding | 0.54 | 0.000237882 |
| AGAP2-AS1 | STPG3 | protein_coding | -0.52 | 0.000435543 |
| AGAP2-AS1 | ICK | protein_coding | -0.61 | 2.18E-05 |
| AGAP2-AS1 | TUBE1 | protein_coding | -0.71 | 1.99E-07 |
| AGAP2-AS1 | ITIH4 | protein_coding | -0.61 | 2.65E-05 |
| AGAP2-AS1 | CACNB1 | protein_coding | 0.66 | 3.00E-06 |
| AGAP2-AS1 | TAOK2 | protein_coding | 0.61 | 2.77E-05 |
| AGAP2-AS1 | DNAJC18 | protein_coding | 0.64 | 7.27E-06 |
| AGAP2-AS1 | ENPEP | protein_coding | -0.64 | 8.07E-06 |
| AGAP2-AS1 | ANXA3 | protein_coding | 0.58 | 8.50E-05 |
| AGAP2-AS1 | FBXL2 | protein_coding | 0.65 | 3.53E-06 |
| AGAP2-AS1 | C10orf95 | protein_coding | 0.52 | 0.000486659 |
| AGAP2-AS1 | MAMDC4 | protein_coding | -0.57 | 0.00011475 |
| AGAP2-AS1 | PPP1R1A | protein_coding | -0.70 | 3.59E-07 |
| AGAP2-AS1 | LAMA5 | protein_coding | 0.60 | 3.50E-05 |
| AGAP2-AS1 | PEBP1 | protein_coding | -0.58 | 8.45E-05 |
| AGAP2-AS1 | UBR2 | protein_coding | -0.54 | 0.000279865 |
| AGAP2-AS1 | RNF152 | protein_coding | -0.54 | 0.000289575 |
| AGAP2-AS1 | GIPC1 | protein_coding | 0.67 | 1.74E-06 |
| AGAP2-AS1 | JMJD6 | protein_coding | 0.66 | 3.32E-06 |
| AGAP2-AS1 | HBB | protein_coding | -0.53 | 0.000426586 |
| AGAP2-AS1 | ZNF629 | protein_coding | 0.55 | 0.000169522 |
| AGAP2-AS1 | ABI2 | protein_coding | 0.59 | 4.44E-05 |
| AGAP2-AS1 | C16orf70 | protein_coding | -0.59 | 4.41E-05 |
| AGAP2-AS1 | LRRC28 | protein_coding | -0.64 | 5.55E-06 |
| AGAP2-AS1 | CHAMP1 | protein_coding | -0.55 | 0.000229475 |
| AGAP2-AS1 | HBA2 | protein_coding | -0.55 | 0.000212095 |
| AGAP2-AS1 | RDM1 | protein_coding | 0.58 | 6.12E-05 |
| AGAP2-AS1 | ZNF512 | protein_coding | 0.66 | 2.32E-06 |
| AGAP2-AS1 | AGAP1 | protein_coding | 0.58 | 6.02E-05 |
| AGAP2-AS1 | ABCA8 | protein_coding | -0.62 | 1.29E-05 |
| AGAP2-AS1 | AGMAT | protein_coding | -0.62 | 1.52E-05 |
| AGAP2-AS1 | LETM2 | protein_coding | 0.60 | 4.05E-05 |
| AGAP2-AS1 | OSBPL3 | protein_coding | 0.68 | 1.16E-06 |
| AGAP2-AS1 | MT1M | protein_coding | -0.58 | 6.92E-05 |
| AGAP2-AS1 | ANXA2 | protein_coding | 0.71 | 2.66E-07 |
| AGAP2-AS1 | SERPINH1 | protein_coding | 0.55 | 0.000177315 |
| AGAP2-AS1 | RAD51 | protein_coding | 0.77 | 4.28E-09 |
| AGAP2-AS1 | ARPP21 | protein_coding | -0.51 | 0.00061558 |
| AGAP2-AS1 | VNN3 | protein_coding | -0.55 | 0.000179507 |
| AGAP2-AS1 | ASPG | protein_coding | -0.62 | 1.75E-05 |
| AGAP2-AS1 | IFNAR1 | protein_coding | -0.51 | 0.000639613 |
| AGAP2-AS1 | GPLD1 | protein_coding | -0.71 | 2.72E-07 |
| AGAP2-AS1 | TIMM8A | protein_coding | -0.60 | 2.96E-05 |
| AGAP2-AS1 | DIS3L2 | protein_coding | 0.62 | 1.58E-05 |
| AGAP2-AS1 | SELENBP1 | protein_coding | -0.63 | 1.01E-05 |
| AGAP2-AS1 | SIGMAR1 | protein_coding | -0.54 | 0.000299129 |
| AGAP2-AS1 | XKR5 | protein_coding | 0.57 | 9.71E-05 |
| AGAP2-AS1 | HS3ST1 | protein_coding | 0.50 | 0.000775174 |
| AGAP2-AS1 | FUS | protein_coding | 0.58 | 7.41E-05 |
| AGAP2-AS1 | MST1R | protein_coding | 0.53 | 0.000339741 |
| AGAP2-AS1 | ASNS | protein_coding | 0.69 | 6.80E-07 |
| AGAP2-AS1 | LGI1 | protein_coding | -0.61 | 2.78E-05 |
| AGAP2-AS1 | TRIP10 | protein_coding | 0.68 | 8.91E-07 |
| AGAP2-AS1 | LGALS3BP | protein_coding | 0.52 | 0.000561341 |
| AGAP2-AS1 | SEPTIN9 | protein_coding | 0.57 | 9.07E-05 |
| AGAP2-AS1 | ABCB9 | protein_coding | 0.67 | 1.82E-06 |
| AGAP2-AS1 | NUDT6 | protein_coding | -0.51 | 0.000710168 |
| AGAP2-AS1 | SRD5A1 | protein_coding | -0.55 | 0.000189561 |
| AGAP2-AS1 | MTUS2 | protein_coding | -0.54 | 0.000255742 |
| AGAP2-AS1 | CD302 | protein_coding | -0.65 | 4.72E-06 |
| AGAP2-AS1 | TRIM56 | protein_coding | 0.55 | 0.000178943 |
| AGAP2-AS1 | H6PD | protein_coding | -0.51 | 0.00064059 |
| AGAP2-AS1 | HMGA1 | protein_coding | 0.63 | 8.83E-06 |
| AGAP2-AS1 | CCDC78 | protein_coding | 0.62 | 1.58E-05 |
| AGAP2-AS1 | GDPD4 | protein_coding | -0.51 | 0.00066745 |
| AGAP2-AS1 | PXN | protein_coding | 0.58 | 7.42E-05 |
| AGAP2-AS1 | ABL1 | protein_coding | 0.57 | 0.000119018 |
| AGAP2-AS1 | SINHCAF | protein_coding | 0.58 | 7.60E-05 |
| AGAP2-AS1 | NLRP6 | protein_coding | -0.61 | 2.50E-05 |
| AGAP2-AS1 | SMARCD3 | protein_coding | 0.58 | 6.02E-05 |
| AGAP2-AS1 | CMTM6 | protein_coding | -0.54 | 0.000270007 |
| AGAP2-AS1 | DPYS | protein_coding | -0.56 | 0.000156905 |
| AGAP2-AS1 | XAF1 | protein_coding | -0.62 | 1.78E-05 |
| AGAP2-AS1 | TUBB3 | protein_coding | 0.74 | 2.47E-08 |
| AGAP2-AS1 | FAM50A | protein_coding | 0.64 | 6.51E-06 |
| AGAP2-AS1 | PPFIBP2 | protein_coding | -0.63 | 1.12E-05 |
| AGAP2-AS1 | PAX9 | protein_coding | 0.60 | 3.11E-05 |
| AGAP2-AS1 | ZYG11A | protein_coding | -0.65 | 4.52E-06 |
| AGAP2-AS1 | CEP72 | protein_coding | 0.51 | 0.000633174 |
| AGAP2-AS1 | FTCD | protein_coding | -0.52 | 0.000475937 |
| AGAP2-AS1 | MARCHF4 | protein_coding | 0.55 | 0.000204957 |
| AGAP2-AS1 | CDO1 | protein_coding | -0.62 | 1.61E-05 |
| AGAP2-AS1 | NT5C | protein_coding | 0.61 | 2.16E-05 |
| AGAP2-AS1 | CPXM1 | protein_coding | 0.56 | 0.000129141 |
| AGAP2-AS1 | CNFN | protein_coding | 0.66 | 2.95E-06 |
| AGAP2-AS1 | STYX | protein_coding | -0.62 | 1.36E-05 |
| AGAP2-AS1 | DEF8 | protein_coding | 0.60 | 4.06E-05 |
| AGAP2-AS1 | ZHX1 | protein_coding | -0.63 | 8.98E-06 |
| AGAP2-AS1 | CA14 | protein_coding | -0.56 | 0.000122253 |
| AGAP2-AS1 | IL27 | protein_coding | -0.69 | 5.94E-07 |
| AGAP2-AS1 | PDCL3 | protein_coding | 0.67 | 1.85E-06 |
| AGAP2-AS1 | TLCD3A | protein_coding | 0.64 | 8.06E-06 |
| AGAP2-AS1 | ALDH5A1 | protein_coding | -0.71 | 1.61E-07 |
| AGAP2-AS1 | DGCR2 | protein_coding | 0.57 | 9.07E-05 |
| AGAP2-AS1 | FYB2 | protein_coding | -0.57 | 0.000114938 |
| AGAP2-AS1 | MXD3 | protein_coding | 0.57 | 9.04E-05 |
| AGAP2-AS1 | SERPINF1 | protein_coding | -0.56 | 0.00016585 |
| AGAP2-AS1 | TMEM87A | protein_coding | 0.56 | 0.000168561 |
| AGAP2-AS1 | EIPR1 | protein_coding | 0.53 | 0.000381648 |
| AGAP2-AS1 | ADHFE1 | protein_coding | -0.59 | 4.90E-05 |
| AGAP2-AS1 | FHOD1 | protein_coding | 0.74 | 3.87E-08 |
| AGAP2-AS1 | AXIN1 | protein_coding | 0.56 | 0.000155389 |
| AGAP2-AS1 | WDR64 | protein_coding | -0.60 | 3.64E-05 |
| AGAP2-AS1 | TMEM231 | protein_coding | 0.63 | 8.55E-06 |
| AGAP2-AS1 | BTBD2 | protein_coding | 0.59 | 5.34E-05 |
| AGAP2-AS1 | SGO2 | protein_coding | 0.66 | 2.46E-06 |
| AGAP2-AS1 | EFTUD2 | protein_coding | 0.70 | 3.77E-07 |
| AGAP2-AS1 | C11orf91 | protein_coding | 0.54 | 0.000258194 |
| AGAP2-AS1 | FSTL3 | protein_coding | 0.54 | 0.000304337 |
| AGAP2-AS1 | DUSP28 | protein_coding | 0.53 | 0.000410571 |
| AGAP2-AS1 | E2F4 | protein_coding | 0.63 | 1.03E-05 |
| AGAP2-AS1 | LRRC1 | protein_coding | 0.72 | 1.18E-07 |
| AGAP2-AS1 | PCSK6 | protein_coding | -0.58 | 7.31E-05 |
| AGAP2-AS1 | PECR | protein_coding | -0.61 | 2.06E-05 |
| AGAP2-AS1 | TMEM127 | protein_coding | 0.56 | 0.000133537 |
| AGAP2-AS1 | PTPN9 | protein_coding | 0.54 | 0.000282851 |
| AGAP2-AS1 | EHBP1L1 | protein_coding | 0.57 | 0.000119041 |
| AGAP2-AS1 | SERPIND1 | protein_coding | -0.56 | 0.000157923 |
| AGAP2-AS1 | APOBEC3B | protein_coding | 0.51 | 0.000638072 |
| AGAP2-AS1 | HELLS | protein_coding | 0.68 | 1.12E-06 |
| AGAP2-AS1 | PDHB | protein_coding | -0.57 | 9.97E-05 |
| AGAP2-AS1 | LPA | protein_coding | -0.55 | 0.000195682 |
| AGAP2-AS1 | ADCY1 | protein_coding | -0.57 | 0.000101977 |
| AGAP2-AS1 | TUBA1A | protein_coding | 0.65 | 4.63E-06 |
| AGAP2-AS1 | NMB | protein_coding | 0.66 | 2.95E-06 |
| AGAP2-AS1 | GPR62 | protein_coding | -0.55 | 0.000211402 |
| AGAP2-AS1 | ARL6IP6 | protein_coding | 0.52 | 0.000464221 |
| AGAP2-AS1 | ITGA3 | protein_coding | 0.67 | 2.01E-06 |
| AGAP2-AS1 | PPT1 | protein_coding | 0.57 | 9.26E-05 |
| AGAP2-AS1 | TRIM28 | protein_coding | 0.64 | 6.65E-06 |
| AGAP2-AS1 | FMO4 | protein_coding | -0.71 | 1.62E-07 |
| AGAP2-AS1 | PHLDA3 | protein_coding | 0.63 | 9.61E-06 |
| AGAP2-AS1 | TMEM135 | protein_coding | -0.51 | 0.000622754 |
| AGAP2-AS1 | STX4 | protein_coding | 0.55 | 0.00019213 |
| AGAP2-AS1 | TLCD3B | protein_coding | 0.65 | 4.50E-06 |
| AGAP2-AS1 | KPNA1 | protein_coding | -0.54 | 0.000242453 |
| AGAP2-AS1 | FOXO1 | protein_coding | -0.64 | 7.35E-06 |
| AGAP2-AS1 | TCF19 | protein_coding | 0.69 | 4.88E-07 |
| AGAP2-AS1 | FOXK1 | protein_coding | 0.50 | 0.00082639 |
| AGAP2-AS1 | DENND11 | protein_coding | -0.52 | 0.000556265 |
| AGAP2-AS1 | SOCS7 | protein_coding | 0.54 | 0.000251416 |
| AGAP2-AS1 | ETNPPL | protein_coding | -0.56 | 0.000126121 |
| AGAP2-AS1 | CYP2C19 | protein_coding | -0.72 | 9.99E-08 |
| AGAP2-AS1 | CRHR1 | protein_coding | 0.65 | 4.13E-06 |
| AGAP2-AS1 | NCOA2 | protein_coding | -0.59 | 4.74E-05 |
| AGAP2-AS1 | RCL1 | protein_coding | -0.70 | 4.54E-07 |
| AGAP2-AS1 | UGT2A1 | protein_coding | -0.54 | 0.000271839 |
| AGAP2-AS1 | OR13C5 | protein_coding | -0.58 | 7.90E-05 |
| AGAP2-AS1 | FMO5 | protein_coding | -0.70 | 3.31E-07 |
| AGAP2-AS1 | FLVCR2 | protein_coding | -0.53 | 0.000415928 |
| AGAP2-AS1 | SOX9 | protein_coding | 0.50 | 0.000826619 |
| AGAP2-AS1 | SLC7A6 | protein_coding | 0.57 | 0.000115931 |
| AGAP2-AS1 | DOK1 | protein_coding | 0.72 | 8.55E-08 |
| AGAP2-AS1 | APBA1 | protein_coding | -0.52 | 0.000547502 |
| AGAP2-AS1 | SNTG1 | protein_coding | -0.62 | 1.30E-05 |
| AGAP2-AS1 | ANP32A | protein_coding | 0.53 | 0.000404707 |
| AGAP2-AS1 | RNFT2 | protein_coding | 0.67 | 1.69E-06 |
| AGAP2-AS1 | CSNK1G2 | protein_coding | 0.52 | 0.000440506 |
| AGAP2-AS1 | BIRC5 | protein_coding | 0.74 | 4.41E-08 |
| AGAP2-AS1 | RAB26 | protein_coding | -0.58 | 6.38E-05 |
| AGAP2-AS1 | AQP9 | protein_coding | -0.57 | 0.000107982 |
| AGAP2-AS1 | GCNT3 | protein_coding | 0.68 | 8.76E-07 |
| AGAP2-AS1 | CCT2 | protein_coding | 0.59 | 4.99E-05 |
| AGAP2-AS1 | PAPSS2 | protein_coding | -0.51 | 0.000736238 |
| AGAP2-AS1 | ZDHHC13 | protein_coding | 0.73 | 5.55E-08 |
| AGAP2-AS1 | ACRV1 | protein_coding | 0.57 | 9.72E-05 |
| AGAP2-AS1 | CBX3 | protein_coding | 0.58 | 7.22E-05 |
| AGAP2-AS1 | TMEM185A | protein_coding | 0.62 | 1.40E-05 |
| AGAP2-AS1 | STARD10 | protein_coding | -0.58 | 8.00E-05 |
| AGAP2-AS1 | CUL7 | protein_coding | 0.52 | 0.000507041 |
| AGAP2-AS1 | SYT9 | protein_coding | -0.51 | 0.000738119 |
| AGAP2-AS1 | PRR5 | protein_coding | -0.52 | 0.000469547 |
| AGAP2-AS1 | FOLH1 | protein_coding | -0.62 | 1.87E-05 |
| AGAP2-AS1 | ANKRD13B | protein_coding | 0.68 | 1.01E-06 |
| AGAP2-AS1 | AGBL4 | protein_coding | -0.65 | 4.64E-06 |
| AGAP2-AS1 | TCTEX1D2 | protein_coding | 0.63 | 1.19E-05 |
| AGAP2-AS1 | HJURP | protein_coding | 0.65 | 4.15E-06 |
| AGAP2-AS1 | MECP2 | protein_coding | 0.51 | 0.000675745 |
| AGAP2-AS1 | MIA3 | protein_coding | -0.68 | 1.32E-06 |
| AGAP2-AS1 | HMGXB3 | protein_coding | 0.57 | 0.000105833 |
| AGAP2-AS1 | CCDC28A | protein_coding | -0.55 | 0.000185907 |
| AGAP2-AS1 | PTEN | protein_coding | -0.56 | 0.000147613 |
| AGAP2-AS1 | MCM2 | protein_coding | 0.72 | 9.32E-08 |
| AGAP2-AS1 | RNASE4 | protein_coding | -0.61 | 1.93E-05 |
| AGAP2-AS1 | GOLGA6B | protein_coding | -0.60 | 3.39E-05 |
| AGAP2-AS1 | KIF2C | protein_coding | 0.71 | 1.88E-07 |
| AGAP2-AS1 | AATF | protein_coding | 0.65 | 4.05E-06 |
| AGAP2-AS1 | AC093155.3 | protein_coding | -0.61 | 1.92E-05 |
| AGAP2-AS1 | ACADM | protein_coding | -0.67 | 1.64E-06 |
| AGAP2-AS1 | PRSS23 | protein_coding | 0.61 | 2.57E-05 |
| AGAP2-AS1 | SGO1 | protein_coding | 0.53 | 0.000370542 |
| AGAP2-AS1 | BLOC1S3 | protein_coding | 0.63 | 1.18E-05 |
| AGAP2-AS1 | ACBD6 | protein_coding | 0.51 | 0.000761606 |
| AGAP2-AS1 | UAP1 | protein_coding | -0.61 | 2.11E-05 |
| AGAP2-AS1 | INSL3 | protein_coding | 0.61 | 2.57E-05 |
| AGAP2-AS1 | CYP11A1 | protein_coding | -0.51 | 0.000709414 |
| AGAP2-AS1 | ZYG11B | protein_coding | -0.62 | 1.32E-05 |
| AGAP2-AS1 | ITPR2 | protein_coding | -0.65 | 4.31E-06 |
| AGAP2-AS1 | TMEM147 | protein_coding | 0.58 | 7.80E-05 |
| AGAP2-AS1 | TPD52L1 | protein_coding | -0.59 | 4.98E-05 |
| AGAP2-AS1 | LYVE1 | protein_coding | -0.56 | 0.000168362 |
| AGAP2-AS1 | ADH4 | protein_coding | -0.55 | 0.000222285 |
| AGAP2-AS1 | LCN12 | protein_coding | -0.51 | 0.000582788 |
| AGAP2-AS1 | METTL1 | protein_coding | 0.55 | 0.00019951 |
| AGAP2-AS1 | MASP1 | protein_coding | -0.55 | 0.000181305 |
| AGAP2-AS1 | ENDOD1 | protein_coding | 0.57 | 9.54E-05 |
| AGAP2-AS1 | TNIP2 | protein_coding | 0.51 | 0.000638757 |
| AGAP2-AS1 | APOC2 | protein_coding | -0.59 | 5.68E-05 |
| AGAP2-AS1 | NUSAP1 | protein_coding | 0.65 | 5.36E-06 |
| AGAP2-AS1 | SERPINC1 | protein_coding | -0.57 | 0.000111069 |
| AGAP2-AS1 | GRIN2B | protein_coding | -0.57 | 0.000100238 |
| AGAP2-AS1 | FES | protein_coding | -0.54 | 0.000254822 |
| AGAP2-AS1 | PAFAH1B3 | protein_coding | 0.70 | 4.12E-07 |
| AGAP2-AS1 | PLA2G4E | protein_coding | 0.56 | 0.000161051 |
| AGAP2-AS1 | SLC22A12 | protein_coding | -0.51 | 0.00075757 |
| AGAP2-AS1 | CSNK2A1 | protein_coding | 0.60 | 3.75E-05 |
| AGAP2-AS1 | TACC3 | protein_coding | 0.59 | 4.81E-05 |
| AGAP2-AS1 | AK3 | protein_coding | -0.61 | 1.97E-05 |
| AGAP2-AS1 | BRWD1 | protein_coding | -0.53 | 0.000414511 |
| AGAP2-AS1 | CSNK1D | protein_coding | 0.63 | 1.18E-05 |
| AGAP2-AS1 | RNF215 | protein_coding | 0.58 | 7.29E-05 |
| AGAP2-AS1 | PDE3B | protein_coding | -0.51 | 0.000581996 |
| AGAP2-AS1 | TMEM192 | protein_coding | -0.60 | 4.06E-05 |
| AGAP2-AS1 | LARP4 | protein_coding | -0.57 | 0.000100934 |
| AGAP2-AS1 | TBC1D24 | protein_coding | -0.54 | 0.000262961 |
| AGAP2-AS1 | SHCBP1 | protein_coding | 0.74 | 3.95E-08 |
| AGAP2-AS1 | IGBP1 | protein_coding | 0.65 | 3.57E-06 |
| AGAP2-AS1 | LPCAT1 | protein_coding | 0.59 | 5.38E-05 |
| AGAP2-AS1 | RAD51AP1 | protein_coding | 0.62 | 1.86E-05 |
| AGAP2-AS1 | UBE2C | protein_coding | 0.74 | 2.42E-08 |
| AGAP2-AS1 | CAVIN1 | protein_coding | 0.62 | 1.63E-05 |
| AGAP2-AS1 | EPHX2 | protein_coding | -0.62 | 1.76E-05 |
| AGAP2-AS1 | AIG1 | protein_coding | -0.58 | 6.29E-05 |
| AGAP2-AS1 | SLC2A4 | protein_coding | -0.57 | 9.31E-05 |
| AGAP2-AS1 | CLBA1 | protein_coding | 0.53 | 0.000365857 |
| AGAP2-AS1 | ORMDL3 | protein_coding | -0.58 | 8.14E-05 |
| AGAP2-AS1 | GSTZ1 | protein_coding | -0.52 | 0.000537693 |
| AGAP2-AS1 | SOD1 | protein_coding | -0.58 | 7.91E-05 |
| AGAP2-AS1 | GAS2 | protein_coding | -0.55 | 0.000212936 |
| AGAP2-AS1 | SCX | protein_coding | 0.56 | 0.000144551 |
| AGAP2-AS1 | GNPDA1 | protein_coding | 0.52 | 0.000500619 |
| AGAP2-AS1 | ZXDB | protein_coding | -0.65 | 4.16E-06 |
| AGAP2-AS1 | HRAS | protein_coding | 0.63 | 1.19E-05 |
| AGAP2-AS1 | CROCC2 | protein_coding | -0.70 | 2.74E-07 |
| AGAP2-AS1 | RMI2 | protein_coding | 0.71 | 1.60E-07 |
| AGAP2-AS1 | MBOAT7 | protein_coding | 0.53 | 0.000327747 |
| AGAP2-AS1 | CWC27 | protein_coding | 0.65 | 4.62E-06 |
| AGAP2-AS1 | TH | protein_coding | -0.54 | 0.000238656 |
| AGAP2-AS1 | FHDC1 | protein_coding | 0.54 | 0.000252069 |
| AGAP2-AS1 | MYCL | protein_coding | -0.67 | 2.11E-06 |
| AGAP2-AS1 | CDCA4 | protein_coding | 0.60 | 3.82E-05 |
| AGAP2-AS1 | VSTM5 | protein_coding | 0.52 | 0.000496995 |
| AGAP2-AS1 | TPP2 | protein_coding | -0.53 | 0.000395464 |
| AGAP2-AS1 | ERVFRD-1 | protein_coding | -0.51 | 0.000628117 |
| AGAP2-AS1 | SLC22A25 | protein_coding | -0.59 | 5.37E-05 |
| AGAP2-AS1 | DIO1 | protein_coding | -0.55 | 0.000216093 |
| AGAP2-AS1 | DNAJC10 | protein_coding | 0.55 | 0.000196895 |
| AGAP2-AS1 | DEAF1 | protein_coding | 0.61 | 2.31E-05 |
| AGAP2-AS1 | ENOX1 | protein_coding | 0.51 | 0.000732958 |
| AGAP2-AS1 | FIBCD1 | protein_coding | 0.55 | 0.000229072 |
| AGAP2-AS1 | CKAP2 | protein_coding | 0.54 | 0.000311627 |
| AGAP2-AS1 | ARNT | protein_coding | -0.61 | 2.44E-05 |
| AGAP2-AS1 | GSTA5 | protein_coding | -0.58 | 8.21E-05 |
| AGAP2-AS1 | TCTN1 | protein_coding | 0.50 | 0.000810669 |
| AGAP2-AS1 | ALPL | protein_coding | -0.53 | 0.000405968 |
| AGAP2-AS1 | WIPF2 | protein_coding | 0.56 | 0.00014363 |
| AGAP2-AS1 | BMP8B | protein_coding | 0.54 | 0.00025653 |
| AGAP2-AS1 | NDRG2 | protein_coding | -0.61 | 2.60E-05 |
| AGAP2-AS1 | ADAP1 | protein_coding | 0.54 | 0.000272621 |
| AGAP2-AS1 | CHRNE | protein_coding | -0.54 | 0.000281672 |
| AGAP2-AS1 | TPM4 | protein_coding | 0.60 | 3.54E-05 |
| AGAP2-AS1 | ACOT1 | protein_coding | -0.67 | 1.48E-06 |
| AGAP2-AS1 | SLCO3A1 | protein_coding | 0.61 | 1.93E-05 |
| AGAP2-AS1 | DBT | protein_coding | -0.63 | 1.14E-05 |
| AGAP2-AS1 | PLA2G12B | protein_coding | -0.57 | 8.92E-05 |
| AGAP2-AS1 | NDE1 | protein_coding | 0.63 | 1.13E-05 |
| AGAP2-AS1 | VSNL1 | protein_coding | -0.54 | 0.000272022 |
| AGAP2-AS1 | HMGCLL1 | protein_coding | -0.59 | 4.31E-05 |
| AGAP2-AS1 | ASPHD2 | protein_coding | 0.56 | 0.000144663 |
| AGAP2-AS1 | SPA17 | protein_coding | 0.63 | 9.28E-06 |
| AGAP2-AS1 | SEC24B | protein_coding | -0.64 | 6.17E-06 |
| AGAP2-AS1 | LGALS3 | protein_coding | 0.60 | 3.59E-05 |
| AGAP2-AS1 | CSNK1E | protein_coding | 0.62 | 1.58E-05 |
| AGAP2-AS1 | DNAH7 | protein_coding | 0.61 | 2.83E-05 |
| AGAP2-AS1 | LRRC56 | protein_coding | 0.54 | 0.00029787 |
| AGAP2-AS1 | COL7A1 | protein_coding | 0.68 | 9.42E-07 |
| AGAP2-AS1 | MBIP | protein_coding | -0.53 | 0.000397916 |
| AGAP2-AS1 | ATP2A1 | protein_coding | 0.54 | 0.000258337 |
| AGAP2-AS1 | PEX13 | protein_coding | -0.55 | 0.000191272 |
| AGAP2-AS1 | COL16A1 | protein_coding | 0.55 | 0.000217867 |
| AGAP2-AS1 | JUP | protein_coding | 0.53 | 0.000360496 |
| AGAP2-AS1 | GP5 | protein_coding | -0.64 | 5.76E-06 |
| AGAP2-AS1 | GPC2 | protein_coding | 0.57 | 9.24E-05 |
| AGAP2-AS1 | CROT | protein_coding | -0.53 | 0.000370073 |
| AGAP2-AS1 | MAPK7 | protein_coding | 0.51 | 0.000653535 |
| AGAP2-AS1 | RNF103-CHMP3 | protein_coding | 0.55 | 0.00020117 |
| AGAP2-AS1 | PLCG2 | protein_coding | -0.52 | 0.000577765 |
| AGAP2-AS1 | SPACA9 | protein_coding | 0.56 | 0.000132683 |
| AGAP2-AS1 | TIMM17B | protein_coding | 0.53 | 0.000326865 |
| AGAP2-AS1 | COPG2 | protein_coding | 0.51 | 0.000621183 |
| AGAP2-AS1 | TNFAIP6 | protein_coding | 0.66 | 2.57E-06 |
| AGAP2-AS1 | E2F7 | protein_coding | 0.72 | 1.02E-07 |
| AGAP2-AS1 | LARP6 | protein_coding | 0.74 | 4.38E-08 |
| AGAP2-AS1 | TFR2 | protein_coding | -0.60 | 3.23E-05 |
| AGAP2-AS1 | TFAP4 | protein_coding | 0.56 | 0.000126982 |
| AGAP2-AS1 | MTMR4 | protein_coding | -0.64 | 5.70E-06 |
| AGAP2-AS1 | MYADML2 | protein_coding | 0.50 | 0.000795403 |
| AGAP2-AS1 | CDCA3 | protein_coding | 0.62 | 1.63E-05 |
| AGAP2-AS1 | ENPP5 | protein_coding | 0.68 | 1.15E-06 |
| AGAP2-AS1 | UNC13A | protein_coding | 0.51 | 0.000695874 |
| AGAP2-AS1 | THAP5 | protein_coding | -0.61 | 2.13E-05 |
| AGAP2-AS1 | TRAPPC6B | protein_coding | -0.52 | 0.00052399 |
| AGAP2-AS1 | PPP1R14C | protein_coding | 0.69 | 5.49E-07 |
| AGAP2-AS1 | RNF224 | protein_coding | 0.58 | 7.89E-05 |
| AGAP2-AS1 | PDGFD | protein_coding | 0.60 | 3.49E-05 |
| AGAP2-AS1 | ZNF646 | protein_coding | 0.59 | 5.33E-05 |
| AGAP2-AS1 | PIGS | protein_coding | 0.68 | 9.56E-07 |
| AGAP2-AS1 | KAT7 | protein_coding | 0.59 | 5.69E-05 |
| AGAP2-AS1 | SEL1L3 | protein_coding | 0.66 | 2.92E-06 |
| AGAP2-AS1 | FAM162A | protein_coding | -0.50 | 0.000856889 |
| AGAP2-AS1 | PDZK1IP1 | protein_coding | 0.54 | 0.00024817 |
| AGAP2-AS1 | SPINDOC | protein_coding | 0.76 | 6.30E-09 |
| AGAP2-AS1 | DGKZ | protein_coding | 0.61 | 2.49E-05 |
| AGAP2-AS1 | CAPN7 | protein_coding | -0.54 | 0.000284867 |
| AGAP2-AS1 | IKBKE | protein_coding | 0.54 | 0.000309289 |
| AGAP2-AS1 | DAAM2 | protein_coding | -0.51 | 0.000668489 |
| AGAP2-AS1 | SEPTIN8 | protein_coding | 0.51 | 0.000715025 |
| AGAP2-AS1 | MAFG | protein_coding | 0.63 | 9.18E-06 |
| AGAP2-AS1 | UXS1 | protein_coding | 0.52 | 0.000542573 |
| AGAP2-AS1 | APOM | protein_coding | -0.53 | 0.000320108 |
| AGAP2-AS1 | FATE1 | protein_coding | 0.52 | 0.000479655 |
| AGAP2-AS1 | ILDR1 | protein_coding | 0.51 | 0.000607422 |
| AGAP2-AS1 | TMED3 | protein_coding | 0.70 | 4.45E-07 |
| AGAP2-AS1 | CISH | protein_coding | -0.52 | 0.000443806 |
| AGAP2-AS1 | GPX3 | protein_coding | -0.51 | 0.000611931 |
| AGAP2-AS1 | KIAA0100 | protein_coding | 0.59 | 4.29E-05 |
| AGAP2-AS1 | PLXNA1 | protein_coding | 0.53 | 0.00039509 |
| AGAP2-AS1 | PRSS27 | protein_coding | 0.60 | 3.33E-05 |
| AGAP2-AS1 | JAG2 | protein_coding | 0.53 | 0.000322991 |
| AGAP2-AS1 | OR10J5 | protein_coding | -0.53 | 0.000388961 |
| AGAP2-AS1 | MDFI | protein_coding | 0.57 | 9.41E-05 |
| AGAP2-AS1 | TPM2 | protein_coding | 0.67 | 2.13E-06 |
| AGAP2-AS1 | ITGA9 | protein_coding | -0.52 | 0.000471052 |
| AGAP2-AS1 | CCDC196 | protein_coding | -0.67 | 1.48E-06 |
| AGAP2-AS1 | CTHRC1 | protein_coding | 0.59 | 5.06E-05 |
| AGAP2-AS1 | SPRYD3 | protein_coding | 0.56 | 0.000133696 |
| AGAP2-AS1 | ATG4D | protein_coding | 0.52 | 0.000463604 |
| AGAP2-AS1 | FCN2 | protein_coding | -0.63 | 1.01E-05 |
| AGAP2-AS1 | RANBP3 | protein_coding | 0.55 | 0.000218266 |
| AGAP2-AS1 | CBX2 | protein_coding | 0.56 | 0.000168017 |
| AGAP2-AS1 | GPC4 | protein_coding | 0.63 | 9.39E-06 |
| AGAP2-AS1 | FBXO8 | protein_coding | -0.54 | 0.000303161 |
| AGAP2-AS1 | ERCC6L | protein_coding | 0.64 | 5.48E-06 |
| AGAP2-AS1 | CCDC74B | protein_coding | 0.63 | 1.16E-05 |
| AGAP2-AS1 | PRIM2 | protein_coding | 0.61 | 2.11E-05 |
| AGAP2-AS1 | CRYL1 | protein_coding | -0.67 | 1.84E-06 |
| AGAP2-AS1 | CCDC69 | protein_coding | -0.51 | 0.000674066 |
| AGAP2-AS1 | PLGLB1 | protein_coding | -0.57 | 0.000107375 |
| AGAP2-AS1 | PPEF1 | protein_coding | 0.54 | 0.000237399 |
| AGAP2-AS1 | DUOXA1 | protein_coding | 0.56 | 0.000162817 |
| AGAP2-AS1 | PLK1 | protein_coding | 0.71 | 2.14E-07 |
| AGAP2-AS1 | PRAMEF33 | protein_coding | -0.59 | 5.43E-05 |
| AGAP2-AS1 | FHAD1 | protein_coding | 0.62 | 1.40E-05 |
| AGAP2-AS1 | AC040160.1 | protein_coding | 0.59 | 5.75E-05 |
| AGAP2-AS1 | KLF11 | protein_coding | -0.58 | 7.79E-05 |
| AGAP2-AS1 | KIAA1324 | protein_coding | 0.51 | 0.000670931 |
| AGAP2-AS1 | PCGF2 | protein_coding | 0.74 | 3.49E-08 |
| AGAP2-AS1 | C2CD3 | protein_coding | 0.55 | 0.00018259 |
| AGAP2-AS1 | KCNF1 | protein_coding | 0.57 | 9.38E-05 |
| AGAP2-AS1 | CENPL | protein_coding | 0.59 | 4.70E-05 |
| AGAP2-AS1 | RDH13 | protein_coding | 0.52 | 0.000530889 |
| AGAP2-AS1 | FAXC | protein_coding | 0.56 | 0.000138585 |
| AGAP2-AS1 | HIGD1A | protein_coding | -0.52 | 0.000449157 |
| AGAP2-AS1 | FAM214A | protein_coding | -0.54 | 0.000297951 |
| AGAP2-AS1 | MTHFD1L | protein_coding | 0.65 | 4.90E-06 |
| AGAP2-AS1 | IL17RC | protein_coding | -0.59 | 5.29E-05 |
| AGAP2-AS1 | TNPO2 | protein_coding | 0.58 | 7.84E-05 |
| AGAP2-AS1 | FAM210B | protein_coding | -0.59 | 5.89E-05 |
| AGAP2-AS1 | PTPN5 | protein_coding | 0.51 | 0.000624073 |
| AGAP2-AS1 | G6PD | protein_coding | 0.64 | 7.16E-06 |
| AGAP2-AS1 | GLA | protein_coding | 0.66 | 3.43E-06 |
| AGAP2-AS1 | CDH24 | protein_coding | 0.66 | 2.18E-06 |
| AGAP2-AS1 | TMSB10 | protein_coding | 0.65 | 4.88E-06 |
| AGAP2-AS1 | KIF4A | protein_coding | 0.72 | 8.83E-08 |
| AGAP2-AS1 | ASL | protein_coding | -0.53 | 0.000334995 |
| AGAP2-AS1 | PTHLH | protein_coding | 0.67 | 2.01E-06 |
| AGAP2-AS1 | FOS | protein_coding | -0.53 | 0.000334099 |
| AGAP2-AS1 | TTC27 | protein_coding | 0.58 | 6.31E-05 |
| AGAP2-AS1 | COL6A6 | protein_coding | -0.56 | 0.000121821 |
| AGAP2-AS1 | PMEPA1 | protein_coding | 0.70 | 2.79E-07 |
| AGAP2-AS1 | DCDC1 | protein_coding | -0.53 | 0.000407406 |
| AGAP2-AS1 | ZNF740 | protein_coding | 0.54 | 0.000298582 |
| AGAP2-AS1 | SUMO4 | protein_coding | -0.51 | 0.000589831 |
| AGAP2-AS1 | CCDC102A | protein_coding | 0.60 | 2.91E-05 |
| AGAP2-AS1 | LDHC | protein_coding | -0.57 | 0.000113304 |
| AGAP2-AS1 | TBX3 | protein_coding | -0.61 | 2.57E-05 |
| AGAP2-AS1 | GNE | protein_coding | -0.67 | 1.62E-06 |
| AGAP2-AS1 | HCN4 | protein_coding | 0.51 | 0.000618027 |
| AGAP2-AS1 | ITGB4 | protein_coding | 0.66 | 3.02E-06 |
| AGAP2-AS1 | XXYLT1 | protein_coding | 0.72 | 1.08E-07 |
| AGAP2-AS1 | RTL8A | protein_coding | 0.72 | 8.92E-08 |
| AGAP2-AS1 | APLP1 | protein_coding | 0.72 | 1.17E-07 |
| AGAP2-AS1 | KLC2 | protein_coding | 0.75 | 1.79E-08 |
| AGAP2-AS1 | FETUB | protein_coding | -0.57 | 0.000114871 |
| AGAP2-AS1 | SLC25A15 | protein_coding | -0.68 | 9.01E-07 |
| AGAP2-AS1 | BHMT2 | protein_coding | -0.58 | 6.41E-05 |
| AGAP2-AS1 | HINFP | protein_coding | 0.58 | 6.75E-05 |
| AGAP2-AS1 | SLC37A1 | protein_coding | 0.55 | 0.000185768 |
| AGAP2-AS1 | NSUN6 | protein_coding | -0.65 | 4.90E-06 |
| AGAP2-AS1 | SLC46A3 | protein_coding | -0.62 | 1.52E-05 |
| AGAP2-AS1 | ZWINT | protein_coding | 0.73 | 7.59E-08 |
| AGAP2-AS1 | PCP4L1 | protein_coding | -0.60 | 3.92E-05 |
| AGAP2-AS1 | MT2A | protein_coding | -0.56 | 0.000153297 |
| AGAP2-AS1 | CUX2 | protein_coding | -0.54 | 0.000291038 |
| AGAP2-AS1 | DBNDD1 | protein_coding | 0.54 | 0.0002952 |
| AGAP2-AS1 | SLC25A24 | protein_coding | 0.61 | 2.42E-05 |
| AGAP2-AS1 | CNGA1 | protein_coding | -0.57 | 8.66E-05 |
| AGAP2-AS1 | CYP2C8 | protein_coding | -0.67 | 1.54E-06 |
| AGAP2-AS1 | ATP8A2 | protein_coding | 0.55 | 0.000211273 |
| AGAP2-AS1 | SPIC | protein_coding | -0.53 | 0.000395477 |
| AGAP2-AS1 | KCNK17 | protein_coding | -0.59 | 5.16E-05 |
| AGAP2-AS1 | NOX4 | protein_coding | 0.61 | 2.40E-05 |
| AGAP2-AS1 | TMSB15B | protein_coding | 0.61 | 2.63E-05 |
| AGAP2-AS1 | DPT | protein_coding | -0.59 | 5.68E-05 |
| AGAP2-AS1 | PYCR1 | protein_coding | 0.56 | 0.000150656 |
| AGAP2-AS1 | LRP6 | protein_coding | -0.56 | 0.000133665 |
| AGAP2-AS1 | PRMT1 | protein_coding | 0.64 | 5.77E-06 |
| AGAP2-AS1 | MAPKAPK5 | protein_coding | 0.53 | 0.000336874 |
| AGAP2-AS1 | POLR3F | protein_coding | 0.53 | 0.000418789 |
| AGAP2-AS1 | CFAP61 | protein_coding | 0.52 | 0.000479968 |
| AGAP2-AS1 | TMC4 | protein_coding | 0.65 | 4.70E-06 |
| AGAP2-AS1 | GOLGA4 | protein_coding | -0.54 | 0.000270873 |
| AGAP2-AS1 | CRX | protein_coding | -0.54 | 0.00031547 |
| AGAP2-AS1 | HIBADH | protein_coding | -0.58 | 6.73E-05 |
| AGAP2-AS1 | ARL2 | protein_coding | 0.69 | 7.67E-07 |
| AGAP2-AS1 | MANEA | protein_coding | -0.64 | 6.25E-06 |
| AGAP2-AS1 | PRKRIP1 | protein_coding | 0.59 | 5.33E-05 |
| AGAP2-AS1 | CAV1 | protein_coding | 0.52 | 0.000509413 |
| AGAP2-AS1 | GOLGA7B | protein_coding | 0.58 | 8.06E-05 |
| AGAP2-AS1 | CCT4 | protein_coding | 0.54 | 0.000256022 |
| AGAP2-AS1 | PIGU | protein_coding | 0.60 | 2.90E-05 |
| AGAP2-AS1 | MYO16 | protein_coding | -0.54 | 0.000272183 |
| AGAP2-AS1 | TTPAL | protein_coding | -0.62 | 1.46E-05 |
| AGAP2-AS1 | RNF125 | protein_coding | -0.60 | 2.98E-05 |
| AGAP2-AS1 | RRM1 | protein_coding | 0.60 | 3.18E-05 |
| AGAP2-AS1 | HOMER3 | protein_coding | 0.73 | 6.39E-08 |
| AGAP2-AS1 | FAP | protein_coding | 0.56 | 0.000159547 |
| AGAP2-AS1 | OXT | protein_coding | -0.59 | 4.92E-05 |
| AGAP2-AS1 | TERB2 | protein_coding | -0.71 | 2.35E-07 |
| AGAP2-AS1 | FTSJ3 | protein_coding | 0.52 | 0.000464837 |
| AGAP2-AS1 | ZNF488 | protein_coding | 0.56 | 0.000130651 |
| AGAP2-AS1 | EMC8 | protein_coding | 0.52 | 0.000561913 |
| AGAP2-AS1 | SNRPA | protein_coding | 0.56 | 0.000143568 |
| AGAP2-AS1 | PYCARD | protein_coding | 0.61 | 2.06E-05 |
| AGAP2-AS1 | LCN1 | protein_coding | 0.53 | 0.000334696 |
| AGAP2-AS1 | F2RL3 | protein_coding | 0.50 | 0.000839609 |
| AGAP2-AS1 | SIRT6 | protein_coding | 0.60 | 2.98E-05 |
| AGAP2-AS1 | ULBP1 | protein_coding | 0.60 | 3.41E-05 |
| AGAP2-AS1 | CMTM1 | protein_coding | 0.59 | 5.10E-05 |
| AGAP2-AS1 | ST6GAL1 | protein_coding | -0.71 | 2.15E-07 |
| AGAP2-AS1 | FRMD7 | protein_coding | -0.53 | 0.000397174 |
| AGAP2-AS1 | STARD5 | protein_coding | -0.64 | 5.57E-06 |
| AGAP2-AS1 | CCDC38 | protein_coding | -0.51 | 0.000642562 |
| AGAP2-AS1 | CYP27B1 | protein_coding | 0.57 | 0.000119368 |
| AGAP2-AS1 | MACROH2A2 | protein_coding | 0.54 | 0.000260869 |
| AGAP2-AS1 | RARRES2 | protein_coding | -0.66 | 2.59E-06 |
| AGAP2-AS1 | SNRPD1 | protein_coding | 0.67 | 1.67E-06 |
| AGAP2-AS1 | STPG1 | protein_coding | 0.56 | 0.000161162 |
| AGAP2-AS1 | PCSK1N | protein_coding | 0.63 | 9.29E-06 |
| AGAP2-AS1 | DCAF5 | protein_coding | -0.50 | 0.000799589 |
| AGAP2-AS1 | DIAPH3 | protein_coding | 0.74 | 4.49E-08 |
| AGAP2-AS1 | MAP7D1 | protein_coding | 0.67 | 1.72E-06 |
| AGAP2-AS1 | MEGF9 | protein_coding | -0.51 | 0.000675729 |
| AGAP2-AS1 | MCM6 | protein_coding | 0.72 | 1.41E-07 |
| AGAP2-AS1 | ANGPTL3 | protein_coding | -0.51 | 0.000701511 |
| AGAP2-AS1 | FGFR1OP | protein_coding | -0.64 | 5.57E-06 |
| AGAP2-AS1 | TYRO3 | protein_coding | 0.62 | 1.58E-05 |
| AGAP2-AS1 | CEP55 | protein_coding | 0.74 | 2.80E-08 |
| AGAP2-AS1 | PXDC1 | protein_coding | -0.54 | 0.000242625 |
| AGAP2-AS1 | RBMX | protein_coding | 0.55 | 0.000226643 |
| AGAP2-AS1 | CDCP1 | protein_coding | 0.67 | 1.57E-06 |
| AGAP2-AS1 | AMZ2 | protein_coding | 0.53 | 0.000332465 |
| AGAP2-AS1 | HDGFL3 | protein_coding | 0.68 | 9.90E-07 |
| AGAP2-AS1 | TTYH3 | protein_coding | 0.55 | 0.00020831 |
| AGAP2-AS1 | RALB | protein_coding | 0.52 | 0.000478553 |
| AGAP2-AS1 | GPC5 | protein_coding | -0.68 | 9.93E-07 |
| AGAP2-AS1 | PIMREG | protein_coding | 0.70 | 3.13E-07 |
| AGAP2-AS1 | CCDC40 | protein_coding | 0.50 | 0.000857056 |
| AGAP2-AS1 | KIF20A | protein_coding | 0.64 | 5.46E-06 |
| AGAP2-AS1 | H2AZ1 | protein_coding | 0.62 | 1.65E-05 |
| AGAP2-AS1 | SLC22A15 | protein_coding | 0.60 | 3.59E-05 |
| AGAP2-AS1 | SCP2 | protein_coding | -0.68 | 1.04E-06 |
| AGAP2-AS1 | MT1X | protein_coding | -0.59 | 4.81E-05 |
| AGAP2-AS1 | FAM241B | protein_coding | 0.60 | 3.08E-05 |
| AGAP2-AS1 | ESCO2 | protein_coding | 0.59 | 4.30E-05 |
| AGAP2-AS1 | KNG1 | protein_coding | -0.51 | 0.000692593 |
| AGAP2-AS1 | SPOCD1 | protein_coding | 0.67 | 2.09E-06 |
| AGAP2-AS1 | IL33 | protein_coding | -0.57 | 9.64E-05 |
| AGAP2-AS1 | CYP2B6 | protein_coding | -0.54 | 0.000269149 |
| AGAP2-AS1 | PRAMEF10 | protein_coding | -0.56 | 0.000151532 |
| AGAP2-AS1 | ARHGEF18 | protein_coding | 0.55 | 0.000218924 |
| AGAP2-AS1 | TOLLIP | protein_coding | -0.53 | 0.000333314 |
| AGAP2-AS1 | TMC2 | protein_coding | 0.51 | 0.00059125 |
| AGAP2-AS1 | SMPD4 | protein_coding | 0.55 | 0.000182352 |
| AGAP2-AS1 | S100A6 | protein_coding | 0.58 | 6.29E-05 |
| AGAP2-AS1 | ECHDC3 | protein_coding | -0.54 | 0.000258203 |
| AGAP2-AS1 | ELOVL7 | protein_coding | 0.68 | 8.09E-07 |
| AGAP2-AS1 | FOXF2 | protein_coding | 0.59 | 4.59E-05 |
| AGAP2-AS1 | KIAA0556 | protein_coding | 0.66 | 2.42E-06 |
| AGAP2-AS1 | PDPR | protein_coding | 0.52 | 0.000531928 |
| AGAP2-AS1 | FSD1L | protein_coding | 0.57 | 0.000103141 |
| AGAP2-AS1 | C2 | protein_coding | -0.58 | 8.41E-05 |
| AGAP2-AS1 | CIZ1 | protein_coding | 0.62 | 1.61E-05 |
| AGAP2-AS1 | ITGA2 | protein_coding | 0.54 | 0.000269233 |
| AGAP2-AS1 | SPTLC3 | protein_coding | -0.51 | 0.000715804 |
| AGAP2-AS1 | MYRFL | protein_coding | 0.53 | 0.00033349 |
| AGAP2-AS1 | USF3 | protein_coding | -0.72 | 1.52E-07 |
| AGAP2-AS1 | SNRPD2 | protein_coding | 0.52 | 0.000443566 |
| AGAP2-AS1 | CFP | protein_coding | -0.59 | 5.64E-05 |
| AGAP2-AS1 | ACER1 | protein_coding | -0.52 | 0.000534184 |
| AGAP2-AS1 | ABHD18 | protein_coding | -0.65 | 4.21E-06 |
| AGAP2-AS1 | DNAH17 | protein_coding | 0.59 | 5.59E-05 |
| AGAP2-AS1 | NOP56 | protein_coding | 0.55 | 0.000204828 |
| AGAP2-AS1 | FADS6 | protein_coding | -0.60 | 3.48E-05 |
| AGAP2-AS1 | N4BP2 | protein_coding | -0.57 | 8.71E-05 |
| AGAP2-AS1 | TIGD2 | protein_coding | -0.70 | 4.40E-07 |
| AGAP2-AS1 | KRT19 | protein_coding | 0.66 | 2.66E-06 |
| AGAP2-AS1 | KCTD1 | protein_coding | 0.51 | 0.000672167 |
| AGAP2-AS1 | HIGD1B | protein_coding | 0.54 | 0.000310598 |
| AGAP2-AS1 | PWWP2B | protein_coding | 0.58 | 7.17E-05 |
| AGAP2-AS1 | FA2H | protein_coding | 0.68 | 9.77E-07 |
| AGAP2-AS1 | MEGF10 | protein_coding | -0.68 | 1.32E-06 |
| AGAP2-AS1 | GCHFR | protein_coding | -0.56 | 0.000145423 |
| AGAP2-AS1 | USP39 | protein_coding | 0.67 | 1.75E-06 |
| AGAP2-AS1 | DTL | protein_coding | 0.66 | 2.92E-06 |
| AGAP2-AS1 | EBP | protein_coding | -0.56 | 0.00013466 |
| AGAP2-AS1 | ABCB11 | protein_coding | -0.66 | 2.61E-06 |
| AGAP2-AS1 | AC012651.1 | protein_coding | -0.51 | 0.000673707 |
| AGAP2-AS1 | GNMT | protein_coding | -0.68 | 1.04E-06 |
| AGAP2-AS1 | KHDRBS1 | protein_coding | 0.58 | 7.51E-05 |
| AGAP2-AS1 | RNF185 | protein_coding | -0.64 | 8.10E-06 |
| AGAP2-AS1 | RAB11A | protein_coding | 0.57 | 0.000106246 |
| AGAP2-AS1 | MAP1S | protein_coding | 0.61 | 2.17E-05 |
| AGAP2-AS1 | HSPA12A | protein_coding | 0.67 | 1.48E-06 |
| AGAP2-AS1 | FNIP2 | protein_coding | -0.63 | 8.87E-06 |
| AGAP2-AS1 | CDKN2C | protein_coding | 0.62 | 1.43E-05 |
| AGAP2-AS1 | CFHR3 | protein_coding | -0.52 | 0.000523916 |
| AGAP2-AS1 | C1R | protein_coding | -0.56 | 0.000127634 |
| AGAP2-AS1 | CCT5 | protein_coding | 0.52 | 0.000523107 |
| AGAP2-AS1 | PIP5KL1 | protein_coding | 0.64 | 6.61E-06 |
| AGAP2-AS1 | SUCLG2 | protein_coding | -0.67 | 1.55E-06 |
| AGAP2-AS1 | SPATA33 | protein_coding | 0.69 | 4.88E-07 |
| AGAP2-AS1 | GGA3 | protein_coding | 0.55 | 0.000208299 |
| AGAP2-AS1 | C4orf48 | protein_coding | 0.65 | 4.90E-06 |
| AGAP2-AS1 | LMO1 | protein_coding | 0.56 | 0.000148725 |
| AGAP2-AS1 | NTM | protein_coding | 0.69 | 6.08E-07 |
| AGAP2-AS1 | CALM2 | protein_coding | 0.64 | 5.57E-06 |
| AGAP2-AS1 | DDIAS | protein_coding | 0.56 | 0.000162074 |
| AGAP2-AS1 | SV2A | protein_coding | 0.55 | 0.000229945 |
| AGAP2-AS1 | TTC36 | protein_coding | -0.64 | 8.10E-06 |
| AGAP2-AS1 | UPK2 | protein_coding | 0.50 | 0.000830425 |
| AGAP2-AS1 | SUMO2 | protein_coding | 0.63 | 1.27E-05 |
| AGAP2-AS1 | CDK4 | protein_coding | 0.71 | 2.44E-07 |
| AGAP2-AS1 | GPR160 | protein_coding | 0.56 | 0.000130564 |
| AGAP2-AS1 | HINT3 | protein_coding | -0.57 | 0.000103568 |
| AGAP2-AS1 | TPM1 | protein_coding | 0.58 | 7.87E-05 |
| AGAP2-AS1 | CES2 | protein_coding | -0.57 | 8.84E-05 |
| AGAP2-AS1 | PLP2 | protein_coding | 0.79 | 9.37E-10 |
| AGAP2-AS1 | PRTFDC1 | protein_coding | 0.57 | 9.21E-05 |
| AGAP2-AS1 | SNRPB | protein_coding | 0.68 | 8.02E-07 |
| AGAP2-AS1 | AR | protein_coding | -0.58 | 6.05E-05 |
| AGAP2-AS1 | MYRF | protein_coding | 0.56 | 0.00012313 |
| AGAP2-AS1 | SEZ6L2 | protein_coding | 0.74 | 2.82E-08 |
| AGAP2-AS1 | FAM72B | protein_coding | 0.64 | 7.33E-06 |
| AGAP2-AS1 | PRR11 | protein_coding | 0.72 | 9.32E-08 |
| AGAP2-AS1 | PERM1 | protein_coding | 0.69 | 6.08E-07 |
| AGAP2-AS1 | CD63 | protein_coding | 0.50 | 0.000847467 |
| AGAP2-AS1 | COL4A2 | protein_coding | 0.61 | 2.80E-05 |
| AGAP2-AS1 | GCAT | protein_coding | -0.57 | 0.00010811 |
| AGAP2-AS1 | HOPX | protein_coding | 0.55 | 0.000188869 |
| AGAP2-AS1 | SLC7A11 | protein_coding | 0.59 | 5.00E-05 |
| AGAP2-AS1 | RAE1 | protein_coding | 0.63 | 1.10E-05 |
| AGAP2-AS1 | LCAT | protein_coding | -0.63 | 8.62E-06 |
| AGAP2-AS1 | GPT | protein_coding | -0.53 | 0.00034273 |
| AGAP2-AS1 | USP12 | protein_coding | -0.59 | 5.57E-05 |
| AGAP2-AS1 | BCLAF3 | protein_coding | 0.54 | 0.000247737 |
| AGAP2-AS1 | DDI2 | protein_coding | -0.69 | 7.11E-07 |
| AGAP2-AS1 | UBR3 | protein_coding | -0.64 | 7.95E-06 |
| AGAP2-AS1 | CD14 | protein_coding | -0.55 | 0.000208132 |
| AGAP2-AS1 | CAD | protein_coding | 0.51 | 0.000582608 |
| AGAP2-AS1 | GINS3 | protein_coding | 0.57 | 9.06E-05 |
| AGAP2-AS1 | CFAP36 | protein_coding | 0.68 | 1.07E-06 |
| AGAP2-AS1 | DDX23 | protein_coding | 0.62 | 1.48E-05 |
| AGAP2-AS1 | TOMM70 | protein_coding | -0.50 | 0.000788114 |
| AGAP2-AS1 | EVA1B | protein_coding | 0.51 | 0.000684289 |
| AGAP2-AS1 | MVB12A | protein_coding | 0.52 | 0.000560297 |
| AGAP2-AS1 | TADA1 | protein_coding | -0.53 | 0.000414289 |
| AGAP2-AS1 | RACGAP1 | protein_coding | 0.71 | 2.14E-07 |
| AGAP2-AS1 | TMEM70 | protein_coding | -0.61 | 2.01E-05 |
| AGAP2-AS1 | NIPAL1 | protein_coding | -0.50 | 0.000817602 |
| AGAP2-AS1 | ATP6V1E2 | protein_coding | 0.66 | 3.41E-06 |
| AGAP2-AS1 | TM4SF1 | protein_coding | 0.54 | 0.000238673 |
| AGAP2-AS1 | FANCB | protein_coding | 0.61 | 2.19E-05 |
| AGAP2-AS1 | CDK5 | protein_coding | 0.54 | 0.00024242 |
| AGAP2-AS1 | KREMEN2 | protein_coding | 0.66 | 2.58E-06 |
| AGAP2-AS1 | CSKMT | protein_coding | 0.56 | 0.000134035 |
| AGAP2-AS1 | CDK7 | protein_coding | 0.51 | 0.000645507 |
| AGAP2-AS1 | C14orf180 | protein_coding | -0.59 | 5.40E-05 |
| AGAP2-AS1 | ITPR3 | protein_coding | 0.65 | 4.35E-06 |
| AGAP2-AS1 | MSH2 | protein_coding | 0.63 | 8.60E-06 |
| AGAP2-AS1 | CLPX | protein_coding | -0.59 | 4.74E-05 |
| AGAP2-AS1 | MRPL10 | protein_coding | 0.65 | 5.26E-06 |
| AGAP2-AS1 | CAPZA3 | protein_coding | -0.51 | 0.000590083 |
| AGAP2-AS1 | ZNF446 | protein_coding | 0.63 | 1.26E-05 |
| AGAP2-AS1 | GOLM1 | protein_coding | 0.60 | 3.95E-05 |
| AGAP2-AS1 | NOL4 | protein_coding | -0.67 | 1.74E-06 |
| AGAP2-AS1 | CCDC74A | protein_coding | 0.60 | 3.41E-05 |
| AGAP2-AS1 | SLC6A13 | protein_coding | -0.62 | 1.35E-05 |
| AGAP2-AS1 | SLC19A3 | protein_coding | -0.50 | 0.000830473 |
| AGAP2-AS1 | NIPSNAP2 | protein_coding | 0.61 | 2.62E-05 |
| AGAP2-AS1 | TDO2 | protein_coding | -0.62 | 1.41E-05 |
| AGAP2-AS1 | TTLL6 | protein_coding | 0.51 | 0.000664967 |
| AGAP2-AS1 | SPC25 | protein_coding | 0.74 | 3.04E-08 |
| AGAP2-AS1 | TBCB | protein_coding | 0.66 | 2.17E-06 |
| AGAP2-AS1 | TMEM61 | protein_coding | 0.65 | 5.25E-06 |
| AGAP2-AS1 | RALGPS2 | protein_coding | -0.61 | 2.15E-05 |
| AGAP2-AS1 | LRRC3 | protein_coding | -0.63 | 1.15E-05 |
| AGAP2-AS1 | SBSN | protein_coding | 0.50 | 0.000819014 |
| AGAP2-AS1 | F2 | protein_coding | -0.52 | 0.000538206 |
| AGAP2-AS1 | SLC52A3 | protein_coding | 0.59 | 4.31E-05 |
| AGAP2-AS1 | TMEM82 | protein_coding | -0.59 | 5.53E-05 |
| AGAP2-AS1 | PNPLA7 | protein_coding | -0.68 | 1.13E-06 |
| AGAP2-AS1 | CYP2D6 | protein_coding | -0.69 | 5.64E-07 |
| AGAP2-AS1 | LSS | protein_coding | -0.54 | 0.000293499 |
| AGAP2-AS1 | GFM1 | protein_coding | -0.57 | 9.70E-05 |
| AGAP2-AS1 | GULP1 | protein_coding | 0.52 | 0.000463516 |
| AGAP2-AS1 | PRKX | protein_coding | 0.50 | 0.000823106 |
| AGAP2-AS1 | C4B | protein_coding | -0.56 | 0.000150917 |
| AGAP2-AS1 | TM7SF2 | protein_coding | -0.62 | 1.55E-05 |
| AGAP2-AS1 | KCNN2 | protein_coding | -0.65 | 3.52E-06 |
| AGAP2-AS1 | NOCT | protein_coding | -0.61 | 2.05E-05 |
| AGAP2-AS1 | PROSER3 | protein_coding | 0.57 | 0.000106007 |
| AGAP2-AS1 | DNASE1L1 | protein_coding | 0.51 | 0.00075619 |
| AGAP2-AS1 | PPP2R1B | protein_coding | -0.60 | 2.97E-05 |
| AGAP2-AS1 | TAPT1 | protein_coding | -0.68 | 1.26E-06 |
| AGAP2-AS1 | MSI2 | protein_coding | 0.56 | 0.000168107 |
| AGAP2-AS1 | SLC39A13 | protein_coding | 0.65 | 4.52E-06 |
| AGAP2-AS1 | RAC1 | protein_coding | 0.53 | 0.000419837 |
| AGAP2-AS1 | EPM2A | protein_coding | -0.63 | 8.88E-06 |
| AGAP2-AS1 | ATXN7 | protein_coding | -0.53 | 0.000358539 |
| AGAP2-AS1 | TRAPPC3L | protein_coding | -0.51 | 0.000670938 |
| AGAP2-AS1 | MIP | protein_coding | -0.57 | 0.000100947 |
| AGAP2-AS1 | PIGG | protein_coding | 0.52 | 0.000443504 |
| AGAP2-AS1 | ABCA6 | protein_coding | -0.62 | 1.38E-05 |
| AGAP2-AS1 | JADE2 | protein_coding | 0.63 | 1.08E-05 |
| AGAP2-AS1 | MICALL2 | protein_coding | 0.59 | 5.00E-05 |
| AGAP2-AS1 | SFN | protein_coding | 0.52 | 0.000570792 |
| AGAP2-AS1 | MCIDAS | protein_coding | 0.75 | 1.56E-08 |
| AGAP2-AS1 | CDH6 | protein_coding | 0.68 | 1.20E-06 |
| AGAP2-AS1 | ADAMTS13 | protein_coding | -0.63 | 1.07E-05 |
| AGAP2-AS1 | THUMPD2 | protein_coding | 0.60 | 3.29E-05 |
| AGAP2-AS1 | OXLD1 | protein_coding | 0.56 | 0.000136392 |
| AGAP2-AS1 | CD24 | protein_coding | 0.58 | 8.26E-05 |
| AGAP2-AS1 | PPME1 | protein_coding | 0.59 | 4.75E-05 |
| AGAP2-AS1 | GTPBP10 | protein_coding | -0.59 | 5.96E-05 |
| AGAP2-AS1 | CTTNBP2NL | protein_coding | 0.51 | 0.000680118 |
| AGAP2-AS1 | WDR60 | protein_coding | 0.51 | 0.000646657 |
| AGAP2-AS1 | JOSD2 | protein_coding | 0.59 | 5.91E-05 |
| AGAP2-AS1 | ANKRD13D | protein_coding | 0.62 | 1.80E-05 |
| AGAP2-AS1 | PLXNA3 | protein_coding | 0.57 | 0.000107524 |
| AGAP2-AS1 | CNDP1 | protein_coding | -0.69 | 4.92E-07 |
| AGAP2-AS1 | MYCT1 | protein_coding | -0.50 | 0.000787257 |
| AGAP2-AS1 | RDH16 | protein_coding | -0.68 | 1.01E-06 |
| AGAP2-AS1 | TREM2 | protein_coding | 0.67 | 2.12E-06 |
| AGAP2-AS1 | MED22 | protein_coding | 0.61 | 2.24E-05 |
| AGAP2-AS1 | POLR3D | protein_coding | 0.52 | 0.000451922 |
| AGAP2-AS1 | IFI27L2 | protein_coding | 0.65 | 4.80E-06 |
| AGAP2-AS1 | INPP5J | protein_coding | 0.63 | 1.05E-05 |
| AGAP2-AS1 | SDS | protein_coding | -0.59 | 4.67E-05 |
| AGAP2-AS1 | ABHD17C | protein_coding | 0.56 | 0.000144442 |
| AGAP2-AS1 | ASF1B | protein_coding | 0.74 | 2.49E-08 |
| AGAP2-AS1 | ESRP1 | protein_coding | 0.58 | 6.27E-05 |
| AGAP2-AS1 | USP11 | protein_coding | 0.56 | 0.000153559 |
| AGAP2-AS1 | MT1B | protein_coding | -0.53 | 0.000401197 |
| AGAP2-AS1 | CGB7 | protein_coding | 0.53 | 0.000319207 |
| AGAP2-AS1 | PDCD6 | protein_coding | 0.58 | 6.77E-05 |
| AGAP2-AS1 | PRG4 | protein_coding | -0.67 | 1.77E-06 |
| AGAP2-AS1 | INS-IGF2 | protein_coding | -0.70 | 3.76E-07 |
| AGAP2-AS1 | LY6E | protein_coding | -0.67 | 1.40E-06 |
| AGAP2-AS1 | LYZL1 | protein_coding | -0.62 | 1.83E-05 |
| AGAP2-AS1 | SLC38A2 | protein_coding | -0.53 | 0.000318648 |
| AGAP2-AS1 | CS | protein_coding | 0.57 | 0.000111083 |
| AGAP2-AS1 | ATXN2L | protein_coding | 0.59 | 4.56E-05 |
| AGAP2-AS1 | OLA1 | protein_coding | 0.52 | 0.000477146 |
| AGAP2-AS1 | GLRX5 | protein_coding | -0.53 | 0.000362826 |
| AGAP2-AS1 | RABL2A | protein_coding | 0.57 | 0.000105344 |
| AGAP2-AS1 | TFAP2A | protein_coding | 0.51 | 0.000749942 |
| AGAP2-AS1 | PLEKHG2 | protein_coding | 0.58 | 6.95E-05 |
| AGAP2-AS1 | DOK7 | protein_coding | 0.52 | 0.000575796 |
| AGAP2-AS1 | UBE2I | protein_coding | 0.63 | 1.23E-05 |
| AGAP2-AS1 | USB1 | protein_coding | 0.50 | 0.000818697 |
| AGAP2-AS1 | ARHGEF10 | protein_coding | 0.58 | 6.93E-05 |
| AGAP2-AS1 | TLCD4-RWDD3 | protein_coding | -0.52 | 0.000434536 |
| AGAP2-AS1 | MAU2 | protein_coding | 0.55 | 0.000226543 |
| AGAP2-AS1 | SPTLC2 | protein_coding | 0.51 | 0.00058883 |
| AGAP2-AS1 | EDNRB | protein_coding | -0.59 | 5.25E-05 |
| AGAP2-AS1 | MITF | protein_coding | 0.51 | 0.00064323 |
| AGAP2-AS1 | RAB27A | protein_coding | -0.51 | 0.00062545 |
| AGAP2-AS1 | TROAP | protein_coding | 0.67 | 1.74E-06 |
| AGAP2-AS1 | MAFB | protein_coding | -0.52 | 0.000544595 |
| AGAP2-AS1 | MAP3K2 | protein_coding | -0.54 | 0.000256938 |
| AGAP2-AS1 | MYOF | protein_coding | 0.51 | 0.00058648 |
| AGAP2-AS1 | SERPING1 | protein_coding | -0.54 | 0.000298972 |
| AGAP2-AS1 | ARSI | protein_coding | 0.51 | 0.000650632 |
| AGAP2-AS1 | DSCC1 | protein_coding | 0.67 | 2.00E-06 |
| AGAP2-AS1 | FAM227A | protein_coding | 0.57 | 0.00010992 |
| AGAP2-AS1 | AASS | protein_coding | -0.54 | 0.000234201 |
| AGAP2-AS1 | METTL9 | protein_coding | 0.66 | 2.65E-06 |
| AGAP2-AS1 | STRIP2 | protein_coding | 0.53 | 0.000375884 |
| AGAP2-AS1 | RAB33B | protein_coding | -0.56 | 0.00014605 |
| AGAP2-AS1 | CBX5 | protein_coding | 0.52 | 0.000518027 |
| AGAP2-AS1 | CDC25C | protein_coding | 0.66 | 2.17E-06 |
| AGAP2-AS1 | CYP17A1 | protein_coding | -0.61 | 2.75E-05 |
| AGAP2-AS1 | HSD17B7 | protein_coding | -0.55 | 0.000190335 |
| AGAP2-AS1 | TBC1D30 | protein_coding | 0.55 | 0.000190358 |
| AGAP2-AS1 | RBL1 | protein_coding | 0.66 | 2.48E-06 |
| AGAP2-AS1 | SMARCA1 | protein_coding | -0.55 | 0.000232057 |
| AGAP2-AS1 | TSPAN9 | protein_coding | -0.51 | 0.000711869 |
| AGAP2-AS1 | METTL16 | protein_coding | 0.51 | 0.0007143 |
| AGAP2-AS1 | LIN9 | protein_coding | 0.56 | 0.00013068 |
| AGAP2-AS1 | NRL | protein_coding | -0.52 | 0.000474452 |
| AGAP2-AS1 | HORMAD2 | protein_coding | -0.58 | 7.59E-05 |
| AGAP2-AS1 | SEPTIN3 | protein_coding | 0.53 | 0.000386657 |
| AGAP2-AS1 | SLC66A2 | protein_coding | -0.52 | 0.000529168 |
| AGAP2-AS1 | ME3 | protein_coding | 0.63 | 1.25E-05 |
| AGAP2-AS1 | GLYAT | protein_coding | -0.55 | 0.000169157 |
| AGAP2-AS1 | ZNF219 | protein_coding | 0.61 | 2.09E-05 |
| AGAP2-AS1 | RIC8B | protein_coding | 0.52 | 0.000566138 |
| AGAP2-AS1 | TUBG2 | protein_coding | 0.69 | 7.52E-07 |
| AGAP2-AS1 | TEX30 | protein_coding | -0.56 | 0.000165781 |
| AGAP2-AS1 | GET3 | protein_coding | 0.67 | 1.59E-06 |
| AGAP2-AS1 | LIPM | protein_coding | 0.53 | 0.00033889 |
| AGAP2-AS1 | KIFC3 | protein_coding | 0.51 | 0.000754863 |
| AGAP2-AS1 | ACOX2 | protein_coding | -0.62 | 1.64E-05 |
| AGAP2-AS1 | RNF182 | protein_coding | 0.70 | 3.46E-07 |
| AGAP2-AS1 | C12orf75 | protein_coding | 0.77 | 5.36E-09 |
| AGAP2-AS1 | ARRDC2 | protein_coding | 0.63 | 1.11E-05 |
| AGAP2-AS1 | SP6 | protein_coding | 0.56 | 0.000155586 |
| AGAP2-AS1 | CDC14B | protein_coding | -0.60 | 4.11E-05 |
| AGAP2-AS1 | B4GALNT3 | protein_coding | 0.52 | 0.000524667 |
| AGAP2-AS1 | KAT2B | protein_coding | -0.69 | 7.39E-07 |
| AGAP2-AS1 | ACAT1 | protein_coding | -0.56 | 0.000142402 |
| AGAP2-AS1 | MPZL1 | protein_coding | 0.51 | 0.000628565 |
| AGAP2-AS1 | ENTPD2 | protein_coding | 0.51 | 0.000734213 |
| AGAP2-AS1 | PRKACA | protein_coding | -0.56 | 0.000164642 |
| AGAP2-AS1 | C3 | protein_coding | -0.51 | 0.000729794 |
| AGAP2-AS1 | FANCI | protein_coding | 0.77 | 3.22E-09 |
| AGAP2-AS1 | TBC1D16 | protein_coding | 0.57 | 0.0001077 |
| AGAP2-AS1 | DHRS1 | protein_coding | -0.65 | 4.64E-06 |
| AGAP2-AS1 | LDHD | protein_coding | -0.53 | 0.000335105 |
| AGAP2-AS1 | EXOC3L4 | protein_coding | -0.59 | 4.87E-05 |
| AGAP2-AS1 | SORL1 | protein_coding | -0.77 | 5.17E-09 |
| AGAP2-AS1 | NOXO1 | protein_coding | -0.53 | 0.000388995 |
| AGAP2-AS1 | CYP4A11 | protein_coding | -0.55 | 0.000198183 |
| AGAP2-AS1 | ABCF2 | protein_coding | 0.64 | 5.57E-06 |
| AGAP2-AS1 | TAF6 | protein_coding | 0.63 | 1.26E-05 |
| AGAP2-AS1 | RTP3 | protein_coding | -0.63 | 1.17E-05 |
| AGAP2-AS1 | SLC25A48 | protein_coding | -0.56 | 0.000151732 |
| AGAP2-AS1 | ANO1 | protein_coding | -0.52 | 0.000536256 |
| AGAP2-AS1 | STMN1 | protein_coding | 0.66 | 2.50E-06 |
| AGAP2-AS1 | ZWILCH | protein_coding | 0.61 | 2.69E-05 |
| AGAP2-AS1 | MCM8 | protein_coding | 0.63 | 1.26E-05 |
| AGAP2-AS1 | KDM8 | protein_coding | -0.65 | 4.14E-06 |
| AGAP2-AS1 | RMDN1 | protein_coding | -0.53 | 0.000320392 |
| AGAP2-AS1 | SRC | protein_coding | 0.63 | 1.18E-05 |
| AGAP2-AS1 | SLC22A4 | protein_coding | 0.52 | 0.000526908 |
| AGAP2-AS1 | ITSN1 | protein_coding | -0.50 | 0.000783631 |
| AGAP2-AS1 | SCN7A | protein_coding | -0.53 | 0.000326855 |
| AGAP2-AS1 | KLKB1 | protein_coding | -0.59 | 4.50E-05 |
| AGAP2-AS1 | COL1A1 | protein_coding | 0.55 | 0.00018031 |
| AGAP2-AS1 | MSANTD3 | protein_coding | 0.56 | 0.000148982 |
| AGAP2-AS1 | PPP1R3B | protein_coding | -0.65 | 5.33E-06 |
| AGAP2-AS1 | SLC34A1 | protein_coding | -0.66 | 3.00E-06 |
| AGAP2-AS1 | HMGN4 | protein_coding | 0.57 | 9.12E-05 |
| AGAP2-AS1 | SLC35E4 | protein_coding | 0.83 | 1.25E-11 |
| AGAP2-AS1 | GYG2 | protein_coding | -0.65 | 3.98E-06 |
| AGAP2-AS1 | FBF1 | protein_coding | 0.61 | 2.39E-05 |
| AGAP2-AS1 | GLYCTK | protein_coding | -0.65 | 3.83E-06 |
| AGAP2-AS1 | RHEBL1 | protein_coding | 0.50 | 0.000803959 |
| AGAP2-AS1 | SCRN1 | protein_coding | 0.74 | 3.25E-08 |
| AGAP2-AS1 | TPRG1L | protein_coding | -0.61 | 2.29E-05 |
| AGAP2-AS1 | TPGS2 | protein_coding | 0.73 | 5.92E-08 |
| AGAP2-AS1 | CDR2L | protein_coding | 0.61 | 1.92E-05 |
| AGAP2-AS1 | KBTBD7 | protein_coding | -0.66 | 2.90E-06 |
| AGAP2-AS1 | SMKR1 | protein_coding | 0.62 | 1.58E-05 |
| AGAP2-AS1 | NKIRAS2 | protein_coding | 0.59 | 5.92E-05 |
| AGAP2-AS1 | MTFR1 | protein_coding | -0.56 | 0.000147895 |
| AGAP2-AS1 | GMCL2 | protein_coding | -0.58 | 6.90E-05 |
| AGAP2-AS1 | DGUOK | protein_coding | 0.51 | 0.000697327 |
| AGAP2-AS1 | TMEM252 | protein_coding | -0.60 | 2.93E-05 |
| AGAP2-AS1 | TUBB6 | protein_coding | 0.65 | 4.89E-06 |

| **AC138430.1** | | | | |
| --- | --- | --- | --- | --- |
| **lncRNA** | **gene** | **geneType** | **cor** | **pvalue** |
| AC138430.1 | TMEM256 | protein_coding | 0.55 | 0.000172386 |
| AC138430.1 | SPACA6 | protein_coding | -0.59 | 5.64E-05 |
| AC138430.1 | NAGS | protein_coding | 0.79 | 5.70E-10 |
| AC138430.1 | CEACAM6 | protein_coding | -0.53 | 0.000389651 |
| AC138430.1 | SH3BGRL2 | protein_coding | 0.75 | 1.42E-08 |
| AC138430.1 | TRIM24 | protein_coding | 0.67 | 1.77E-06 |
| AC138430.1 | MGST1 | protein_coding | 0.65 | 4.06E-06 |
| AC138430.1 | RAPGEF2 | protein_coding | 0.57 | 9.44E-05 |
| AC138430.1 | NUF2 | protein_coding | -0.70 | 4.48E-07 |
| AC138430.1 | HSDL2 | protein_coding | 0.82 | 6.02E-11 |
| AC138430.1 | FGGY | protein_coding | 0.62 | 1.84E-05 |
| AC138430.1 | ADRA2B | protein_coding | 0.70 | 3.50E-07 |
| AC138430.1 | BNIP3 | protein_coding | 0.75 | 1.72E-08 |
| AC138430.1 | HUNK | protein_coding | -0.57 | 0.000120018 |
| AC138430.1 | KRT80 | protein_coding | -0.50 | 0.0008076 |
| AC138430.1 | IQCB1 | protein_coding | -0.58 | 6.34E-05 |
| AC138430.1 | ZNF692 | protein_coding | -0.60 | 3.92E-05 |
| AC138430.1 | MAGEE1 | protein_coding | -0.64 | 7.60E-06 |
| AC138430.1 | TSLP | protein_coding | 0.58 | 7.98E-05 |
| AC138430.1 | HIP1R | protein_coding | -0.74 | 2.66E-08 |
| AC138430.1 | PRPF38B | protein_coding | -0.55 | 0.000173407 |
| AC138430.1 | CCND2 | protein_coding | -0.64 | 6.67E-06 |
| AC138430.1 | ALDH2 | protein_coding | 0.77 | 5.17E-09 |
| AC138430.1 | HSD17B13 | protein_coding | 0.68 | 1.13E-06 |
| AC138430.1 | CNIH1 | protein_coding | 0.73 | 8.18E-08 |
| AC138430.1 | KLF9 | protein_coding | 0.70 | 4.03E-07 |
| AC138430.1 | AZGP1 | protein_coding | 0.72 | 1.36E-07 |
| AC138430.1 | EPS8L3 | protein_coding | -0.65 | 4.01E-06 |
| AC138430.1 | VKORC1 | protein_coding | 0.61 | 2.01E-05 |
| AC138430.1 | MYEF2 | protein_coding | -0.77 | 3.32E-09 |
| AC138430.1 | CYB5D2 | protein_coding | 0.59 | 5.18E-05 |
| AC138430.1 | GNAI1 | protein_coding | 0.54 | 0.000234611 |
| AC138430.1 | NR1I3 | protein_coding | 0.74 | 2.45E-08 |
| AC138430.1 | SERPINA1 | protein_coding | 0.59 | 4.51E-05 |
| AC138430.1 | FAM53B | protein_coding | -0.58 | 7.09E-05 |
| AC138430.1 | MPST | protein_coding | 0.66 | 2.83E-06 |
| AC138430.1 | NSUN5 | protein_coding | -0.67 | 1.48E-06 |
| AC138430.1 | ABHD11 | protein_coding | -0.62 | 1.33E-05 |
| AC138430.1 | GDF2 | protein_coding | 0.62 | 1.34E-05 |
| AC138430.1 | PADI3 | protein_coding | -0.54 | 0.000285844 |
| AC138430.1 | TMC5 | protein_coding | -0.64 | 8.24E-06 |
| AC138430.1 | UPB1 | protein_coding | 0.72 | 1.55E-07 |
| AC138430.1 | ORC6 | protein_coding | -0.65 | 4.00E-06 |
| AC138430.1 | TP53INP2 | protein_coding | 0.70 | 2.83E-07 |
| AC138430.1 | ALAS1 | protein_coding | 0.78 | 1.79E-09 |
| AC138430.1 | METTL14 | protein_coding | 0.69 | 5.45E-07 |
| AC138430.1 | DCK | protein_coding | -0.63 | 9.64E-06 |
| AC138430.1 | NEK2 | protein_coding | -0.69 | 7.95E-07 |
| AC138430.1 | DAND5 | protein_coding | -0.62 | 1.46E-05 |
| AC138430.1 | RCBTB2 | protein_coding | 0.50 | 0.000809949 |
| AC138430.1 | INSYN2A | protein_coding | -0.51 | 0.000618435 |
| AC138430.1 | ATF7-NPFF | protein_coding | -0.52 | 0.000476023 |
| AC138430.1 | RAP2C | protein_coding | 0.66 | 2.32E-06 |
| AC138430.1 | ELMO1 | protein_coding | 0.55 | 0.000214254 |
| AC138430.1 | PHLDB1 | protein_coding | -0.65 | 5.14E-06 |
| AC138430.1 | ADORA2A | protein_coding | 0.70 | 4.48E-07 |
| AC138430.1 | SLC2A8 | protein_coding | 0.69 | 4.96E-07 |
| AC138430.1 | FSTL5 | protein_coding | 0.54 | 0.000259658 |
| AC138430.1 | NUDCD3 | protein_coding | -0.51 | 0.00066881 |
| AC138430.1 | VXN | protein_coding | 0.62 | 1.75E-05 |
| AC138430.1 | ACTG2 | protein_coding | -0.53 | 0.000372495 |
| AC138430.1 | SLC25A44 | protein_coding | 0.58 | 6.47E-05 |
| AC138430.1 | RHPN1 | protein_coding | -0.75 | 1.43E-08 |
| AC138430.1 | DENND10 | protein_coding | 0.62 | 1.40E-05 |
| AC138430.1 | MLX | protein_coding | 0.63 | 1.12E-05 |
| AC138430.1 | TRPC5 | protein_coding | 0.69 | 6.65E-07 |
| AC138430.1 | PHIP | protein_coding | -0.53 | 0.000402127 |
| AC138430.1 | ALDH1B1 | protein_coding | 0.65 | 4.45E-06 |
| AC138430.1 | RABL6 | protein_coding | -0.63 | 8.89E-06 |
| AC138430.1 | ZNF680 | protein_coding | 0.68 | 1.05E-06 |
| AC138430.1 | NCAM2 | protein_coding | 0.55 | 0.000170828 |
| AC138430.1 | TESK2 | protein_coding | 0.52 | 0.000437721 |
| AC138430.1 | DGKH | protein_coding | -0.54 | 0.000249619 |
| AC138430.1 | GGH | protein_coding | 0.65 | 4.27E-06 |
| AC138430.1 | FBXL4 | protein_coding | 0.63 | 1.16E-05 |
| AC138430.1 | SEMA4D | protein_coding | -0.59 | 4.54E-05 |
| AC138430.1 | DAB1 | protein_coding | 0.69 | 5.39E-07 |
| AC138430.1 | S100PBP | protein_coding | -0.69 | 7.82E-07 |
| AC138430.1 | F11 | protein_coding | 0.79 | 1.13E-09 |
| AC138430.1 | HMGCL | protein_coding | 0.82 | 4.23E-11 |
| AC138430.1 | AFM | protein_coding | 0.67 | 1.40E-06 |
| AC138430.1 | SLC7A9 | protein_coding | 0.63 | 1.24E-05 |
| AC138430.1 | SMAGP | protein_coding | -0.57 | 8.81E-05 |
| AC138430.1 | DRD1 | protein_coding | 0.53 | 0.000384748 |
| AC138430.1 | TRAFD1 | protein_coding | -0.54 | 0.000273073 |
| AC138430.1 | COLEC11 | protein_coding | 0.64 | 5.70E-06 |
| AC138430.1 | NRSN1 | protein_coding | -0.60 | 3.08E-05 |
| AC138430.1 | MFN2 | protein_coding | 0.67 | 1.51E-06 |
| AC138430.1 | DLGAP5 | protein_coding | -0.70 | 4.11E-07 |
| AC138430.1 | PRKAG2 | protein_coding | 0.70 | 4.66E-07 |
| AC138430.1 | OR2H2 | protein_coding | -0.58 | 6.94E-05 |
| AC138430.1 | SEMA6A | protein_coding | -0.59 | 5.35E-05 |
| AC138430.1 | ABHD14B | protein_coding | 0.76 | 8.47E-09 |
| AC138430.1 | TMC7 | protein_coding | -0.54 | 0.000281086 |
| AC138430.1 | PROB1 | protein_coding | -0.51 | 0.000615188 |
| AC138430.1 | RITA1 | protein_coding | 0.52 | 0.000471205 |
| AC138430.1 | RGS10 | protein_coding | -0.54 | 0.000272123 |
| AC138430.1 | UNK | protein_coding | -0.58 | 6.84E-05 |
| AC138430.1 | HSDL1 | protein_coding | -0.53 | 0.000359623 |
| AC138430.1 | GREB1 | protein_coding | 0.59 | 4.81E-05 |
| AC138430.1 | BTD | protein_coding | 0.83 | 2.49E-11 |
| AC138430.1 | PHTF2 | protein_coding | -0.66 | 2.96E-06 |
| AC138430.1 | BMS1 | protein_coding | -0.63 | 1.21E-05 |
| AC138430.1 | DLG3 | protein_coding | -0.77 | 4.37E-09 |
| AC138430.1 | DCTN2 | protein_coding | -0.66 | 3.05E-06 |
| AC138430.1 | ESS2 | protein_coding | -0.63 | 1.22E-05 |
| AC138430.1 | PRPF6 | protein_coding | -0.52 | 0.000511956 |
| AC138430.1 | C12orf49 | protein_coding | -0.74 | 3.09E-08 |
| AC138430.1 | WNT10A | protein_coding | -0.57 | 0.000112634 |
| AC138430.1 | ZNF579 | protein_coding | -0.56 | 0.000160862 |
| AC138430.1 | SIDT2 | protein_coding | 0.62 | 1.46E-05 |
| AC138430.1 | H2BC18 | protein_coding | -0.58 | 6.71E-05 |
| AC138430.1 | MAN2B2 | protein_coding | 0.57 | 0.000100395 |
| AC138430.1 | GAL3ST4 | protein_coding | -0.71 | 2.71E-07 |
| AC138430.1 | TMEM65 | protein_coding | -0.59 | 4.59E-05 |
| AC138430.1 | ZNF84 | protein_coding | -0.54 | 0.000298426 |
| AC138430.1 | ITCH | protein_coding | 0.70 | 4.70E-07 |
| AC138430.1 | ABCD4 | protein_coding | 0.57 | 9.17E-05 |
| AC138430.1 | ARF3 | protein_coding | -0.68 | 1.21E-06 |
| AC138430.1 | TTLL5 | protein_coding | -0.55 | 0.000207469 |
| AC138430.1 | ALMS1 | protein_coding | -0.56 | 0.000160662 |
| AC138430.1 | TM6SF2 | protein_coding | 0.87 | 2.06E-13 |
| AC138430.1 | STARD4 | protein_coding | 0.66 | 3.37E-06 |
| AC138430.1 | ICA1L | protein_coding | -0.66 | 2.20E-06 |
| AC138430.1 | SLC9A3R2 | protein_coding | 0.70 | 3.27E-07 |
| AC138430.1 | CTSL | protein_coding | 0.56 | 0.000150703 |
| AC138430.1 | CLYBL | protein_coding | 0.80 | 4.17E-10 |
| AC138430.1 | BHLHE41 | protein_coding | -0.64 | 5.67E-06 |
| AC138430.1 | TSPAN17 | protein_coding | -0.58 | 6.92E-05 |
| AC138430.1 | CMTM3 | protein_coding | -0.66 | 2.55E-06 |
| AC138430.1 | PTPRM | protein_coding | -0.54 | 0.000309244 |
| AC138430.1 | SURF4 | protein_coding | 0.50 | 0.00077836 |
| AC138430.1 | DZANK1 | protein_coding | -0.66 | 2.66E-06 |
| AC138430.1 | GMIP | protein_coding | -0.76 | 1.10E-08 |
| AC138430.1 | DNM1L | protein_coding | -0.60 | 2.95E-05 |
| AC138430.1 | DPYD | protein_coding | 0.65 | 4.57E-06 |
| AC138430.1 | MAST2 | protein_coding | -0.52 | 0.000475194 |
| AC138430.1 | ORM2 | protein_coding | 0.54 | 0.000263701 |
| AC138430.1 | LFNG | protein_coding | -0.52 | 0.00045943 |
| AC138430.1 | ANKLE2 | protein_coding | -0.58 | 6.08E-05 |
| AC138430.1 | ACTR3 | protein_coding | -0.55 | 0.000184929 |
| AC138430.1 | H3C4 | protein_coding | -0.61 | 2.79E-05 |
| AC138430.1 | CDCA7L | protein_coding | -0.51 | 0.000619383 |
| AC138430.1 | PACRGL | protein_coding | -0.63 | 1.06E-05 |
| AC138430.1 | ANKH | protein_coding | 0.55 | 0.000199763 |
| AC138430.1 | CMTM7 | protein_coding | -0.58 | 6.41E-05 |
| AC138430.1 | IKBKB | protein_coding | -0.59 | 5.13E-05 |
| AC138430.1 | ZNF117 | protein_coding | -0.59 | 5.40E-05 |
| AC138430.1 | SLC25A45 | protein_coding | 0.52 | 0.00055708 |
| AC138430.1 | ORM1 | protein_coding | 0.53 | 0.000355488 |
| AC138430.1 | EMC3 | protein_coding | 0.69 | 6.72E-07 |
| AC138430.1 | ITIH2 | protein_coding | 0.52 | 0.000483986 |
| AC138430.1 | RAB11FIP4 | protein_coding | -0.54 | 0.000250686 |
| AC138430.1 | RREB1 | protein_coding | 0.60 | 4.11E-05 |
| AC138430.1 | SHF | protein_coding | 0.68 | 9.78E-07 |
| AC138430.1 | AKR7A3 | protein_coding | 0.64 | 5.71E-06 |
| AC138430.1 | ZNF320 | protein_coding | -0.57 | 0.000115679 |
| AC138430.1 | HNMT | protein_coding | 0.52 | 0.00057148 |
| AC138430.1 | SIVA1 | protein_coding | 0.57 | 8.78E-05 |
| AC138430.1 | PAGE5 | protein_coding | 0.61 | 2.44E-05 |
| AC138430.1 | TEX22 | protein_coding | -0.58 | 7.17E-05 |
| AC138430.1 | TMEM123 | protein_coding | 0.62 | 1.44E-05 |
| AC138430.1 | ARID3C | protein_coding | 0.77 | 5.17E-09 |
| AC138430.1 | C3orf80 | protein_coding | -0.61 | 2.02E-05 |
| AC138430.1 | DTYMK | protein_coding | -0.50 | 0.000794602 |
| AC138430.1 | CYTH2 | protein_coding | -0.68 | 1.00E-06 |
| AC138430.1 | H2BU1 | protein_coding | -0.62 | 1.82E-05 |
| AC138430.1 | STX17 | protein_coding | 0.54 | 0.000294856 |
| AC138430.1 | MICU1 | protein_coding | 0.67 | 1.43E-06 |
| AC138430.1 | MAPK8 | protein_coding | 0.60 | 3.48E-05 |
| AC138430.1 | RSAD1 | protein_coding | 0.53 | 0.000397731 |
| AC138430.1 | CBR1 | protein_coding | 0.65 | 3.73E-06 |
| AC138430.1 | MT1F | protein_coding | 0.62 | 1.58E-05 |
| AC138430.1 | GLIPR2 | protein_coding | -0.56 | 0.000160979 |
| AC138430.1 | NTHL1 | protein_coding | 0.52 | 0.00044014 |
| AC138430.1 | DPCD | protein_coding | -0.61 | 2.24E-05 |
| AC138430.1 | KLHL15 | protein_coding | 0.73 | 6.96E-08 |
| AC138430.1 | TTBK1 | protein_coding | 0.75 | 1.24E-08 |
| AC138430.1 | GAB2 | protein_coding | -0.58 | 7.72E-05 |
| AC138430.1 | TOP2A | protein_coding | -0.66 | 2.57E-06 |
| AC138430.1 | ITPRID2 | protein_coding | 0.63 | 9.82E-06 |
| AC138430.1 | YBX3 | protein_coding | -0.63 | 8.58E-06 |
| AC138430.1 | GNAT1 | protein_coding | 0.77 | 4.38E-09 |
| AC138430.1 | UGT2B10 | protein_coding | 0.70 | 4.30E-07 |
| AC138430.1 | DKC1 | protein_coding | -0.59 | 5.00E-05 |
| AC138430.1 | YKT6 | protein_coding | -0.64 | 6.45E-06 |
| AC138430.1 | MTHFD1 | protein_coding | 0.77 | 4.16E-09 |
| AC138430.1 | SSB | protein_coding | -0.60 | 3.78E-05 |
| AC138430.1 | CHCHD10 | protein_coding | 0.52 | 0.000519158 |
| AC138430.1 | TGFB2 | protein_coding | -0.55 | 0.000172883 |
| AC138430.1 | ATP2B2 | protein_coding | 0.63 | 1.15E-05 |
| AC138430.1 | MTFR2 | protein_coding | -0.71 | 1.66E-07 |
| AC138430.1 | RACK1 | protein_coding | -0.52 | 0.000490847 |
| AC138430.1 | WDR35 | protein_coding | -0.53 | 0.00033105 |
| AC138430.1 | GOLIM4 | protein_coding | 0.60 | 3.08E-05 |
| AC138430.1 | ACADSB | protein_coding | 0.83 | 2.23E-11 |
| AC138430.1 | RNF145 | protein_coding | -0.51 | 0.000596511 |
| AC138430.1 | LRRN2 | protein_coding | -0.55 | 0.000172309 |
| AC138430.1 | ENO3 | protein_coding | 0.64 | 7.95E-06 |
| AC138430.1 | SLC25A18 | protein_coding | 0.73 | 7.30E-08 |
| AC138430.1 | KMO | protein_coding | 0.67 | 1.75E-06 |
| AC138430.1 | HGF | protein_coding | 0.58 | 7.71E-05 |
| AC138430.1 | SNED1 | protein_coding | 0.57 | 0.000115669 |
| AC138430.1 | DTD1 | protein_coding | -0.57 | 0.00010115 |
| AC138430.1 | NUP93 | protein_coding | -0.65 | 4.29E-06 |
| AC138430.1 | MT1A | protein_coding | 0.53 | 0.00039299 |
| AC138430.1 | CRTC2 | protein_coding | -0.50 | 0.000851102 |
| AC138430.1 | ATP1A1 | protein_coding | -0.58 | 6.27E-05 |
| AC138430.1 | HSF2BP | protein_coding | -0.69 | 6.51E-07 |
| AC138430.1 | RUFY2 | protein_coding | -0.59 | 5.38E-05 |
| AC138430.1 | AMT | protein_coding | 0.70 | 4.21E-07 |
| AC138430.1 | MASP2 | protein_coding | 0.68 | 1.24E-06 |
| AC138430.1 | CYP3A4 | protein_coding | 0.68 | 1.25E-06 |
| AC138430.1 | CEP68 | protein_coding | -0.63 | 1.13E-05 |
| AC138430.1 | NDUFAF1 | protein_coding | 0.73 | 7.65E-08 |
| AC138430.1 | GPRIN1 | protein_coding | -0.71 | 2.54E-07 |
| AC138430.1 | SLC2A10 | protein_coding | 0.53 | 0.000411018 |
| AC138430.1 | TOPBP1 | protein_coding | -0.58 | 6.75E-05 |
| AC138430.1 | RBL2 | protein_coding | 0.58 | 8.19E-05 |
| AC138430.1 | MMAB | protein_coding | 0.69 | 6.94E-07 |
| AC138430.1 | AC002316.1 | protein_coding | -0.63 | 1.03E-05 |
| AC138430.1 | NPHP1 | protein_coding | -0.71 | 2.41E-07 |
| AC138430.1 | SMDT1 | protein_coding | 0.62 | 1.55E-05 |
| AC138430.1 | PTPN13 | protein_coding | -0.64 | 7.72E-06 |
| AC138430.1 | TXNRD2 | protein_coding | 0.65 | 4.92E-06 |
| AC138430.1 | SMYD5 | protein_coding | -0.68 | 9.64E-07 |
| AC138430.1 | FRMD5 | protein_coding | -0.58 | 6.02E-05 |
| AC138430.1 | DSTYK | protein_coding | -0.56 | 0.000168371 |
| AC138430.1 | ZNF14 | protein_coding | -0.64 | 5.51E-06 |
| AC138430.1 | AP3M2 | protein_coding | -0.64 | 5.62E-06 |
| AC138430.1 | PLCB3 | protein_coding | -0.66 | 3.05E-06 |
| AC138430.1 | B4GALT3 | protein_coding | -0.57 | 0.000104173 |
| AC138430.1 | SHPK | protein_coding | 0.76 | 1.12E-08 |
| AC138430.1 | NCOA6 | protein_coding | -0.52 | 0.000532955 |
| AC138430.1 | CCNG1 | protein_coding | 0.56 | 0.000152334 |
| AC138430.1 | RIPPLY1 | protein_coding | 0.78 | 2.11E-09 |
| AC138430.1 | BUB1B | protein_coding | -0.71 | 2.51E-07 |
| AC138430.1 | MYO5C | protein_coding | -0.54 | 0.000244579 |
| AC138430.1 | RNF103 | protein_coding | 0.61 | 2.04E-05 |
| AC138430.1 | IDS | protein_coding | -0.53 | 0.000389372 |
| AC138430.1 | RARG | protein_coding | -0.68 | 9.83E-07 |
| AC138430.1 | C4orf3 | protein_coding | 0.65 | 3.81E-06 |
| AC138430.1 | SIRT5 | protein_coding | 0.60 | 3.48E-05 |
| AC138430.1 | FAM189B | protein_coding | -0.74 | 3.98E-08 |
| AC138430.1 | PRDM11 | protein_coding | -0.57 | 0.000113846 |
| AC138430.1 | CFHR1 | protein_coding | 0.62 | 1.36E-05 |
| AC138430.1 | SLC25A26 | protein_coding | 0.65 | 4.49E-06 |
| AC138430.1 | NTN3 | protein_coding | 0.58 | 7.73E-05 |
| AC138430.1 | ASXL1 | protein_coding | -0.62 | 1.38E-05 |
| AC138430.1 | INTS4 | protein_coding | -0.53 | 0.000375985 |
| AC138430.1 | SLCO1C1 | protein_coding | -0.52 | 0.000537681 |
| AC138430.1 | AGBL5 | protein_coding | -0.51 | 0.00058636 |
| AC138430.1 | NOB1 | protein_coding | -0.53 | 0.000424761 |
| AC138430.1 | IL1RN | protein_coding | 0.61 | 2.25E-05 |
| AC138430.1 | PAOX | protein_coding | 0.61 | 2.64E-05 |
| AC138430.1 | PPIF | protein_coding | 0.60 | 3.09E-05 |
| AC138430.1 | CPT2 | protein_coding | 0.68 | 1.06E-06 |
| AC138430.1 | APPL2 | protein_coding | -0.52 | 0.00055083 |
| AC138430.1 | PITPNM1 | protein_coding | -0.54 | 0.000316561 |
| AC138430.1 | SNX18 | protein_coding | 0.56 | 0.000131531 |
| AC138430.1 | VPS35 | protein_coding | -0.51 | 0.000694718 |
| AC138430.1 | CHTF18 | protein_coding | -0.69 | 6.02E-07 |
| AC138430.1 | CYB561D1 | protein_coding | -0.68 | 8.80E-07 |
| AC138430.1 | ADSS1 | protein_coding | 0.65 | 3.62E-06 |
| AC138430.1 | HEATR4 | protein_coding | 0.51 | 0.000624743 |
| AC138430.1 | PGLYRP2 | protein_coding | 0.68 | 9.74E-07 |
| AC138430.1 | APOBEC3F | protein_coding | -0.57 | 8.69E-05 |
| AC138430.1 | HPD | protein_coding | 0.61 | 2.79E-05 |
| AC138430.1 | TMEM102 | protein_coding | -0.58 | 7.83E-05 |
| AC138430.1 | C16orf87 | protein_coding | 0.74 | 3.79E-08 |
| AC138430.1 | HAO2 | protein_coding | 0.66 | 2.46E-06 |
| AC138430.1 | NUGGC | protein_coding | 0.60 | 4.13E-05 |
| AC138430.1 | MACROH2A1 | protein_coding | -0.66 | 3.18E-06 |
| AC138430.1 | SEC14L3 | protein_coding | 0.71 | 2.05E-07 |
| AC138430.1 | MTCH2 | protein_coding | 0.70 | 3.21E-07 |
| AC138430.1 | KCNJ4 | protein_coding | 0.54 | 0.000308887 |
| AC138430.1 | E2F1 | protein_coding | -0.63 | 1.01E-05 |
| AC138430.1 | COG2 | protein_coding | -0.59 | 4.88E-05 |
| AC138430.1 | ADGRF4 | protein_coding | -0.59 | 4.46E-05 |
| AC138430.1 | RECQL4 | protein_coding | -0.52 | 0.000528764 |
| AC138430.1 | CXorf38 | protein_coding | -0.63 | 8.51E-06 |
| AC138430.1 | STX1A | protein_coding | -0.65 | 3.80E-06 |
| AC138430.1 | DERA | protein_coding | 0.74 | 4.00E-08 |
| AC138430.1 | DCDC2 | protein_coding | -0.55 | 0.000193069 |
| AC138430.1 | NUMB | protein_coding | 0.51 | 0.000669206 |
| AC138430.1 | PRR7 | protein_coding | -0.74 | 4.35E-08 |
| AC138430.1 | HBS1L | protein_coding | 0.61 | 2.27E-05 |
| AC138430.1 | SLCO1B3 | protein_coding | 0.65 | 4.57E-06 |
| AC138430.1 | TMEM53 | protein_coding | 0.72 | 1.23E-07 |
| AC138430.1 | PEX3 | protein_coding | 0.73 | 6.27E-08 |
| AC138430.1 | ERICH1 | protein_coding | -0.59 | 4.79E-05 |
| AC138430.1 | FAM9A | protein_coding | 0.67 | 1.70E-06 |
| AC138430.1 | APOOL | protein_coding | 0.71 | 2.70E-07 |
| AC138430.1 | MRPS31 | protein_coding | 0.74 | 2.95E-08 |
| AC138430.1 | MNS1 | protein_coding | -0.51 | 0.000599959 |
| AC138430.1 | PFDN1 | protein_coding | -0.69 | 7.11E-07 |
| AC138430.1 | ENPP7 | protein_coding | 0.70 | 2.88E-07 |
| AC138430.1 | ACOT12 | protein_coding | 0.68 | 1.12E-06 |
| AC138430.1 | EHHADH | protein_coding | 0.77 | 3.42E-09 |
| AC138430.1 | IKZF5 | protein_coding | 0.53 | 0.000385942 |
| AC138430.1 | SFI1 | protein_coding | -0.70 | 3.02E-07 |
| AC138430.1 | LOXL2 | protein_coding | -0.50 | 0.000840981 |
| AC138430.1 | CLMN | protein_coding | 0.56 | 0.000159083 |
| AC138430.1 | COX7A2 | protein_coding | 0.52 | 0.000523572 |
| AC138430.1 | KCNJ11 | protein_coding | -0.56 | 0.000125482 |
| AC138430.1 | UBXN8 | protein_coding | 0.57 | 0.00011542 |
| AC138430.1 | UBE2Q2 | protein_coding | -0.67 | 2.11E-06 |
| AC138430.1 | KCNQ3 | protein_coding | -0.63 | 1.01E-05 |
| AC138430.1 | SYK | protein_coding | -0.51 | 0.000705777 |
| AC138430.1 | SLC13A3 | protein_coding | 0.58 | 6.32E-05 |
| AC138430.1 | SLC16A3 | protein_coding | -0.63 | 1.11E-05 |
| AC138430.1 | SLC16A13 | protein_coding | 0.77 | 5.86E-09 |
| AC138430.1 | PRKCD | protein_coding | -0.54 | 0.000242864 |
| AC138430.1 | TPX2 | protein_coding | -0.57 | 0.000118341 |
| AC138430.1 | TSC22D2 | protein_coding | 0.56 | 0.000147719 |
| AC138430.1 | ATF5 | protein_coding | 0.76 | 6.25E-09 |
| AC138430.1 | SULT2B1 | protein_coding | -0.52 | 0.00049928 |
| AC138430.1 | CRACR2B | protein_coding | -0.59 | 5.88E-05 |
| AC138430.1 | SARAF | protein_coding | 0.58 | 8.16E-05 |
| AC138430.1 | EPHA1 | protein_coding | 0.50 | 0.000822229 |
| AC138430.1 | CARM1 | protein_coding | -0.53 | 0.000367871 |
| AC138430.1 | LRATD1 | protein_coding | -0.53 | 0.000395651 |
| AC138430.1 | SYPL2 | protein_coding | 0.78 | 2.79E-09 |
| AC138430.1 | MFGE8 | protein_coding | -0.71 | 1.83E-07 |
| AC138430.1 | SLC25A47 | protein_coding | 0.60 | 3.44E-05 |
| AC138430.1 | ABHD13 | protein_coding | 0.58 | 8.08E-05 |
| AC138430.1 | E2F2 | protein_coding | -0.59 | 4.51E-05 |
| AC138430.1 | LDOC1 | protein_coding | -0.69 | 5.42E-07 |
| AC138430.1 | SNX31 | protein_coding | 0.56 | 0.000166797 |
| AC138430.1 | SLC38A4 | protein_coding | 0.71 | 2.16E-07 |
| AC138430.1 | CDC20 | protein_coding | -0.75 | 1.32E-08 |
| AC138430.1 | PACS1 | protein_coding | -0.83 | 1.81E-11 |
| AC138430.1 | LIN52 | protein_coding | 0.59 | 4.99E-05 |
| AC138430.1 | MMGT1 | protein_coding | -0.52 | 0.000526449 |
| AC138430.1 | TESC | protein_coding | -0.72 | 1.28E-07 |
| AC138430.1 | TECTB | protein_coding | 0.75 | 2.18E-08 |
| AC138430.1 | ZNF385B | protein_coding | 0.66 | 2.70E-06 |
| AC138430.1 | TSKU | protein_coding | 0.66 | 3.03E-06 |
| AC138430.1 | C19orf44 | protein_coding | -0.57 | 0.00010381 |
| AC138430.1 | FLNC | protein_coding | -0.53 | 0.000332499 |
| AC138430.1 | SPRED3 | protein_coding | -0.66 | 2.42E-06 |
| AC138430.1 | IBTK | protein_coding | 0.55 | 0.000178175 |
| AC138430.1 | SECISBP2L | protein_coding | 0.53 | 0.000421713 |
| AC138430.1 | PLA2G12A | protein_coding | 0.62 | 1.76E-05 |
| AC138430.1 | ADI1 | protein_coding | 0.84 | 3.96E-12 |
| AC138430.1 | BOK | protein_coding | 0.68 | 1.07E-06 |
| AC138430.1 | SBK3 | protein_coding | -0.54 | 0.000301529 |
| AC138430.1 | EIF5 | protein_coding | 0.69 | 7.18E-07 |
| AC138430.1 | APOBEC3D | protein_coding | -0.53 | 0.000405004 |
| AC138430.1 | MELTF | protein_coding | -0.75 | 1.33E-08 |
| AC138430.1 | HSD17B12 | protein_coding | 0.50 | 0.000852468 |
| AC138430.1 | ZNF738 | protein_coding | -0.64 | 5.78E-06 |
| AC138430.1 | SCRN2 | protein_coding | 0.65 | 4.17E-06 |
| AC138430.1 | RNF130 | protein_coding | 0.63 | 8.94E-06 |
| AC138430.1 | MCC | protein_coding | 0.62 | 1.66E-05 |
| AC138430.1 | CSRNP2 | protein_coding | -0.52 | 0.000473805 |
| AC138430.1 | XKR6 | protein_coding | -0.66 | 2.55E-06 |
| AC138430.1 | VWA8 | protein_coding | 0.73 | 5.76E-08 |
| AC138430.1 | SLCO2B1 | protein_coding | 0.68 | 8.36E-07 |
| AC138430.1 | STX6 | protein_coding | -0.74 | 3.15E-08 |
| AC138430.1 | RAB34 | protein_coding | -0.65 | 4.03E-06 |
| AC138430.1 | ETNK2 | protein_coding | 0.81 | 1.09E-10 |
| AC138430.1 | CCDC9B | protein_coding | -0.55 | 0.000187274 |
| AC138430.1 | ST3GAL6 | protein_coding | 0.68 | 8.80E-07 |
| AC138430.1 | CYP2J2 | protein_coding | 0.68 | 9.26E-07 |
| AC138430.1 | SLC1A5 | protein_coding | -0.74 | 4.46E-08 |
| AC138430.1 | RPL3L | protein_coding | 0.57 | 9.76E-05 |
| AC138430.1 | C6orf132 | protein_coding | -0.62 | 1.41E-05 |
| AC138430.1 | AKR1D1 | protein_coding | 0.65 | 3.97E-06 |
| AC138430.1 | TP53 | protein_coding | -0.52 | 0.000472447 |
| AC138430.1 | ZNF630 | protein_coding | -0.64 | 6.29E-06 |
| AC138430.1 | LHFPL2 | protein_coding | -0.68 | 1.12E-06 |
| AC138430.1 | PCCB | protein_coding | 0.75 | 1.33E-08 |
| AC138430.1 | FAM155A | protein_coding | -0.61 | 2.30E-05 |
| AC138430.1 | UHRF1 | protein_coding | -0.74 | 4.53E-08 |
| AC138430.1 | WDR90 | protein_coding | -0.62 | 1.31E-05 |
| AC138430.1 | ANKRD1 | protein_coding | -0.52 | 0.000452819 |
| AC138430.1 | PSG9 | protein_coding | -0.50 | 0.000777772 |
| AC138430.1 | APOA2 | protein_coding | 0.59 | 4.78E-05 |
| AC138430.1 | MNX1 | protein_coding | -0.74 | 4.58E-08 |
| AC138430.1 | HSD3B2 | protein_coding | 0.70 | 3.60E-07 |
| AC138430.1 | RFWD3 | protein_coding | -0.64 | 6.39E-06 |
| AC138430.1 | PAIP2B | protein_coding | 0.75 | 1.94E-08 |
| AC138430.1 | CHST10 | protein_coding | -0.66 | 2.32E-06 |
| AC138430.1 | DENND4B | protein_coding | -0.54 | 0.00024798 |
| AC138430.1 | CYP2C18 | protein_coding | 0.56 | 0.000133329 |
| AC138430.1 | LIMK2 | protein_coding | -0.53 | 0.000418667 |
| AC138430.1 | WDR75 | protein_coding | -0.62 | 1.82E-05 |
| AC138430.1 | MINDY4 | protein_coding | -0.64 | 7.77E-06 |
| AC138430.1 | EIF3H | protein_coding | -0.60 | 2.89E-05 |
| AC138430.1 | KCTD17 | protein_coding | -0.73 | 4.82E-08 |
| AC138430.1 | AC022966.1 | protein_coding | -0.57 | 9.19E-05 |
| AC138430.1 | ASAH2 | protein_coding | 0.69 | 7.55E-07 |
| AC138430.1 | IPO4 | protein_coding | -0.52 | 0.000461941 |
| AC138430.1 | KIF3C | protein_coding | -0.75 | 1.83E-08 |
| AC138430.1 | ATP10A | protein_coding | -0.55 | 0.000189081 |
| AC138430.1 | SUGCT | protein_coding | 0.57 | 0.000117088 |
| AC138430.1 | NAA40 | protein_coding | -0.68 | 9.14E-07 |
| AC138430.1 | FTSJ1 | protein_coding | -0.70 | 4.07E-07 |
| AC138430.1 | UGT2B7 | protein_coding | 0.64 | 6.06E-06 |
| AC138430.1 | PTK7 | protein_coding | -0.58 | 6.25E-05 |
| AC138430.1 | CCDC34 | protein_coding | -0.68 | 1.31E-06 |
| AC138430.1 | RDH5 | protein_coding | 0.71 | 2.11E-07 |
| AC138430.1 | TCERG1 | protein_coding | -0.70 | 2.77E-07 |
| AC138430.1 | PUS3 | protein_coding | 0.62 | 1.81E-05 |
| AC138430.1 | PTPN3 | protein_coding | 0.55 | 0.000214328 |
| AC138430.1 | FUBP3 | protein_coding | 0.51 | 0.000597953 |
| AC138430.1 | ARMC3 | protein_coding | -0.66 | 3.04E-06 |
| AC138430.1 | COG3 | protein_coding | 0.64 | 7.18E-06 |
| AC138430.1 | TUBGCP3 | protein_coding | 0.52 | 0.000505755 |
| AC138430.1 | CCNF | protein_coding | -0.60 | 3.89E-05 |
| AC138430.1 | CITED2 | protein_coding | 0.66 | 2.35E-06 |
| AC138430.1 | HEPACAM | protein_coding | 0.69 | 7.15E-07 |
| AC138430.1 | PLEC | protein_coding | -0.53 | 0.000419112 |
| AC138430.1 | HACL1 | protein_coding | 0.75 | 2.23E-08 |
| AC138430.1 | LXN | protein_coding | -0.64 | 6.15E-06 |
| AC138430.1 | PCLAF | protein_coding | -0.67 | 1.47E-06 |
| AC138430.1 | IL6R | protein_coding | 0.67 | 1.89E-06 |
| AC138430.1 | ENPP3 | protein_coding | 0.78 | 1.99E-09 |
| AC138430.1 | NHLH1 | protein_coding | -0.61 | 2.52E-05 |
| AC138430.1 | SLC38A1 | protein_coding | -0.82 | 4.19E-11 |
| AC138430.1 | MTSS2 | protein_coding | 0.55 | 0.000203052 |
| AC138430.1 | DNAJC9 | protein_coding | -0.62 | 1.39E-05 |
| AC138430.1 | TCTN2 | protein_coding | -0.78 | 2.36E-09 |
| AC138430.1 | ETFRF1 | protein_coding | 0.81 | 1.98E-10 |
| AC138430.1 | DLG2 | protein_coding | 0.57 | 0.000106925 |
| AC138430.1 | DAPK3 | protein_coding | -0.58 | 7.11E-05 |
| AC138430.1 | RCAN1 | protein_coding | 0.70 | 2.86E-07 |
| AC138430.1 | C1orf198 | protein_coding | -0.56 | 0.000122341 |
| AC138430.1 | TRO | protein_coding | 0.72 | 1.12E-07 |
| AC138430.1 | SEPHS2 | protein_coding | 0.60 | 3.64E-05 |
| AC138430.1 | SRSF5 | protein_coding | 0.65 | 4.52E-06 |
| AC138430.1 | HSD17B6 | protein_coding | 0.69 | 6.24E-07 |
| AC138430.1 | GCDH | protein_coding | 0.85 | 3.64E-12 |
| AC138430.1 | SPEG | protein_coding | -0.75 | 1.67E-08 |
| AC138430.1 | MT-ND2 | protein_coding | 0.70 | 4.53E-07 |
| AC138430.1 | C6orf223 | protein_coding | -0.56 | 0.000136558 |
| AC138430.1 | DRP2 | protein_coding | -0.66 | 2.75E-06 |
| AC138430.1 | PIEZO1 | protein_coding | -0.61 | 2.09E-05 |
| AC138430.1 | C12orf4 | protein_coding | -0.72 | 1.15E-07 |
| AC138430.1 | XYLB | protein_coding | 0.65 | 3.69E-06 |
| AC138430.1 | SUCO | protein_coding | -0.59 | 4.88E-05 |
| AC138430.1 | GPR84 | protein_coding | -0.72 | 9.43E-08 |
| AC138430.1 | POLR3C | protein_coding | -0.60 | 3.54E-05 |
| AC138430.1 | MT-ND1 | protein_coding | 0.70 | 4.14E-07 |
| AC138430.1 | ISM1 | protein_coding | 0.59 | 4.83E-05 |
| AC138430.1 | DNAAF4 | protein_coding | -0.73 | 8.13E-08 |
| AC138430.1 | P2RX4 | protein_coding | -0.54 | 0.000275701 |
| AC138430.1 | TM2D3 | protein_coding | 0.68 | 8.94E-07 |
| AC138430.1 | MFSD2A | protein_coding | 0.65 | 4.78E-06 |
| AC138430.1 | ZC3HAV1L | protein_coding | -0.66 | 2.63E-06 |
| AC138430.1 | ARFGAP1 | protein_coding | -0.53 | 0.000380659 |
| AC138430.1 | LPCAT3 | protein_coding | 0.56 | 0.00012341 |
| AC138430.1 | MAPKBP1 | protein_coding | -0.62 | 1.86E-05 |
| AC138430.1 | NANOS3 | protein_coding | -0.57 | 0.000107996 |
| AC138430.1 | CENPW | protein_coding | -0.56 | 0.000164783 |
| AC138430.1 | MOGAT3 | protein_coding | 0.70 | 4.61E-07 |
| AC138430.1 | GPSM2 | protein_coding | -0.76 | 1.16E-08 |
| AC138430.1 | KCTD2 | protein_coding | -0.65 | 3.80E-06 |
| AC138430.1 | RASD2 | protein_coding | -0.60 | 3.85E-05 |
| AC138430.1 | TGFBR3L | protein_coding | 0.57 | 0.000116485 |
| AC138430.1 | TMEM176B | protein_coding | 0.65 | 4.69E-06 |
| AC138430.1 | CLUAP1 | protein_coding | -0.65 | 3.47E-06 |
| AC138430.1 | RYK | protein_coding | -0.58 | 7.62E-05 |
| AC138430.1 | NDEL1 | protein_coding | 0.52 | 0.00052246 |
| AC138430.1 | C9orf72 | protein_coding | 0.53 | 0.000353974 |
| AC138430.1 | USP54 | protein_coding | -0.52 | 0.000567718 |
| AC138430.1 | DECR2 | protein_coding | 0.67 | 1.91E-06 |
| AC138430.1 | SSTR1 | protein_coding | 0.57 | 0.000109169 |
| AC138430.1 | IGF1R | protein_coding | -0.59 | 5.28E-05 |
| AC138430.1 | DCAF13 | protein_coding | -0.52 | 0.000465754 |
| AC138430.1 | TMEM143 | protein_coding | 0.66 | 2.66E-06 |
| AC138430.1 | SRGAP1 | protein_coding | -0.72 | 1.16E-07 |
| AC138430.1 | RBM8A | protein_coding | -0.52 | 0.000503177 |
| AC138430.1 | ADAMDEC1 | protein_coding | -0.65 | 4.40E-06 |
| AC138430.1 | ITGB1BP1 | protein_coding | -0.60 | 3.86E-05 |
| AC138430.1 | ASB8 | protein_coding | 0.55 | 0.000232335 |
| AC138430.1 | HRH1 | protein_coding | -0.53 | 0.000390361 |
| AC138430.1 | ORC2 | protein_coding | -0.53 | 0.000330216 |
| AC138430.1 | AC024592.3 | protein_coding | 0.54 | 0.000310404 |
| AC138430.1 | SLC22A7 | protein_coding | 0.67 | 1.58E-06 |
| AC138430.1 | CUTC | protein_coding | 0.51 | 0.000688831 |
| AC138430.1 | ARMC2 | protein_coding | -0.52 | 0.000513328 |
| AC138430.1 | FAAP24 | protein_coding | -0.66 | 3.15E-06 |
| AC138430.1 | DNAJC28 | protein_coding | 0.57 | 0.000101251 |
| AC138430.1 | OLFML1 | protein_coding | 0.57 | 8.66E-05 |
| AC138430.1 | TLCD5 | protein_coding | -0.66 | 2.98E-06 |
| AC138430.1 | GPD1 | protein_coding | 0.66 | 2.27E-06 |
| AC138430.1 | LYRM1 | protein_coding | 0.68 | 1.24E-06 |
| AC138430.1 | SVOP | protein_coding | 0.79 | 9.88E-10 |
| AC138430.1 | SPOCK1 | protein_coding | -0.66 | 2.75E-06 |
| AC138430.1 | DCTN5 | protein_coding | -0.71 | 1.55E-07 |
| AC138430.1 | MERTK | protein_coding | 0.55 | 0.000172688 |
| AC138430.1 | ZNF282 | protein_coding | -0.62 | 1.47E-05 |
| AC138430.1 | REPS2 | protein_coding | 0.58 | 6.17E-05 |
| AC138430.1 | IAPP | protein_coding | 0.57 | 0.000113614 |
| AC138430.1 | KNOP1 | protein_coding | -0.73 | 4.77E-08 |
| AC138430.1 | MED17 | protein_coding | -0.54 | 0.00023518 |
| AC138430.1 | RAB9B | protein_coding | -0.58 | 7.09E-05 |
| AC138430.1 | IQSEC1 | protein_coding | 0.52 | 0.000467007 |
| AC138430.1 | ADAM28 | protein_coding | -0.66 | 2.49E-06 |
| AC138430.1 | TTF2 | protein_coding | -0.66 | 3.43E-06 |
| AC138430.1 | AKAP8 | protein_coding | -0.63 | 1.03E-05 |
| AC138430.1 | RASGRF1 | protein_coding | -0.58 | 7.87E-05 |
| AC138430.1 | RBM34 | protein_coding | -0.58 | 6.56E-05 |
| AC138430.1 | ITGA1 | protein_coding | 0.67 | 1.41E-06 |
| AC138430.1 | SETD7 | protein_coding | 0.68 | 9.94E-07 |
| AC138430.1 | IQGAP3 | protein_coding | -0.65 | 4.24E-06 |
| AC138430.1 | CAT | protein_coding | 0.86 | 7.63E-13 |
| AC138430.1 | TKFC | protein_coding | 0.72 | 8.62E-08 |
| AC138430.1 | SYT10 | protein_coding | 0.57 | 9.13E-05 |
| AC138430.1 | COLGALT1 | protein_coding | -0.71 | 1.93E-07 |
| AC138430.1 | DNAJB9 | protein_coding | 0.66 | 3.33E-06 |
| AC138430.1 | P4HTM | protein_coding | -0.51 | 0.000605928 |
| AC138430.1 | CIAO2A | protein_coding | 0.67 | 1.99E-06 |
| AC138430.1 | CFAP73 | protein_coding | -0.56 | 0.000130996 |
| AC138430.1 | HMMR | protein_coding | -0.54 | 0.000264563 |
| AC138430.1 | RPS27A | protein_coding | -0.52 | 0.000567351 |
| AC138430.1 | GSTA2 | protein_coding | 0.66 | 2.75E-06 |
| AC138430.1 | ZNF684 | protein_coding | 0.67 | 1.51E-06 |
| AC138430.1 | PLEKHG5 | protein_coding | -0.52 | 0.000557332 |
| AC138430.1 | HNF4A | protein_coding | 0.62 | 1.90E-05 |
| AC138430.1 | DNAL4 | protein_coding | -0.71 | 1.91E-07 |
| AC138430.1 | MSH3 | protein_coding | 0.60 | 3.07E-05 |
| AC138430.1 | ADGRL2 | protein_coding | 0.51 | 0.000637313 |
| AC138430.1 | AANAT | protein_coding | -0.57 | 8.97E-05 |
| AC138430.1 | FBXL13 | protein_coding | -0.53 | 0.000391813 |
| AC138430.1 | PHF21A | protein_coding | -0.69 | 5.28E-07 |
| AC138430.1 | ZNF711 | protein_coding | -0.69 | 5.65E-07 |
| AC138430.1 | XAGE3 | protein_coding | 0.79 | 5.86E-10 |
| AC138430.1 | MYCBPAP | protein_coding | -0.53 | 0.000367771 |
| AC138430.1 | FECH | protein_coding | 0.67 | 1.82E-06 |
| AC138430.1 | WDR59 | protein_coding | 0.65 | 5.05E-06 |
| AC138430.1 | MRO | protein_coding | 0.71 | 2.20E-07 |
| AC138430.1 | LINC00634 | protein_coding | -0.58 | 7.72E-05 |
| AC138430.1 | SLC31A1 | protein_coding | 0.80 | 4.04E-10 |
| AC138430.1 | ARHGAP22 | protein_coding | -0.71 | 1.96E-07 |
| AC138430.1 | BPIFB2 | protein_coding | 0.60 | 3.42E-05 |
| AC138430.1 | NKAPL | protein_coding | 0.55 | 0.000217245 |
| AC138430.1 | SERPINA11 | protein_coding | 0.62 | 1.46E-05 |
| AC138430.1 | ZNF791 | protein_coding | -0.50 | 0.000789607 |
| AC138430.1 | PRSS51 | protein_coding | 0.52 | 0.000513637 |
| AC138430.1 | CNBP | protein_coding | 0.60 | 3.72E-05 |
| AC138430.1 | CA2 | protein_coding | 0.51 | 0.000720107 |
| AC138430.1 | NTN1 | protein_coding | 0.51 | 0.000649402 |
| AC138430.1 | SLC17A1 | protein_coding | 0.57 | 0.000112127 |
| AC138430.1 | LOXL1 | protein_coding | -0.60 | 2.87E-05 |
| AC138430.1 | ERO1B | protein_coding | 0.59 | 4.60E-05 |
| AC138430.1 | MFN1 | protein_coding | -0.54 | 0.000308318 |
| AC138430.1 | CCS | protein_coding | 0.55 | 0.00022426 |
| AC138430.1 | PDE6B | protein_coding | -0.58 | 6.76E-05 |
| AC138430.1 | XG | protein_coding | 0.57 | 8.83E-05 |
| AC138430.1 | SLC51A | protein_coding | 0.74 | 3.15E-08 |
| AC138430.1 | MAP3K10 | protein_coding | -0.65 | 4.25E-06 |
| AC138430.1 | ISL2 | protein_coding | -0.55 | 0.000229869 |
| AC138430.1 | FMR1NB | protein_coding | 0.64 | 6.19E-06 |
| AC138430.1 | CYP20A1 | protein_coding | 0.61 | 2.67E-05 |
| AC138430.1 | TXN2 | protein_coding | 0.54 | 0.000237311 |
| AC138430.1 | SPIB | protein_coding | -0.51 | 0.000667869 |
| AC138430.1 | MAT1A | protein_coding | 0.60 | 3.76E-05 |
| AC138430.1 | FAM184A | protein_coding | 0.53 | 0.000319602 |
| AC138430.1 | TONSL | protein_coding | -0.62 | 1.54E-05 |
| AC138430.1 | ZNF142 | protein_coding | -0.75 | 1.47E-08 |
| AC138430.1 | SMIM10L2A | protein_coding | -0.60 | 3.37E-05 |
| AC138430.1 | PLPBP | protein_coding | 0.77 | 3.01E-09 |
| AC138430.1 | VIM | protein_coding | -0.72 | 8.71E-08 |
| AC138430.1 | STK17A | protein_coding | -0.57 | 8.77E-05 |
| AC138430.1 | TEX38 | protein_coding | -0.56 | 0.000151808 |
| AC138430.1 | CDH23 | protein_coding | 0.67 | 2.03E-06 |
| AC138430.1 | COL15A1 | protein_coding | -0.64 | 5.73E-06 |
| AC138430.1 | CCDC157 | protein_coding | -0.65 | 4.08E-06 |
| AC138430.1 | COL9A2 | protein_coding | -0.60 | 4.10E-05 |
| AC138430.1 | MRPL34 | protein_coding | 0.64 | 6.45E-06 |
| AC138430.1 | SHKBP1 | protein_coding | -0.63 | 9.16E-06 |
| AC138430.1 | SLC26A2 | protein_coding | -0.64 | 5.56E-06 |
| AC138430.1 | CAMK2B | protein_coding | 0.75 | 1.47E-08 |
| AC138430.1 | ARPC5 | protein_coding | -0.58 | 7.39E-05 |
| AC138430.1 | AKAP1 | protein_coding | 0.64 | 8.35E-06 |
| AC138430.1 | CCDC30 | protein_coding | -0.66 | 2.29E-06 |
| AC138430.1 | PROCA1 | protein_coding | -0.59 | 5.63E-05 |
| AC138430.1 | PLEKHN1 | protein_coding | -0.75 | 2.02E-08 |
| AC138430.1 | PPP1R1C | protein_coding | 0.50 | 0.00081682 |
| AC138430.1 | RABEP2 | protein_coding | -0.65 | 4.59E-06 |
| AC138430.1 | SFR1 | protein_coding | -0.64 | 7.05E-06 |
| AC138430.1 | PID1 | protein_coding | 0.71 | 2.61E-07 |
| AC138430.1 | ACSS1 | protein_coding | -0.51 | 0.000704572 |
| AC138430.1 | ITGAE | protein_coding | -0.52 | 0.000516593 |
| AC138430.1 | FADS1 | protein_coding | 0.53 | 0.000367954 |
| AC138430.1 | CYP7A1 | protein_coding | 0.71 | 1.78E-07 |
| AC138430.1 | AKR1B1 | protein_coding | -0.62 | 1.86E-05 |
| AC138430.1 | ACSM5 | protein_coding | 0.74 | 4.23E-08 |
| AC138430.1 | TUSC3 | protein_coding | -0.61 | 2.08E-05 |
| AC138430.1 | ZFAT | protein_coding | -0.53 | 0.000345296 |
| AC138430.1 | CEP295 | protein_coding | -0.60 | 3.16E-05 |
| AC138430.1 | PCGF6 | protein_coding | 0.70 | 4.59E-07 |
| AC138430.1 | PRR32 | protein_coding | 0.74 | 3.34E-08 |
| AC138430.1 | PSD3 | protein_coding | 0.57 | 0.000116085 |
| AC138430.1 | MTCL1 | protein_coding | -0.66 | 3.11E-06 |
| AC138430.1 | CETN2 | protein_coding | -0.54 | 0.000309785 |
| AC138430.1 | HOXB9 | protein_coding | -0.63 | 1.24E-05 |
| AC138430.1 | UBALD1 | protein_coding | 0.54 | 0.000307675 |
| AC138430.1 | STAU1 | protein_coding | 0.50 | 0.000790605 |
| AC138430.1 | ERLIN1 | protein_coding | 0.79 | 7.05E-10 |
| AC138430.1 | DHRS3 | protein_coding | 0.77 | 4.46E-09 |
| AC138430.1 | NUP107 | protein_coding | -0.71 | 2.12E-07 |
| AC138430.1 | TMOD1 | protein_coding | 0.62 | 1.52E-05 |
| AC138430.1 | SLC12A9 | protein_coding | -0.63 | 1.25E-05 |
| AC138430.1 | BDH1 | protein_coding | 0.68 | 1.02E-06 |
| AC138430.1 | CAPN9 | protein_coding | -0.52 | 0.00048993 |
| AC138430.1 | ADIPOR2 | protein_coding | 0.76 | 7.32E-09 |
| AC138430.1 | MCRS1 | protein_coding | -0.59 | 4.96E-05 |
| AC138430.1 | ZFAND5 | protein_coding | 0.67 | 1.97E-06 |
| AC138430.1 | PML | protein_coding | -0.67 | 1.63E-06 |
| AC138430.1 | LRRC37B | protein_coding | -0.67 | 1.45E-06 |
| AC138430.1 | DYNC2LI1 | protein_coding | -0.57 | 0.000112449 |
| AC138430.1 | RNMT | protein_coding | -0.53 | 0.000375064 |
| AC138430.1 | ZBTB32 | protein_coding | -0.53 | 0.000357142 |
| AC138430.1 | CAPN6 | protein_coding | -0.52 | 0.000554711 |
| AC138430.1 | C8A | protein_coding | 0.62 | 1.43E-05 |
| AC138430.1 | MAPK3 | protein_coding | -0.64 | 7.12E-06 |
| AC138430.1 | ACD | protein_coding | -0.53 | 0.000381853 |
| AC138430.1 | DENND5B | protein_coding | 0.67 | 2.08E-06 |
| AC138430.1 | LYPLA1 | protein_coding | 0.51 | 0.00066548 |
| AC138430.1 | S100A3 | protein_coding | -0.73 | 7.38E-08 |
| AC138430.1 | MDN1 | protein_coding | 0.69 | 6.31E-07 |
| AC138430.1 | GLT1D1 | protein_coding | 0.63 | 9.49E-06 |
| AC138430.1 | LOXL4 | protein_coding | -0.57 | 9.27E-05 |
| AC138430.1 | ALDH3A2 | protein_coding | 0.67 | 1.47E-06 |
| AC138430.1 | VIPR1 | protein_coding | 0.69 | 5.03E-07 |
| AC138430.1 | CCT6A | protein_coding | -0.62 | 1.47E-05 |
| AC138430.1 | RAB19 | protein_coding | -0.54 | 0.00023884 |
| AC138430.1 | GALNT12 | protein_coding | -0.62 | 1.68E-05 |
| AC138430.1 | MAPK13 | protein_coding | -0.68 | 1.01E-06 |
| AC138430.1 | FN3K | protein_coding | 0.65 | 4.46E-06 |
| AC138430.1 | KANSL2 | protein_coding | -0.66 | 2.26E-06 |
| AC138430.1 | PRRC2A | protein_coding | -0.61 | 2.18E-05 |
| AC138430.1 | MSMO1 | protein_coding | 0.80 | 4.41E-10 |
| AC138430.1 | CD80 | protein_coding | -0.61 | 2.21E-05 |
| AC138430.1 | CAPN15 | protein_coding | -0.60 | 4.15E-05 |
| AC138430.1 | HAPLN3 | protein_coding | -0.77 | 5.34E-09 |
| AC138430.1 | SULT1A2 | protein_coding | 0.57 | 0.000102921 |
| AC138430.1 | C15orf65 | protein_coding | -0.63 | 1.02E-05 |
| AC138430.1 | C3orf85 | protein_coding | 0.77 | 3.43E-09 |
| AC138430.1 | CAAP1 | protein_coding | 0.62 | 1.37E-05 |
| AC138430.1 | ATP7B | protein_coding | 0.64 | 7.14E-06 |
| AC138430.1 | ATP13A1 | protein_coding | -0.64 | 8.17E-06 |
| AC138430.1 | AACS | protein_coding | -0.68 | 1.25E-06 |
| AC138430.1 | MT1E | protein_coding | 0.61 | 2.37E-05 |
| AC138430.1 | BTN2A1 | protein_coding | -0.63 | 1.12E-05 |
| AC138430.1 | MMP11 | protein_coding | -0.69 | 5.45E-07 |
| AC138430.1 | NDUFAB1 | protein_coding | 0.51 | 0.00063342 |
| AC138430.1 | PIAS3 | protein_coding | -0.50 | 0.000788453 |
| AC138430.1 | MXI1 | protein_coding | 0.68 | 9.31E-07 |
| AC138430.1 | RPS8 | protein_coding | -0.50 | 0.000783992 |
| AC138430.1 | SASS6 | protein_coding | -0.57 | 0.00010859 |
| AC138430.1 | EXTL1 | protein_coding | -0.60 | 3.23E-05 |
| AC138430.1 | TTLL11 | protein_coding | 0.51 | 0.000698911 |
| AC138430.1 | RAD51D | protein_coding | -0.73 | 8.23E-08 |
| AC138430.1 | CYP3A5 | protein_coding | 0.54 | 0.000303302 |
| AC138430.1 | MND1 | protein_coding | -0.63 | 9.84E-06 |
| AC138430.1 | ABTB2 | protein_coding | 0.52 | 0.000504874 |
| AC138430.1 | TMEM237 | protein_coding | -0.54 | 0.000256298 |
| AC138430.1 | ANLN | protein_coding | -0.66 | 3.22E-06 |
| AC138430.1 | IL1RAP | protein_coding | 0.54 | 0.000303639 |
| AC138430.1 | TUBA1B | protein_coding | -0.53 | 0.000354387 |
| AC138430.1 | MTMR10 | protein_coding | 0.68 | 1.31E-06 |
| AC138430.1 | GPN1 | protein_coding | -0.53 | 0.000377965 |
| AC138430.1 | ZNF335 | protein_coding | -0.54 | 0.000301236 |
| AC138430.1 | SERINC5 | protein_coding | 0.70 | 3.99E-07 |
| AC138430.1 | KLC4 | protein_coding | 0.79 | 1.10E-09 |
| AC138430.1 | CTDNEP1 | protein_coding | -0.63 | 1.13E-05 |
| AC138430.1 | TPD52L2 | protein_coding | -0.69 | 4.97E-07 |
| AC138430.1 | PRR29 | protein_coding | -0.58 | 8.14E-05 |
| AC138430.1 | AMACR | protein_coding | 0.56 | 0.000162475 |
| AC138430.1 | BZW2 | protein_coding | -0.56 | 0.0001338 |
| AC138430.1 | MISP | protein_coding | -0.63 | 8.97E-06 |
| AC138430.1 | CAPS | protein_coding | -0.60 | 3.97E-05 |
| AC138430.1 | TRIM45 | protein_coding | -0.68 | 9.87E-07 |
| AC138430.1 | DGCR6 | protein_coding | 0.57 | 0.000109523 |
| AC138430.1 | SDCCAG8 | protein_coding | -0.61 | 1.97E-05 |
| AC138430.1 | KIF15 | protein_coding | -0.67 | 1.51E-06 |
| AC138430.1 | RABEP1 | protein_coding | 0.62 | 1.76E-05 |
| AC138430.1 | FGFR1 | protein_coding | -0.70 | 3.68E-07 |
| AC138430.1 | MRTFA | protein_coding | -0.76 | 6.62E-09 |
| AC138430.1 | SKIDA1 | protein_coding | 0.76 | 9.19E-09 |
| AC138430.1 | CDC6 | protein_coding | -0.69 | 7.31E-07 |
| AC138430.1 | LPAR2 | protein_coding | -0.81 | 1.36E-10 |
| AC138430.1 | KCTD21 | protein_coding | 0.55 | 0.00019314 |
| AC138430.1 | PLPP3 | protein_coding | 0.56 | 0.000145272 |
| AC138430.1 | PLGLB2 | protein_coding | 0.72 | 1.03E-07 |
| AC138430.1 | C12orf29 | protein_coding | 0.57 | 0.000115228 |
| AC138430.1 | ZSCAN16 | protein_coding | -0.61 | 2.19E-05 |
| AC138430.1 | PDE12 | protein_coding | 0.63 | 1.16E-05 |
| AC138430.1 | ECM2 | protein_coding | 0.73 | 6.87E-08 |
| AC138430.1 | ABCC11 | protein_coding | 0.76 | 7.63E-09 |
| AC138430.1 | RPL22L1 | protein_coding | -0.68 | 1.32E-06 |
| AC138430.1 | ITIH3 | protein_coding | 0.62 | 1.73E-05 |
| AC138430.1 | ITGA2B | protein_coding | -0.54 | 0.00026895 |
| AC138430.1 | RNF24 | protein_coding | -0.70 | 3.38E-07 |
| AC138430.1 | NAGLU | protein_coding | 0.51 | 0.000608685 |
| AC138430.1 | MFSD12 | protein_coding | -0.50 | 0.000834198 |
| AC138430.1 | FNDC5 | protein_coding | 0.78 | 1.51E-09 |
| AC138430.1 | GDI1 | protein_coding | -0.55 | 0.000199248 |
| AC138430.1 | PCK2 | protein_coding | 0.82 | 3.85E-11 |
| AC138430.1 | ADGRE5 | protein_coding | -0.72 | 8.81E-08 |
| AC138430.1 | OSBP2 | protein_coding | -0.71 | 2.04E-07 |
| AC138430.1 | FSCN1 | protein_coding | -0.60 | 3.39E-05 |
| AC138430.1 | AS3MT | protein_coding | 0.64 | 5.58E-06 |
| AC138430.1 | ZCCHC9 | protein_coding | 0.50 | 0.000799978 |
| AC138430.1 | SLC25A52 | protein_coding | 0.68 | 8.41E-07 |
| AC138430.1 | GPR35 | protein_coding | -0.52 | 0.000499719 |
| AC138430.1 | SLC26A3 | protein_coding | 0.54 | 0.000278228 |
| AC138430.1 | ESPL1 | protein_coding | -0.57 | 0.00010716 |
| AC138430.1 | FGA | protein_coding | 0.65 | 5.37E-06 |
| AC138430.1 | NIT2 | protein_coding | 0.62 | 1.28E-05 |
| AC138430.1 | PKP1 | protein_coding | -0.55 | 0.000219932 |
| AC138430.1 | UBE2T | protein_coding | -0.64 | 6.14E-06 |
| AC138430.1 | NUP37 | protein_coding | -0.55 | 0.000174236 |
| AC138430.1 | TAS2R60 | protein_coding | 0.73 | 6.44E-08 |
| AC138430.1 | CCNB2 | protein_coding | -0.76 | 7.01E-09 |
| AC138430.1 | PCOLCE2 | protein_coding | 0.55 | 0.000172727 |
| AC138430.1 | UBE2Z | protein_coding | -0.77 | 2.89E-09 |
| AC138430.1 | ZIC1 | protein_coding | 0.63 | 1.01E-05 |
| AC138430.1 | SEPTIN5 | protein_coding | -0.56 | 0.00012933 |
| AC138430.1 | SLC16A10 | protein_coding | 0.67 | 1.38E-06 |
| AC138430.1 | UNC45A | protein_coding | -0.61 | 2.77E-05 |
| AC138430.1 | CDK16 | protein_coding | -0.72 | 1.21E-07 |
| AC138430.1 | ASB4 | protein_coding | 0.80 | 2.34E-10 |
| AC138430.1 | VANGL2 | protein_coding | -0.57 | 0.00010983 |
| AC138430.1 | SMARCAL1 | protein_coding | -0.75 | 1.74E-08 |
| AC138430.1 | ARHGAP35 | protein_coding | 0.67 | 1.67E-06 |
| AC138430.1 | CRYAA | protein_coding | 0.71 | 1.71E-07 |
| AC138430.1 | WWC3 | protein_coding | -0.53 | 0.000340904 |
| AC138430.1 | ANXA5 | protein_coding | -0.50 | 0.000826107 |
| AC138430.1 | SLC41A2 | protein_coding | 0.66 | 2.79E-06 |
| AC138430.1 | CEBPB | protein_coding | 0.53 | 0.000369573 |
| AC138430.1 | BIK | protein_coding | -0.56 | 0.000127606 |
| AC138430.1 | C1orf100 | protein_coding | -0.55 | 0.000206561 |
| AC138430.1 | TTF1 | protein_coding | -0.51 | 0.000692253 |
| AC138430.1 | UGT1A4 | protein_coding | 0.67 | 1.64E-06 |
| AC138430.1 | ZNF526 | protein_coding | -0.69 | 6.33E-07 |
| AC138430.1 | CALB2 | protein_coding | -0.55 | 0.000183905 |
| AC138430.1 | C3orf52 | protein_coding | -0.73 | 7.32E-08 |
| AC138430.1 | NUP205 | protein_coding | -0.60 | 3.22E-05 |
| AC138430.1 | SAA4 | protein_coding | 0.62 | 1.84E-05 |
| AC138430.1 | PALM2AKAP2 | protein_coding | 0.53 | 0.000428708 |
| AC138430.1 | CHRM2 | protein_coding | 0.65 | 4.29E-06 |
| AC138430.1 | DZIP3 | protein_coding | -0.53 | 0.000340746 |
| AC138430.1 | CFAP221 | protein_coding | -0.62 | 1.67E-05 |
| AC138430.1 | PLAAT2 | protein_coding | 0.61 | 2.58E-05 |
| AC138430.1 | VASH2 | protein_coding | -0.64 | 7.25E-06 |
| AC138430.1 | ELMO3 | protein_coding | -0.62 | 1.79E-05 |
| AC138430.1 | PDE5A | protein_coding | -0.63 | 1.22E-05 |
| AC138430.1 | PPP1R37 | protein_coding | -0.61 | 2.63E-05 |
| AC138430.1 | ACAD8 | protein_coding | 0.78 | 1.72E-09 |
| AC138430.1 | ABHD10 | protein_coding | 0.60 | 4.11E-05 |
| AC138430.1 | OSBP | protein_coding | 0.55 | 0.000189764 |
| AC138430.1 | SPSB2 | protein_coding | -0.61 | 2.11E-05 |
| AC138430.1 | MTRNR2L12 | protein_coding | 0.61 | 2.32E-05 |
| AC138430.1 | KDM3B | protein_coding | -0.52 | 0.000543027 |
| AC138430.1 | CFHR5 | protein_coding | 0.61 | 2.83E-05 |
| AC138430.1 | ARHGAP27 | protein_coding | -0.69 | 7.00E-07 |
| AC138430.1 | SPRYD4 | protein_coding | 0.80 | 3.67E-10 |
| AC138430.1 | DNER | protein_coding | -0.52 | 0.000457292 |
| AC138430.1 | DNAJA2 | protein_coding | 0.60 | 4.10E-05 |
| AC138430.1 | ACSL5 | protein_coding | 0.61 | 2.69E-05 |
| AC138430.1 | PCYT2 | protein_coding | 0.74 | 3.36E-08 |
| AC138430.1 | CCDC191 | protein_coding | -0.79 | 6.17E-10 |
| AC138430.1 | PAN2 | protein_coding | 0.68 | 1.17E-06 |
| AC138430.1 | ARID4A | protein_coding | 0.50 | 0.000811647 |
| AC138430.1 | RER1 | protein_coding | 0.64 | 8.24E-06 |
| AC138430.1 | NAIP | protein_coding | -0.52 | 0.000520633 |
| AC138430.1 | SLC25A30 | protein_coding | 0.53 | 0.000374115 |
| AC138430.1 | NUDT7 | protein_coding | 0.73 | 8.47E-08 |
| AC138430.1 | ERG28 | protein_coding | 0.73 | 7.35E-08 |
| AC138430.1 | SUCLG1 | protein_coding | 0.66 | 2.33E-06 |
| AC138430.1 | C4BPA | protein_coding | 0.63 | 9.76E-06 |
| AC138430.1 | KIF23 | protein_coding | -0.69 | 4.74E-07 |
| AC138430.1 | FAM189A2 | protein_coding | -0.54 | 0.000280426 |
| AC138430.1 | ST13 | protein_coding | 0.57 | 0.000115306 |
| AC138430.1 | GSTK1 | protein_coding | 0.65 | 4.45E-06 |
| AC138430.1 | HPDL | protein_coding | -0.69 | 5.41E-07 |
| AC138430.1 | TIGD1 | protein_coding | -0.69 | 5.71E-07 |
| AC138430.1 | RNF41 | protein_coding | -0.66 | 2.51E-06 |
| AC138430.1 | GOLGA6A | protein_coding | 0.71 | 1.58E-07 |
| AC138430.1 | CHIC1 | protein_coding | -0.61 | 2.42E-05 |
| AC138430.1 | ZNF202 | protein_coding | -0.74 | 3.33E-08 |
| AC138430.1 | OVOL2 | protein_coding | -0.59 | 5.70E-05 |
| AC138430.1 | POLA1 | protein_coding | -0.60 | 3.28E-05 |
| AC138430.1 | MIA2 | protein_coding | 0.67 | 1.33E-06 |
| AC138430.1 | CNOT6 | protein_coding | -0.71 | 2.45E-07 |
| AC138430.1 | CACYBP | protein_coding | -0.55 | 0.000222051 |
| AC138430.1 | NCDN | protein_coding | -0.64 | 6.89E-06 |
| AC138430.1 | CLSPN | protein_coding | -0.56 | 0.000122852 |
| AC138430.1 | IFNGR2 | protein_coding | -0.63 | 1.03E-05 |
| AC138430.1 | GLUD2 | protein_coding | 0.62 | 1.40E-05 |
| AC138430.1 | GLYATL3 | protein_coding | 0.80 | 2.55E-10 |
| AC138430.1 | ANKDD1B | protein_coding | -0.52 | 0.000486594 |
| AC138430.1 | HNRNPUL1 | protein_coding | -0.67 | 1.48E-06 |
| AC138430.1 | SRSF12 | protein_coding | -0.57 | 8.85E-05 |
| AC138430.1 | MMP9 | protein_coding | -0.63 | 1.25E-05 |
| AC138430.1 | CEMIP | protein_coding | -0.62 | 1.35E-05 |
| AC138430.1 | TRPM8 | protein_coding | 0.60 | 3.56E-05 |
| AC138430.1 | CCDC192 | protein_coding | -0.54 | 0.000307334 |
| AC138430.1 | ADAMTS14 | protein_coding | -0.61 | 2.41E-05 |
| AC138430.1 | HSD17B14 | protein_coding | 0.62 | 1.76E-05 |
| AC138430.1 | ARMC6 | protein_coding | 0.68 | 8.66E-07 |
| AC138430.1 | PRELID3A | protein_coding | -0.81 | 1.06E-10 |
| AC138430.1 | TFB2M | protein_coding | 0.51 | 0.00058084 |
| AC138430.1 | CPNE1 | protein_coding | -0.56 | 0.000162023 |
| AC138430.1 | MOCS2 | protein_coding | 0.74 | 2.41E-08 |
| AC138430.1 | CCNB1 | protein_coding | -0.73 | 4.63E-08 |
| AC138430.1 | FOXO6 | protein_coding | -0.53 | 0.000359353 |
| AC138430.1 | ZNHIT1 | protein_coding | 0.57 | 0.000117588 |
| AC138430.1 | KLHDC1 | protein_coding | 0.51 | 0.000661568 |
| AC138430.1 | MRPL39 | protein_coding | 0.66 | 2.33E-06 |
| AC138430.1 | RASGEF1A | protein_coding | -0.52 | 0.00055879 |
| AC138430.1 | RFPL1 | protein_coding | 0.66 | 3.41E-06 |
| AC138430.1 | SEPTIN2 | protein_coding | -0.68 | 9.76E-07 |
| AC138430.1 | SAPCD2 | protein_coding | -0.71 | 1.74E-07 |
| AC138430.1 | FAM155B | protein_coding | -0.59 | 5.17E-05 |
| AC138430.1 | RASL12 | protein_coding | -0.54 | 0.000244198 |
| AC138430.1 | TCOF1 | protein_coding | -0.77 | 4.37E-09 |
| AC138430.1 | PAQR5 | protein_coding | -0.55 | 0.00019784 |
| AC138430.1 | RSPH14 | protein_coding | -0.65 | 4.57E-06 |
| AC138430.1 | DCLRE1B | protein_coding | -0.65 | 4.93E-06 |
| AC138430.1 | ECT2 | protein_coding | -0.71 | 1.73E-07 |
| AC138430.1 | LRRC47 | protein_coding | 0.69 | 7.35E-07 |
| AC138430.1 | GBP7 | protein_coding | 0.67 | 1.43E-06 |
| AC138430.1 | FMNL2 | protein_coding | -0.66 | 2.52E-06 |
| AC138430.1 | NIPSNAP1 | protein_coding | 0.62 | 1.83E-05 |
| AC138430.1 | ZNF182 | protein_coding | -0.56 | 0.000138083 |
| AC138430.1 | USH1C | protein_coding | -0.57 | 9.69E-05 |
| AC138430.1 | IQCK | protein_coding | -0.64 | 6.88E-06 |
| AC138430.1 | LIPJ | protein_coding | 0.60 | 3.60E-05 |
| AC138430.1 | SNRK | protein_coding | 0.54 | 0.000295411 |
| AC138430.1 | GPR132 | protein_coding | -0.56 | 0.000130523 |
| AC138430.1 | CPOX | protein_coding | 0.72 | 1.31E-07 |
| AC138430.1 | DBF4B | protein_coding | -0.59 | 5.08E-05 |
| AC138430.1 | SHMT2 | protein_coding | 0.62 | 1.57E-05 |
| AC138430.1 | ZNF273 | protein_coding | -0.66 | 2.47E-06 |
| AC138430.1 | MAP3K1 | protein_coding | -0.64 | 6.30E-06 |
| AC138430.1 | PARP8 | protein_coding | -0.76 | 7.52E-09 |
| AC138430.1 | FAM160B1 | protein_coding | 0.65 | 4.01E-06 |
| AC138430.1 | NAGA | protein_coding | 0.63 | 1.00E-05 |
| AC138430.1 | SLC39A6 | protein_coding | -0.66 | 2.97E-06 |
| AC138430.1 | DRAXIN | protein_coding | -0.60 | 2.99E-05 |
| AC138430.1 | CPT1A | protein_coding | 0.51 | 0.000676386 |
| AC138430.1 | TTC30B | protein_coding | -0.60 | 3.47E-05 |
| AC138430.1 | ELOVL6 | protein_coding | 0.74 | 3.48E-08 |
| AC138430.1 | LGALS1 | protein_coding | -0.52 | 0.000444464 |
| AC138430.1 | CROCC | protein_coding | -0.63 | 1.11E-05 |
| AC138430.1 | ACYP2 | protein_coding | 0.69 | 7.24E-07 |
| AC138430.1 | HNRNPUL2 | protein_coding | -0.51 | 0.000618944 |
| AC138430.1 | IQANK1 | protein_coding | -0.60 | 3.34E-05 |
| AC138430.1 | PCNX3 | protein_coding | -0.62 | 1.59E-05 |
| AC138430.1 | LRG1 | protein_coding | 0.67 | 2.03E-06 |
| AC138430.1 | ZNF648 | protein_coding | 0.50 | 0.000854292 |
| AC138430.1 | ZDHHC7 | protein_coding | -0.62 | 1.42E-05 |
| AC138430.1 | RASGEF1B | protein_coding | 0.69 | 6.88E-07 |
| AC138430.1 | NCAPD2 | protein_coding | -0.75 | 2.28E-08 |
| AC138430.1 | ZNF875 | protein_coding | -0.55 | 0.000173498 |
| AC138430.1 | KANK1 | protein_coding | 0.56 | 0.000135889 |
| AC138430.1 | HDAC7 | protein_coding | -0.81 | 1.05E-10 |
| AC138430.1 | ARTN | protein_coding | -0.55 | 0.000205225 |
| AC138430.1 | MYO19 | protein_coding | -0.54 | 0.000239911 |
| AC138430.1 | NRG4 | protein_coding | 0.72 | 8.73E-08 |
| AC138430.1 | NKPD1 | protein_coding | -0.54 | 0.000312212 |
| AC138430.1 | RANBP3L | protein_coding | 0.74 | 2.64E-08 |
| AC138430.1 | PGM1 | protein_coding | 0.68 | 9.55E-07 |
| AC138430.1 | SFXN5 | protein_coding | 0.78 | 1.77E-09 |
| AC138430.1 | PRICKLE3 | protein_coding | -0.57 | 0.000119924 |
| AC138430.1 | UBB | protein_coding | 0.81 | 1.81E-10 |
| AC138430.1 | FAM24B | protein_coding | -0.55 | 0.000190898 |
| AC138430.1 | ADAM12 | protein_coding | -0.52 | 0.000460847 |
| AC138430.1 | C18orf54 | protein_coding | -0.58 | 6.40E-05 |
| AC138430.1 | RRS1 | protein_coding | -0.57 | 9.69E-05 |
| AC138430.1 | ARL6IP1 | protein_coding | 0.64 | 5.96E-06 |
| AC138430.1 | AP4M1 | protein_coding | -0.71 | 2.41E-07 |
| AC138430.1 | ARNTL2 | protein_coding | -0.53 | 0.000329665 |
| AC138430.1 | CHSY1 | protein_coding | -0.53 | 0.000333636 |
| AC138430.1 | MRPS25 | protein_coding | 0.59 | 5.47E-05 |
| AC138430.1 | CD101 | protein_coding | -0.50 | 0.000780507 |
| AC138430.1 | ZG16 | protein_coding | 0.69 | 6.76E-07 |
| AC138430.1 | LRRC75A | protein_coding | -0.54 | 0.000268439 |
| AC138430.1 | MOCOS | protein_coding | 0.51 | 0.000628133 |
| AC138430.1 | CAPN2 | protein_coding | -0.63 | 8.57E-06 |
| AC138430.1 | CRHBP | protein_coding | 0.64 | 8.25E-06 |
| AC138430.1 | DNM1 | protein_coding | -0.55 | 0.000189306 |
| AC138430.1 | CRYBG2 | protein_coding | -0.55 | 0.000180315 |
| AC138430.1 | PRR12 | protein_coding | -0.71 | 1.73E-07 |
| AC138430.1 | FTL | protein_coding | 0.59 | 5.44E-05 |
| AC138430.1 | LYPD1 | protein_coding | -0.61 | 2.74E-05 |
| AC138430.1 | TMEM164 | protein_coding | -0.63 | 9.44E-06 |
| AC138430.1 | SEL1L | protein_coding | 0.57 | 0.000106482 |
| AC138430.1 | EXOC6B | protein_coding | -0.58 | 7.37E-05 |
| AC138430.1 | APBA2 | protein_coding | -0.60 | 2.92E-05 |
| AC138430.1 | CISD2 | protein_coding | 0.58 | 6.27E-05 |
| AC138430.1 | ZNF624 | protein_coding | -0.53 | 0.000421744 |
| AC138430.1 | GON4L | protein_coding | -0.53 | 0.000426766 |
| AC138430.1 | TAF4B | protein_coding | -0.65 | 5.27E-06 |
| AC138430.1 | GPR180 | protein_coding | 0.57 | 9.60E-05 |
| AC138430.1 | WDR47 | protein_coding | -0.53 | 0.000336077 |
| AC138430.1 | DIPK2B | protein_coding | -0.54 | 0.000268219 |
| AC138430.1 | GTF3C1 | protein_coding | -0.57 | 0.000116819 |
| AC138430.1 | RILP | protein_coding | 0.69 | 6.84E-07 |
| AC138430.1 | NIT1 | protein_coding | 0.64 | 8.15E-06 |
| AC138430.1 | RHOBTB2 | protein_coding | -0.51 | 0.000758 |
| AC138430.1 | PHYHD1 | protein_coding | 0.68 | 1.17E-06 |
| AC138430.1 | SLC25A4 | protein_coding | 0.65 | 4.45E-06 |
| AC138430.1 | KIAA2012 | protein_coding | 0.73 | 5.03E-08 |
| AC138430.1 | EEF1E1 | protein_coding | -0.70 | 3.93E-07 |
| AC138430.1 | MPC1 | protein_coding | 0.80 | 2.51E-10 |
| AC138430.1 | ASPDH | protein_coding | 0.74 | 2.84E-08 |
| AC138430.1 | CCDC189 | protein_coding | -0.67 | 1.65E-06 |
| AC138430.1 | SUN2 | protein_coding | 0.63 | 9.86E-06 |
| AC138430.1 | CYP2E1 | protein_coding | 0.63 | 1.01E-05 |
| AC138430.1 | PHKA2 | protein_coding | 0.62 | 1.76E-05 |
| AC138430.1 | GPRIN2 | protein_coding | -0.70 | 4.22E-07 |
| AC138430.1 | NSDHL | protein_coding | 0.57 | 0.000100248 |
| AC138430.1 | POLQ | protein_coding | -0.64 | 7.50E-06 |
| AC138430.1 | NLE1 | protein_coding | -0.68 | 1.20E-06 |
| AC138430.1 | NPW | protein_coding | 0.67 | 1.85E-06 |
| AC138430.1 | PGF | protein_coding | -0.50 | 0.000855116 |
| AC138430.1 | ST6GALNAC5 | protein_coding | -0.54 | 0.000291287 |
| AC138430.1 | SDC2 | protein_coding | 0.77 | 3.17E-09 |
| AC138430.1 | OXTR | protein_coding | -0.65 | 4.67E-06 |
| AC138430.1 | KIRREL1 | protein_coding | -0.54 | 0.000239072 |
| AC138430.1 | NUTF2 | protein_coding | -0.51 | 0.00069596 |
| AC138430.1 | RETSAT | protein_coding | 0.75 | 1.86E-08 |
| AC138430.1 | SOWAHA | protein_coding | 0.68 | 1.04E-06 |
| AC138430.1 | GABBR1 | protein_coding | -0.75 | 1.29E-08 |
| AC138430.1 | RNF220 | protein_coding | -0.54 | 0.000242454 |
| AC138430.1 | HCN3 | protein_coding | 0.77 | 3.59E-09 |
| AC138430.1 | RGPD8 | protein_coding | 0.60 | 3.82E-05 |
| AC138430.1 | NCKAP5L | protein_coding | -0.52 | 0.000553415 |
| AC138430.1 | PPM1G | protein_coding | -0.62 | 1.38E-05 |
| AC138430.1 | QDPR | protein_coding | 0.80 | 5.08E-10 |
| AC138430.1 | NAT1 | protein_coding | 0.73 | 5.78E-08 |
| AC138430.1 | RGS17 | protein_coding | -0.66 | 2.44E-06 |
| AC138430.1 | SCUBE1 | protein_coding | 0.71 | 1.95E-07 |
| AC138430.1 | ATP13A2 | protein_coding | -0.73 | 5.00E-08 |
| AC138430.1 | COBLL1 | protein_coding | 0.73 | 7.33E-08 |
| AC138430.1 | ENTPD8 | protein_coding | 0.61 | 2.72E-05 |
| AC138430.1 | APOF | protein_coding | 0.60 | 3.13E-05 |
| AC138430.1 | IFI16 | protein_coding | -0.58 | 7.57E-05 |
| AC138430.1 | PDE4A | protein_coding | -0.66 | 2.20E-06 |
| AC138430.1 | VTA1 | protein_coding | 0.53 | 0.000341841 |
| AC138430.1 | SHLD2 | protein_coding | 0.54 | 0.000260635 |
| AC138430.1 | DTX3 | protein_coding | -0.68 | 1.25E-06 |
| AC138430.1 | KIAA1211L | protein_coding | -0.69 | 5.84E-07 |
| AC138430.1 | FADS2 | protein_coding | 0.63 | 9.15E-06 |
| AC138430.1 | IFT80 | protein_coding | -0.73 | 4.91E-08 |
| AC138430.1 | XRCC4 | protein_coding | -0.62 | 1.71E-05 |
| AC138430.1 | NIPAL3 | protein_coding | -0.50 | 0.000782861 |
| AC138430.1 | NUDT1 | protein_coding | -0.65 | 4.20E-06 |
| AC138430.1 | BLVRA | protein_coding | -0.69 | 5.50E-07 |
| AC138430.1 | UPF3B | protein_coding | -0.51 | 0.000673928 |
| AC138430.1 | FASN | protein_coding | 0.62 | 1.56E-05 |
| AC138430.1 | FTCDNL1 | protein_coding | 0.63 | 1.25E-05 |
| AC138430.1 | IMPG2 | protein_coding | -0.52 | 0.000455936 |
| AC138430.1 | CHRNA6 | protein_coding | -0.53 | 0.000331817 |
| AC138430.1 | ACKR2 | protein_coding | 0.59 | 5.14E-05 |
| AC138430.1 | SPX | protein_coding | 0.61 | 2.03E-05 |
| AC138430.1 | PGM2L1 | protein_coding | -0.62 | 1.67E-05 |
| AC138430.1 | CDC42BPG | protein_coding | -0.68 | 1.22E-06 |
| AC138430.1 | NPM1 | protein_coding | -0.70 | 3.95E-07 |
| AC138430.1 | ADRA2C | protein_coding | -0.69 | 6.20E-07 |
| AC138430.1 | DTNBP1 | protein_coding | -0.64 | 7.36E-06 |
| AC138430.1 | SIRT3 | protein_coding | 0.57 | 8.60E-05 |
| AC138430.1 | NMD3 | protein_coding | 0.51 | 0.000624669 |
| AC138430.1 | NBL1 | protein_coding | -0.55 | 0.000217078 |
| AC138430.1 | C1S | protein_coding | 0.58 | 6.25E-05 |
| AC138430.1 | VWCE | protein_coding | 0.70 | 3.53E-07 |
| AC138430.1 | GALT | protein_coding | 0.72 | 8.93E-08 |
| AC138430.1 | LARP1B | protein_coding | 0.72 | 1.36E-07 |
| AC138430.1 | AC006254.1 | protein_coding | 0.67 | 1.83E-06 |
| AC138430.1 | TESMIN | protein_coding | -0.74 | 3.59E-08 |
| AC138430.1 | TNFAIP8 | protein_coding | -0.65 | 4.89E-06 |
| AC138430.1 | CATSPER1 | protein_coding | -0.55 | 0.000221281 |
| AC138430.1 | FAM8A1 | protein_coding | 0.62 | 1.52E-05 |
| AC138430.1 | ST7 | protein_coding | 0.73 | 8.05E-08 |
| AC138430.1 | ACAA1 | protein_coding | 0.83 | 1.81E-11 |
| AC138430.1 | ACAD11 | protein_coding | 0.74 | 3.97E-08 |
| AC138430.1 | FAM122A | protein_coding | 0.62 | 1.48E-05 |
| AC138430.1 | ARHGAP44 | protein_coding | -0.54 | 0.000306811 |
| AC138430.1 | SPP1 | protein_coding | -0.56 | 0.000156008 |
| AC138430.1 | SHMT1 | protein_coding | 0.80 | 4.84E-10 |
| AC138430.1 | METTL7B | protein_coding | 0.63 | 1.13E-05 |
| AC138430.1 | FXYD5 | protein_coding | -0.69 | 6.10E-07 |
| AC138430.1 | CFAP77 | protein_coding | -0.50 | 0.000792252 |
| AC138430.1 | ARHGAP39 | protein_coding | -0.55 | 0.000216865 |
| AC138430.1 | FBXO48 | protein_coding | 0.62 | 1.42E-05 |
| AC138430.1 | NINJ2 | protein_coding | 0.51 | 0.000737265 |
| AC138430.1 | MYL9 | protein_coding | -0.56 | 0.000124903 |
| AC138430.1 | FZD2 | protein_coding | -0.60 | 3.34E-05 |
| AC138430.1 | DENND6B | protein_coding | -0.72 | 1.26E-07 |
| AC138430.1 | NFIA | protein_coding | 0.60 | 3.21E-05 |
| AC138430.1 | KIF18A | protein_coding | -0.72 | 8.56E-08 |
| AC138430.1 | SLC25A29 | protein_coding | -0.59 | 5.00E-05 |
| AC138430.1 | MLYCD | protein_coding | 0.84 | 5.08E-12 |
| AC138430.1 | TMEM52 | protein_coding | 0.67 | 1.38E-06 |
| AC138430.1 | SELENOO | protein_coding | 0.73 | 5.91E-08 |
| AC138430.1 | PEX11A | protein_coding | 0.84 | 1.02E-11 |
| AC138430.1 | CAMKK1 | protein_coding | -0.58 | 6.74E-05 |
| AC138430.1 | FAM3A | protein_coding | 0.50 | 0.000795738 |
| AC138430.1 | SH3D21 | protein_coding | -0.66 | 2.48E-06 |
| AC138430.1 | BRD9 | protein_coding | -0.57 | 9.08E-05 |
| AC138430.1 | ASCL1 | protein_coding | 0.73 | 6.13E-08 |
| AC138430.1 | CCDC168 | protein_coding | 0.60 | 3.84E-05 |
| AC138430.1 | IGFBP1 | protein_coding | 0.66 | 2.62E-06 |
| AC138430.1 | MAD2L1 | protein_coding | -0.61 | 2.00E-05 |
| AC138430.1 | CYB561 | protein_coding | -0.58 | 7.80E-05 |
| AC138430.1 | NRM | protein_coding | -0.73 | 7.93E-08 |
| AC138430.1 | NRSN2 | protein_coding | -0.69 | 5.22E-07 |
| AC138430.1 | AUTS2 | protein_coding | 0.59 | 4.64E-05 |
| AC138430.1 | ALOX5AP | protein_coding | -0.53 | 0.00034946 |
| AC138430.1 | FBXO7 | protein_coding | 0.70 | 4.42E-07 |
| AC138430.1 | ZNF165 | protein_coding | -0.51 | 0.000735744 |
| AC138430.1 | BCKDHB | protein_coding | 0.82 | 7.59E-11 |
| AC138430.1 | CA5B | protein_coding | -0.59 | 4.58E-05 |
| AC138430.1 | COL18A1 | protein_coding | 0.66 | 3.21E-06 |
| AC138430.1 | KCNJ8 | protein_coding | 0.64 | 5.93E-06 |
| AC138430.1 | LIF | protein_coding | -0.66 | 2.29E-06 |
| AC138430.1 | LMTK3 | protein_coding | -0.63 | 9.79E-06 |
| AC138430.1 | NT5DC2 | protein_coding | -0.72 | 1.49E-07 |
| AC138430.1 | KIAA1958 | protein_coding | 0.60 | 3.88E-05 |
| AC138430.1 | DMWD | protein_coding | -0.70 | 4.15E-07 |
| AC138430.1 | SPAG8 | protein_coding | -0.52 | 0.000536357 |
| AC138430.1 | LRRK1 | protein_coding | -0.57 | 0.000101693 |
| AC138430.1 | EEF1AKMT1 | protein_coding | 0.56 | 0.000125895 |
| AC138430.1 | SULT1B1 | protein_coding | 0.69 | 5.82E-07 |
| AC138430.1 | GRIN1 | protein_coding | -0.56 | 0.000160819 |
| AC138430.1 | TRMT10A | protein_coding | 0.66 | 3.06E-06 |
| AC138430.1 | C20orf204 | protein_coding | -0.59 | 4.46E-05 |
| AC138430.1 | FAM210A | protein_coding | 0.52 | 0.000574859 |
| AC138430.1 | MYO9B | protein_coding | -0.64 | 6.07E-06 |
| AC138430.1 | ARHGEF2 | protein_coding | -0.59 | 5.16E-05 |
| AC138430.1 | PRPSAP1 | protein_coding | 0.65 | 4.31E-06 |
| AC138430.1 | MTHFD2L | protein_coding | 0.57 | 9.30E-05 |
| AC138430.1 | PI15 | protein_coding | -0.67 | 1.47E-06 |
| AC138430.1 | VAX2 | protein_coding | -0.55 | 0.00017501 |
| AC138430.1 | SLITRK3 | protein_coding | 0.63 | 1.13E-05 |
| AC138430.1 | AC021066.1 | protein_coding | -0.65 | 4.73E-06 |
| AC138430.1 | ITFG2 | protein_coding | -0.59 | 4.33E-05 |
| AC138430.1 | MKRN2OS | protein_coding | -0.54 | 0.00025801 |
| AC138430.1 | GGN | protein_coding | -0.60 | 3.03E-05 |
| AC138430.1 | SLC4A3 | protein_coding | -0.68 | 9.96E-07 |
| AC138430.1 | CRLF2 | protein_coding | -0.57 | 0.000120566 |
| AC138430.1 | AKAP8L | protein_coding | -0.51 | 0.000630763 |
| AC138430.1 | CCDC154 | protein_coding | -0.53 | 0.000423705 |
| AC138430.1 | SIPA1L3 | protein_coding | -0.70 | 4.40E-07 |
| AC138430.1 | TMEM125 | protein_coding | -0.52 | 0.000447342 |
| AC138430.1 | MGAT1 | protein_coding | 0.53 | 0.000377492 |
| AC138430.1 | CERS5 | protein_coding | -0.75 | 2.23E-08 |
| AC138430.1 | TRIM17 | protein_coding | -0.56 | 0.000125249 |
| AC138430.1 | COQ8A | protein_coding | 0.66 | 3.38E-06 |
| AC138430.1 | AMFR | protein_coding | 0.59 | 4.20E-05 |
| AC138430.1 | NLRC5 | protein_coding | -0.55 | 0.000207531 |
| AC138430.1 | PSMD6 | protein_coding | 0.59 | 4.71E-05 |
| AC138430.1 | SPG21 | protein_coding | 0.57 | 0.000104298 |
| AC138430.1 | ARMC9 | protein_coding | -0.76 | 1.11E-08 |
| AC138430.1 | VAMP7 | protein_coding | 0.67 | 1.85E-06 |
| AC138430.1 | LYPD5 | protein_coding | -0.57 | 0.00011668 |
| AC138430.1 | RBBP7 | protein_coding | -0.57 | 9.42E-05 |
| AC138430.1 | ARHGEF1 | protein_coding | -0.61 | 2.76E-05 |
| AC138430.1 | TMEM165 | protein_coding | -0.67 | 1.93E-06 |
| AC138430.1 | DLD | protein_coding | 0.57 | 9.48E-05 |
| AC138430.1 | PCTP | protein_coding | 0.77 | 4.78E-09 |
| AC138430.1 | HINT2 | protein_coding | 0.67 | 1.74E-06 |
| AC138430.1 | EPS15L1 | protein_coding | -0.53 | 0.000366034 |
| AC138430.1 | BBOX1 | protein_coding | 0.53 | 0.000345014 |
| AC138430.1 | TYMS | protein_coding | -0.52 | 0.00056222 |
| AC138430.1 | HSH2D | protein_coding | -0.66 | 2.88E-06 |
| AC138430.1 | ADGRG1 | protein_coding | -0.68 | 1.18E-06 |
| AC138430.1 | LEMD2 | protein_coding | -0.54 | 0.000234698 |
| AC138430.1 | SLC30A4 | protein_coding | 0.57 | 0.000105674 |
| AC138430.1 | CHMP4B | protein_coding | -0.58 | 6.32E-05 |
| AC138430.1 | CD1D | protein_coding | 0.54 | 0.000269493 |
| AC138430.1 | PDGFB | protein_coding | -0.57 | 8.73E-05 |
| AC138430.1 | AGRN | protein_coding | -0.51 | 0.000587049 |
| AC138430.1 | PRKAR2A | protein_coding | 0.65 | 5.11E-06 |
| AC138430.1 | CCNE2 | protein_coding | -0.54 | 0.000309229 |
| AC138430.1 | FCGR1A | protein_coding | -0.63 | 8.84E-06 |
| AC138430.1 | C4orf19 | protein_coding | 0.55 | 0.00019228 |
| AC138430.1 | ANKS6 | protein_coding | -0.67 | 1.63E-06 |
| AC138430.1 | PIWIL4 | protein_coding | -0.74 | 2.76E-08 |
| AC138430.1 | NAP1L1 | protein_coding | -0.77 | 3.16E-09 |
| AC138430.1 | MKS1 | protein_coding | -0.59 | 5.81E-05 |
| AC138430.1 | METTL7A | protein_coding | 0.81 | 9.53E-11 |
| AC138430.1 | SART3 | protein_coding | -0.52 | 0.000551366 |
| AC138430.1 | HTR1D | protein_coding | -0.51 | 0.000648358 |
| AC138430.1 | ACADS | protein_coding | 0.73 | 8.38E-08 |
| AC138430.1 | ATIC | protein_coding | -0.67 | 1.34E-06 |
| AC138430.1 | KDELR2 | protein_coding | 0.51 | 0.000671232 |
| AC138430.1 | STEAP3 | protein_coding | 0.67 | 1.71E-06 |
| AC138430.1 | PCLO | protein_coding | -0.52 | 0.00056816 |
| AC138430.1 | APOBEC3G | protein_coding | -0.56 | 0.000131275 |
| AC138430.1 | DGCR6L | protein_coding | 0.68 | 1.06E-06 |
| AC138430.1 | DKKL1 | protein_coding | -0.56 | 0.000162775 |
| AC138430.1 | NPNT | protein_coding | -0.57 | 0.000114697 |
| AC138430.1 | TCP10L | protein_coding | 0.70 | 4.60E-07 |
| AC138430.1 | ZNF184 | protein_coding | -0.52 | 0.000491966 |
| AC138430.1 | F12 | protein_coding | 0.66 | 2.79E-06 |
| AC138430.1 | MSH5 | protein_coding | -0.55 | 0.000173676 |
| AC138430.1 | CASP9 | protein_coding | 0.62 | 1.63E-05 |
| AC138430.1 | SLC17A2 | protein_coding | 0.54 | 0.000302621 |
| AC138430.1 | RTL4 | protein_coding | 0.73 | 4.61E-08 |
| AC138430.1 | ARPC1B | protein_coding | -0.62 | 1.87E-05 |
| AC138430.1 | ROR1 | protein_coding | -0.60 | 3.55E-05 |
| AC138430.1 | FKBP1A | protein_coding | -0.60 | 3.22E-05 |
| AC138430.1 | CNIH2 | protein_coding | -0.56 | 0.000164043 |
| AC138430.1 | PRADC1 | protein_coding | 0.67 | 1.95E-06 |
| AC138430.1 | PFKM | protein_coding | -0.52 | 0.000569099 |
| AC138430.1 | TBX15 | protein_coding | 0.77 | 3.46E-09 |
| AC138430.1 | ACSS3 | protein_coding | 0.66 | 3.37E-06 |
| AC138430.1 | MAP2K4 | protein_coding | 0.51 | 0.00073413 |
| AC138430.1 | DBI | protein_coding | 0.61 | 2.74E-05 |
| AC138430.1 | PYGB | protein_coding | -0.59 | 4.68E-05 |
| AC138430.1 | ABCD3 | protein_coding | 0.70 | 2.86E-07 |
| AC138430.1 | DMBX1 | protein_coding | -0.58 | 7.15E-05 |
| AC138430.1 | KIAA2013 | protein_coding | 0.65 | 4.70E-06 |
| AC138430.1 | H2AC7 | protein_coding | -0.54 | 0.000271327 |
| AC138430.1 | UQCRC2 | protein_coding | 0.55 | 0.000220844 |
| AC138430.1 | AOX1 | protein_coding | 0.65 | 4.75E-06 |
| AC138430.1 | CST6 | protein_coding | -0.53 | 0.000343616 |
| AC138430.1 | KIAA0930 | protein_coding | -0.53 | 0.000388057 |
| AC138430.1 | TP53I11 | protein_coding | -0.76 | 9.14E-09 |
| AC138430.1 | CRAT | protein_coding | 0.81 | 1.36E-10 |
| AC138430.1 | CYP2A7 | protein_coding | 0.73 | 8.14E-08 |
| AC138430.1 | ORAI3 | protein_coding | 0.52 | 0.000529771 |
| AC138430.1 | AHSG | protein_coding | 0.68 | 9.35E-07 |
| AC138430.1 | FUBP1 | protein_coding | -0.72 | 1.34E-07 |
| AC138430.1 | SSBP4 | protein_coding | -0.67 | 1.98E-06 |
| AC138430.1 | DALRD3 | protein_coding | -0.54 | 0.000278583 |
| AC138430.1 | RMND5A | protein_coding | 0.85 | 1.50E-12 |
| AC138430.1 | NDST3 | protein_coding | 0.66 | 2.29E-06 |
| AC138430.1 | LRIG2 | protein_coding | -0.54 | 0.00031774 |
| AC138430.1 | PIP4K2B | protein_coding | -0.66 | 3.24E-06 |
| AC138430.1 | AC068946.1 | protein_coding | -0.58 | 6.66E-05 |
| AC138430.1 | TMEM54 | protein_coding | -0.57 | 0.000109685 |
| AC138430.1 | INHBE | protein_coding | 0.59 | 5.49E-05 |
| AC138430.1 | EFHC1 | protein_coding | -0.62 | 1.78E-05 |
| AC138430.1 | SMARCA4 | protein_coding | -0.80 | 4.57E-10 |
| AC138430.1 | CYP27A1 | protein_coding | 0.74 | 3.20E-08 |
| AC138430.1 | COX16 | protein_coding | 0.58 | 7.95E-05 |
| AC138430.1 | ADGRG7 | protein_coding | 0.54 | 0.000246947 |
| AC138430.1 | ZFP1 | protein_coding | 0.75 | 1.37E-08 |
| AC138430.1 | CXorf66 | protein_coding | 0.74 | 3.19E-08 |
| AC138430.1 | SLC44A2 | protein_coding | -0.74 | 3.39E-08 |
| AC138430.1 | SPRYD7 | protein_coding | 0.73 | 7.52E-08 |
| AC138430.1 | SLC6A2 | protein_coding | 0.61 | 1.92E-05 |
| AC138430.1 | DLGAP4 | protein_coding | -0.69 | 7.30E-07 |
| AC138430.1 | P3H4 | protein_coding | -0.73 | 7.62E-08 |
| AC138430.1 | SLC2A9 | protein_coding | 0.72 | 1.05E-07 |
| AC138430.1 | FUT11 | protein_coding | -0.59 | 4.76E-05 |
| AC138430.1 | APH1B | protein_coding | -0.62 | 1.64E-05 |
| AC138430.1 | TSSK6 | protein_coding | -0.52 | 0.000467805 |
| AC138430.1 | SLC25A42 | protein_coding | 0.82 | 7.87E-11 |
| AC138430.1 | MPV17 | protein_coding | -0.68 | 9.83E-07 |
| AC138430.1 | HDAC6 | protein_coding | 0.84 | 7.09E-12 |
| AC138430.1 | MYH10 | protein_coding | 0.51 | 0.00072202 |
| AC138430.1 | NEDD4 | protein_coding | 0.60 | 3.03E-05 |
| AC138430.1 | MAIP1 | protein_coding | 0.73 | 5.48E-08 |
| AC138430.1 | NIFK | protein_coding | -0.61 | 1.99E-05 |
| AC138430.1 | SMLR1 | protein_coding | 0.62 | 1.67E-05 |
| AC138430.1 | PMPCB | protein_coding | 0.69 | 7.32E-07 |
| AC138430.1 | AARSD1 | protein_coding | -0.56 | 0.00013072 |
| AC138430.1 | NEDD1 | protein_coding | -0.53 | 0.00042915 |
| AC138430.1 | FCN3 | protein_coding | 0.66 | 3.42E-06 |
| AC138430.1 | REEP4 | protein_coding | -0.61 | 2.35E-05 |
| AC138430.1 | ACACB | protein_coding | 0.83 | 2.68E-11 |
| AC138430.1 | RIDA | protein_coding | 0.76 | 6.68E-09 |
| AC138430.1 | BHLHA9 | protein_coding | 0.58 | 6.06E-05 |
| AC138430.1 | RUNDC1 | protein_coding | -0.71 | 1.89E-07 |
| AC138430.1 | EFNA2 | protein_coding | 0.59 | 5.67E-05 |
| AC138430.1 | MNT | protein_coding | -0.57 | 9.57E-05 |
| AC138430.1 | ABCA10 | protein_coding | 0.72 | 9.61E-08 |
| AC138430.1 | YWHAZ | protein_coding | -0.73 | 5.81E-08 |
| AC138430.1 | RHCG | protein_coding | 0.52 | 0.000506694 |
| AC138430.1 | BEX4 | protein_coding | -0.52 | 0.000573709 |
| AC138430.1 | EGFL6 | protein_coding | -0.67 | 1.99E-06 |
| AC138430.1 | EPAS1 | protein_coding | 0.67 | 2.09E-06 |
| AC138430.1 | AP1G2 | protein_coding | -0.54 | 0.00025612 |
| AC138430.1 | STIL | protein_coding | -0.77 | 4.02E-09 |
| AC138430.1 | USP1 | protein_coding | -0.60 | 3.16E-05 |
| AC138430.1 | GPD2 | protein_coding | -0.61 | 1.92E-05 |
| AC138430.1 | PITPNM2 | protein_coding | 0.56 | 0.000148661 |
| AC138430.1 | PTH1R | protein_coding | 0.57 | 8.73E-05 |
| AC138430.1 | RMDN3 | protein_coding | 0.78 | 1.81E-09 |
| AC138430.1 | TMEM38A | protein_coding | 0.56 | 0.000151591 |
| AC138430.1 | CYP4V2 | protein_coding | 0.80 | 3.34E-10 |
| AC138430.1 | BDH2 | protein_coding | 0.76 | 6.54E-09 |
| AC138430.1 | LRRC36 | protein_coding | -0.53 | 0.000398686 |
| AC138430.1 | PSTK | protein_coding | 0.72 | 1.17E-07 |
| AC138430.1 | PUS10 | protein_coding | 0.71 | 1.73E-07 |
| AC138430.1 | XPO5 | protein_coding | -0.56 | 0.0001365 |
| AC138430.1 | YWHAQ | protein_coding | -0.59 | 5.68E-05 |
| AC138430.1 | HSP90AB1 | protein_coding | -0.61 | 2.01E-05 |
| AC138430.1 | FAM20A | protein_coding | 0.63 | 9.60E-06 |
| AC138430.1 | SLC23A2 | protein_coding | 0.77 | 4.46E-09 |
| AC138430.1 | NUCB1 | protein_coding | 0.60 | 3.45E-05 |
| AC138430.1 | EEF1AKNMT | protein_coding | -0.54 | 0.000293138 |
| AC138430.1 | DDX39A | protein_coding | -0.77 | 3.01E-09 |
| AC138430.1 | DHRS4 | protein_coding | 0.77 | 5.55E-09 |
| AC138430.1 | CEP89 | protein_coding | -0.75 | 1.34E-08 |
| AC138430.1 | IDH2 | protein_coding | 0.54 | 0.000260285 |
| AC138430.1 | F13B | protein_coding | 0.73 | 5.58E-08 |
| AC138430.1 | TMBIM6 | protein_coding | 0.79 | 1.15E-09 |
| AC138430.1 | CPB2 | protein_coding | 0.62 | 1.28E-05 |
| AC138430.1 | CETP | protein_coding | 0.52 | 0.000459235 |
| AC138430.1 | TFCP2 | protein_coding | -0.61 | 1.95E-05 |
| AC138430.1 | ASAP3 | protein_coding | 0.51 | 0.000664221 |
| AC138430.1 | NUDT18 | protein_coding | -0.58 | 7.89E-05 |
| AC138430.1 | ILF3 | protein_coding | -0.83 | 2.25E-11 |
| AC138430.1 | ASIC1 | protein_coding | -0.54 | 0.000258852 |
| AC138430.1 | STBD1 | protein_coding | 0.68 | 8.35E-07 |
| AC138430.1 | PRRX2 | protein_coding | -0.62 | 1.62E-05 |
| AC138430.1 | INTS6L | protein_coding | -0.56 | 0.000156476 |
| AC138430.1 | PGS1 | protein_coding | -0.61 | 2.06E-05 |
| AC138430.1 | HNRNPA1P48 | protein_coding | -0.61 | 2.50E-05 |
| AC138430.1 | NPIPA1 | protein_coding | -0.53 | 0.000398344 |
| AC138430.1 | SLC25A10 | protein_coding | 0.67 | 1.86E-06 |
| AC138430.1 | INSIG1 | protein_coding | 0.78 | 1.37E-09 |
| AC138430.1 | GNB1L | protein_coding | -0.67 | 1.62E-06 |
| AC138430.1 | GPT2 | protein_coding | 0.76 | 7.42E-09 |
| AC138430.1 | ZNF514 | protein_coding | -0.67 | 1.43E-06 |
| AC138430.1 | PITX1 | protein_coding | -0.69 | 6.17E-07 |
| AC138430.1 | EP400 | protein_coding | -0.60 | 3.49E-05 |
| AC138430.1 | TEDC2 | protein_coding | -0.66 | 3.15E-06 |
| AC138430.1 | SMAD3 | protein_coding | -0.61 | 2.49E-05 |
| AC138430.1 | ABCB7 | protein_coding | 0.55 | 0.000226912 |
| AC138430.1 | KIF18B | protein_coding | -0.70 | 3.49E-07 |
| AC138430.1 | C1orf35 | protein_coding | -0.56 | 0.000163629 |
| AC138430.1 | PRDX3 | protein_coding | 0.82 | 3.67E-11 |
| AC138430.1 | TRIM34 | protein_coding | -0.51 | 0.000714596 |
| AC138430.1 | ASGR1 | protein_coding | 0.71 | 1.74E-07 |
| AC138430.1 | SLC25A38 | protein_coding | 0.61 | 1.99E-05 |
| AC138430.1 | XDH | protein_coding | 0.68 | 9.28E-07 |
| AC138430.1 | DHCR7 | protein_coding | 0.78 | 1.77E-09 |
| AC138430.1 | DCLK1 | protein_coding | -0.56 | 0.000130322 |
| AC138430.1 | RAB31 | protein_coding | -0.57 | 9.96E-05 |
| AC138430.1 | MKRN1 | protein_coding | -0.52 | 0.000449048 |
| AC138430.1 | CCDC24 | protein_coding | -0.51 | 0.000747505 |
| AC138430.1 | CFAP157 | protein_coding | -0.63 | 1.18E-05 |
| AC138430.1 | AGXT | protein_coding | 0.64 | 6.44E-06 |
| AC138430.1 | SDHB | protein_coding | 0.81 | 1.84E-10 |
| AC138430.1 | RBBP9 | protein_coding | 0.61 | 2.76E-05 |
| AC138430.1 | SLC5A7 | protein_coding | 0.57 | 0.000100252 |
| AC138430.1 | RFXANK | protein_coding | -0.65 | 4.60E-06 |
| AC138430.1 | TDRD15 | protein_coding | 0.78 | 1.91E-09 |
| AC138430.1 | FMO3 | protein_coding | 0.70 | 4.28E-07 |
| AC138430.1 | EPHX1 | protein_coding | 0.84 | 4.13E-12 |
| AC138430.1 | RAC3 | protein_coding | 0.52 | 0.000461603 |
| AC138430.1 | ERP27 | protein_coding | -0.58 | 7.36E-05 |
| AC138430.1 | DNMT1 | protein_coding | -0.66 | 2.70E-06 |
| AC138430.1 | SLC22A10 | protein_coding | 0.73 | 4.60E-08 |
| AC138430.1 | MORC1 | protein_coding | 0.53 | 0.000349284 |
| AC138430.1 | ITLN2 | protein_coding | 0.56 | 0.000120967 |
| AC138430.1 | LIPC | protein_coding | 0.56 | 0.000166929 |
| AC138430.1 | RUFY1 | protein_coding | -0.72 | 1.54E-07 |
| AC138430.1 | ZNF280B | protein_coding | -0.56 | 0.000129782 |
| AC138430.1 | CNTLN | protein_coding | 0.69 | 5.78E-07 |
| AC138430.1 | CHDH | protein_coding | 0.56 | 0.000123336 |
| AC138430.1 | TAF1D | protein_coding | -0.72 | 9.58E-08 |
| AC138430.1 | ADRA1A | protein_coding | 0.71 | 2.72E-07 |
| AC138430.1 | FANCG | protein_coding | -0.66 | 3.28E-06 |
| AC138430.1 | TMEM44 | protein_coding | -0.67 | 1.33E-06 |
| AC138430.1 | ABHD14A-ACY1 | protein_coding | 0.64 | 6.34E-06 |
| AC138430.1 | NCOA5 | protein_coding | -0.58 | 8.20E-05 |
| AC138430.1 | TMEM41B | protein_coding | 0.66 | 2.54E-06 |
| AC138430.1 | MT-CO3 | protein_coding | 0.66 | 3.20E-06 |
| AC138430.1 | ARSF | protein_coding | 0.69 | 7.38E-07 |
| AC138430.1 | GLOD5 | protein_coding | 0.57 | 0.000109771 |
| AC138430.1 | GPSM1 | protein_coding | -0.67 | 1.57E-06 |
| AC138430.1 | SRCAP | protein_coding | -0.78 | 1.76E-09 |
| AC138430.1 | RNF34 | protein_coding | -0.60 | 3.27E-05 |
| AC138430.1 | MRPL17 | protein_coding | -0.55 | 0.000208457 |
| AC138430.1 | ZCCHC2 | protein_coding | 0.74 | 3.11E-08 |
| AC138430.1 | DMD | protein_coding | 0.58 | 8.52E-05 |
| AC138430.1 | NR1H3 | protein_coding | 0.61 | 2.08E-05 |
| AC138430.1 | DENND3 | protein_coding | -0.71 | 2.24E-07 |
| AC138430.1 | ELOVL1 | protein_coding | -0.59 | 4.34E-05 |
| AC138430.1 | GPN3 | protein_coding | 0.54 | 0.00029397 |
| AC138430.1 | NCK2 | protein_coding | -0.74 | 2.95E-08 |
| AC138430.1 | SLC35D1 | protein_coding | 0.74 | 2.48E-08 |
| AC138430.1 | ABCC4 | protein_coding | -0.67 | 1.56E-06 |
| AC138430.1 | SLC43A1 | protein_coding | 0.60 | 3.83E-05 |
| AC138430.1 | C5orf30 | protein_coding | -0.67 | 1.63E-06 |
| AC138430.1 | GATB | protein_coding | 0.63 | 8.86E-06 |
| AC138430.1 | PRR18 | protein_coding | 0.64 | 8.22E-06 |
| AC138430.1 | AP2B1 | protein_coding | -0.73 | 7.49E-08 |
| AC138430.1 | SMIM5 | protein_coding | -0.59 | 4.40E-05 |
| AC138430.1 | ABCA9 | protein_coding | 0.66 | 3.36E-06 |
| AC138430.1 | NCOR2 | protein_coding | -0.76 | 6.83E-09 |
| AC138430.1 | ZNHIT6 | protein_coding | -0.63 | 1.17E-05 |
| AC138430.1 | SPATS2 | protein_coding | -0.79 | 5.92E-10 |
| AC138430.1 | PINK1 | protein_coding | 0.78 | 2.80E-09 |
| AC138430.1 | RMDN2 | protein_coding | 0.82 | 5.42E-11 |
| AC138430.1 | HIGD1C | protein_coding | 0.51 | 0.000667508 |
| AC138430.1 | SLC28A2 | protein_coding | 0.66 | 2.53E-06 |
| AC138430.1 | RPUSD1 | protein_coding | -0.61 | 2.28E-05 |
| AC138430.1 | DPH3 | protein_coding | 0.50 | 0.000841417 |
| AC138430.1 | SMCO4 | protein_coding | 0.73 | 8.38E-08 |
| AC138430.1 | CCNG2 | protein_coding | -0.54 | 0.000297441 |
| AC138430.1 | SCYL1 | protein_coding | 0.55 | 0.00018319 |
| AC138430.1 | CMC1 | protein_coding | 0.51 | 0.000749374 |
| AC138430.1 | LLGL1 | protein_coding | -0.67 | 1.46E-06 |
| AC138430.1 | SMG9 | protein_coding | -0.69 | 5.03E-07 |
| AC138430.1 | DNASE1 | protein_coding | -0.57 | 0.000104809 |
| AC138430.1 | GRHPR | protein_coding | 0.79 | 6.47E-10 |
| AC138430.1 | SAP30BP | protein_coding | -0.57 | 0.000114227 |
| AC138430.1 | GATD3B | protein_coding | 0.56 | 0.000149262 |
| AC138430.1 | TPM3 | protein_coding | -0.70 | 4.65E-07 |
| AC138430.1 | LRP8 | protein_coding | -0.66 | 2.79E-06 |
| AC138430.1 | SORBS3 | protein_coding | 0.51 | 0.0007374 |
| AC138430.1 | AHR | protein_coding | 0.56 | 0.000141074 |
| AC138430.1 | SLC39A8 | protein_coding | 0.72 | 1.10E-07 |
| AC138430.1 | DNAJC15 | protein_coding | 0.51 | 0.000580755 |
| AC138430.1 | UBE2D4 | protein_coding | 0.68 | 1.27E-06 |
| AC138430.1 | A2M | protein_coding | 0.77 | 2.99E-09 |
| AC138430.1 | TSPAN14 | protein_coding | -0.62 | 1.29E-05 |
| AC138430.1 | RXRA | protein_coding | 0.85 | 1.45E-12 |
| AC138430.1 | COX6C | protein_coding | 0.53 | 0.000387876 |
| AC138430.1 | MRPL57 | protein_coding | 0.68 | 1.23E-06 |
| AC138430.1 | UBAP2L | protein_coding | -0.64 | 8.12E-06 |
| AC138430.1 | ANKRD27 | protein_coding | -0.61 | 2.21E-05 |
| AC138430.1 | SERINC1 | protein_coding | 0.67 | 1.62E-06 |
| AC138430.1 | ADH6 | protein_coding | 0.67 | 2.16E-06 |
| AC138430.1 | MITD1 | protein_coding | -0.68 | 1.03E-06 |
| AC138430.1 | DNAJB6 | protein_coding | -0.59 | 4.35E-05 |
| AC138430.1 | FMR1 | protein_coding | 0.57 | 0.000120629 |
| AC138430.1 | APOH | protein_coding | 0.71 | 2.34E-07 |
| AC138430.1 | RGN | protein_coding | 0.77 | 5.70E-09 |
| AC138430.1 | KLHL2 | protein_coding | 0.67 | 1.82E-06 |
| AC138430.1 | TXNDC9 | protein_coding | -0.53 | 0.000387409 |
| AC138430.1 | CSRNP1 | protein_coding | 0.54 | 0.000254552 |
| AC138430.1 | NRBF2 | protein_coding | 0.66 | 2.51E-06 |
| AC138430.1 | SIRT2 | protein_coding | 0.51 | 0.00075641 |
| AC138430.1 | GUSB | protein_coding | 0.75 | 1.51E-08 |
| AC138430.1 | CASTOR3 | protein_coding | -0.64 | 7.24E-06 |
| AC138430.1 | ASPHD1 | protein_coding | -0.63 | 8.72E-06 |
| AC138430.1 | GOLGA6D | protein_coding | 0.53 | 0.000337469 |
| AC138430.1 | PLG | protein_coding | 0.62 | 1.68E-05 |
| AC138430.1 | QRFP | protein_coding | -0.67 | 1.57E-06 |
| AC138430.1 | ZNF469 | protein_coding | -0.62 | 1.74E-05 |
| AC138430.1 | PC | protein_coding | 0.73 | 5.35E-08 |
| AC138430.1 | C1RL | protein_coding | 0.62 | 1.29E-05 |
| AC138430.1 | OLFML2B | protein_coding | -0.69 | 7.29E-07 |
| AC138430.1 | GGACT | protein_coding | 0.69 | 7.23E-07 |
| AC138430.1 | PTGFRN | protein_coding | -0.51 | 0.000719491 |
| AC138430.1 | PUM3 | protein_coding | -0.51 | 0.000661207 |
| AC138430.1 | TDRD6 | protein_coding | 0.51 | 0.000655629 |
| AC138430.1 | HAL | protein_coding | 0.74 | 3.39E-08 |
| AC138430.1 | CCL20 | protein_coding | -0.54 | 0.000234847 |
| AC138430.1 | MIS18BP1 | protein_coding | -0.53 | 0.000381447 |
| AC138430.1 | SPAG17 | protein_coding | -0.56 | 0.000139637 |
| AC138430.1 | IYD | protein_coding | 0.60 | 3.81E-05 |
| AC138430.1 | CYP3A43 | protein_coding | 0.72 | 9.91E-08 |
| AC138430.1 | UBXN11 | protein_coding | -0.55 | 0.000206584 |
| AC138430.1 | SPSB3 | protein_coding | 0.56 | 0.00014683 |
| AC138430.1 | SMPDL3A | protein_coding | 0.74 | 3.10E-08 |
| AC138430.1 | PGRMC1 | protein_coding | 0.83 | 2.03E-11 |
| AC138430.1 | CD36 | protein_coding | 0.54 | 0.000314309 |
| AC138430.1 | XRCC1 | protein_coding | -0.70 | 3.73E-07 |
| AC138430.1 | NUDCD2 | protein_coding | 0.58 | 6.87E-05 |
| AC138430.1 | HS3ST3B1 | protein_coding | 0.55 | 0.000186979 |
| AC138430.1 | NR2C2AP | protein_coding | -0.72 | 1.32E-07 |
| AC138430.1 | ZNF213 | protein_coding | -0.79 | 8.87E-10 |
| AC138430.1 | MARCKS | protein_coding | -0.59 | 4.27E-05 |
| AC138430.1 | AMDHD1 | protein_coding | 0.70 | 4.28E-07 |
| AC138430.1 | GIT1 | protein_coding | -0.74 | 4.11E-08 |
| AC138430.1 | EPHA10 | protein_coding | -0.69 | 6.93E-07 |
| AC138430.1 | GSK3A | protein_coding | -0.54 | 0.000305038 |
| AC138430.1 | MYBL2 | protein_coding | -0.69 | 5.67E-07 |
| AC138430.1 | CHML | protein_coding | -0.60 | 3.83E-05 |
| AC138430.1 | C9orf116 | protein_coding | -0.57 | 0.000109392 |
| AC138430.1 | STRN4 | protein_coding | -0.57 | 8.81E-05 |
| AC138430.1 | TRMT11 | protein_coding | 0.55 | 0.000228728 |
| AC138430.1 | FBXW9 | protein_coding | -0.55 | 0.000201915 |
| AC138430.1 | DEPDC1 | protein_coding | -0.68 | 8.78E-07 |
| AC138430.1 | PDSS2 | protein_coding | 0.74 | 2.73E-08 |
| AC138430.1 | CDNF | protein_coding | 0.65 | 3.74E-06 |
| AC138430.1 | FOXRED1 | protein_coding | 0.65 | 3.81E-06 |
| AC138430.1 | ELK3 | protein_coding | -0.54 | 0.000309209 |
| AC138430.1 | LNX2 | protein_coding | 0.66 | 2.65E-06 |
| AC138430.1 | ZFP64 | protein_coding | -0.55 | 0.000232343 |
| AC138430.1 | ATRNL1 | protein_coding | 0.50 | 0.000819317 |
| AC138430.1 | ETFA | protein_coding | 0.75 | 1.50E-08 |
| AC138430.1 | SURF1 | protein_coding | 0.56 | 0.000157988 |
| AC138430.1 | PAX8 | protein_coding | -0.51 | 0.000715929 |
| AC138430.1 | GLUD1 | protein_coding | 0.83 | 1.36E-11 |
| AC138430.1 | LIFR | protein_coding | 0.58 | 6.78E-05 |
| AC138430.1 | CDCA2 | protein_coding | -0.70 | 3.45E-07 |
| AC138430.1 | SEC62 | protein_coding | 0.68 | 1.14E-06 |
| AC138430.1 | ATP11C | protein_coding | 0.66 | 2.19E-06 |
| AC138430.1 | GRAMD1B | protein_coding | -0.58 | 8.12E-05 |
| AC138430.1 | OSBPL5 | protein_coding | -0.69 | 7.79E-07 |
| AC138430.1 | IQCD | protein_coding | -0.72 | 9.58E-08 |
| AC138430.1 | MBNL3 | protein_coding | 0.53 | 0.000355921 |
| AC138430.1 | PCCA | protein_coding | 0.68 | 1.22E-06 |
| AC138430.1 | PPP2R1A | protein_coding | -0.54 | 0.000294538 |
| AC138430.1 | TEX9 | protein_coding | -0.60 | 3.29E-05 |
| AC138430.1 | ZG16B | protein_coding | -0.58 | 7.46E-05 |
| AC138430.1 | ZBTB26 | protein_coding | -0.52 | 0.000551348 |
| AC138430.1 | BLM | protein_coding | -0.66 | 2.45E-06 |
| AC138430.1 | GPHN | protein_coding | 0.80 | 2.52E-10 |
| AC138430.1 | GSTA4 | protein_coding | 0.52 | 0.000540105 |
| AC138430.1 | ITIH1 | protein_coding | 0.62 | 1.85E-05 |
| AC138430.1 | XPR1 | protein_coding | -0.54 | 0.000247898 |
| AC138430.1 | RTKN2 | protein_coding | -0.65 | 5.03E-06 |
| AC138430.1 | ZNF605 | protein_coding | -0.51 | 0.000631695 |
| AC138430.1 | TNFSF15 | protein_coding | -0.68 | 8.15E-07 |
| AC138430.1 | WNK3 | protein_coding | 0.77 | 5.87E-09 |
| AC138430.1 | TMEM51 | protein_coding | -0.71 | 1.76E-07 |
| AC138430.1 | BCL9 | protein_coding | -0.55 | 0.000179893 |
| AC138430.1 | NSD2 | protein_coding | -0.58 | 6.39E-05 |
| AC138430.1 | SMOX | protein_coding | -0.61 | 2.10E-05 |
| AC138430.1 | EZH1 | protein_coding | -0.60 | 3.99E-05 |
| AC138430.1 | EIF3D | protein_coding | -0.79 | 6.29E-10 |
| AC138430.1 | IL27RA | protein_coding | -0.54 | 0.000278638 |
| AC138430.1 | ANXA10 | protein_coding | 0.66 | 2.50E-06 |
| AC138430.1 | XPO6 | protein_coding | -0.60 | 2.96E-05 |
| AC138430.1 | RUNX1 | protein_coding | -0.50 | 0.000853782 |
| AC138430.1 | CDIP1 | protein_coding | 0.65 | 5.01E-06 |
| AC138430.1 | TMLHE | protein_coding | 0.70 | 4.17E-07 |
| AC138430.1 | C20orf144 | protein_coding | -0.54 | 0.000290504 |
| AC138430.1 | SHFL | protein_coding | 0.61 | 2.15E-05 |
| AC138430.1 | DHCR24 | protein_coding | 0.50 | 0.000809858 |
| AC138430.1 | BAX | protein_coding | -0.61 | 2.79E-05 |
| AC138430.1 | CLIP2 | protein_coding | -0.79 | 7.24E-10 |
| AC138430.1 | DECR1 | protein_coding | 0.76 | 6.75E-09 |
| AC138430.1 | DPF2 | protein_coding | -0.69 | 6.57E-07 |
| AC138430.1 | CBX1 | protein_coding | -0.67 | 1.75E-06 |
| AC138430.1 | PAEP | protein_coding | -0.56 | 0.000138386 |
| AC138430.1 | PHLPP1 | protein_coding | 0.60 | 3.57E-05 |
| AC138430.1 | TRIM11 | protein_coding | -0.70 | 3.67E-07 |
| AC138430.1 | RAPGEF4 | protein_coding | 0.78 | 1.43E-09 |
| AC138430.1 | MYL6B | protein_coding | -0.56 | 0.00015591 |
| AC138430.1 | LMBRD1 | protein_coding | 0.54 | 0.000247498 |
| AC138430.1 | AGER | protein_coding | -0.52 | 0.000472608 |
| AC138430.1 | VASP | protein_coding | -0.66 | 2.51E-06 |
| AC138430.1 | TMTC2 | protein_coding | -0.66 | 2.96E-06 |
| AC138430.1 | MSRB1 | protein_coding | 0.74 | 4.15E-08 |
| AC138430.1 | ABCA3 | protein_coding | -0.81 | 1.78E-10 |
| AC138430.1 | KIAA1841 | protein_coding | -0.74 | 3.74E-08 |
| AC138430.1 | MRE11 | protein_coding | -0.65 | 4.37E-06 |
| AC138430.1 | PRSS22 | protein_coding | -0.66 | 3.11E-06 |
| AC138430.1 | NCF2 | protein_coding | -0.58 | 7.07E-05 |
| AC138430.1 | SLC20A2 | protein_coding | 0.68 | 1.28E-06 |
| AC138430.1 | AL845331.2 | protein_coding | 0.65 | 4.45E-06 |
| AC138430.1 | CLHC1 | protein_coding | -0.60 | 3.95E-05 |
| AC138430.1 | ZNF496 | protein_coding | -0.64 | 7.11E-06 |
| AC138430.1 | ZCCHC14 | protein_coding | 0.60 | 3.55E-05 |
| AC138430.1 | ENHO | protein_coding | 0.70 | 4.09E-07 |
| AC138430.1 | RBP5 | protein_coding | 0.65 | 3.68E-06 |
| AC138430.1 | MATN3 | protein_coding | -0.55 | 0.000211873 |
| AC138430.1 | BAIAP2 | protein_coding | 0.52 | 0.000461224 |
| AC138430.1 | PRKD2 | protein_coding | -0.71 | 2.14E-07 |
| AC138430.1 | MCCC2 | protein_coding | 0.55 | 0.000183471 |
| AC138430.1 | PDZD7 | protein_coding | -0.65 | 3.62E-06 |
| AC138430.1 | EVA1A | protein_coding | 0.67 | 1.72E-06 |
| AC138430.1 | AMBP | protein_coding | 0.66 | 3.40E-06 |
| AC138430.1 | SACS | protein_coding | -0.53 | 0.000426785 |
| AC138430.1 | SCML1 | protein_coding | 0.61 | 2.13E-05 |
| AC138430.1 | CTXND1 | protein_coding | 0.66 | 2.49E-06 |
| AC138430.1 | PPP4R4 | protein_coding | 0.70 | 4.53E-07 |
| AC138430.1 | TRAF1 | protein_coding | -0.53 | 0.000374625 |
| AC138430.1 | CHST3 | protein_coding | -0.56 | 0.000142129 |
| AC138430.1 | CYP2W1 | protein_coding | -0.52 | 0.000539348 |
| AC138430.1 | B3GALT5 | protein_coding | -0.55 | 0.000202639 |
| AC138430.1 | F9 | protein_coding | 0.63 | 9.61E-06 |
| AC138430.1 | AP003419.1 | protein_coding | -0.52 | 0.000435958 |
| AC138430.1 | SARDH | protein_coding | 0.74 | 4.46E-08 |
| AC138430.1 | CDCA8 | protein_coding | -0.63 | 1.07E-05 |
| AC138430.1 | GJC1 | protein_coding | -0.68 | 1.20E-06 |
| AC138430.1 | LRFN1 | protein_coding | -0.59 | 5.38E-05 |
| AC138430.1 | DYRK1B | protein_coding | -0.66 | 2.58E-06 |
| AC138430.1 | MFAP3L | protein_coding | 0.68 | 1.24E-06 |
| AC138430.1 | MRPL41 | protein_coding | 0.54 | 0.000252816 |
| AC138430.1 | GLMN | protein_coding | -0.55 | 0.000205514 |
| AC138430.1 | CCDC25 | protein_coding | 0.69 | 4.93E-07 |
| AC138430.1 | HIPK2 | protein_coding | 0.52 | 0.000476898 |
| AC138430.1 | ACY3 | protein_coding | 0.69 | 5.70E-07 |
| AC138430.1 | SIRT7 | protein_coding | -0.57 | 0.00011669 |
| AC138430.1 | GIPC2 | protein_coding | 0.51 | 0.000714911 |
| AC138430.1 | GGCX | protein_coding | 0.73 | 8.42E-08 |
| AC138430.1 | RORC | protein_coding | 0.69 | 5.46E-07 |
| AC138430.1 | ABHD1 | protein_coding | 0.69 | 5.68E-07 |
| AC138430.1 | ELL2 | protein_coding | 0.72 | 9.24E-08 |
| AC138430.1 | SDF4 | protein_coding | 0.63 | 1.01E-05 |
| AC138430.1 | MKI67 | protein_coding | -0.71 | 2.34E-07 |
| AC138430.1 | ENTPD1 | protein_coding | -0.61 | 2.27E-05 |
| AC138430.1 | TRPC1 | protein_coding | -0.58 | 7.44E-05 |
| AC138430.1 | BPHL | protein_coding | 0.69 | 5.83E-07 |
| AC138430.1 | IMMP2L | protein_coding | 0.51 | 0.000610681 |
| AC138430.1 | TEAD2 | protein_coding | -0.71 | 2.31E-07 |
| AC138430.1 | ARHGEF10L | protein_coding | 0.70 | 3.11E-07 |
| AC138430.1 | TMEM132A | protein_coding | -0.74 | 2.47E-08 |
| AC138430.1 | MED11 | protein_coding | 0.58 | 7.25E-05 |
| AC138430.1 | CPN2 | protein_coding | 0.64 | 6.06E-06 |
| AC138430.1 | TNFAIP8L1 | protein_coding | 0.58 | 6.52E-05 |
| AC138430.1 | BBS1 | protein_coding | -0.62 | 1.60E-05 |
| AC138430.1 | CHD4 | protein_coding | -0.58 | 6.02E-05 |
| AC138430.1 | FHOD3 | protein_coding | -0.51 | 0.000611286 |
| AC138430.1 | IGFBP4 | protein_coding | 0.64 | 6.54E-06 |
| AC138430.1 | WDR81 | protein_coding | 0.57 | 9.79E-05 |
| AC138430.1 | WDR63 | protein_coding | -0.54 | 0.000286121 |
| AC138430.1 | SLC4A5 | protein_coding | -0.62 | 1.79E-05 |
| AC138430.1 | PTBP3 | protein_coding | -0.61 | 2.27E-05 |
| AC138430.1 | REEP6 | protein_coding | 0.63 | 9.01E-06 |
| AC138430.1 | POLG2 | protein_coding | -0.51 | 0.000644863 |
| AC138430.1 | HIF1AN | protein_coding | -0.59 | 5.17E-05 |
| AC138430.1 | MYD88 | protein_coding | 0.54 | 0.000295756 |
| AC138430.1 | MVP | protein_coding | -0.75 | 2.35E-08 |
| AC138430.1 | ARNT2 | protein_coding | -0.73 | 5.37E-08 |
| AC138430.1 | P2RX3 | protein_coding | 0.75 | 1.79E-08 |
| AC138430.1 | SMIM4 | protein_coding | 0.55 | 0.00019573 |
| AC138430.1 | FBLIM1 | protein_coding | -0.74 | 2.43E-08 |
| AC138430.1 | C10orf143 | protein_coding | 0.53 | 0.00034673 |
| AC138430.1 | HACD3 | protein_coding | 0.57 | 9.03E-05 |
| AC138430.1 | PITHD1 | protein_coding | 0.63 | 1.20E-05 |
| AC138430.1 | SGK1 | protein_coding | 0.50 | 0.000833509 |
| AC138430.1 | SH2B2 | protein_coding | -0.53 | 0.000346444 |
| AC138430.1 | OMP | protein_coding | 0.51 | 0.000580552 |
| AC138430.1 | CFH | protein_coding | 0.72 | 1.45E-07 |
| AC138430.1 | HID1 | protein_coding | -0.66 | 2.72E-06 |
| AC138430.1 | VNN1 | protein_coding | 0.55 | 0.000222717 |
| AC138430.1 | PTGR1 | protein_coding | 0.76 | 7.22E-09 |
| AC138430.1 | RAF1 | protein_coding | 0.69 | 4.79E-07 |
| AC138430.1 | SBNO2 | protein_coding | -0.66 | 2.96E-06 |
| AC138430.1 | TAX1BP3 | protein_coding | -0.70 | 4.17E-07 |
| AC138430.1 | ARL4D | protein_coding | 0.67 | 1.57E-06 |
| AC138430.1 | SAMD4B | protein_coding | -0.56 | 0.000134051 |
| AC138430.1 | SLC43A2 | protein_coding | -0.52 | 0.000499695 |
| AC138430.1 | DGAT2 | protein_coding | 0.70 | 3.49E-07 |
| AC138430.1 | IGFALS | protein_coding | 0.61 | 1.93E-05 |
| AC138430.1 | DDA1 | protein_coding | -0.66 | 2.97E-06 |
| AC138430.1 | FAR1 | protein_coding | -0.66 | 2.69E-06 |
| AC138430.1 | OTC | protein_coding | 0.65 | 3.61E-06 |
| AC138430.1 | XYLT2 | protein_coding | -0.62 | 1.61E-05 |
| AC138430.1 | TRAM1L1 | protein_coding | -0.66 | 2.73E-06 |
| AC138430.1 | CPN1 | protein_coding | 0.59 | 4.75E-05 |
| AC138430.1 | HROB | protein_coding | -0.69 | 7.26E-07 |
| AC138430.1 | RUVBL1 | protein_coding | -0.67 | 1.75E-06 |
| AC138430.1 | GPR27 | protein_coding | -0.59 | 5.66E-05 |
| AC138430.1 | SLITRK6 | protein_coding | 0.55 | 0.000194899 |
| AC138430.1 | TOR3A | protein_coding | -0.53 | 0.000393481 |
| AC138430.1 | RAB3IP | protein_coding | -0.53 | 0.000410947 |
| AC138430.1 | SORD | protein_coding | 0.81 | 1.81E-10 |
| AC138430.1 | PDCD11 | protein_coding | -0.55 | 0.000178969 |
| AC138430.1 | GMNN | protein_coding | -0.58 | 8.29E-05 |
| AC138430.1 | GCK | protein_coding | 0.78 | 2.54E-09 |
| AC138430.1 | ZNF48 | protein_coding | -0.55 | 0.000220175 |
| AC138430.1 | INTS6 | protein_coding | 0.54 | 0.000242245 |
| AC138430.1 | ADH5 | protein_coding | 0.72 | 1.05E-07 |
| AC138430.1 | CSNK1G1 | protein_coding | -0.72 | 8.96E-08 |
| AC138430.1 | GPR88 | protein_coding | 0.71 | 2.06E-07 |
| AC138430.1 | CACNB2 | protein_coding | 0.72 | 8.91E-08 |
| AC138430.1 | TMEM184B | protein_coding | -0.66 | 3.08E-06 |
| AC138430.1 | PSAT1 | protein_coding | 0.54 | 0.000242272 |
| AC138430.1 | SLC25A14 | protein_coding | -0.53 | 0.000419138 |
| AC138430.1 | MEX3C | protein_coding | -0.52 | 0.000529969 |
| AC138430.1 | FUZ | protein_coding | -0.54 | 0.000236523 |
| AC138430.1 | BCL7A | protein_coding | -0.52 | 0.000503952 |
| AC138430.1 | CYP4F2 | protein_coding | 0.70 | 3.11E-07 |
| AC138430.1 | SOCS6 | protein_coding | 0.52 | 0.000497849 |
| AC138430.1 | RAB40A | protein_coding | -0.75 | 1.47E-08 |
| AC138430.1 | ACY1 | protein_coding | 0.78 | 2.32E-09 |
| AC138430.1 | HNRNPA1 | protein_coding | -0.73 | 5.00E-08 |
| AC138430.1 | AMPD3 | protein_coding | -0.68 | 1.25E-06 |
| AC138430.1 | TP53BP1 | protein_coding | -0.62 | 1.82E-05 |
| AC138430.1 | FOXJ1 | protein_coding | -0.63 | 1.18E-05 |
| AC138430.1 | MFAP2 | protein_coding | -0.60 | 3.35E-05 |
| AC138430.1 | NOD1 | protein_coding | -0.65 | 3.91E-06 |
| AC138430.1 | RRBP1 | protein_coding | 0.59 | 4.99E-05 |
| AC138430.1 | SLC4A9 | protein_coding | -0.55 | 0.000190108 |
| AC138430.1 | PRXL2A | protein_coding | 0.69 | 6.31E-07 |
| AC138430.1 | PACRG | protein_coding | 0.58 | 6.26E-05 |
| AC138430.1 | HLF | protein_coding | 0.65 | 3.87E-06 |
| AC138430.1 | TOR4A | protein_coding | -0.65 | 3.60E-06 |
| AC138430.1 | AURKB | protein_coding | -0.70 | 4.58E-07 |
| AC138430.1 | ZNF384 | protein_coding | -0.54 | 0.000281005 |
| AC138430.1 | ZNF92 | protein_coding | -0.54 | 0.000241819 |
| AC138430.1 | TMEM156 | protein_coding | -0.52 | 0.000501585 |
| AC138430.1 | POC5 | protein_coding | -0.65 | 3.67E-06 |
| AC138430.1 | PWP1 | protein_coding | -0.50 | 0.000825872 |
| AC138430.1 | ZNF341 | protein_coding | -0.58 | 8.56E-05 |
| AC138430.1 | CCDC102B | protein_coding | -0.61 | 2.64E-05 |
| AC138430.1 | PIK3R1 | protein_coding | 0.71 | 2.51E-07 |
| AC138430.1 | TUT7 | protein_coding | 0.56 | 0.000131756 |
| AC138430.1 | NEK6 | protein_coding | 0.58 | 7.78E-05 |
| AC138430.1 | AIFM1 | protein_coding | 0.74 | 3.54E-08 |
| AC138430.1 | ADAM9 | protein_coding | -0.58 | 6.49E-05 |
| AC138430.1 | PACS2 | protein_coding | -0.71 | 1.83E-07 |
| AC138430.1 | TRIM16 | protein_coding | -0.64 | 8.30E-06 |
| AC138430.1 | GAMT | protein_coding | 0.77 | 3.31E-09 |
| AC138430.1 | MRAP | protein_coding | 0.53 | 0.000394427 |
| AC138430.1 | RPGRIP1L | protein_coding | -0.73 | 4.85E-08 |
| AC138430.1 | C1orf226 | protein_coding | 0.55 | 0.000211144 |
| AC138430.1 | SLC31A2 | protein_coding | 0.53 | 0.000404501 |
| AC138430.1 | NAT14 | protein_coding | -0.59 | 4.64E-05 |
| AC138430.1 | CEP131 | protein_coding | -0.73 | 7.42E-08 |
| AC138430.1 | CHMP3 | protein_coding | -0.69 | 7.60E-07 |
| AC138430.1 | PEX14 | protein_coding | 0.71 | 2.00E-07 |
| AC138430.1 | PLIN3 | protein_coding | -0.56 | 0.000154906 |
| AC138430.1 | LCE2D | protein_coding | 0.67 | 1.62E-06 |
| AC138430.1 | IGFBP2 | protein_coding | 0.63 | 1.14E-05 |
| AC138430.1 | HPR | protein_coding | 0.63 | 8.78E-06 |
| AC138430.1 | NPHP4 | protein_coding | -0.58 | 6.03E-05 |
| AC138430.1 | CISD3 | protein_coding | 0.62 | 1.34E-05 |
| AC138430.1 | ASS1 | protein_coding | 0.67 | 1.62E-06 |
| AC138430.1 | RHBDL2 | protein_coding | -0.55 | 0.000232411 |
| AC138430.1 | TMEM67 | protein_coding | -0.59 | 5.41E-05 |
| AC138430.1 | MED15 | protein_coding | -0.80 | 3.90E-10 |
| AC138430.1 | DRAM1 | protein_coding | -0.73 | 8.40E-08 |
| AC138430.1 | CDT1 | protein_coding | -0.72 | 1.39E-07 |
| AC138430.1 | DISP3 | protein_coding | -0.52 | 0.000520086 |
| AC138430.1 | PPP1R9A | protein_coding | -0.65 | 5.16E-06 |
| AC138430.1 | PNMA1 | protein_coding | -0.70 | 3.12E-07 |
| AC138430.1 | NFATC1 | protein_coding | -0.52 | 0.000506819 |
| AC138430.1 | RIMKLA | protein_coding | -0.64 | 5.83E-06 |
| AC138430.1 | RAI1 | protein_coding | -0.65 | 5.07E-06 |
| AC138430.1 | S100A2 | protein_coding | -0.59 | 4.50E-05 |
| AC138430.1 | KLF3 | protein_coding | 0.56 | 0.000153162 |
| AC138430.1 | EEF1D | protein_coding | -0.56 | 0.000153776 |
| AC138430.1 | TMED5 | protein_coding | 0.60 | 3.04E-05 |
| AC138430.1 | CNTD2 | protein_coding | 0.52 | 0.000505854 |
| AC138430.1 | NEIL3 | protein_coding | -0.58 | 6.79E-05 |
| AC138430.1 | CACNA1F | protein_coding | -0.62 | 1.48E-05 |
| AC138430.1 | MMS22L | protein_coding | -0.53 | 0.000342846 |
| AC138430.1 | ZBTB45 | protein_coding | -0.72 | 1.53E-07 |
| AC138430.1 | NAT9 | protein_coding | -0.63 | 1.25E-05 |
| AC138430.1 | CRK | protein_coding | 0.59 | 4.48E-05 |
| AC138430.1 | PIPOX | protein_coding | 0.76 | 1.09E-08 |
| AC138430.1 | FAM110A | protein_coding | -0.60 | 3.64E-05 |
| AC138430.1 | GC | protein_coding | 0.59 | 5.58E-05 |
| AC138430.1 | BRD3OS | protein_coding | -0.56 | 0.000123454 |
| AC138430.1 | CYP4F12 | protein_coding | 0.70 | 4.42E-07 |
| AC138430.1 | ADCY7 | protein_coding | -0.53 | 0.000372791 |
| AC138430.1 | REPS1 | protein_coding | 0.58 | 6.07E-05 |
| AC138430.1 | PHF19 | protein_coding | -0.57 | 9.16E-05 |
| AC138430.1 | FAM171A2 | protein_coding | -0.72 | 1.49E-07 |
| AC138430.1 | IL13RA1 | protein_coding | 0.66 | 2.65E-06 |
| AC138430.1 | BMP10 | protein_coding | 0.73 | 7.95E-08 |
| AC138430.1 | COMP | protein_coding | -0.54 | 0.000313007 |
| AC138430.1 | RPRD1B | protein_coding | 0.57 | 9.59E-05 |
| AC138430.1 | ADAMTSL2 | protein_coding | 0.59 | 5.95E-05 |
| AC138430.1 | A4GALT | protein_coding | -0.54 | 0.000306005 |
| AC138430.1 | ERICH3 | protein_coding | 0.56 | 0.000142701 |
| AC138430.1 | SLFN12 | protein_coding | -0.61 | 1.96E-05 |
| AC138430.1 | CELSR1 | protein_coding | -0.58 | 7.75E-05 |
| AC138430.1 | CYP4F11 | protein_coding | 0.71 | 1.76E-07 |
| AC138430.1 | FRY | protein_coding | 0.54 | 0.000280148 |
| AC138430.1 | SRSF3 | protein_coding | -0.52 | 0.00043375 |
| AC138430.1 | GLRX | protein_coding | 0.65 | 3.90E-06 |
| AC138430.1 | BTBD10 | protein_coding | -0.51 | 0.000705998 |
| AC138430.1 | COL8A1 | protein_coding | -0.51 | 0.00069634 |
| AC138430.1 | AC005041.1 | protein_coding | -0.67 | 2.16E-06 |
| AC138430.1 | UGT8 | protein_coding | -0.54 | 0.000264038 |
| AC138430.1 | AGO2 | protein_coding | -0.60 | 3.53E-05 |
| AC138430.1 | CCDC43 | protein_coding | -0.69 | 6.12E-07 |
| AC138430.1 | FDCSP | protein_coding | -0.51 | 0.000585216 |
| AC138430.1 | CFHR2 | protein_coding | 0.71 | 1.90E-07 |
| AC138430.1 | PON1 | protein_coding | 0.69 | 4.74E-07 |
| AC138430.1 | AVP | protein_coding | 0.55 | 0.000208894 |
| AC138430.1 | STXBP2 | protein_coding | -0.64 | 5.93E-06 |
| AC138430.1 | PPP4R2 | protein_coding | 0.70 | 3.85E-07 |
| AC138430.1 | ZBTB16 | protein_coding | 0.58 | 8.39E-05 |
| AC138430.1 | ZNF485 | protein_coding | -0.68 | 8.07E-07 |
| AC138430.1 | PPP1R13L | protein_coding | -0.60 | 3.76E-05 |
| AC138430.1 | SPTY2D1OS | protein_coding | -0.57 | 8.99E-05 |
| AC138430.1 | RTL10 | protein_coding | -0.58 | 6.29E-05 |
| AC138430.1 | DMGDH | protein_coding | 0.67 | 2.01E-06 |
| AC138430.1 | CEP135 | protein_coding | -0.53 | 0.000395064 |
| AC138430.1 | SH3YL1 | protein_coding | -0.59 | 5.95E-05 |
| AC138430.1 | SCIN | protein_coding | -0.54 | 0.000306215 |
| AC138430.1 | RAN | protein_coding | -0.73 | 8.18E-08 |
| AC138430.1 | UAP1L1 | protein_coding | -0.65 | 3.72E-06 |
| AC138430.1 | STC2 | protein_coding | -0.70 | 3.38E-07 |
| AC138430.1 | UTP18 | protein_coding | -0.61 | 2.74E-05 |
| AC138430.1 | ADAM32 | protein_coding | -0.68 | 1.28E-06 |
| AC138430.1 | SLC25A1 | protein_coding | 0.58 | 6.10E-05 |
| AC138430.1 | GPR162 | protein_coding | 0.60 | 3.59E-05 |
| AC138430.1 | CYP4F22 | protein_coding | 0.71 | 1.93E-07 |
| AC138430.1 | GSTCD | protein_coding | -0.55 | 0.000186421 |
| AC138430.1 | TMEM201 | protein_coding | -0.51 | 0.000690483 |
| AC138430.1 | MED25 | protein_coding | -0.65 | 5.38E-06 |
| AC138430.1 | EPB41L4B | protein_coding | 0.72 | 1.43E-07 |
| AC138430.1 | PEMT | protein_coding | 0.83 | 1.26E-11 |
| AC138430.1 | FUOM | protein_coding | 0.66 | 3.24E-06 |
| AC138430.1 | CERCAM | protein_coding | -0.76 | 9.57E-09 |
| AC138430.1 | NME2 | protein_coding | -0.61 | 1.95E-05 |
| AC138430.1 | FOXA3 | protein_coding | 0.52 | 0.000498195 |
| AC138430.1 | TIGD5 | protein_coding | -0.57 | 0.000104784 |
| AC138430.1 | LINGO4 | protein_coding | 0.63 | 9.98E-06 |
| AC138430.1 | CRIP1 | protein_coding | -0.57 | 9.96E-05 |
| AC138430.1 | UGT1A3 | protein_coding | 0.67 | 2.09E-06 |
| AC138430.1 | ZNF506 | protein_coding | -0.56 | 0.000139691 |
| AC138430.1 | CTNNA3 | protein_coding | 0.69 | 5.80E-07 |
| AC138430.1 | SP1 | protein_coding | -0.55 | 0.000226008 |
| AC138430.1 | NEUROD6 | protein_coding | 0.64 | 5.79E-06 |
| AC138430.1 | NAXD | protein_coding | 0.77 | 3.28E-09 |
| AC138430.1 | GEMIN8 | protein_coding | -0.59 | 5.23E-05 |
| AC138430.1 | RFX7 | protein_coding | -0.51 | 0.000635615 |
| AC138430.1 | TXNDC11 | protein_coding | 0.58 | 7.72E-05 |
| AC138430.1 | NFS1 | protein_coding | 0.71 | 2.25E-07 |
| AC138430.1 | FITM1 | protein_coding | 0.71 | 1.82E-07 |
| AC138430.1 | FOXC1 | protein_coding | -0.54 | 0.000255547 |
| AC138430.1 | IP6K3 | protein_coding | 0.54 | 0.000293994 |
| AC138430.1 | MCHR1 | protein_coding | 0.68 | 9.97E-07 |
| AC138430.1 | FRAS1 | protein_coding | -0.62 | 1.55E-05 |
| AC138430.1 | CSNK2A3 | protein_coding | -0.58 | 6.05E-05 |
| AC138430.1 | EIF1B | protein_coding | 0.61 | 2.41E-05 |
| AC138430.1 | NABP2 | protein_coding | -0.67 | 1.60E-06 |
| AC138430.1 | IFT140 | protein_coding | -0.70 | 3.08E-07 |
| AC138430.1 | RANBP10 | protein_coding | 0.60 | 3.75E-05 |
| AC138430.1 | MRPS22 | protein_coding | 0.60 | 3.35E-05 |
| AC138430.1 | TPPP2 | protein_coding | 0.79 | 7.37E-10 |
| AC138430.1 | TRIB1 | protein_coding | 0.65 | 5.32E-06 |
| AC138430.1 | GINS1 | protein_coding | -0.70 | 4.17E-07 |
| AC138430.1 | GHITM | protein_coding | 0.64 | 7.56E-06 |
| AC138430.1 | IFT52 | protein_coding | -0.74 | 2.91E-08 |
| AC138430.1 | SS18L1 | protein_coding | 0.73 | 5.05E-08 |
| AC138430.1 | DENND2C | protein_coding | 0.69 | 6.86E-07 |
| AC138430.1 | SPCS1 | protein_coding | 0.64 | 5.83E-06 |
| AC138430.1 | ZNF248 | protein_coding | -0.58 | 8.41E-05 |
| AC138430.1 | CASP2 | protein_coding | -0.73 | 5.55E-08 |
| AC138430.1 | ZNF174 | protein_coding | -0.55 | 0.000226471 |
| AC138430.1 | SMIM19 | protein_coding | 0.63 | 1.24E-05 |
| AC138430.1 | AKR1A1 | protein_coding | 0.53 | 0.000425488 |
| AC138430.1 | ABCC6 | protein_coding | 0.73 | 4.96E-08 |
| AC138430.1 | DNAJC16 | protein_coding | 0.83 | 2.48E-11 |
| AC138430.1 | TMEM230 | protein_coding | 0.55 | 0.000206358 |
| AC138430.1 | UCK2 | protein_coding | -0.72 | 1.27E-07 |
| AC138430.1 | BICC1 | protein_coding | -0.66 | 2.94E-06 |
| AC138430.1 | CLCN5 | protein_coding | 0.60 | 3.14E-05 |
| AC138430.1 | PLCZ1 | protein_coding | 0.62 | 1.33E-05 |
| AC138430.1 | RFX5 | protein_coding | -0.64 | 6.51E-06 |
| AC138430.1 | CECR2 | protein_coding | 0.78 | 2.31E-09 |
| AC138430.1 | DHFR | protein_coding | 0.59 | 4.69E-05 |
| AC138430.1 | VTCN1 | protein_coding | -0.53 | 0.000359233 |
| AC138430.1 | OR51E2 | protein_coding | -0.61 | 2.18E-05 |
| AC138430.1 | TMEM30A | protein_coding | 0.53 | 0.000336928 |
| AC138430.1 | HGS | protein_coding | -0.64 | 6.36E-06 |
| AC138430.1 | SYNGR3 | protein_coding | -0.50 | 0.000827701 |
| AC138430.1 | FLII | protein_coding | -0.59 | 5.46E-05 |
| AC138430.1 | SH2D4B | protein_coding | -0.57 | 9.50E-05 |
| AC138430.1 | TRIP13 | protein_coding | -0.75 | 1.77E-08 |
| AC138430.1 | INAVA | protein_coding | -0.68 | 1.26E-06 |
| AC138430.1 | TCEA3 | protein_coding | 0.74 | 4.23E-08 |
| AC138430.1 | TP53I3 | protein_coding | -0.52 | 0.000489541 |
| AC138430.1 | GYPB | protein_coding | 0.55 | 0.000179904 |
| AC138430.1 | TMEM52B | protein_coding | -0.50 | 0.000815942 |
| AC138430.1 | NUDT17 | protein_coding | -0.59 | 4.59E-05 |
| AC138430.1 | CFL2 | protein_coding | 0.73 | 8.30E-08 |
| AC138430.1 | GPM6A | protein_coding | 0.53 | 0.000322692 |
| AC138430.1 | FAM81A | protein_coding | -0.75 | 1.84E-08 |
| AC138430.1 | SFSWAP | protein_coding | -0.69 | 7.09E-07 |
| AC138430.1 | CRPPA | protein_coding | 0.76 | 8.00E-09 |
| AC138430.1 | LMNB2 | protein_coding | -0.74 | 2.58E-08 |
| AC138430.1 | ATP1B2 | protein_coding | 0.57 | 0.000119602 |
| AC138430.1 | LYRM4 | protein_coding | -0.51 | 0.000727823 |
| AC138430.1 | RIOK3 | protein_coding | 0.58 | 7.85E-05 |
| AC138430.1 | LGALS9 | protein_coding | -0.82 | 4.94E-11 |
| AC138430.1 | ANO4 | protein_coding | -0.51 | 0.000620357 |
| AC138430.1 | SLC52A2 | protein_coding | -0.76 | 8.60E-09 |
| AC138430.1 | KDM7A | protein_coding | 0.59 | 5.18E-05 |
| AC138430.1 | HJV | protein_coding | 0.66 | 2.69E-06 |
| AC138430.1 | TRIM52 | protein_coding | -0.57 | 0.00011543 |
| AC138430.1 | ZNF785 | protein_coding | -0.52 | 0.000463383 |
| AC138430.1 | SKA3 | protein_coding | -0.66 | 2.38E-06 |
| AC138430.1 | SLC10A3 | protein_coding | -0.74 | 2.95E-08 |
| AC138430.1 | CTSZ | protein_coding | 0.52 | 0.000468551 |
| AC138430.1 | RIC8A | protein_coding | -0.66 | 2.57E-06 |
| AC138430.1 | TPMT | protein_coding | 0.70 | 2.91E-07 |
| AC138430.1 | SAMD15 | protein_coding | -0.63 | 9.44E-06 |
| AC138430.1 | HDDC2 | protein_coding | -0.57 | 9.00E-05 |
| AC138430.1 | SENP8 | protein_coding | 0.59 | 5.74E-05 |
| AC138430.1 | HARS2 | protein_coding | -0.54 | 0.000279287 |
| AC138430.1 | SELENOM | protein_coding | -0.64 | 6.52E-06 |
| AC138430.1 | TMEM120A | protein_coding | 0.50 | 0.000819033 |
| AC138430.1 | THOC5 | protein_coding | -0.59 | 5.50E-05 |
| AC138430.1 | CARMIL1 | protein_coding | -0.60 | 3.42E-05 |
| AC138430.1 | THY1 | protein_coding | -0.66 | 2.89E-06 |
| AC138430.1 | CPNE2 | protein_coding | -0.51 | 0.000724302 |
| AC138430.1 | SLCO1B3-SLCO1B7 | protein_coding | 0.73 | 7.06E-08 |
| AC138430.1 | HP | protein_coding | 0.57 | 0.000115246 |
| AC138430.1 | LSM2 | protein_coding | -0.56 | 0.000157927 |
| AC138430.1 | C21orf58 | protein_coding | -0.55 | 0.000183941 |
| AC138430.1 | SSRP1 | protein_coding | -0.60 | 3.50E-05 |
| AC138430.1 | ACTL10 | protein_coding | -0.70 | 4.21E-07 |
| AC138430.1 | TNFRSF18 | protein_coding | -0.67 | 1.62E-06 |
| AC138430.1 | GFRA1 | protein_coding | 0.56 | 0.000138047 |
| AC138430.1 | ZNF330 | protein_coding | 0.77 | 5.00E-09 |
| AC138430.1 | AL139011.2 | protein_coding | 0.75 | 1.49E-08 |
| AC138430.1 | TMEM170B | protein_coding | 0.78 | 1.43E-09 |
| AC138430.1 | DIRAS3 | protein_coding | 0.59 | 4.33E-05 |
| AC138430.1 | FNBP4 | protein_coding | -0.54 | 0.00029136 |
| AC138430.1 | ADM5 | protein_coding | -0.63 | 1.17E-05 |
| AC138430.1 | ATF6B | protein_coding | -0.55 | 0.00020473 |
| AC138430.1 | SMIM8 | protein_coding | 0.61 | 2.55E-05 |
| AC138430.1 | TK1 | protein_coding | -0.58 | 8.12E-05 |
| AC138430.1 | PMPCA | protein_coding | 0.72 | 1.42E-07 |
| AC138430.1 | PLAU | protein_coding | -0.52 | 0.000520936 |
| AC138430.1 | APOL6 | protein_coding | 0.56 | 0.000144713 |
| AC138430.1 | GNRH1 | protein_coding | -0.54 | 0.000300089 |
| AC138430.1 | HOXD9 | protein_coding | -0.52 | 0.000550732 |
| AC138430.1 | SSR2 | protein_coding | -0.58 | 6.10E-05 |
| AC138430.1 | GLG1 | protein_coding | -0.54 | 0.000280413 |
| AC138430.1 | TFPI | protein_coding | 0.65 | 3.52E-06 |
| AC138430.1 | CLEC4G | protein_coding | 0.56 | 0.00014518 |
| AC138430.1 | LINC00672 | protein_coding | -0.59 | 4.23E-05 |
| AC138430.1 | CDC37L1 | protein_coding | 0.80 | 5.15E-10 |
| AC138430.1 | LRP1 | protein_coding | 0.63 | 9.13E-06 |
| AC138430.1 | NAPA | protein_coding | 0.60 | 3.31E-05 |
| AC138430.1 | FAM217B | protein_coding | -0.58 | 7.24E-05 |
| AC138430.1 | MSTO1 | protein_coding | -0.68 | 1.01E-06 |
| AC138430.1 | FANK1 | protein_coding | -0.66 | 2.40E-06 |
| AC138430.1 | NELFA | protein_coding | -0.65 | 3.88E-06 |
| AC138430.1 | C11orf45 | protein_coding | -0.54 | 0.000244231 |
| AC138430.1 | HEY2 | protein_coding | 0.73 | 5.96E-08 |
| AC138430.1 | COL4A1 | protein_coding | -0.50 | 0.000855883 |
| AC138430.1 | FAM49B | protein_coding | -0.71 | 2.03E-07 |
| AC138430.1 | DEGS2 | protein_coding | -0.54 | 0.000314564 |
| AC138430.1 | HGD | protein_coding | 0.58 | 7.00E-05 |
| AC138430.1 | CENPE | protein_coding | -0.65 | 5.18E-06 |
| AC138430.1 | NR0B2 | protein_coding | 0.62 | 1.47E-05 |
| AC138430.1 | APCDD1 | protein_coding | -0.60 | 3.65E-05 |
| AC138430.1 | KIF11 | protein_coding | -0.70 | 3.74E-07 |
| AC138430.1 | MGMT | protein_coding | 0.71 | 2.39E-07 |
| AC138430.1 | HMGCS1 | protein_coding | 0.73 | 7.49E-08 |
| AC138430.1 | MUS81 | protein_coding | -0.53 | 0.00038902 |
| AC138430.1 | SLC9A5 | protein_coding | -0.60 | 3.63E-05 |
| AC138430.1 | GCKR | protein_coding | 0.67 | 2.01E-06 |
| AC138430.1 | GLTPD2 | protein_coding | 0.77 | 3.33E-09 |
| AC138430.1 | SLC25A27 | protein_coding | 0.63 | 8.85E-06 |
| AC138430.1 | EXO1 | protein_coding | -0.66 | 2.74E-06 |
| AC138430.1 | RAVER2 | protein_coding | -0.67 | 2.11E-06 |
| AC138430.1 | IL17RD | protein_coding | -0.64 | 6.30E-06 |
| AC138430.1 | LTB | protein_coding | -0.58 | 6.29E-05 |
| AC138430.1 | TCTEX1D1 | protein_coding | 0.72 | 9.40E-08 |
| AC138430.1 | DAXX | protein_coding | -0.57 | 8.58E-05 |
| AC138430.1 | KRTAP5-1 | protein_coding | -0.62 | 1.74E-05 |
| AC138430.1 | PTMS | protein_coding | 0.65 | 3.98E-06 |
| AC138430.1 | WIZ | protein_coding | -0.53 | 0.000386829 |
| AC138430.1 | AMHR2 | protein_coding | 0.70 | 3.26E-07 |
| AC138430.1 | CENPM | protein_coding | -0.74 | 3.70E-08 |
| AC138430.1 | SMPD2 | protein_coding | -0.52 | 0.000535756 |
| AC138430.1 | ARHGEF39 | protein_coding | -0.62 | 1.68E-05 |
| AC138430.1 | MAP2K1 | protein_coding | 0.58 | 6.29E-05 |
| AC138430.1 | NCEH1 | protein_coding | -0.80 | 2.49E-10 |
| AC138430.1 | KLF12 | protein_coding | 0.58 | 6.14E-05 |
| AC138430.1 | MAN2B1 | protein_coding | -0.58 | 7.33E-05 |
| AC138430.1 | RBPMS | protein_coding | -0.54 | 0.000265296 |
| AC138430.1 | IQCC | protein_coding | -0.61 | 1.92E-05 |
| AC138430.1 | DOLPP1 | protein_coding | 0.73 | 5.68E-08 |
| AC138430.1 | LDLRAP1 | protein_coding | 0.60 | 3.32E-05 |
| AC138430.1 | ACOT2 | protein_coding | 0.85 | 1.86E-12 |
| AC138430.1 | STAB2 | protein_coding | 0.57 | 0.000112832 |
| AC138430.1 | GPRC5B | protein_coding | -0.53 | 0.000340791 |
| AC138430.1 | NARS2 | protein_coding | 0.79 | 6.37E-10 |
| AC138430.1 | RNF4 | protein_coding | -0.60 | 3.66E-05 |
| AC138430.1 | C15orf39 | protein_coding | -0.61 | 2.03E-05 |
| AC138430.1 | UBL3 | protein_coding | 0.70 | 4.70E-07 |
| AC138430.1 | ABR | protein_coding | -0.78 | 1.87E-09 |
| AC138430.1 | PPP2R2C | protein_coding | -0.52 | 0.000437341 |
| AC138430.1 | ZGPAT | protein_coding | 0.53 | 0.000336864 |
| AC138430.1 | MT-CO2 | protein_coding | 0.71 | 2.52E-07 |
| AC138430.1 | C6orf141 | protein_coding | -0.54 | 0.000300684 |
| AC138430.1 | SH2D3A | protein_coding | -0.80 | 4.89E-10 |
| AC138430.1 | MGAT5 | protein_coding | -0.77 | 4.17E-09 |
| AC138430.1 | CGAS | protein_coding | -0.60 | 3.81E-05 |
| AC138430.1 | STARD3NL | protein_coding | -0.62 | 1.49E-05 |
| AC138430.1 | CREB5 | protein_coding | -0.61 | 2.41E-05 |
| AC138430.1 | ATAD5 | protein_coding | -0.64 | 6.96E-06 |
| AC138430.1 | SLFN5 | protein_coding | -0.57 | 0.000114073 |
| AC138430.1 | KRTCAP3 | protein_coding | -0.59 | 4.71E-05 |
| AC138430.1 | CWH43 | protein_coding | 0.77 | 2.85E-09 |
| AC138430.1 | GALNT18 | protein_coding | -0.59 | 4.96E-05 |
| AC138430.1 | LACTB | protein_coding | 0.54 | 0.000263191 |
| AC138430.1 | SLC35A5 | protein_coding | 0.56 | 0.000137679 |
| AC138430.1 | KSR2 | protein_coding | -0.68 | 1.23E-06 |
| AC138430.1 | PTPN14 | protein_coding | -0.72 | 1.15E-07 |
| AC138430.1 | DUSP1 | protein_coding | 0.64 | 7.50E-06 |
| AC138430.1 | BCAN | protein_coding | -0.68 | 8.55E-07 |
| AC138430.1 | ARHGAP1 | protein_coding | -0.56 | 0.000125759 |
| AC138430.1 | SLCO1A2 | protein_coding | 0.52 | 0.000459321 |
| AC138430.1 | CEP295NL | protein_coding | -0.58 | 8.55E-05 |
| AC138430.1 | PI4KB | protein_coding | -0.51 | 0.000666025 |
| AC138430.1 | CD81 | protein_coding | 0.51 | 0.000589554 |
| AC138430.1 | ANKS4B | protein_coding | 0.64 | 5.91E-06 |
| AC138430.1 | MRPL44 | protein_coding | 0.57 | 0.000119877 |
| AC138430.1 | SEC63 | protein_coding | 0.54 | 0.000252049 |
| AC138430.1 | PODNL1 | protein_coding | -0.51 | 0.000625559 |
| AC138430.1 | MTBP | protein_coding | -0.63 | 1.13E-05 |
| AC138430.1 | GINS4 | protein_coding | -0.64 | 7.92E-06 |
| AC138430.1 | GABARAPL1 | protein_coding | 0.63 | 8.64E-06 |
| AC138430.1 | RAB25 | protein_coding | -0.66 | 3.29E-06 |
| AC138430.1 | KAAG1 | protein_coding | -0.56 | 0.000133783 |
| AC138430.1 | MT-CYB | protein_coding | 0.72 | 1.06E-07 |
| AC138430.1 | SAT2 | protein_coding | 0.73 | 5.44E-08 |
| AC138430.1 | LDLRAD2 | protein_coding | -0.54 | 0.000236219 |
| AC138430.1 | NECAB2 | protein_coding | 0.62 | 1.68E-05 |
| AC138430.1 | CYP2U1 | protein_coding | 0.61 | 2.34E-05 |
| AC138430.1 | ALB | protein_coding | 0.63 | 9.44E-06 |
| AC138430.1 | EVI5 | protein_coding | 0.53 | 0.000391391 |
| AC138430.1 | FBXO40 | protein_coding | 0.61 | 2.42E-05 |
| AC138430.1 | BIRC7 | protein_coding | -0.53 | 0.000350497 |
| AC138430.1 | ZNF431 | protein_coding | -0.73 | 7.23E-08 |
| AC138430.1 | WDR46 | protein_coding | -0.53 | 0.000336749 |
| AC138430.1 | SCMH1 | protein_coding | -0.53 | 0.000324338 |
| AC138430.1 | MT-ATP6 | protein_coding | 0.73 | 6.77E-08 |
| AC138430.1 | TENT5A | protein_coding | 0.63 | 1.00E-05 |
| AC138430.1 | KIF25 | protein_coding | 0.58 | 6.33E-05 |
| AC138430.1 | RDH11 | protein_coding | 0.67 | 1.53E-06 |
| AC138430.1 | H2AW | protein_coding | -0.68 | 1.09E-06 |
| AC138430.1 | TMEM45A | protein_coding | 0.57 | 8.65E-05 |
| AC138430.1 | HDAC9 | protein_coding | -0.65 | 4.12E-06 |
| AC138430.1 | GLYATL1 | protein_coding | 0.66 | 2.75E-06 |
| AC138430.1 | MEX3A | protein_coding | -0.53 | 0.000340753 |
| AC138430.1 | CFAP45 | protein_coding | -0.73 | 6.63E-08 |
| AC138430.1 | A1CF | protein_coding | 0.59 | 4.34E-05 |
| AC138430.1 | CYP4F3 | protein_coding | 0.72 | 1.49E-07 |
| AC138430.1 | NDRG3 | protein_coding | -0.72 | 8.88E-08 |
| AC138430.1 | DUSP6 | protein_coding | 0.60 | 4.14E-05 |
| AC138430.1 | KCTD7 | protein_coding | -0.63 | 8.95E-06 |
| AC138430.1 | IL13 | protein_coding | 0.51 | 0.000711721 |
| AC138430.1 | ANGPTL6 | protein_coding | 0.66 | 3.03E-06 |
| AC138430.1 | UBE2A | protein_coding | -0.59 | 5.46E-05 |
| AC138430.1 | CCDC126 | protein_coding | 0.63 | 9.07E-06 |
| AC138430.1 | COX17 | protein_coding | 0.56 | 0.000131883 |
| AC138430.1 | NT5C3A | protein_coding | -0.62 | 1.50E-05 |
| AC138430.1 | TRIM65 | protein_coding | -0.79 | 1.01E-09 |
| AC138430.1 | FANCD2 | protein_coding | -0.70 | 3.30E-07 |
| AC138430.1 | MLXIPL | protein_coding | 0.71 | 2.17E-07 |
| AC138430.1 | SLC5A12 | protein_coding | 0.67 | 1.97E-06 |
| AC138430.1 | GRIN2A | protein_coding | -0.68 | 9.35E-07 |
| AC138430.1 | HLX | protein_coding | 0.59 | 4.94E-05 |
| AC138430.1 | SERPINA10 | protein_coding | 0.65 | 3.49E-06 |
| AC138430.1 | SLC7A7 | protein_coding | -0.65 | 4.06E-06 |
| AC138430.1 | HBM | protein_coding | 0.56 | 0.000161362 |
| AC138430.1 | CCDC6 | protein_coding | -0.70 | 4.55E-07 |
| AC138430.1 | PEX19 | protein_coding | 0.74 | 2.68E-08 |
| AC138430.1 | SSH2 | protein_coding | -0.61 | 2.49E-05 |
| AC138430.1 | TRIOBP | protein_coding | -0.53 | 0.000356903 |
| AC138430.1 | NRIP1 | protein_coding | 0.61 | 2.20E-05 |
| AC138430.1 | TES | protein_coding | -0.78 | 1.81E-09 |
| AC138430.1 | ZC3H3 | protein_coding | -0.52 | 0.000449885 |
| AC138430.1 | PTPRE | protein_coding | -0.63 | 1.07E-05 |
| AC138430.1 | COA1 | protein_coding | -0.51 | 0.000616782 |
| AC138430.1 | CIP2A | protein_coding | -0.73 | 6.03E-08 |
| AC138430.1 | ABI1 | protein_coding | -0.57 | 8.59E-05 |
| AC138430.1 | SNURF | protein_coding | -0.61 | 2.59E-05 |
| AC138430.1 | BMPER | protein_coding | 0.61 | 2.13E-05 |
| AC138430.1 | PYGO2 | protein_coding | -0.61 | 2.10E-05 |
| AC138430.1 | SLC39A9 | protein_coding | 0.60 | 3.40E-05 |
| AC138430.1 | ARHGEF3 | protein_coding | -0.67 | 1.35E-06 |
| AC138430.1 | TTK | protein_coding | -0.66 | 2.43E-06 |
| AC138430.1 | SPDL1 | protein_coding | -0.76 | 7.89E-09 |
| AC138430.1 | EVX1 | protein_coding | -0.51 | 0.000674565 |
| AC138430.1 | ACSF2 | protein_coding | 0.65 | 3.54E-06 |
| AC138430.1 | PPHLN1 | protein_coding | -0.66 | 3.04E-06 |
| AC138430.1 | SERPINB6 | protein_coding | -0.58 | 6.48E-05 |
| AC138430.1 | UBE2M | protein_coding | -0.50 | 0.000786731 |
| AC138430.1 | ALDH9A1 | protein_coding | 0.80 | 4.44E-10 |
| AC138430.1 | NME9 | protein_coding | -0.55 | 0.000178241 |
| AC138430.1 | SERPINE2 | protein_coding | -0.53 | 0.000321604 |
| AC138430.1 | RAB6B | protein_coding | -0.60 | 3.85E-05 |
| AC138430.1 | SCRIB | protein_coding | -0.53 | 0.00032237 |
| AC138430.1 | CLEC1B | protein_coding | 0.67 | 1.81E-06 |
| AC138430.1 | COX19 | protein_coding | -0.68 | 8.90E-07 |
| AC138430.1 | RBM33 | protein_coding | -0.51 | 0.000629956 |
| AC138430.1 | NOP2 | protein_coding | -0.66 | 3.20E-06 |
| AC138430.1 | FAHD2A | protein_coding | 0.76 | 1.14E-08 |
| AC138430.1 | EIF2S3 | protein_coding | -0.57 | 0.000116831 |
| AC138430.1 | AUH | protein_coding | 0.75 | 2.36E-08 |
| AC138430.1 | RCC2 | protein_coding | -0.83 | 3.22E-11 |
| AC138430.1 | INCENP | protein_coding | -0.52 | 0.000447014 |
| AC138430.1 | MEX3B | protein_coding | -0.61 | 2.20E-05 |
| AC138430.1 | ST14 | protein_coding | -0.76 | 1.11E-08 |
| AC138430.1 | HSD3B1 | protein_coding | 0.71 | 2.10E-07 |
| AC138430.1 | ZSWIM1 | protein_coding | -0.62 | 1.79E-05 |
| AC138430.1 | MROH1 | protein_coding | -0.58 | 6.75E-05 |
| AC138430.1 | TANC2 | protein_coding | -0.65 | 4.67E-06 |
| AC138430.1 | GRPEL2 | protein_coding | -0.64 | 5.76E-06 |
| AC138430.1 | MBNL2 | protein_coding | 0.55 | 0.000178112 |
| AC138430.1 | ENPP1 | protein_coding | 0.82 | 8.88E-11 |
| AC138430.1 | CPS1 | protein_coding | 0.64 | 6.14E-06 |
| AC138430.1 | SLCO1B7 | protein_coding | 0.73 | 7.00E-08 |
| AC138430.1 | BCL2L1 | protein_coding | -0.53 | 0.000367189 |
| AC138430.1 | MTA3 | protein_coding | -0.61 | 2.19E-05 |
| AC138430.1 | NDC1 | protein_coding | -0.52 | 0.000529579 |
| AC138430.1 | MAP3K12 | protein_coding | -0.56 | 0.000154971 |
| AC138430.1 | C9orf64 | protein_coding | 0.67 | 1.75E-06 |
| AC138430.1 | ETV6 | protein_coding | -0.65 | 5.03E-06 |
| AC138430.1 | AQP7 | protein_coding | 0.65 | 4.98E-06 |
| AC138430.1 | RCAN3 | protein_coding | -0.70 | 4.14E-07 |
| AC138430.1 | GCM1 | protein_coding | -0.57 | 9.97E-05 |
| AC138430.1 | SLC39A12 | protein_coding | 0.53 | 0.000422065 |
| AC138430.1 | RPL8 | protein_coding | -0.55 | 0.000174137 |
| AC138430.1 | PLVAP | protein_coding | -0.67 | 1.95E-06 |
| AC138430.1 | DCUN1D5 | protein_coding | -0.51 | 0.000710744 |
| AC138430.1 | MOB3B | protein_coding | -0.64 | 7.88E-06 |
| AC138430.1 | PZP | protein_coding | 0.58 | 7.50E-05 |
| AC138430.1 | LCMT1 | protein_coding | -0.74 | 4.44E-08 |
| AC138430.1 | EVC2 | protein_coding | -0.69 | 5.96E-07 |
| AC138430.1 | LARS2 | protein_coding | 0.75 | 1.52E-08 |
| AC138430.1 | LEPROTL1 | protein_coding | -0.53 | 0.000336812 |
| AC138430.1 | COL9A1 | protein_coding | -0.55 | 0.000220302 |
| AC138430.1 | 43892 | protein_coding | 0.69 | 5.14E-07 |
| AC138430.1 | SLC47A1 | protein_coding | 0.73 | 7.51E-08 |
| AC138430.1 | FBXL18 | protein_coding | -0.53 | 0.000334068 |
| AC138430.1 | NR1H4 | protein_coding | 0.54 | 0.000285927 |
| AC138430.1 | DROSHA | protein_coding | -0.65 | 3.46E-06 |
| AC138430.1 | NUP85 | protein_coding | -0.53 | 0.000346637 |
| AC138430.1 | SULT2A1 | protein_coding | 0.63 | 1.19E-05 |
| AC138430.1 | MPDZ | protein_coding | 0.71 | 2.17E-07 |
| AC138430.1 | NOTCH3 | protein_coding | -0.67 | 1.53E-06 |
| AC138430.1 | OXSM | protein_coding | 0.70 | 4.61E-07 |
| AC138430.1 | ZFP41 | protein_coding | -0.68 | 8.25E-07 |
| AC138430.1 | WDR62 | protein_coding | -0.64 | 8.13E-06 |
| AC138430.1 | COPS7B | protein_coding | -0.65 | 3.52E-06 |
| AC138430.1 | C11orf71 | protein_coding | 0.68 | 9.23E-07 |
| AC138430.1 | STAT5B | protein_coding | 0.50 | 0.000782266 |
| AC138430.1 | FBXO41 | protein_coding | -0.56 | 0.000159771 |
| AC138430.1 | FMN1 | protein_coding | -0.55 | 0.000209455 |
| AC138430.1 | DDTL | protein_coding | 0.71 | 2.03E-07 |
| AC138430.1 | TMEM106C | protein_coding | -0.63 | 1.11E-05 |
| AC138430.1 | IMPA1 | protein_coding | 0.58 | 6.71E-05 |
| AC138430.1 | HPX | protein_coding | 0.63 | 1.06E-05 |
| AC138430.1 | NFE2L3 | protein_coding | -0.75 | 1.63E-08 |
| AC138430.1 | KCTD10 | protein_coding | -0.75 | 1.29E-08 |
| AC138430.1 | BMP5 | protein_coding | 0.51 | 0.000662329 |
| AC138430.1 | DHRS4L2 | protein_coding | 0.68 | 1.30E-06 |
| AC138430.1 | ATXN7L3 | protein_coding | -0.67 | 1.82E-06 |
| AC138430.1 | SLC19A2 | protein_coding | 0.56 | 0.000139299 |
| AC138430.1 | ITGB8 | protein_coding | -0.65 | 4.94E-06 |
| AC138430.1 | SCO1 | protein_coding | 0.70 | 3.31E-07 |
| AC138430.1 | AGT | protein_coding | 0.51 | 0.000748717 |
| AC138430.1 | ABHD15 | protein_coding | 0.77 | 5.28E-09 |
| AC138430.1 | ZSCAN20 | protein_coding | -0.68 | 1.04E-06 |
| AC138430.1 | CCDC97 | protein_coding | -0.74 | 3.14E-08 |
| AC138430.1 | FZD4 | protein_coding | 0.52 | 0.000566644 |
| AC138430.1 | LAT2 | protein_coding | -0.59 | 5.63E-05 |
| AC138430.1 | IL3 | protein_coding | 0.60 | 3.83E-05 |
| AC138430.1 | SMARCB1 | protein_coding | -0.51 | 0.0007308 |
| AC138430.1 | SALL2 | protein_coding | -0.58 | 7.00E-05 |
| AC138430.1 | GARNL3 | protein_coding | 0.58 | 7.55E-05 |
| AC138430.1 | ATN1 | protein_coding | -0.72 | 1.00E-07 |
| AC138430.1 | HADH | protein_coding | 0.89 | 7.72E-15 |
| AC138430.1 | JAK3 | protein_coding | -0.58 | 6.01E-05 |
| AC138430.1 | RBM4 | protein_coding | -0.67 | 1.97E-06 |
| AC138430.1 | CDH15 | protein_coding | 0.66 | 2.85E-06 |
| AC138430.1 | HADHB | protein_coding | 0.67 | 1.60E-06 |
| AC138430.1 | HBA1 | protein_coding | 0.58 | 6.37E-05 |
| AC138430.1 | CYP39A1 | protein_coding | 0.78 | 2.31E-09 |
| AC138430.1 | MIF4GD | protein_coding | -0.54 | 0.000246017 |
| AC138430.1 | OCEL1 | protein_coding | 0.63 | 1.01E-05 |
| AC138430.1 | RPS2 | protein_coding | -0.60 | 3.57E-05 |
| AC138430.1 | MCM7 | protein_coding | -0.67 | 1.88E-06 |
| AC138430.1 | CHPT1 | protein_coding | 0.59 | 5.44E-05 |
| AC138430.1 | MELK | protein_coding | -0.69 | 6.62E-07 |
| AC138430.1 | RNF165 | protein_coding | 0.73 | 7.55E-08 |
| AC138430.1 | GPR146 | protein_coding | 0.71 | 2.37E-07 |
| AC138430.1 | MT1HL1 | protein_coding | 0.68 | 9.87E-07 |
| AC138430.1 | AC013489.1 | protein_coding | -0.55 | 0.000187086 |
| AC138430.1 | NPIPB13 | protein_coding | -0.52 | 0.000530854 |
| AC138430.1 | TMEM242 | protein_coding | 0.59 | 4.28E-05 |
| AC138430.1 | COQ3 | protein_coding | 0.52 | 0.000535238 |
| AC138430.1 | SERPINA12 | protein_coding | 0.69 | 6.84E-07 |
| AC138430.1 | SNX29 | protein_coding | -0.52 | 0.000447053 |
| AC138430.1 | KIF26B | protein_coding | -0.67 | 1.60E-06 |
| AC138430.1 | REEP2 | protein_coding | -0.57 | 8.75E-05 |
| AC138430.1 | SLC13A5 | protein_coding | 0.66 | 2.63E-06 |
| AC138430.1 | SHD | protein_coding | 0.64 | 8.21E-06 |
| AC138430.1 | PTGR2 | protein_coding | 0.58 | 8.12E-05 |
| AC138430.1 | THRB | protein_coding | 0.71 | 2.64E-07 |
| AC138430.1 | DPF1 | protein_coding | -0.65 | 5.16E-06 |
| AC138430.1 | ADK | protein_coding | 0.70 | 4.07E-07 |
| AC138430.1 | CLU | protein_coding | 0.60 | 4.08E-05 |
| AC138430.1 | TBX10 | protein_coding | 0.65 | 5.15E-06 |
| AC138430.1 | DEPDC1B | protein_coding | -0.60 | 2.95E-05 |
| AC138430.1 | SRPX2 | protein_coding | -0.50 | 0.0007859 |
| AC138430.1 | RNLS | protein_coding | 0.69 | 7.83E-07 |
| AC138430.1 | LARP4B | protein_coding | -0.69 | 7.62E-07 |
| AC138430.1 | TNNT2 | protein_coding | -0.58 | 7.95E-05 |
| AC138430.1 | ATP2C2 | protein_coding | -0.59 | 4.51E-05 |
| AC138430.1 | C19orf12 | protein_coding | 0.71 | 2.37E-07 |
| AC138430.1 | PNPO | protein_coding | 0.70 | 4.38E-07 |
| AC138430.1 | ECHDC2 | protein_coding | 0.76 | 8.21E-09 |
| AC138430.1 | VANGL1 | protein_coding | -0.56 | 0.000139289 |
| AC138430.1 | INKA2 | protein_coding | -0.60 | 3.04E-05 |
| AC138430.1 | SLC22A3 | protein_coding | 0.50 | 0.000810506 |
| AC138430.1 | UNC5A | protein_coding | -0.61 | 2.46E-05 |
| AC138430.1 | ABCC9 | protein_coding | 0.64 | 7.42E-06 |
| AC138430.1 | ASCL5 | protein_coding | -0.58 | 6.27E-05 |
| AC138430.1 | EEFSEC | protein_coding | 0.61 | 2.21E-05 |
| AC138430.1 | MOB3A | protein_coding | -0.76 | 1.05E-08 |
| AC138430.1 | UCK1 | protein_coding | 0.59 | 4.66E-05 |
| AC138430.1 | LRRC49 | protein_coding | -0.65 | 4.59E-06 |
| AC138430.1 | XPA | protein_coding | 0.55 | 0.000170512 |
| AC138430.1 | HNRNPA0 | protein_coding | -0.64 | 5.83E-06 |
| AC138430.1 | GTF3C2 | protein_coding | -0.71 | 1.67E-07 |
| AC138430.1 | MFSD10 | protein_coding | -0.81 | 1.47E-10 |
| AC138430.1 | DDX52 | protein_coding | -0.51 | 0.000735347 |
| AC138430.1 | HK1 | protein_coding | -0.71 | 2.18E-07 |
| AC138430.1 | PHYKPL | protein_coding | 0.60 | 3.08E-05 |
| AC138430.1 | ZNF275 | protein_coding | 0.60 | 3.89E-05 |
| AC138430.1 | AKT1 | protein_coding | 0.60 | 4.01E-05 |
| AC138430.1 | ACOT13 | protein_coding | 0.75 | 1.33E-08 |
| AC138430.1 | NEU4 | protein_coding | 0.70 | 4.37E-07 |
| AC138430.1 | C4BPB | protein_coding | 0.64 | 6.13E-06 |
| AC138430.1 | CDYL | protein_coding | -0.52 | 0.00050771 |
| AC138430.1 | PDLIM7 | protein_coding | -0.73 | 5.64E-08 |
| AC138430.1 | LDB2 | protein_coding | 0.52 | 0.000577528 |
| AC138430.1 | INO80E | protein_coding | -0.59 | 4.92E-05 |
| AC138430.1 | YWHAB | protein_coding | -0.58 | 8.21E-05 |
| AC138430.1 | RYBP | protein_coding | 0.56 | 0.000125591 |
| AC138430.1 | CDC7 | protein_coding | -0.64 | 6.37E-06 |
| AC138430.1 | ZDHHC1 | protein_coding | -0.71 | 2.01E-07 |
| AC138430.1 | YIPF4 | protein_coding | 0.62 | 1.59E-05 |
| AC138430.1 | TBL2 | protein_coding | -0.58 | 8.16E-05 |
| AC138430.1 | STRADA | protein_coding | -0.52 | 0.000565786 |
| AC138430.1 | FKBP10 | protein_coding | -0.73 | 6.47E-08 |
| AC138430.1 | S100A5 | protein_coding | -0.54 | 0.000304085 |
| AC138430.1 | TMEM33 | protein_coding | 0.54 | 0.000244336 |
| AC138430.1 | COLCA2 | protein_coding | -0.63 | 1.22E-05 |
| AC138430.1 | SLC38A3 | protein_coding | 0.75 | 1.53E-08 |
| AC138430.1 | SNTB1 | protein_coding | 0.56 | 0.000146293 |
| AC138430.1 | TRIM59 | protein_coding | -0.80 | 2.98E-10 |
| AC138430.1 | PON3 | protein_coding | 0.69 | 5.09E-07 |
| AC138430.1 | RPP14 | protein_coding | 0.80 | 5.40E-10 |
| AC138430.1 | ZNF207 | protein_coding | -0.61 | 2.16E-05 |
| AC138430.1 | SLC13A1 | protein_coding | -0.54 | 0.000250605 |
| AC138430.1 | MEAK7 | protein_coding | -0.67 | 1.39E-06 |
| AC138430.1 | MT-CO1 | protein_coding | 0.67 | 1.72E-06 |
| AC138430.1 | SEPSECS | protein_coding | 0.76 | 7.72E-09 |
| AC138430.1 | FOXD4L1 | protein_coding | -0.65 | 4.43E-06 |
| AC138430.1 | MYLK2 | protein_coding | -0.62 | 1.60E-05 |
| AC138430.1 | MTMR11 | protein_coding | -0.62 | 1.50E-05 |
| AC138430.1 | ADH1C | protein_coding | 0.60 | 3.30E-05 |
| AC138430.1 | ARG1 | protein_coding | 0.68 | 1.21E-06 |
| AC138430.1 | BEST3 | protein_coding | -0.59 | 4.31E-05 |
| AC138430.1 | SLC6A1 | protein_coding | 0.76 | 9.44E-09 |
| AC138430.1 | CTH | protein_coding | 0.79 | 1.20E-09 |
| AC138430.1 | CTBP2 | protein_coding | -0.72 | 8.63E-08 |
| AC138430.1 | ELFN1 | protein_coding | 0.77 | 3.46E-09 |
| AC138430.1 | SPAG1 | protein_coding | -0.55 | 0.000217872 |
| AC138430.1 | TST | protein_coding | 0.65 | 3.58E-06 |
| AC138430.1 | SLCO5A1 | protein_coding | -0.66 | 2.68E-06 |
| AC138430.1 | NUP62 | protein_coding | -0.73 | 6.53E-08 |
| AC138430.1 | RAB38 | protein_coding | -0.51 | 0.000656655 |
| AC138430.1 | UNC119B | protein_coding | -0.64 | 6.05E-06 |
| AC138430.1 | RD3L | protein_coding | 0.78 | 2.64E-09 |
| AC138430.1 | CYP4A22 | protein_coding | 0.61 | 2.07E-05 |
| AC138430.1 | CYP3A7 | protein_coding | 0.68 | 9.15E-07 |
| AC138430.1 | DDN | protein_coding | -0.51 | 0.000651712 |
| AC138430.1 | UIMC1 | protein_coding | -0.68 | 1.08E-06 |
| AC138430.1 | GSTP1 | protein_coding | -0.62 | 1.35E-05 |
| AC138430.1 | AGTR1 | protein_coding | 0.59 | 5.97E-05 |
| AC138430.1 | LIMD1 | protein_coding | 0.53 | 0.000336925 |
| AC138430.1 | RBMX2 | protein_coding | -0.67 | 1.63E-06 |
| AC138430.1 | LAMP1 | protein_coding | 0.69 | 7.54E-07 |
| AC138430.1 | NOL4L | protein_coding | -0.60 | 2.89E-05 |
| AC138430.1 | MSRA | protein_coding | 0.81 | 1.07E-10 |
| AC138430.1 | MICAL1 | protein_coding | -0.76 | 9.44E-09 |
| AC138430.1 | SCAMP4 | protein_coding | -0.51 | 0.000593112 |
| AC138430.1 | SLC39A14 | protein_coding | 0.75 | 2.40E-08 |
| AC138430.1 | RGPD3 | protein_coding | 0.68 | 1.06E-06 |
| AC138430.1 | MARS1 | protein_coding | -0.53 | 0.000318962 |
| AC138430.1 | HMGCR | protein_coding | 0.61 | 2.25E-05 |
| AC138430.1 | PKMYT1 | protein_coding | -0.75 | 2.35E-08 |
| AC138430.1 | ZNF74 | protein_coding | -0.68 | 1.05E-06 |
| AC138430.1 | DHX40 | protein_coding | -0.54 | 0.000237838 |
| AC138430.1 | PEA15 | protein_coding | -0.51 | 0.00062149 |
| AC138430.1 | NDUFS1 | protein_coding | 0.68 | 9.94E-07 |
| AC138430.1 | N4BP2L1 | protein_coding | 0.79 | 1.14E-09 |
| AC138430.1 | CTBS | protein_coding | 0.65 | 3.54E-06 |
| AC138430.1 | SOGA1 | protein_coding | -0.84 | 1.05E-11 |
| AC138430.1 | KCNK12 | protein_coding | -0.67 | 1.97E-06 |
| AC138430.1 | ZP3 | protein_coding | -0.73 | 6.24E-08 |
| AC138430.1 | TSPAN16 | protein_coding | -0.55 | 0.000217771 |
| AC138430.1 | BCAS4 | protein_coding | -0.76 | 7.85E-09 |
| AC138430.1 | RAD54L | protein_coding | -0.76 | 9.01E-09 |
| AC138430.1 | CRAMP1 | protein_coding | -0.66 | 2.84E-06 |
| AC138430.1 | GPRC5C | protein_coding | 0.58 | 6.70E-05 |
| AC138430.1 | ACOT9 | protein_coding | -0.56 | 0.000128832 |
| AC138430.1 | PRODH2 | protein_coding | 0.60 | 3.96E-05 |
| AC138430.1 | SLC7A2 | protein_coding | 0.75 | 1.88E-08 |
| AC138430.1 | NDFIP1 | protein_coding | 0.66 | 3.19E-06 |
| AC138430.1 | ORC1 | protein_coding | -0.70 | 3.93E-07 |
| AC138430.1 | FAM13A | protein_coding | 0.74 | 3.36E-08 |
| AC138430.1 | CNOT11 | protein_coding | -0.57 | 0.000116197 |
| AC138430.1 | SLC25A36 | protein_coding | -0.70 | 4.31E-07 |
| AC138430.1 | AK5 | protein_coding | -0.52 | 0.000535782 |
| AC138430.1 | GGA1 | protein_coding | -0.56 | 0.000128399 |
| AC138430.1 | PSORS1C1 | protein_coding | -0.73 | 7.19E-08 |
| AC138430.1 | PADI2 | protein_coding | -0.73 | 6.99E-08 |
| AC138430.1 | LAT | protein_coding | -0.59 | 4.27E-05 |
| AC138430.1 | OSBPL11 | protein_coding | 0.52 | 0.000436548 |
| AC138430.1 | RTL8C | protein_coding | -0.60 | 3.55E-05 |
| AC138430.1 | ATOH8 | protein_coding | 0.55 | 0.00020925 |
| AC138430.1 | CRIM1 | protein_coding | -0.57 | 8.89E-05 |
| AC138430.1 | HADHA | protein_coding | 0.66 | 2.39E-06 |
| AC138430.1 | GTPBP4 | protein_coding | -0.63 | 1.03E-05 |
| AC138430.1 | ZFP90 | protein_coding | -0.59 | 5.06E-05 |
| AC138430.1 | GCC2 | protein_coding | -0.54 | 0.000233476 |
| AC138430.1 | BCKDK | protein_coding | 0.62 | 1.33E-05 |
| AC138430.1 | TRIM36 | protein_coding | -0.54 | 0.000305703 |
| AC138430.1 | TNFRSF11A | protein_coding | -0.55 | 0.000186909 |
| AC138430.1 | SPATA17 | protein_coding | -0.66 | 3.13E-06 |
| AC138430.1 | GALNT3 | protein_coding | -0.56 | 0.000130805 |
| AC138430.1 | MT-ND4L | protein_coding | 0.68 | 1.28E-06 |
| AC138430.1 | ACLY | protein_coding | -0.58 | 7.42E-05 |
| AC138430.1 | VAV3 | protein_coding | -0.50 | 0.000844625 |
| AC138430.1 | NIPSNAP3A | protein_coding | 0.75 | 1.36E-08 |
| AC138430.1 | CYP2A13 | protein_coding | 0.78 | 1.81E-09 |
| AC138430.1 | TMSB15A | protein_coding | -0.65 | 4.90E-06 |
| AC138430.1 | RGL2 | protein_coding | -0.60 | 3.45E-05 |
| AC138430.1 | PLIN1 | protein_coding | 0.77 | 3.62E-09 |
| AC138430.1 | ECHS1 | protein_coding | 0.80 | 3.86E-10 |
| AC138430.1 | ACSL1 | protein_coding | 0.81 | 2.16E-10 |
| AC138430.1 | FURIN | protein_coding | 0.66 | 2.31E-06 |
| AC138430.1 | HTRA4 | protein_coding | -0.61 | 1.99E-05 |
| AC138430.1 | WWTR1 | protein_coding | -0.54 | 0.000285832 |
| AC138430.1 | TPBG | protein_coding | -0.58 | 6.12E-05 |
| AC138430.1 | RGSL1 | protein_coding | 0.66 | 2.42E-06 |
| AC138430.1 | C20orf194 | protein_coding | -0.70 | 2.90E-07 |
| AC138430.1 | ISOC1 | protein_coding | 0.84 | 7.16E-12 |
| AC138430.1 | SLC10A1 | protein_coding | 0.70 | 4.55E-07 |
| AC138430.1 | RASSF3 | protein_coding | -0.56 | 0.000133931 |
| AC138430.1 | EMILIN2 | protein_coding | -0.59 | 4.83E-05 |
| AC138430.1 | ALOXE3 | protein_coding | -0.55 | 0.000179713 |
| AC138430.1 | LRRIQ1 | protein_coding | -0.54 | 0.000237677 |
| AC138430.1 | NR1D2 | protein_coding | 0.55 | 0.000184982 |
| AC138430.1 | MT1G | protein_coding | 0.59 | 4.54E-05 |
| AC138430.1 | MFSD6L | protein_coding | -0.50 | 0.00079169 |
| AC138430.1 | DUSP18 | protein_coding | -0.77 | 4.53E-09 |
| AC138430.1 | UROC1 | protein_coding | 0.69 | 7.58E-07 |
| AC138430.1 | C8B | protein_coding | 0.62 | 1.83E-05 |
| AC138430.1 | WDR7 | protein_coding | 0.67 | 1.63E-06 |
| AC138430.1 | PLCD3 | protein_coding | -0.72 | 1.25E-07 |
| AC138430.1 | TRAF2 | protein_coding | -0.60 | 3.57E-05 |
| AC138430.1 | LPIN2 | protein_coding | 0.64 | 5.74E-06 |
| AC138430.1 | ZBTB12 | protein_coding | -0.71 | 1.79E-07 |
| AC138430.1 | CLSTN1 | protein_coding | -0.59 | 5.46E-05 |
| AC138430.1 | ZCCHC18 | protein_coding | -0.57 | 0.000119671 |
| AC138430.1 | ACOT4 | protein_coding | 0.73 | 8.24E-08 |
| AC138430.1 | PANK1 | protein_coding | 0.78 | 2.40E-09 |
| AC138430.1 | MRGBP | protein_coding | -0.60 | 3.02E-05 |
| AC138430.1 | PFKFB1 | protein_coding | 0.75 | 1.61E-08 |
| AC138430.1 | OAF | protein_coding | 0.77 | 3.15E-09 |
| AC138430.1 | GALK1 | protein_coding | 0.59 | 4.91E-05 |
| AC138430.1 | KMT5C | protein_coding | -0.58 | 6.62E-05 |
| AC138430.1 | APMAP | protein_coding | 0.76 | 1.21E-08 |
| AC138430.1 | AQP8 | protein_coding | 0.57 | 0.000108054 |
| AC138430.1 | PPM1E | protein_coding | 0.56 | 0.00013028 |
| AC138430.1 | EAF1 | protein_coding | 0.55 | 0.000177408 |
| AC138430.1 | MMP14 | protein_coding | -0.73 | 5.00E-08 |
| AC138430.1 | BOP1 | protein_coding | -0.59 | 5.34E-05 |
| AC138430.1 | ANKRD36C | protein_coding | -0.61 | 2.31E-05 |
| AC138430.1 | LAMB1 | protein_coding | -0.60 | 3.08E-05 |
| AC138430.1 | MTHFS | protein_coding | 0.77 | 5.35E-09 |
| AC138430.1 | FAM160B2 | protein_coding | -0.62 | 1.76E-05 |
| AC138430.1 | ESPN | protein_coding | 0.65 | 5.07E-06 |
| AC138430.1 | RXRG | protein_coding | 0.55 | 0.000203968 |
| AC138430.1 | IMPDH2 | protein_coding | -0.54 | 0.00024109 |
| AC138430.1 | PDE1C | protein_coding | -0.61 | 2.18E-05 |
| AC138430.1 | PLIN5 | protein_coding | 0.54 | 0.00030718 |
| AC138430.1 | JPT1 | protein_coding | -0.63 | 1.15E-05 |
| AC138430.1 | LOXL3 | protein_coding | -0.53 | 0.000410515 |
| AC138430.1 | C11orf54 | protein_coding | 0.59 | 4.28E-05 |
| AC138430.1 | LIMK1 | protein_coding | -0.81 | 1.29E-10 |
| AC138430.1 | SMURF1 | protein_coding | -0.51 | 0.000722653 |
| AC138430.1 | CEP104 | protein_coding | 0.66 | 3.38E-06 |
| AC138430.1 | KIF2A | protein_coding | -0.63 | 9.21E-06 |
| AC138430.1 | CD58 | protein_coding | -0.72 | 9.81E-08 |
| AC138430.1 | UBALD2 | protein_coding | -0.63 | 8.96E-06 |
| AC138430.1 | UGT1A1 | protein_coding | 0.70 | 4.20E-07 |
| AC138430.1 | SLX4 | protein_coding | -0.56 | 0.000150386 |
| AC138430.1 | SLC35F2 | protein_coding | -0.67 | 1.83E-06 |
| AC138430.1 | RAPH1 | protein_coding | 0.53 | 0.000403424 |
| AC138430.1 | ABCC2 | protein_coding | 0.61 | 2.65E-05 |
| AC138430.1 | CUEDC1 | protein_coding | -0.70 | 3.99E-07 |
| AC138430.1 | CCAR2 | protein_coding | -0.55 | 0.000218401 |
| AC138430.1 | FAM171A1 | protein_coding | -0.52 | 0.000477227 |
| AC138430.1 | AC023055.1 | protein_coding | -0.55 | 0.000179256 |
| AC138430.1 | CTSV | protein_coding | -0.64 | 7.40E-06 |
| AC138430.1 | TMEM234 | protein_coding | -0.53 | 0.000397224 |
| AC138430.1 | NR2F6 | protein_coding | 0.51 | 0.000602513 |
| AC138430.1 | TNFSF9 | protein_coding | -0.62 | 1.47E-05 |
| AC138430.1 | SFXN3 | protein_coding | -0.69 | 7.95E-07 |
| AC138430.1 | WWOX | protein_coding | 0.66 | 2.72E-06 |
| AC138430.1 | TMPRSS13 | protein_coding | -0.54 | 0.000316374 |
| AC138430.1 | AGBL2 | protein_coding | 0.50 | 0.000808259 |
| AC138430.1 | ALDOC | protein_coding | 0.73 | 5.79E-08 |
| AC138430.1 | DLX4 | protein_coding | -0.74 | 3.39E-08 |
| AC138430.1 | ABHD14A | protein_coding | 0.55 | 0.000214268 |
| AC138430.1 | PKLR | protein_coding | 0.65 | 4.69E-06 |
| AC138430.1 | KIAA0753 | protein_coding | -0.61 | 2.70E-05 |
| AC138430.1 | FBL | protein_coding | -0.59 | 4.43E-05 |
| AC138430.1 | GSTA1 | protein_coding | 0.72 | 1.50E-07 |
| AC138430.1 | B3GALT4 | protein_coding | -0.67 | 1.45E-06 |
| AC138430.1 | PDIK1L | protein_coding | 0.70 | 3.50E-07 |
| AC138430.1 | CBFA2T2 | protein_coding | -0.64 | 6.25E-06 |
| AC138430.1 | USP38 | protein_coding | 0.62 | 1.83E-05 |
| AC138430.1 | UBD | protein_coding | -0.69 | 5.80E-07 |
| AC138430.1 | NHLRC3 | protein_coding | 0.54 | 0.000233997 |
| AC138430.1 | CFL1 | protein_coding | -0.60 | 3.72E-05 |
| AC138430.1 | POFUT1 | protein_coding | 0.65 | 3.82E-06 |
| AC138430.1 | OLFML2A | protein_coding | -0.68 | 9.39E-07 |
| AC138430.1 | ZFAND2A | protein_coding | 0.52 | 0.000495282 |
| AC138430.1 | CNIH4 | protein_coding | -0.51 | 0.000697381 |
| AC138430.1 | JAG1 | protein_coding | -0.67 | 1.96E-06 |
| AC138430.1 | ZSWIM4 | protein_coding | -0.74 | 3.16E-08 |
| AC138430.1 | U2AF2 | protein_coding | -0.61 | 2.56E-05 |
| AC138430.1 | OS9 | protein_coding | 0.59 | 5.61E-05 |
| AC138430.1 | NYNRIN | protein_coding | 0.64 | 6.58E-06 |
| AC138430.1 | UBE2D3 | protein_coding | 0.58 | 7.63E-05 |
| AC138430.1 | TMEM220 | protein_coding | 0.78 | 2.16E-09 |
| AC138430.1 | PRMT2 | protein_coding | -0.66 | 2.62E-06 |
| AC138430.1 | F8 | protein_coding | 0.66 | 3.42E-06 |
| AC138430.1 | TREX2 | protein_coding | -0.66 | 2.63E-06 |
| AC138430.1 | SLC6A8 | protein_coding | -0.54 | 0.000275147 |
| AC138430.1 | COX6A2 | protein_coding | 0.61 | 2.68E-05 |
| AC138430.1 | ABCG2 | protein_coding | 0.78 | 1.91E-09 |
| AC138430.1 | CENPK | protein_coding | -0.61 | 2.76E-05 |
| AC138430.1 | ALAD | protein_coding | 0.83 | 1.56E-11 |
| AC138430.1 | KLHL42 | protein_coding | -0.51 | 0.000587877 |
| AC138430.1 | PTP4A3 | protein_coding | -0.76 | 1.18E-08 |
| AC138430.1 | POLD3 | protein_coding | -0.65 | 4.10E-06 |
| AC138430.1 | SLC22A24 | protein_coding | 0.71 | 1.95E-07 |
| AC138430.1 | KNTC1 | protein_coding | -0.69 | 6.70E-07 |
| AC138430.1 | ARHGAP8 | protein_coding | -0.57 | 0.000107693 |
| AC138430.1 | ZNF519 | protein_coding | -0.56 | 0.000142059 |
| AC138430.1 | FST | protein_coding | 0.53 | 0.000334105 |
| AC138430.1 | DDT | protein_coding | 0.71 | 1.71E-07 |
| AC138430.1 | TMEM254 | protein_coding | 0.65 | 4.85E-06 |
| AC138430.1 | SLC2A4RG | protein_coding | 0.64 | 7.45E-06 |
| AC138430.1 | GHR | protein_coding | 0.72 | 1.04E-07 |
| AC138430.1 | IGDCC4 | protein_coding | -0.51 | 0.000588158 |
| AC138430.1 | KCTD13 | protein_coding | -0.74 | 2.54E-08 |
| AC138430.1 | EDIL3 | protein_coding | -0.62 | 1.86E-05 |
| AC138430.1 | C11orf24 | protein_coding | 0.53 | 0.000416964 |
| AC138430.1 | TBC1D10B | protein_coding | -0.75 | 1.22E-08 |
| AC138430.1 | ID2 | protein_coding | 0.65 | 4.27E-06 |
| AC138430.1 | MCEE | protein_coding | 0.79 | 9.76E-10 |
| AC138430.1 | ZNF862 | protein_coding | -0.51 | 0.000649713 |
| AC138430.1 | ZNF296 | protein_coding | -0.76 | 8.81E-09 |
| AC138430.1 | ARL14 | protein_coding | -0.71 | 2.49E-07 |
| AC138430.1 | ALDH1A1 | protein_coding | 0.64 | 7.52E-06 |
| AC138430.1 | SELENOP | protein_coding | 0.82 | 8.32E-11 |
| AC138430.1 | FAM102B | protein_coding | -0.54 | 0.000249377 |
| AC138430.1 | STIMATE-MUSTN1 | protein_coding | 0.63 | 1.11E-05 |
| AC138430.1 | ING1 | protein_coding | 0.51 | 0.000767701 |
| AC138430.1 | RRM2 | protein_coding | -0.61 | 2.02E-05 |
| AC138430.1 | BBC3 | protein_coding | -0.66 | 3.08E-06 |
| AC138430.1 | VPS37C | protein_coding | -0.63 | 8.77E-06 |
| AC138430.1 | NMNAT2 | protein_coding | -0.56 | 0.000134772 |
| AC138430.1 | ZNF500 | protein_coding | -0.67 | 1.63E-06 |
| AC138430.1 | CLCF1 | protein_coding | -0.70 | 3.95E-07 |
| AC138430.1 | TCEAL9 | protein_coding | -0.64 | 6.87E-06 |
| AC138430.1 | RFNG | protein_coding | 0.61 | 2.27E-05 |
| AC138430.1 | LYPD6 | protein_coding | -0.59 | 4.41E-05 |
| AC138430.1 | BAHD1 | protein_coding | 0.52 | 0.000517391 |
| AC138430.1 | USH2A | protein_coding | 0.82 | 5.70E-11 |
| AC138430.1 | GALR3 | protein_coding | 0.69 | 7.63E-07 |
| AC138430.1 | RPL39L | protein_coding | -0.54 | 0.000240466 |
| AC138430.1 | PIK3IP1 | protein_coding | -0.52 | 0.000446362 |
| AC138430.1 | S100A11 | protein_coding | -0.84 | 9.07E-12 |
| AC138430.1 | TOR1B | protein_coding | 0.52 | 0.000526538 |
| AC138430.1 | HTRA3 | protein_coding | -0.57 | 9.43E-05 |
| AC138430.1 | PCGF5 | protein_coding | 0.62 | 1.63E-05 |
| AC138430.1 | GALNT10 | protein_coding | -0.61 | 2.15E-05 |
| AC138430.1 | CCDC93 | protein_coding | -0.62 | 1.49E-05 |
| AC138430.1 | DIAPH1 | protein_coding | 0.55 | 0.000169692 |
| AC138430.1 | MRPS28 | protein_coding | 0.76 | 1.10E-08 |
| AC138430.1 | ALOX12B | protein_coding | -0.51 | 0.000578753 |
| AC138430.1 | ADM2 | protein_coding | -0.55 | 0.000182064 |
| AC138430.1 | SEC24D | protein_coding | 0.57 | 0.00011609 |
| AC138430.1 | ERCC3 | protein_coding | -0.73 | 6.65E-08 |
| AC138430.1 | COL10A1 | protein_coding | -0.54 | 0.000259383 |
| AC138430.1 | PFKP | protein_coding | -0.81 | 2.10E-10 |
| AC138430.1 | COX5B | protein_coding | 0.51 | 0.00063924 |
| AC138430.1 | ACSM1 | protein_coding | 0.50 | 0.00084825 |
| AC138430.1 | DNMT3L | protein_coding | 0.79 | 6.27E-10 |
| AC138430.1 | RASAL1 | protein_coding | -0.53 | 0.000381044 |
| AC138430.1 | B4GALNT2 | protein_coding | -0.65 | 4.43E-06 |
| AC138430.1 | POMZP3 | protein_coding | -0.53 | 0.000372671 |
| AC138430.1 | CCDC65 | protein_coding | -0.73 | 4.94E-08 |
| AC138430.1 | SPDYA | protein_coding | -0.55 | 0.000172929 |
| AC138430.1 | PABPC1 | protein_coding | -0.66 | 3.15E-06 |
| AC138430.1 | PPM1A | protein_coding | 0.81 | 1.21E-10 |
| AC138430.1 | ACAT2 | protein_coding | 0.78 | 1.75E-09 |
| AC138430.1 | ZNF354A | protein_coding | -0.67 | 1.59E-06 |
| AC138430.1 | NDRG4 | protein_coding | -0.50 | 0.000828555 |
| AC138430.1 | CD200 | protein_coding | -0.55 | 0.000180801 |
| AC138430.1 | CEP112 | protein_coding | -0.51 | 0.000695678 |
| AC138430.1 | HEATR1 | protein_coding | -0.53 | 0.000401088 |
| AC138430.1 | SOX11 | protein_coding | -0.55 | 0.000170837 |
| AC138430.1 | EDEM1 | protein_coding | 0.56 | 0.000140004 |
| AC138430.1 | MFSD8 | protein_coding | 0.59 | 4.58E-05 |
| AC138430.1 | FMNL1 | protein_coding | -0.55 | 0.000217368 |
| AC138430.1 | C9 | protein_coding | 0.57 | 0.000113259 |
| AC138430.1 | TGDS | protein_coding | 0.74 | 2.42E-08 |
| AC138430.1 | SGK3 | protein_coding | 0.77 | 5.68E-09 |
| AC138430.1 | CPEB4 | protein_coding | 0.66 | 2.69E-06 |
| AC138430.1 | GALNT6 | protein_coding | -0.60 | 3.47E-05 |
| AC138430.1 | BICD1 | protein_coding | -0.74 | 2.97E-08 |
| AC138430.1 | NME7 | protein_coding | -0.56 | 0.000124039 |
| AC138430.1 | RIN1 | protein_coding | -0.64 | 6.79E-06 |
| AC138430.1 | ANKRD46 | protein_coding | 0.71 | 1.73E-07 |
| AC138430.1 | PRAP1 | protein_coding | 0.55 | 0.000213109 |
| AC138430.1 | APOC3 | protein_coding | 0.63 | 1.15E-05 |
| AC138430.1 | SLC7A13 | protein_coding | 0.60 | 3.29E-05 |
| AC138430.1 | SAMD1 | protein_coding | -0.51 | 0.000607233 |
| AC138430.1 | RAB35 | protein_coding | -0.63 | 1.23E-05 |
| AC138430.1 | BEND6 | protein_coding | -0.56 | 0.000154956 |
| AC138430.1 | BTNL9 | protein_coding | 0.55 | 0.000171005 |
| AC138430.1 | NAAA | protein_coding | 0.56 | 0.000124398 |
| AC138430.1 | CD248 | protein_coding | -0.53 | 0.000384577 |
| AC138430.1 | NAP1L5 | protein_coding | 0.58 | 6.10E-05 |
| AC138430.1 | MAF1 | protein_coding | -0.58 | 6.45E-05 |
| AC138430.1 | SUPT3H | protein_coding | -0.64 | 5.72E-06 |
| AC138430.1 | TRMT6 | protein_coding | -0.63 | 8.92E-06 |
| AC138430.1 | C19orf48 | protein_coding | -0.63 | 1.25E-05 |
| AC138430.1 | RBM22 | protein_coding | -0.56 | 0.000129253 |
| AC138430.1 | WDHD1 | protein_coding | -0.63 | 1.08E-05 |
| AC138430.1 | SMIM12 | protein_coding | 0.52 | 0.000495146 |
| AC138430.1 | DHX34 | protein_coding | -0.59 | 4.23E-05 |
| AC138430.1 | MT-ND5 | protein_coding | 0.75 | 1.38E-08 |
| AC138430.1 | EHMT2 | protein_coding | -0.72 | 1.13E-07 |
| AC138430.1 | ARMCX6 | protein_coding | -0.80 | 2.46E-10 |
| AC138430.1 | UNC93A | protein_coding | 0.71 | 2.71E-07 |
| AC138430.1 | CERS4 | protein_coding | 0.53 | 0.000357024 |
| AC138430.1 | KPNA3 | protein_coding | 0.50 | 0.000804358 |
| AC138430.1 | CELF1 | protein_coding | 0.57 | 0.000109377 |
| AC138430.1 | TRPC4 | protein_coding | -0.51 | 0.000659205 |
| AC138430.1 | KCNMB3 | protein_coding | -0.62 | 1.52E-05 |
| AC138430.1 | UNC13D | protein_coding | -0.73 | 7.95E-08 |
| AC138430.1 | C1orf53 | protein_coding | 0.54 | 0.000260842 |
| AC138430.1 | CDPF1 | protein_coding | -0.57 | 0.000101998 |
| AC138430.1 | ZNF574 | protein_coding | -0.50 | 0.000792827 |
| AC138430.1 | SLC26A1 | protein_coding | 0.71 | 2.70E-07 |
| AC138430.1 | ARRDC4 | protein_coding | 0.74 | 3.09E-08 |
| AC138430.1 | KHK | protein_coding | 0.79 | 1.19E-09 |
| AC138430.1 | ARMT1 | protein_coding | 0.62 | 1.33E-05 |
| AC138430.1 | PPP1R3G | protein_coding | 0.59 | 4.26E-05 |
| AC138430.1 | ADAM8 | protein_coding | -0.53 | 0.000412548 |
| AC138430.1 | HPS5 | protein_coding | 0.65 | 4.28E-06 |
| AC138430.1 | SYCE1L | protein_coding | -0.67 | 1.50E-06 |
| AC138430.1 | CYBA | protein_coding | -0.68 | 1.17E-06 |
| AC138430.1 | SEMA4A | protein_coding | -0.53 | 0.000339879 |
| AC138430.1 | TTC31 | protein_coding | 0.58 | 7.34E-05 |
| AC138430.1 | SLC39A10 | protein_coding | -0.73 | 6.22E-08 |
| AC138430.1 | SUSD6 | protein_coding | -0.58 | 7.45E-05 |
| AC138430.1 | HKDC1 | protein_coding | -0.64 | 5.60E-06 |
| AC138430.1 | ISYNA1 | protein_coding | -0.57 | 0.00010152 |
| AC138430.1 | KRTAP10-6 | protein_coding | 0.60 | 3.04E-05 |
| AC138430.1 | WNT3A | protein_coding | -0.52 | 0.000442578 |
| AC138430.1 | CEND1 | protein_coding | -0.58 | 7.88E-05 |
| AC138430.1 | ATG4A | protein_coding | 0.67 | 1.48E-06 |
| AC138430.1 | ASB1 | protein_coding | -0.58 | 6.61E-05 |
| AC138430.1 | CDC42SE2 | protein_coding | -0.71 | 2.13E-07 |
| AC138430.1 | CANT1 | protein_coding | -0.66 | 2.48E-06 |
| AC138430.1 | C1orf115 | protein_coding | 0.76 | 8.57E-09 |
| AC138430.1 | RAB11FIP5 | protein_coding | -0.54 | 0.000308916 |
| AC138430.1 | CNTNAP4 | protein_coding | 0.56 | 0.000133496 |
| AC138430.1 | PPIH | protein_coding | -0.55 | 0.000190862 |
| AC138430.1 | FAM193B | protein_coding | -0.56 | 0.000126667 |
| AC138430.1 | AGL | protein_coding | 0.77 | 3.86E-09 |
| AC138430.1 | GYS2 | protein_coding | 0.68 | 1.12E-06 |
| AC138430.1 | POPDC2 | protein_coding | -0.57 | 0.000101853 |
| AC138430.1 | ZADH2 | protein_coding | 0.67 | 1.49E-06 |
| AC138430.1 | TMEM190 | protein_coding | -0.56 | 0.000145845 |
| AC138430.1 | SYDE1 | protein_coding | -0.52 | 0.000455368 |
| AC138430.1 | IL11 | protein_coding | -0.60 | 3.07E-05 |
| AC138430.1 | NDUFC2 | protein_coding | 0.68 | 8.12E-07 |
| AC138430.1 | SEC16B | protein_coding | 0.53 | 0.000322114 |
| AC138430.1 | DOK3 | protein_coding | -0.55 | 0.000204121 |
| AC138430.1 | ETS2 | protein_coding | 0.67 | 1.42E-06 |
| AC138430.1 | DGAT1 | protein_coding | 0.67 | 1.90E-06 |
| AC138430.1 | BEND3 | protein_coding | -0.56 | 0.000161959 |
| AC138430.1 | SYT7 | protein_coding | 0.70 | 4.71E-07 |
| AC138430.1 | CAMSAP2 | protein_coding | -0.55 | 0.000180679 |
| AC138430.1 | FOXD4 | protein_coding | -0.52 | 0.000473498 |
| AC138430.1 | C1orf167 | protein_coding | 0.53 | 0.00041969 |
| AC138430.1 | PHEX | protein_coding | -0.72 | 1.23E-07 |
| AC138430.1 | AKR7A2 | protein_coding | 0.71 | 1.84E-07 |
| AC138430.1 | SETD4 | protein_coding | -0.62 | 1.73E-05 |
| AC138430.1 | RHBDF2 | protein_coding | -0.78 | 1.74E-09 |
| AC138430.1 | TCP10L2 | protein_coding | 0.69 | 7.77E-07 |
| AC138430.1 | PTMA | protein_coding | -0.69 | 6.74E-07 |
| AC138430.1 | IGSF3 | protein_coding | -0.70 | 3.74E-07 |
| AC138430.1 | ORAI2 | protein_coding | -0.87 | 3.23E-13 |
| AC138430.1 | CAP1 | protein_coding | -0.63 | 1.02E-05 |
| AC138430.1 | FAM163B | protein_coding | 0.55 | 0.00020707 |
| AC138430.1 | EZH2 | protein_coding | -0.70 | 2.99E-07 |
| AC138430.1 | CGNL1 | protein_coding | 0.66 | 2.27E-06 |
| AC138430.1 | FBP1 | protein_coding | 0.76 | 9.53E-09 |
| AC138430.1 | APOBEC3C | protein_coding | -0.64 | 5.88E-06 |
| AC138430.1 | IDNK | protein_coding | 0.76 | 9.34E-09 |
| AC138430.1 | PLEKHB2 | protein_coding | -0.62 | 1.42E-05 |
| AC138430.1 | LIN7C | protein_coding | 0.87 | 1.67E-13 |
| AC138430.1 | ALYREF | protein_coding | -0.62 | 1.82E-05 |
| AC138430.1 | SPEF1 | protein_coding | -0.56 | 0.000142679 |
| AC138430.1 | SRRM5 | protein_coding | -0.53 | 0.000396076 |
| AC138430.1 | LECT2 | protein_coding | 0.63 | 1.02E-05 |
| AC138430.1 | NDUFV2 | protein_coding | 0.58 | 8.34E-05 |
| AC138430.1 | ABCC5 | protein_coding | -0.65 | 4.44E-06 |
| AC138430.1 | POGK | protein_coding | -0.63 | 8.97E-06 |
| AC138430.1 | NPR2 | protein_coding | 0.55 | 0.000179339 |
| AC138430.1 | LRRC14 | protein_coding | -0.57 | 9.52E-05 |
| AC138430.1 | NPM3 | protein_coding | -0.59 | 5.49E-05 |
| AC138430.1 | LRP3 | protein_coding | 0.53 | 0.000420969 |
| AC138430.1 | GALNT2 | protein_coding | 0.59 | 5.00E-05 |
| AC138430.1 | LRRFIP2 | protein_coding | 0.63 | 8.95E-06 |
| AC138430.1 | OTX1 | protein_coding | -0.63 | 9.60E-06 |
| AC138430.1 | TSPAN7 | protein_coding | 0.51 | 0.000688505 |
| AC138430.1 | RIMS3 | protein_coding | -0.58 | 6.31E-05 |
| AC138430.1 | BLCAP | protein_coding | 0.61 | 2.52E-05 |
| AC138430.1 | TTC13 | protein_coding | -0.63 | 1.28E-05 |
| AC138430.1 | ARHGEF17 | protein_coding | -0.53 | 0.000369607 |
| AC138430.1 | FNDC10 | protein_coding | -0.58 | 8.10E-05 |
| AC138430.1 | PRCC | protein_coding | -0.59 | 5.05E-05 |
| AC138430.1 | RHOV | protein_coding | -0.73 | 5.98E-08 |
| AC138430.1 | KANK4 | protein_coding | 0.72 | 1.12E-07 |
| AC138430.1 | ASTL | protein_coding | -0.50 | 0.000797737 |
| AC138430.1 | APOA1 | protein_coding | 0.62 | 1.30E-05 |
| AC138430.1 | PARPBP | protein_coding | -0.64 | 8.30E-06 |
| AC138430.1 | RNFT1 | protein_coding | 0.51 | 0.0006424 |
| AC138430.1 | C1orf174 | protein_coding | 0.57 | 0.00011023 |
| AC138430.1 | SLC4A11 | protein_coding | -0.69 | 4.75E-07 |
| AC138430.1 | ADRB2 | protein_coding | 0.67 | 1.65E-06 |
| AC138430.1 | C11orf80 | protein_coding | -0.72 | 1.08E-07 |
| AC138430.1 | OSBPL6 | protein_coding | 0.53 | 0.000324106 |
| AC138430.1 | FBXL22 | protein_coding | -0.53 | 0.000342255 |
| AC138430.1 | UCHL1 | protein_coding | -0.58 | 8.49E-05 |
| AC138430.1 | FLNA | protein_coding | -0.77 | 3.99E-09 |
| AC138430.1 | VIP | protein_coding | 0.56 | 0.000140947 |
| AC138430.1 | DPP4 | protein_coding | 0.61 | 2.21E-05 |
| AC138430.1 | ZNF532 | protein_coding | -0.65 | 5.13E-06 |
| AC138430.1 | CARNMT1 | protein_coding | 0.61 | 2.03E-05 |
| AC138430.1 | RASEF | protein_coding | -0.60 | 3.44E-05 |
| AC138430.1 | CENPO | protein_coding | -0.69 | 7.90E-07 |
| AC138430.1 | LARS1 | protein_coding | -0.59 | 5.35E-05 |
| AC138430.1 | MTM1 | protein_coding | 0.76 | 7.12E-09 |
| AC138430.1 | SMOC1 | protein_coding | 0.65 | 3.74E-06 |
| AC138430.1 | BIRC3 | protein_coding | -0.51 | 0.000751724 |
| AC138430.1 | CYP1A2 | protein_coding | 0.64 | 5.52E-06 |
| AC138430.1 | NT5DC1 | protein_coding | 0.74 | 2.58E-08 |
| AC138430.1 | DELE1 | protein_coding | 0.51 | 0.000764447 |
| AC138430.1 | CHRD | protein_coding | 0.73 | 4.63E-08 |
| AC138430.1 | CSE1L | protein_coding | -0.53 | 0.000370305 |
| AC138430.1 | IGFBP3 | protein_coding | 0.60 | 3.15E-05 |
| AC138430.1 | SLC25A6 | protein_coding | -0.68 | 8.59E-07 |
| AC138430.1 | IRAK1 | protein_coding | -0.60 | 3.23E-05 |
| AC138430.1 | AURKAIP1 | protein_coding | 0.55 | 0.000173747 |
| AC138430.1 | GFM2 | protein_coding | 0.57 | 9.96E-05 |
| AC138430.1 | ATL2 | protein_coding | 0.60 | 3.45E-05 |
| AC138430.1 | MFSD5 | protein_coding | -0.71 | 2.16E-07 |
| AC138430.1 | CMBL | protein_coding | 0.71 | 1.79E-07 |
| AC138430.1 | TNS2 | protein_coding | 0.54 | 0.000244973 |
| AC138430.1 | APOC4-APOC2 | protein_coding | 0.70 | 3.38E-07 |
| AC138430.1 | TLK2 | protein_coding | -0.51 | 0.000587951 |
| AC138430.1 | LACTB2 | protein_coding | 0.55 | 0.000216387 |
| AC138430.1 | TAT | protein_coding | 0.70 | 3.39E-07 |
| AC138430.1 | ABHD6 | protein_coding | 0.74 | 2.99E-08 |
| AC138430.1 | KIAA1549L | protein_coding | -0.52 | 0.000496981 |
| AC138430.1 | GREM2 | protein_coding | 0.70 | 4.71E-07 |
| AC138430.1 | ANKRD52 | protein_coding | -0.70 | 2.79E-07 |
| AC138430.1 | APOE | protein_coding | 0.70 | 3.32E-07 |
| AC138430.1 | MAP9 | protein_coding | -0.56 | 0.000149274 |
| AC138430.1 | LRFN4 | protein_coding | -0.80 | 3.74E-10 |
| AC138430.1 | SPECC1 | protein_coding | -0.78 | 1.83E-09 |
| AC138430.1 | NUDT16L1 | protein_coding | 0.58 | 7.72E-05 |
| AC138430.1 | SLC22A2 | protein_coding | 0.59 | 5.01E-05 |
| AC138430.1 | C8orf58 | protein_coding | -0.59 | 5.62E-05 |
| AC138430.1 | CCN3 | protein_coding | -0.68 | 1.16E-06 |
| AC138430.1 | HYAL1 | protein_coding | 0.69 | 7.40E-07 |
| AC138430.1 | ZNF83 | protein_coding | -0.55 | 0.000225717 |
| AC138430.1 | IFRD2 | protein_coding | 0.61 | 2.53E-05 |
| AC138430.1 | RCN2 | protein_coding | -0.64 | 5.78E-06 |
| AC138430.1 | TRIM47 | protein_coding | -0.69 | 6.75E-07 |
| AC138430.1 | FUNDC1 | protein_coding | -0.55 | 0.000192371 |
| AC138430.1 | MLIP | protein_coding | 0.72 | 1.01E-07 |
| AC138430.1 | MARVELD1 | protein_coding | -0.54 | 0.00029296 |
| AC138430.1 | EHF | protein_coding | -0.54 | 0.00026821 |
| AC138430.1 | TTC9C | protein_coding | -0.58 | 7.67E-05 |
| AC138430.1 | PKN1 | protein_coding | -0.76 | 9.82E-09 |
| AC138430.1 | MORC2 | protein_coding | -0.67 | 1.76E-06 |
| AC138430.1 | FAM220A | protein_coding | -0.58 | 8.51E-05 |
| AC138430.1 | CACNB3 | protein_coding | -0.68 | 9.20E-07 |
| AC138430.1 | LARP1 | protein_coding | -0.52 | 0.000432189 |
| AC138430.1 | MFNG | protein_coding | -0.55 | 0.000218518 |
| AC138430.1 | ANG | protein_coding | 0.78 | 2.25E-09 |
| AC138430.1 | GPER1 | protein_coding | 0.59 | 5.94E-05 |
| AC138430.1 | SYTL5 | protein_coding | 0.52 | 0.000517544 |
| AC138430.1 | MARK2 | protein_coding | -0.59 | 5.33E-05 |
| AC138430.1 | MAOB | protein_coding | 0.56 | 0.000160811 |
| AC138430.1 | ANXA11 | protein_coding | -0.69 | 7.69E-07 |
| AC138430.1 | PRKAA1 | protein_coding | 0.51 | 0.000763302 |
| AC138430.1 | NXNL2 | protein_coding | -0.75 | 1.60E-08 |
| AC138430.1 | CCNJ | protein_coding | -0.52 | 0.000578475 |
| AC138430.1 | PFKFB3 | protein_coding | -0.53 | 0.000401229 |
| AC138430.1 | ADAMTS9 | protein_coding | -0.63 | 1.20E-05 |
| AC138430.1 | UBE2V2 | protein_coding | 0.57 | 0.00010055 |
| AC138430.1 | FCHSD1 | protein_coding | -0.75 | 2.16E-08 |
| AC138430.1 | SPINT1 | protein_coding | -0.77 | 3.00E-09 |
| AC138430.1 | TICRR | protein_coding | -0.66 | 2.41E-06 |
| AC138430.1 | COL22A1 | protein_coding | -0.72 | 1.13E-07 |
| AC138430.1 | SPRED1 | protein_coding | -0.57 | 0.000111089 |
| AC138430.1 | TTC26 | protein_coding | -0.57 | 0.000114289 |
| AC138430.1 | CTSF | protein_coding | 0.64 | 8.20E-06 |
| AC138430.1 | WNT9A | protein_coding | -0.75 | 1.99E-08 |
| AC138430.1 | PBK | protein_coding | -0.59 | 4.98E-05 |
| AC138430.1 | CA5A | protein_coding | 0.64 | 7.39E-06 |
| AC138430.1 | CTSK | protein_coding | -0.54 | 0.000276087 |
| AC138430.1 | G0S2 | protein_coding | 0.63 | 1.05E-05 |
| AC138430.1 | SPPL2A | protein_coding | 0.75 | 1.47E-08 |
| AC138430.1 | TMX4 | protein_coding | 0.50 | 0.000775266 |
| AC138430.1 | TTC34 | protein_coding | -0.58 | 6.80E-05 |
| AC138430.1 | DHRS2 | protein_coding | 0.64 | 7.63E-06 |
| AC138430.1 | RALY | protein_coding | -0.58 | 7.01E-05 |
| AC138430.1 | PAK6 | protein_coding | -0.53 | 0.0003916 |
| AC138430.1 | TMEM43 | protein_coding | -0.50 | 0.000858678 |
| AC138430.1 | ADH1B | protein_coding | 0.64 | 6.66E-06 |
| AC138430.1 | PCIF1 | protein_coding | -0.60 | 3.78E-05 |
| AC138430.1 | TMEM120B | protein_coding | -0.59 | 5.17E-05 |
| AC138430.1 | CD5L | protein_coding | 0.53 | 0.000320414 |
| AC138430.1 | ALDOB | protein_coding | 0.66 | 2.95E-06 |
| AC138430.1 | SCARB1 | protein_coding | 0.73 | 4.81E-08 |
| AC138430.1 | ACTL6B | protein_coding | 0.52 | 0.000495036 |
| AC138430.1 | GTSE1 | protein_coding | -0.69 | 4.99E-07 |
| AC138430.1 | FBRS | protein_coding | -0.76 | 7.71E-09 |
| AC138430.1 | PES1 | protein_coding | -0.68 | 8.06E-07 |
| AC138430.1 | IL18BP | protein_coding | -0.56 | 0.000155504 |
| AC138430.1 | GALP | protein_coding | 0.76 | 7.98E-09 |
| AC138430.1 | ARFGEF3 | protein_coding | -0.66 | 2.97E-06 |
| AC138430.1 | GPR155 | protein_coding | 0.59 | 5.38E-05 |
| AC138430.1 | USP21 | protein_coding | -0.58 | 6.64E-05 |
| AC138430.1 | ADCY10 | protein_coding | 0.62 | 1.66E-05 |
| AC138430.1 | ST6GALNAC4 | protein_coding | -0.65 | 5.31E-06 |
| AC138430.1 | CAPN10 | protein_coding | -0.60 | 4.12E-05 |
| AC138430.1 | ACTG1 | protein_coding | -0.64 | 6.19E-06 |
| AC138430.1 | FAH | protein_coding | 0.75 | 1.44E-08 |
| AC138430.1 | STK32C | protein_coding | -0.57 | 9.47E-05 |
| AC138430.1 | ADCY3 | protein_coding | -0.65 | 4.26E-06 |
| AC138430.1 | SREBF1 | protein_coding | 0.64 | 7.95E-06 |
| AC138430.1 | ZNF107 | protein_coding | -0.67 | 1.93E-06 |
| AC138430.1 | LAMC2 | protein_coding | -0.83 | 3.25E-11 |
| AC138430.1 | HPGD | protein_coding | 0.70 | 2.76E-07 |
| AC138430.1 | AFMID | protein_coding | 0.75 | 1.67E-08 |
| AC138430.1 | SEC23A | protein_coding | 0.68 | 8.99E-07 |
| AC138430.1 | CDKN2A | protein_coding | -0.62 | 1.37E-05 |
| AC138430.1 | LRIG1 | protein_coding | 0.56 | 0.000124847 |
| AC138430.1 | CEP164 | protein_coding | -0.60 | 3.08E-05 |
| AC138430.1 | FXYD1 | protein_coding | 0.70 | 3.32E-07 |
| AC138430.1 | TDGF1 | protein_coding | 0.64 | 8.18E-06 |
| AC138430.1 | CKLF-CMTM1 | protein_coding | -0.65 | 5.24E-06 |
| AC138430.1 | BTN2A2 | protein_coding | -0.65 | 3.80E-06 |
| AC138430.1 | PEG3 | protein_coding | 0.70 | 3.01E-07 |
| AC138430.1 | HCFC1 | protein_coding | -0.74 | 4.53E-08 |
| AC138430.1 | ASPA | protein_coding | 0.61 | 2.44E-05 |
| AC138430.1 | ALKBH5 | protein_coding | 0.63 | 9.36E-06 |
| AC138430.1 | NRARP | protein_coding | -0.59 | 4.84E-05 |
| AC138430.1 | INSR | protein_coding | 0.51 | 0.000616107 |
| AC138430.1 | KLHDC2 | protein_coding | 0.67 | 1.81E-06 |
| AC138430.1 | BAZ2A | protein_coding | -0.58 | 6.66E-05 |
| AC138430.1 | SLC24A3 | protein_coding | -0.64 | 6.81E-06 |
| AC138430.1 | GRN | protein_coding | -0.53 | 0.000362064 |
| AC138430.1 | HDHD3 | protein_coding | 0.65 | 4.53E-06 |
| AC138430.1 | PSORS1C2 | protein_coding | -0.58 | 8.03E-05 |
| AC138430.1 | WDR4 | protein_coding | -0.68 | 1.10E-06 |
| AC138430.1 | KRT15 | protein_coding | -0.51 | 0.000617118 |
| AC138430.1 | SCAP | protein_coding | 0.70 | 4.55E-07 |
| AC138430.1 | ZCCHC12 | protein_coding | -0.60 | 3.41E-05 |
| AC138430.1 | STAT3 | protein_coding | 0.54 | 0.000298483 |
| AC138430.1 | SIAH2 | protein_coding | 0.59 | 5.35E-05 |
| AC138430.1 | NKIRAS1 | protein_coding | 0.77 | 3.11E-09 |
| AC138430.1 | ATP1B3 | protein_coding | -0.84 | 6.16E-12 |
| AC138430.1 | THPO | protein_coding | 0.68 | 1.24E-06 |
| AC138430.1 | PDXP | protein_coding | 0.74 | 2.60E-08 |
| AC138430.1 | CENPH | protein_coding | -0.67 | 1.94E-06 |
| AC138430.1 | APOC4 | protein_coding | 0.77 | 4.25E-09 |
| AC138430.1 | MTMR2 | protein_coding | -0.77 | 4.05E-09 |
| AC138430.1 | MASTL | protein_coding | -0.59 | 5.99E-05 |
| AC138430.1 | ESYT3 | protein_coding | -0.51 | 0.000674723 |
| AC138430.1 | MST1 | protein_coding | 0.73 | 6.86E-08 |
| AC138430.1 | BUB1 | protein_coding | -0.74 | 4.13E-08 |
| AC138430.1 | SPHK1 | protein_coding | -0.55 | 0.000203732 |
| AC138430.1 | IST1 | protein_coding | -0.51 | 0.000750995 |
| AC138430.1 | GRPEL1 | protein_coding | 0.70 | 3.40E-07 |
| AC138430.1 | PDE7A | protein_coding | -0.66 | 2.20E-06 |
| AC138430.1 | PIK3C2G | protein_coding | 0.63 | 1.26E-05 |
| AC138430.1 | ETV4 | protein_coding | -0.55 | 0.000215831 |
| AC138430.1 | CHD3 | protein_coding | -0.69 | 6.15E-07 |
| AC138430.1 | MT-ATP8 | protein_coding | 0.67 | 1.68E-06 |
| AC138430.1 | ANGPTL8 | protein_coding | 0.63 | 1.13E-05 |
| AC138430.1 | USP19 | protein_coding | 0.62 | 1.57E-05 |
| AC138430.1 | NHSL1 | protein_coding | 0.50 | 0.000858037 |
| AC138430.1 | EHD4 | protein_coding | -0.51 | 0.000581027 |
| AC138430.1 | SAP18 | protein_coding | 0.57 | 9.93E-05 |
| AC138430.1 | SERTAD3 | protein_coding | -0.52 | 0.000543991 |
| AC138430.1 | RASL11A | protein_coding | 0.50 | 0.000852171 |
| AC138430.1 | SLC17A8 | protein_coding | 0.67 | 1.35E-06 |
| AC138430.1 | SMARCA2 | protein_coding | 0.56 | 0.000147418 |
| AC138430.1 | IFT22 | protein_coding | -0.63 | 9.46E-06 |
| AC138430.1 | JRK | protein_coding | -0.65 | 5.37E-06 |
| AC138430.1 | RHOF | protein_coding | -0.64 | 6.58E-06 |
| AC138430.1 | GSTO1 | protein_coding | 0.60 | 3.17E-05 |
| AC138430.1 | ODF2L | protein_coding | -0.57 | 0.000111138 |
| AC138430.1 | SEC14L2 | protein_coding | 0.78 | 1.71E-09 |
| AC138430.1 | ETFB | protein_coding | 0.59 | 5.04E-05 |
| AC138430.1 | PHF7 | protein_coding | 0.69 | 7.13E-07 |
| AC138430.1 | SENP2 | protein_coding | 0.57 | 0.000120157 |
| AC138430.1 | AVPI1 | protein_coding | 0.77 | 4.68E-09 |
| AC138430.1 | GSDMC | protein_coding | -0.59 | 4.34E-05 |
| AC138430.1 | VPS26B | protein_coding | 0.58 | 8.00E-05 |
| AC138430.1 | RHOC | protein_coding | -0.52 | 0.000558347 |
| AC138430.1 | APOA5 | protein_coding | 0.65 | 4.59E-06 |
| AC138430.1 | KCTD6 | protein_coding | 0.60 | 3.19E-05 |
| AC138430.1 | DMTF1 | protein_coding | -0.50 | 0.000818776 |
| AC138430.1 | SPP2 | protein_coding | 0.66 | 3.38E-06 |
| AC138430.1 | PRXL2B | protein_coding | -0.64 | 6.70E-06 |
| AC138430.1 | RPS19 | protein_coding | -0.53 | 0.00037047 |
| AC138430.1 | COL26A1 | protein_coding | 0.65 | 5.23E-06 |
| AC138430.1 | SYNGR2 | protein_coding | -0.79 | 6.55E-10 |
| AC138430.1 | FERMT2 | protein_coding | 0.66 | 2.54E-06 |
| AC138430.1 | CRBN | protein_coding | 0.68 | 1.09E-06 |
| AC138430.1 | CKLF | protein_coding | -0.53 | 0.000396034 |
| AC138430.1 | USP15 | protein_coding | 0.50 | 0.000845691 |
| AC138430.1 | INTS9 | protein_coding | -0.53 | 0.000396396 |
| AC138430.1 | ECI2 | protein_coding | 0.72 | 1.27E-07 |
| AC138430.1 | ALDOA | protein_coding | -0.66 | 3.22E-06 |
| AC138430.1 | CDH11 | protein_coding | -0.54 | 0.00027102 |
| AC138430.1 | TRAIP | protein_coding | -0.62 | 1.29E-05 |
| AC138430.1 | LRCOL1 | protein_coding | 0.64 | 6.16E-06 |
| AC138430.1 | DBNDD2 | protein_coding | -0.78 | 1.31E-09 |
| AC138430.1 | EFCAB7 | protein_coding | -0.68 | 8.17E-07 |
| AC138430.1 | HSPB9 | protein_coding | 0.64 | 6.20E-06 |
| AC138430.1 | ELOA | protein_coding | 0.74 | 3.98E-08 |
| AC138430.1 | MIER2 | protein_coding | -0.77 | 4.80E-09 |
| AC138430.1 | FBXO31 | protein_coding | 0.66 | 2.29E-06 |
| AC138430.1 | TTC39A | protein_coding | -0.70 | 3.91E-07 |
| AC138430.1 | ARPC2 | protein_coding | -0.63 | 1.11E-05 |
| AC138430.1 | RNF223 | protein_coding | -0.59 | 4.19E-05 |
| AC138430.1 | XKR8 | protein_coding | 0.68 | 9.26E-07 |
| AC138430.1 | HSPB1 | protein_coding | -0.58 | 6.54E-05 |
| AC138430.1 | TRDMT1 | protein_coding | -0.57 | 0.000105755 |
| AC138430.1 | LEPR | protein_coding | 0.74 | 3.28E-08 |
| AC138430.1 | ZDHHC19 | protein_coding | 0.56 | 0.000132096 |
| AC138430.1 | TF | protein_coding | 0.69 | 5.23E-07 |
| AC138430.1 | MTHFD2 | protein_coding | -0.67 | 1.60E-06 |
| AC138430.1 | PTPN2 | protein_coding | -0.50 | 0.000800369 |
| AC138430.1 | PIK3R4 | protein_coding | 0.56 | 0.000157297 |
| AC138430.1 | CEP95 | protein_coding | -0.57 | 9.23E-05 |
| AC138430.1 | TFAP2C | protein_coding | -0.51 | 0.000660491 |
| AC138430.1 | MRPS23 | protein_coding | -0.57 | 8.76E-05 |
| AC138430.1 | NEO1 | protein_coding | -0.55 | 0.000180602 |
| AC138430.1 | PHF20 | protein_coding | -0.66 | 2.85E-06 |
| AC138430.1 | RORA | protein_coding | 0.62 | 1.87E-05 |
| AC138430.1 | HRG | protein_coding | 0.58 | 7.40E-05 |
| AC138430.1 | WNT7B | protein_coding | -0.60 | 2.90E-05 |
| AC138430.1 | PSMG3 | protein_coding | -0.57 | 0.000111249 |
| AC138430.1 | LZTS2 | protein_coding | -0.65 | 3.67E-06 |
| AC138430.1 | SPTAN1 | protein_coding | -0.64 | 5.76E-06 |
| AC138430.1 | PIFO | protein_coding | -0.72 | 9.79E-08 |
| AC138430.1 | PPP1R14B | protein_coding | -0.54 | 0.000234126 |
| AC138430.1 | TARBP1 | protein_coding | -0.55 | 0.0002212 |
| AC138430.1 | KPNB1 | protein_coding | -0.54 | 0.000240937 |
| AC138430.1 | GNAO1 | protein_coding | 0.74 | 4.40E-08 |
| AC138430.1 | PAQR9 | protein_coding | 0.53 | 0.000398971 |
| AC138430.1 | NQO2 | protein_coding | 0.67 | 2.01E-06 |
| AC138430.1 | PLD2 | protein_coding | -0.55 | 0.000204099 |
| AC138430.1 | VPS33B | protein_coding | -0.56 | 0.000165141 |
| AC138430.1 | ZNF267 | protein_coding | -0.64 | 5.44E-06 |
| AC138430.1 | SSPN | protein_coding | -0.55 | 0.000219763 |
| AC138430.1 | MED1 | protein_coding | -0.55 | 0.000217312 |
| AC138430.1 | CCNA2 | protein_coding | -0.64 | 6.31E-06 |
| AC138430.1 | MTRF1 | protein_coding | 0.60 | 3.43E-05 |
| AC138430.1 | KCNC4 | protein_coding | -0.68 | 1.01E-06 |
| AC138430.1 | RPL17 | protein_coding | -0.50 | 0.000816748 |
| AC138430.1 | KLHL30 | protein_coding | -0.51 | 0.000702459 |
| AC138430.1 | CD300LG | protein_coding | 0.54 | 0.00029517 |
| AC138430.1 | ZNF367 | protein_coding | 0.56 | 0.000127065 |
| AC138430.1 | C8orf82 | protein_coding | 0.77 | 4.58E-09 |
| AC138430.1 | GPAM | protein_coding | 0.77 | 3.81E-09 |
| AC138430.1 | NLGN3 | protein_coding | -0.54 | 0.000234928 |
| AC138430.1 | PLEKHB1 | protein_coding | -0.70 | 3.99E-07 |
| AC138430.1 | SLC66A1L | protein_coding | -0.68 | 1.17E-06 |
| AC138430.1 | CYB5A | protein_coding | 0.73 | 5.28E-08 |
| AC138430.1 | IMPDH1 | protein_coding | -0.69 | 7.92E-07 |
| AC138430.1 | TRPM7 | protein_coding | 0.65 | 4.15E-06 |
| AC138430.1 | IQGAP1 | protein_coding | -0.70 | 2.90E-07 |
| AC138430.1 | MAPRE1 | protein_coding | -0.77 | 4.37E-09 |
| AC138430.1 | EIF4ENIF1 | protein_coding | -0.60 | 3.12E-05 |
| AC138430.1 | CALCOCO2 | protein_coding | 0.63 | 1.04E-05 |
| AC138430.1 | MCF2L2 | protein_coding | -0.58 | 7.61E-05 |
| AC138430.1 | GCSAM | protein_coding | -0.56 | 0.000134055 |
| AC138430.1 | TMEM216 | protein_coding | -0.56 | 0.00012104 |
| AC138430.1 | ZNF385A | protein_coding | -0.62 | 1.64E-05 |
| AC138430.1 | STRADB | protein_coding | 0.51 | 0.000694572 |
| AC138430.1 | MAPK8IP3 | protein_coding | -0.64 | 6.48E-06 |
| AC138430.1 | DHODH | protein_coding | 0.76 | 8.11E-09 |
| AC138430.1 | AP5M1 | protein_coding | 0.53 | 0.000327949 |
| AC138430.1 | SLC25A20 | protein_coding | 0.84 | 4.48E-12 |
| AC138430.1 | TCF3 | protein_coding | -0.77 | 4.48E-09 |
| AC138430.1 | MRPS36 | protein_coding | 0.51 | 0.000631522 |
| AC138430.1 | BICDL1 | protein_coding | -0.51 | 0.000629151 |
| AC138430.1 | NXT1 | protein_coding | -0.53 | 0.000349688 |
| AC138430.1 | CFAP57 | protein_coding | 0.56 | 0.000138005 |
| AC138430.1 | CDA | protein_coding | 0.74 | 4.50E-08 |
| AC138430.1 | LONP2 | protein_coding | 0.83 | 2.63E-11 |
| AC138430.1 | SLFN13 | protein_coding | -0.75 | 1.44E-08 |
| AC138430.1 | DCXR | protein_coding | 0.83 | 1.68E-11 |
| AC138430.1 | PLA2G4F | protein_coding | -0.55 | 0.000231791 |
| AC138430.1 | COX18 | protein_coding | 0.72 | 1.17E-07 |
| AC138430.1 | TMEM159 | protein_coding | -0.60 | 3.34E-05 |
| AC138430.1 | SFXN1 | protein_coding | 0.80 | 3.30E-10 |
| AC138430.1 | DBN1 | protein_coding | -0.80 | 4.39E-10 |
| AC138430.1 | PPP1R18 | protein_coding | -0.53 | 0.000374194 |
| AC138430.1 | ASB13 | protein_coding | 0.59 | 4.66E-05 |
| AC138430.1 | GLCE | protein_coding | 0.53 | 0.00037618 |
| AC138430.1 | CBR3 | protein_coding | -0.54 | 0.000281522 |
| AC138430.1 | PPDPF | protein_coding | -0.58 | 6.02E-05 |
| AC138430.1 | NELFE | protein_coding | -0.61 | 2.19E-05 |
| AC138430.1 | INPPL1 | protein_coding | -0.63 | 1.02E-05 |
| AC138430.1 | MPLKIP | protein_coding | 0.69 | 5.56E-07 |
| AC138430.1 | PHLDA2 | protein_coding | -0.53 | 0.000374226 |
| AC138430.1 | GOLGA3 | protein_coding | -0.65 | 3.50E-06 |
| AC138430.1 | ZSCAN25 | protein_coding | -0.51 | 0.000689951 |
| AC138430.1 | PLEKHO1 | protein_coding | -0.50 | 0.000820263 |
| AC138430.1 | ANKS3 | protein_coding | -0.76 | 8.48E-09 |
| AC138430.1 | PRC1 | protein_coding | -0.67 | 1.59E-06 |
| AC138430.1 | STIMATE | protein_coding | 0.79 | 9.86E-10 |
| AC138430.1 | GCH1 | protein_coding | 0.75 | 1.88E-08 |
| AC138430.1 | PLK4 | protein_coding | -0.71 | 2.68E-07 |
| AC138430.1 | SYNJ2BP | protein_coding | 0.65 | 3.57E-06 |
| AC138430.1 | DNAJC11 | protein_coding | 0.58 | 6.01E-05 |
| AC138430.1 | B3GNT3 | protein_coding | -0.58 | 7.35E-05 |
| AC138430.1 | VEZF1 | protein_coding | -0.68 | 1.31E-06 |
| AC138430.1 | CNOT3 | protein_coding | -0.57 | 9.36E-05 |
| AC138430.1 | DPF3 | protein_coding | 0.56 | 0.000167365 |
| AC138430.1 | SLC38A9 | protein_coding | 0.54 | 0.000300101 |
| AC138430.1 | MAN1A1 | protein_coding | 0.75 | 1.45E-08 |
| AC138430.1 | RNF148 | protein_coding | -0.52 | 0.000550205 |
| AC138430.1 | PRDX6 | protein_coding | 0.73 | 6.91E-08 |
| AC138430.1 | SOAT2 | protein_coding | 0.58 | 7.59E-05 |
| AC138430.1 | PKP2 | protein_coding | 0.56 | 0.000137273 |
| AC138430.1 | ADGRV1 | protein_coding | 0.69 | 7.14E-07 |
| AC138430.1 | ANAPC4 | protein_coding | -0.52 | 0.000503017 |
| AC138430.1 | NEURL3 | protein_coding | -0.61 | 2.07E-05 |
| AC138430.1 | GID4 | protein_coding | 0.66 | 3.09E-06 |
| AC138430.1 | CCNE1 | protein_coding | -0.69 | 6.98E-07 |
| AC138430.1 | MDK | protein_coding | -0.52 | 0.000507272 |
| AC138430.1 | LYRM7 | protein_coding | 0.61 | 2.60E-05 |
| AC138430.1 | TTLL1 | protein_coding | -0.75 | 1.99E-08 |
| AC138430.1 | USP2 | protein_coding | 0.60 | 3.61E-05 |
| AC138430.1 | CLIC1 | protein_coding | -0.76 | 6.48E-09 |
| AC138430.1 | PXMP2 | protein_coding | 0.81 | 1.06E-10 |
| AC138430.1 | LIN7A | protein_coding | 0.56 | 0.000126389 |
| AC138430.1 | ZNF580 | protein_coding | -0.56 | 0.000158428 |
| AC138430.1 | CPNE9 | protein_coding | -0.56 | 0.000152957 |
| AC138430.1 | SLC6A12 | protein_coding | 0.74 | 3.21E-08 |
| AC138430.1 | SULF1 | protein_coding | -0.69 | 6.66E-07 |
| AC138430.1 | PHACTR4 | protein_coding | 0.54 | 0.0002974 |
| AC138430.1 | VOPP1 | protein_coding | -0.61 | 2.22E-05 |
| AC138430.1 | AKR1C4 | protein_coding | 0.68 | 9.57E-07 |
| AC138430.1 | C11orf97 | protein_coding | 0.55 | 0.000224256 |
| AC138430.1 | CTXN1 | protein_coding | -0.76 | 9.60E-09 |
| AC138430.1 | OXNAD1 | protein_coding | 0.62 | 1.75E-05 |
| AC138430.1 | CDHR5 | protein_coding | 0.67 | 1.77E-06 |
| AC138430.1 | SEPTIN4 | protein_coding | 0.68 | 9.42E-07 |
| AC138430.1 | DDAH1 | protein_coding | 0.64 | 8.19E-06 |
| AC138430.1 | DYNC1I2 | protein_coding | -0.62 | 1.42E-05 |
| AC138430.1 | ACSM2B | protein_coding | 0.69 | 6.33E-07 |
| AC138430.1 | TRAF5 | protein_coding | -0.68 | 1.03E-06 |
| AC138430.1 | UGT3A1 | protein_coding | 0.60 | 3.06E-05 |
| AC138430.1 | TBC1D22B | protein_coding | -0.60 | 3.34E-05 |
| AC138430.1 | PGAP3 | protein_coding | 0.73 | 7.55E-08 |
| AC138430.1 | RRP12 | protein_coding | -0.60 | 3.31E-05 |
| AC138430.1 | PKM | protein_coding | -0.83 | 2.10E-11 |
| AC138430.1 | PI4K2B | protein_coding | 0.77 | 4.84E-09 |
| AC138430.1 | KCNG1 | protein_coding | -0.69 | 7.39E-07 |
| AC138430.1 | HMGCS2 | protein_coding | 0.68 | 8.33E-07 |
| AC138430.1 | SUPT5H | protein_coding | -0.57 | 8.97E-05 |
| AC138430.1 | ATP6V1FNB | protein_coding | -0.69 | 6.16E-07 |
| AC138430.1 | DDX11 | protein_coding | -0.60 | 3.62E-05 |
| AC138430.1 | MCM3 | protein_coding | -0.70 | 3.00E-07 |
| AC138430.1 | PIP4P2 | protein_coding | -0.54 | 0.000233727 |
| AC138430.1 | PIGC | protein_coding | -0.62 | 1.32E-05 |
| AC138430.1 | SLC25A16 | protein_coding | 0.50 | 0.000800547 |
| AC138430.1 | RBM3 | protein_coding | -0.78 | 1.57E-09 |
| AC138430.1 | MLLT6 | protein_coding | -0.66 | 3.04E-06 |
| AC138430.1 | DMPK | protein_coding | -0.68 | 9.30E-07 |
| AC138430.1 | RTN2 | protein_coding | -0.53 | 0.000422667 |
| AC138430.1 | PA2G4 | protein_coding | -0.50 | 0.000808766 |
| AC138430.1 | PHF12 | protein_coding | -0.52 | 0.000531766 |
| AC138430.1 | CHAF1A | protein_coding | -0.61 | 2.43E-05 |
| AC138430.1 | MORN3 | protein_coding | -0.72 | 9.90E-08 |
| AC138430.1 | MYH1 | protein_coding | -0.50 | 0.000792367 |
| AC138430.1 | CYP1A1 | protein_coding | 0.68 | 8.79E-07 |
| AC138430.1 | CEBPA | protein_coding | 0.65 | 3.63E-06 |
| AC138430.1 | TC2N | protein_coding | -0.51 | 0.000656014 |
| AC138430.1 | ZSWIM9 | protein_coding | -0.54 | 0.000314876 |
| AC138430.1 | CDC45 | protein_coding | -0.69 | 5.61E-07 |
| AC138430.1 | F10 | protein_coding | 0.73 | 6.56E-08 |
| AC138430.1 | GDPD3 | protein_coding | -0.51 | 0.000678732 |
| AC138430.1 | MARCHF3 | protein_coding | -0.53 | 0.000385608 |
| AC138430.1 | DNAH6 | protein_coding | 0.61 | 2.30E-05 |
| AC138430.1 | AURKA | protein_coding | -0.55 | 0.000185901 |
| AC138430.1 | TSPO | protein_coding | -0.60 | 3.31E-05 |
| AC138430.1 | DHRS12 | protein_coding | 0.74 | 4.21E-08 |
| AC138430.1 | AC010542.3 | protein_coding | 0.59 | 5.83E-05 |
| AC138430.1 | PBX4 | protein_coding | -0.67 | 1.50E-06 |
| AC138430.1 | YEATS2 | protein_coding | -0.82 | 3.51E-11 |
| AC138430.1 | ALDH7A1 | protein_coding | 0.82 | 6.02E-11 |
| AC138430.1 | SLC35F3 | protein_coding | -0.67 | 2.11E-06 |
| AC138430.1 | TMEM256-PLSCR3 | protein_coding | 0.57 | 0.000112727 |
| AC138430.1 | JPT2 | protein_coding | -0.56 | 0.000158604 |
| AC138430.1 | DNMT3A | protein_coding | -0.76 | 6.15E-09 |
| AC138430.1 | SAR1B | protein_coding | 0.80 | 4.07E-10 |
| AC138430.1 | WDR73 | protein_coding | -0.71 | 2.57E-07 |
| AC138430.1 | MT1H | protein_coding | 0.62 | 1.71E-05 |
| AC138430.1 | MLLT3 | protein_coding | -0.72 | 8.90E-08 |
| AC138430.1 | C5orf34 | protein_coding | -0.65 | 3.63E-06 |
| AC138430.1 | SERPINF2 | protein_coding | 0.61 | 2.07E-05 |
| AC138430.1 | HAGHL | protein_coding | -0.71 | 2.48E-07 |
| AC138430.1 | EBPL | protein_coding | 0.71 | 2.69E-07 |
| AC138430.1 | IDI1 | protein_coding | 0.73 | 6.52E-08 |
| AC138430.1 | ABCC10 | protein_coding | -0.70 | 2.97E-07 |
| AC138430.1 | KLF15 | protein_coding | 0.78 | 2.19E-09 |
| AC138430.1 | N4BP3 | protein_coding | -0.75 | 2.13E-08 |
| AC138430.1 | RHCE | protein_coding | 0.72 | 1.28E-07 |
| AC138430.1 | ZNF365 | protein_coding | -0.64 | 6.98E-06 |
| AC138430.1 | VEPH1 | protein_coding | -0.57 | 0.000108123 |
| AC138430.1 | GYPA | protein_coding | 0.58 | 8.08E-05 |
| AC138430.1 | TP53TG5 | protein_coding | -0.66 | 2.97E-06 |
| AC138430.1 | VMA21 | protein_coding | -0.50 | 0.000788857 |
| AC138430.1 | MICU2 | protein_coding | 0.52 | 0.000561858 |
| AC138430.1 | GPAT3 | protein_coding | 0.62 | 1.64E-05 |
| AC138430.1 | FOXM1 | protein_coding | -0.73 | 7.15E-08 |
| AC138430.1 | SLCO1B1 | protein_coding | 0.58 | 8.30E-05 |
| AC138430.1 | FAM83B | protein_coding | -0.63 | 1.15E-05 |
| AC138430.1 | KIF3A | protein_coding | -0.54 | 0.000277569 |
| AC138430.1 | SSBP2 | protein_coding | -0.57 | 9.86E-05 |
| AC138430.1 | PLPP2 | protein_coding | -0.68 | 8.99E-07 |
| AC138430.1 | YAE1 | protein_coding | -0.50 | 0.000814211 |
| AC138430.1 | ADH1A | protein_coding | 0.66 | 2.96E-06 |
| AC138430.1 | PROC | protein_coding | 0.61 | 2.71E-05 |
| AC138430.1 | PKD1L3 | protein_coding | 0.59 | 5.20E-05 |
| AC138430.1 | CCDC137 | protein_coding | -0.69 | 5.05E-07 |
| AC138430.1 | TRIP4 | protein_coding | -0.52 | 0.000489393 |
| AC138430.1 | GASK1A | protein_coding | 0.60 | 3.56E-05 |
| AC138430.1 | ACTC1 | protein_coding | -0.63 | 8.49E-06 |
| AC138430.1 | KLHL29 | protein_coding | -0.52 | 0.000480307 |
| AC138430.1 | FZD10 | protein_coding | -0.76 | 7.43E-09 |
| AC138430.1 | UTP20 | protein_coding | -0.57 | 9.37E-05 |
| AC138430.1 | CPEB3 | protein_coding | 0.79 | 1.00E-09 |
| AC138430.1 | ZBED5 | protein_coding | -0.73 | 7.50E-08 |
| AC138430.1 | PLCB4 | protein_coding | -0.53 | 0.000416873 |
| AC138430.1 | SLC22A9 | protein_coding | 0.73 | 6.82E-08 |
| AC138430.1 | PLIN4 | protein_coding | 0.64 | 6.25E-06 |
| AC138430.1 | ADO | protein_coding | -0.67 | 1.55E-06 |
| AC138430.1 | AMH | protein_coding | -0.56 | 0.000129543 |
| AC138430.1 | PPID | protein_coding | 0.74 | 3.94E-08 |
| AC138430.1 | A1BG | protein_coding | 0.69 | 4.77E-07 |
| AC138430.1 | GNAZ | protein_coding | -0.61 | 2.53E-05 |
| AC138430.1 | TLX1 | protein_coding | 0.62 | 1.37E-05 |
| AC138430.1 | CES1 | protein_coding | 0.70 | 3.43E-07 |
| AC138430.1 | VARS1 | protein_coding | -0.54 | 0.000273556 |
| AC138430.1 | UPP2 | protein_coding | 0.64 | 5.65E-06 |
| AC138430.1 | CDK1 | protein_coding | -0.65 | 3.56E-06 |
| AC138430.1 | MGP | protein_coding | -0.52 | 0.000566292 |
| AC138430.1 | TENM1 | protein_coding | 0.70 | 3.89E-07 |
| AC138430.1 | LONP1 | protein_coding | 0.55 | 0.000191184 |
| AC138430.1 | INHBC | protein_coding | 0.72 | 9.59E-08 |
| AC138430.1 | ALDH1L1 | protein_coding | 0.64 | 7.11E-06 |
| AC138430.1 | MEP1B | protein_coding | 0.85 | 1.19E-12 |
| AC138430.1 | PLIN2 | protein_coding | 0.72 | 1.23E-07 |
| AC138430.1 | GRIA3 | protein_coding | 0.64 | 8.34E-06 |
| AC138430.1 | ADGRB2 | protein_coding | -0.53 | 0.00032347 |
| AC138430.1 | PDGFA | protein_coding | -0.66 | 3.15E-06 |
| AC138430.1 | CD99L2 | protein_coding | 0.62 | 1.35E-05 |
| AC138430.1 | ZNF563 | protein_coding | 0.60 | 3.46E-05 |
| AC138430.1 | TMEM97 | protein_coding | 0.57 | 0.000116262 |
| AC138430.1 | COLEC10 | protein_coding | 0.69 | 6.18E-07 |
| AC138430.1 | LZTR1 | protein_coding | -0.59 | 5.65E-05 |
| AC138430.1 | PDXDC1 | protein_coding | 0.61 | 2.03E-05 |
| AC138430.1 | MDH1B | protein_coding | -0.54 | 0.000308371 |
| AC138430.1 | CALML3 | protein_coding | 0.82 | 5.68E-11 |
| AC138430.1 | UBE2G1 | protein_coding | 0.61 | 2.22E-05 |
| AC138430.1 | UFC1 | protein_coding | -0.53 | 0.00036769 |
| AC138430.1 | SERPINA6 | protein_coding | 0.56 | 0.000168589 |
| AC138430.1 | SERPINA5 | protein_coding | 0.57 | 0.000102693 |
| AC138430.1 | TBCEL | protein_coding | 0.55 | 0.000172464 |
| AC138430.1 | FAXDC2 | protein_coding | 0.66 | 3.24E-06 |
| AC138430.1 | ACSBG2 | protein_coding | -0.62 | 1.80E-05 |
| AC138430.1 | MACC1 | protein_coding | -0.70 | 4.51E-07 |
| AC138430.1 | TAB2 | protein_coding | 0.53 | 0.000390748 |
| AC138430.1 | HSD17B8 | protein_coding | 0.78 | 1.51E-09 |
| AC138430.1 | RNF44 | protein_coding | -0.73 | 7.17E-08 |
| AC138430.1 | PLEKHG4B | protein_coding | -0.58 | 7.21E-05 |
| AC138430.1 | ETAA1 | protein_coding | -0.51 | 0.000655596 |
| AC138430.1 | PCBD1 | protein_coding | 0.60 | 3.13E-05 |
| AC138430.1 | RBM45 | protein_coding | -0.61 | 1.97E-05 |
| AC138430.1 | NPTX1 | protein_coding | -0.58 | 6.01E-05 |
| AC138430.1 | SLC2A14 | protein_coding | 0.50 | 0.000843581 |
| AC138430.1 | MOCS1 | protein_coding | 0.68 | 8.49E-07 |
| AC138430.1 | MYRIP | protein_coding | 0.56 | 0.000136519 |
| AC138430.1 | NT5DC4 | protein_coding | -0.76 | 8.92E-09 |
| AC138430.1 | SLC27A5 | protein_coding | 0.72 | 8.64E-08 |
| AC138430.1 | FABP5 | protein_coding | -0.60 | 3.84E-05 |
| AC138430.1 | ATPAF1 | protein_coding | 0.65 | 4.28E-06 |
| AC138430.1 | KLB | protein_coding | 0.75 | 2.03E-08 |
| AC138430.1 | EPB41L5 | protein_coding | 0.67 | 1.72E-06 |
| AC138430.1 | MUC1 | protein_coding | -0.57 | 9.63E-05 |
| AC138430.1 | CHN2 | protein_coding | 0.80 | 4.01E-10 |
| AC138430.1 | SBK1 | protein_coding | -0.65 | 5.29E-06 |
| AC138430.1 | HAAO | protein_coding | 0.74 | 4.19E-08 |
| AC138430.1 | SELENOK | protein_coding | 0.61 | 2.27E-05 |
| AC138430.1 | SLC37A3 | protein_coding | -0.54 | 0.000261153 |
| AC138430.1 | MROH2A | protein_coding | 0.55 | 0.000223377 |
| AC138430.1 | CPSF6 | protein_coding | -0.74 | 2.48E-08 |
| AC138430.1 | OBSCN | protein_coding | -0.61 | 2.01E-05 |
| AC138430.1 | TAP1 | protein_coding | -0.73 | 5.73E-08 |
| AC138430.1 | TEAD4 | protein_coding | -0.81 | 1.48E-10 |
| AC138430.1 | C1QL4 | protein_coding | -0.61 | 2.15E-05 |
| AC138430.1 | BAAT | protein_coding | 0.55 | 0.000175813 |
| AC138430.1 | ACSM2A | protein_coding | 0.67 | 1.85E-06 |
| AC138430.1 | MOGAT2 | protein_coding | 0.66 | 2.33E-06 |
| AC138430.1 | AL031708.1 | protein_coding | -0.64 | 7.17E-06 |
| AC138430.1 | TWIST2 | protein_coding | -0.53 | 0.000428735 |
| AC138430.1 | MTMR1 | protein_coding | -0.50 | 0.000846269 |
| AC138430.1 | NMRK1 | protein_coding | 0.67 | 2.00E-06 |
| AC138430.1 | TSEN54 | protein_coding | -0.59 | 4.61E-05 |
| AC138430.1 | ARL17A | protein_coding | -0.51 | 0.000675344 |
| AC138430.1 | KCNIP4 | protein_coding | -0.64 | 7.97E-06 |
| AC138430.1 | STK11IP | protein_coding | -0.73 | 4.61E-08 |
| AC138430.1 | RFC4 | protein_coding | -0.72 | 1.48E-07 |
| AC138430.1 | PRPS1 | protein_coding | 0.56 | 0.000121126 |
| AC138430.1 | MRPL32 | protein_coding | 0.73 | 4.65E-08 |
| AC138430.1 | AL136295.3 | protein_coding | 0.72 | 1.24E-07 |
| AC138430.1 | PDE2A | protein_coding | 0.55 | 0.000186526 |
| AC138430.1 | GCGR | protein_coding | 0.63 | 9.75E-06 |
| AC138430.1 | B9D2 | protein_coding | -0.61 | 2.34E-05 |
| AC138430.1 | CLIP4 | protein_coding | -0.53 | 0.000354633 |
| AC138430.1 | PPP1R35 | protein_coding | -0.55 | 0.000206248 |
| AC138430.1 | RMND1 | protein_coding | 0.65 | 4.23E-06 |
| AC138430.1 | GLB1L2 | protein_coding | -0.76 | 1.07E-08 |
| AC138430.1 | NR1I2 | protein_coding | 0.69 | 5.17E-07 |
| AC138430.1 | BCHE | protein_coding | 0.71 | 1.66E-07 |
| AC138430.1 | LENG9 | protein_coding | -0.54 | 0.000241872 |
| AC138430.1 | NRTN | protein_coding | 0.53 | 0.000357921 |
| AC138430.1 | POF1B | protein_coding | -0.72 | 1.05E-07 |
| AC138430.1 | IL17B | protein_coding | -0.52 | 0.000571266 |
| AC138430.1 | OIP5 | protein_coding | -0.58 | 7.90E-05 |
| AC138430.1 | SERPINA4 | protein_coding | 0.63 | 9.43E-06 |
| AC138430.1 | ACOT6 | protein_coding | 0.67 | 1.40E-06 |
| AC138430.1 | PPARA | protein_coding | 0.80 | 2.80E-10 |
| AC138430.1 | NOS1AP | protein_coding | 0.51 | 0.00061033 |
| AC138430.1 | BDKRB1 | protein_coding | -0.76 | 6.43E-09 |
| AC138430.1 | BLACAT1 | protein_coding | -0.58 | 7.56E-05 |
| AC138430.1 | FSCN2 | protein_coding | -0.79 | 9.25E-10 |
| AC138430.1 | HSD17B4 | protein_coding | 0.86 | 8.33E-13 |
| AC138430.1 | GYG1 | protein_coding | -0.52 | 0.000546545 |
| AC138430.1 | AC022335.1 | protein_coding | 0.68 | 1.21E-06 |
| AC138430.1 | FEN1 | protein_coding | -0.55 | 0.000230423 |
| AC138430.1 | ELF4 | protein_coding | -0.83 | 1.35E-11 |
| AC138430.1 | AKAIN1 | protein_coding | 0.78 | 2.23E-09 |
| AC138430.1 | TAP2 | protein_coding | -0.50 | 0.000798892 |
| AC138430.1 | EME1 | protein_coding | -0.68 | 1.28E-06 |
| AC138430.1 | FGG | protein_coding | 0.58 | 6.44E-05 |
| AC138430.1 | WDR76 | protein_coding | -0.58 | 7.74E-05 |
| AC138430.1 | CDH3 | protein_coding | -0.60 | 4.10E-05 |
| AC138430.1 | DICER1 | protein_coding | 0.54 | 0.000270681 |
| AC138430.1 | RNF144A | protein_coding | -0.57 | 0.000111924 |
| AC138430.1 | PDK2 | protein_coding | 0.75 | 1.73E-08 |
| AC138430.1 | SMO | protein_coding | 0.54 | 0.000250748 |
| AC138430.1 | IRS1 | protein_coding | 0.70 | 3.85E-07 |
| AC138430.1 | ZNF581 | protein_coding | -0.67 | 1.80E-06 |
| AC138430.1 | SETD3 | protein_coding | 0.68 | 1.07E-06 |
| AC138430.1 | IGIP | protein_coding | 0.55 | 0.000179897 |
| AC138430.1 | FAM221B | protein_coding | 0.62 | 1.61E-05 |
| AC138430.1 | CRLS1 | protein_coding | 0.70 | 4.49E-07 |
| AC138430.1 | CES3 | protein_coding | 0.73 | 6.73E-08 |
| AC138430.1 | HASPIN | protein_coding | -0.68 | 1.06E-06 |
| AC138430.1 | DDR1 | protein_coding | -0.68 | 1.15E-06 |
| AC138430.1 | AICDA | protein_coding | -0.51 | 0.000713567 |
| AC138430.1 | DCAF16 | protein_coding | -0.69 | 5.05E-07 |
| AC138430.1 | CPNE7 | protein_coding | -0.67 | 1.39E-06 |
| AC138430.1 | CABIN1 | protein_coding | -0.64 | 6.67E-06 |
| AC138430.1 | L3MBTL1 | protein_coding | -0.66 | 2.39E-06 |
| AC138430.1 | NOTUM | protein_coding | 0.70 | 3.01E-07 |
| AC138430.1 | DHX57 | protein_coding | -0.58 | 6.29E-05 |
| AC138430.1 | STK32B | protein_coding | -0.62 | 1.42E-05 |
| AC138430.1 | MT-ND6 | protein_coding | 0.72 | 8.62E-08 |
| AC138430.1 | PRPF40B | protein_coding | -0.51 | 0.000600923 |
| AC138430.1 | IDUA | protein_coding | -0.64 | 5.81E-06 |
| AC138430.1 | DPPA4 | protein_coding | 0.57 | 0.000104711 |
| AC138430.1 | ADAMTS17 | protein_coding | 0.65 | 4.54E-06 |
| AC138430.1 | ARHGEF25 | protein_coding | -0.61 | 2.01E-05 |
| AC138430.1 | ETFBKMT | protein_coding | 0.69 | 7.00E-07 |
| AC138430.1 | LIG4 | protein_coding | 0.61 | 2.22E-05 |
| AC138430.1 | ERLIN2 | protein_coding | 0.60 | 3.58E-05 |
| AC138430.1 | DNAJB13 | protein_coding | -0.62 | 1.70E-05 |
| AC138430.1 | GRID2IP | protein_coding | -0.76 | 6.40E-09 |
| AC138430.1 | OSCP1 | protein_coding | -0.53 | 0.000374439 |
| AC138430.1 | CD2BP2 | protein_coding | -0.74 | 3.50E-08 |
| AC138430.1 | ABHD8 | protein_coding | 0.57 | 0.000108042 |
| AC138430.1 | AC092042.3 | protein_coding | 0.55 | 0.000218495 |
| AC138430.1 | RSPH1 | protein_coding | -0.52 | 0.000512806 |
| AC138430.1 | MCM4 | protein_coding | -0.59 | 4.44E-05 |
| AC138430.1 | DZIP1 | protein_coding | -0.53 | 0.000359964 |
| AC138430.1 | SOWAHB | protein_coding | 0.63 | 1.08E-05 |
| AC138430.1 | STRA6 | protein_coding | -0.62 | 1.39E-05 |
| AC138430.1 | SOX5 | protein_coding | 0.73 | 6.74E-08 |
| AC138430.1 | GATM | protein_coding | 0.68 | 9.64E-07 |
| AC138430.1 | SLC27A2 | protein_coding | 0.56 | 0.000135089 |
| AC138430.1 | ACYP1 | protein_coding | -0.56 | 0.000125384 |
| AC138430.1 | MTO1 | protein_coding | 0.65 | 4.49E-06 |
| AC138430.1 | SEPTIN7 | protein_coding | -0.50 | 0.000786192 |
| AC138430.1 | PRDM4 | protein_coding | -0.71 | 2.18E-07 |
| AC138430.1 | DSN1 | protein_coding | -0.54 | 0.000236036 |
| AC138430.1 | DHX37 | protein_coding | -0.66 | 2.91E-06 |
| AC138430.1 | HAO1 | protein_coding | 0.68 | 1.21E-06 |
| AC138430.1 | ANGPTL4 | protein_coding | 0.56 | 0.000123927 |
| AC138430.1 | CES5A | protein_coding | 0.71 | 2.55E-07 |
| AC138430.1 | DEPDC7 | protein_coding | 0.77 | 3.21E-09 |
| AC138430.1 | PTAFR | protein_coding | -0.62 | 1.53E-05 |
| AC138430.1 | RAB36 | protein_coding | -0.81 | 1.35E-10 |
| AC138430.1 | AKR1C8P | protein_coding | 0.71 | 2.48E-07 |
| AC138430.1 | SLC30A1 | protein_coding | 0.75 | 1.63E-08 |
| AC138430.1 | MTTP | protein_coding | 0.66 | 2.99E-06 |
| AC138430.1 | ITFG1 | protein_coding | 0.51 | 0.000742867 |
| AC138430.1 | RUSC1 | protein_coding | -0.70 | 3.31E-07 |
| AC138430.1 | ERGIC1 | protein_coding | 0.52 | 0.000483875 |
| AC138430.1 | FARSB | protein_coding | -0.52 | 0.000467985 |
| AC138430.1 | FGB | protein_coding | 0.63 | 9.82E-06 |
| AC138430.1 | NSMAF | protein_coding | -0.64 | 6.01E-06 |
| AC138430.1 | SMIM1 | protein_coding | 0.70 | 3.44E-07 |
| AC138430.1 | SRD5A2 | protein_coding | 0.73 | 8.50E-08 |
| AC138430.1 | CENPA | protein_coding | -0.72 | 1.37E-07 |
| AC138430.1 | MTRNR2L8 | protein_coding | 0.53 | 0.000345258 |
| AC138430.1 | GLI4 | protein_coding | -0.52 | 0.000462901 |
| AC138430.1 | MAP1A | protein_coding | -0.70 | 4.13E-07 |
| AC138430.1 | GRIP1 | protein_coding | -0.70 | 3.07E-07 |
| AC138430.1 | C4orf46 | protein_coding | -0.66 | 3.22E-06 |
| AC138430.1 | LPIN1 | protein_coding | 0.50 | 0.00081095 |
| AC138430.1 | IPMK | protein_coding | 0.58 | 7.33E-05 |
| AC138430.1 | HSPA14 | protein_coding | -0.56 | 0.000140933 |
| AC138430.1 | MOB1A | protein_coding | -0.55 | 0.000169715 |
| AC138430.1 | GPATCH4 | protein_coding | -0.52 | 0.000552761 |
| AC138430.1 | DDX20 | protein_coding | -0.57 | 9.86E-05 |
| AC138430.1 | ITLN1 | protein_coding | 0.55 | 0.000205841 |
| AC138430.1 | H4C8 | protein_coding | -0.65 | 3.52E-06 |
| AC138430.1 | ATP6V1F | protein_coding | -0.60 | 3.75E-05 |
| AC138430.1 | NNT | protein_coding | 0.67 | 1.44E-06 |
| AC138430.1 | C19orf33 | protein_coding | -0.51 | 0.000703521 |
| AC138430.1 | TFCP2L1 | protein_coding | -0.53 | 0.000413222 |
| AC138430.1 | APOC1 | protein_coding | 0.69 | 7.32E-07 |
| AC138430.1 | JHY | protein_coding | -0.58 | 6.66E-05 |
| AC138430.1 | NUMBL | protein_coding | -0.59 | 4.35E-05 |
| AC138430.1 | CLPB | protein_coding | 0.71 | 2.35E-07 |
| AC138430.1 | PIH1D2 | protein_coding | -0.57 | 9.49E-05 |
| AC138430.1 | SULT1E1 | protein_coding | 0.59 | 5.78E-05 |
| AC138430.1 | POLR3GL | protein_coding | 0.57 | 0.000106207 |
| AC138430.1 | FXN | protein_coding | 0.60 | 2.91E-05 |
| AC138430.1 | FGF21 | protein_coding | 0.59 | 5.92E-05 |
| AC138430.1 | MMAA | protein_coding | 0.73 | 4.87E-08 |
| AC138430.1 | HECTD1 | protein_coding | 0.60 | 3.69E-05 |
| AC138430.1 | G6PC | protein_coding | 0.69 | 7.23E-07 |
| AC138430.1 | SYT16 | protein_coding | -0.50 | 0.000826013 |
| AC138430.1 | MCM10 | protein_coding | -0.72 | 1.10E-07 |
| AC138430.1 | RPS3 | protein_coding | -0.58 | 7.21E-05 |
| AC138430.1 | AP5Z1 | protein_coding | -0.60 | 3.28E-05 |
| AC138430.1 | TSPAN19 | protein_coding | -0.56 | 0.000159768 |
| AC138430.1 | CHEK1 | protein_coding | -0.78 | 1.86E-09 |
| AC138430.1 | WDTC1 | protein_coding | 0.62 | 1.39E-05 |
| AC138430.1 | ATF7IP | protein_coding | -0.53 | 0.000367172 |
| AC138430.1 | KHDC1 | protein_coding | -0.66 | 2.96E-06 |
| AC138430.1 | BARD1 | protein_coding | -0.62 | 1.51E-05 |
| AC138430.1 | ABCA5 | protein_coding | 0.82 | 7.47E-11 |
| AC138430.1 | SLC2A1 | protein_coding | -0.58 | 7.12E-05 |
| AC138430.1 | CYP2C9 | protein_coding | 0.65 | 4.41E-06 |
| AC138430.1 | CLEC4M | protein_coding | 0.61 | 2.24E-05 |
| AC138430.1 | PIP5K1C | protein_coding | -0.74 | 4.38E-08 |
| AC138430.1 | SEC61A2 | protein_coding | 0.55 | 0.000181424 |
| AC138430.1 | AFP | protein_coding | 0.65 | 5.03E-06 |
| AC138430.1 | PRRT2 | protein_coding | -0.64 | 7.45E-06 |
| AC138430.1 | ITIH5 | protein_coding | -0.71 | 1.79E-07 |
| AC138430.1 | SETD1A | protein_coding | -0.64 | 7.20E-06 |
| AC138430.1 | SLC9A1 | protein_coding | -0.61 | 2.75E-05 |
| AC138430.1 | ATP5IF1 | protein_coding | 0.57 | 0.000115444 |
| AC138430.1 | DCTPP1 | protein_coding | -0.65 | 4.54E-06 |
| AC138430.1 | G6PC3 | protein_coding | -0.64 | 5.63E-06 |
| AC138430.1 | C2orf15 | protein_coding | -0.69 | 4.91E-07 |
| AC138430.1 | ACOX3 | protein_coding | 0.61 | 2.65E-05 |
| AC138430.1 | STAM | protein_coding | -0.52 | 0.000508792 |
| AC138430.1 | MT-ND4 | protein_coding | 0.72 | 1.25E-07 |
| AC138430.1 | AGXT2 | protein_coding | 0.63 | 8.46E-06 |
| AC138430.1 | ATG2A | protein_coding | 0.60 | 3.70E-05 |
| AC138430.1 | LILRB5 | protein_coding | 0.53 | 0.000374511 |
| AC138430.1 | ZNF286A | protein_coding | -0.55 | 0.000193134 |
| AC138430.1 | SALL1 | protein_coding | 0.63 | 9.93E-06 |
| AC138430.1 | TRIO | protein_coding | -0.50 | 0.000786224 |
| AC138430.1 | DBNL | protein_coding | -0.51 | 0.000704122 |
| AC138430.1 | TNNT1 | protein_coding | -0.52 | 0.000491949 |
| AC138430.1 | PPTC7 | protein_coding | 0.64 | 6.53E-06 |
| AC138430.1 | LSM8 | protein_coding | -0.67 | 1.97E-06 |
| AC138430.1 | SLC22A1 | protein_coding | 0.70 | 2.77E-07 |
| AC138430.1 | HERC3 | protein_coding | 0.54 | 0.00031601 |
| AC138430.1 | DLC1 | protein_coding | 0.56 | 0.000132349 |
| AC138430.1 | LRP10 | protein_coding | -0.53 | 0.000328349 |
| AC138430.1 | IL4I1 | protein_coding | -0.62 | 1.51E-05 |
| AC138430.1 | UBE2S | protein_coding | -0.59 | 4.96E-05 |
| AC138430.1 | PRR19 | protein_coding | -0.59 | 5.13E-05 |
| AC138430.1 | CTNND2 | protein_coding | -0.51 | 0.000594004 |
| AC138430.1 | LAMP3 | protein_coding | -0.66 | 2.27E-06 |
| AC138430.1 | SLC6A9 | protein_coding | -0.55 | 0.00020043 |
| AC138430.1 | ZMYND12 | protein_coding | 0.60 | 4.03E-05 |
| AC138430.1 | OPRPN | protein_coding | 0.68 | 9.36E-07 |
| AC138430.1 | DCT | protein_coding | 0.54 | 0.000292835 |
| AC138430.1 | NUDT12 | protein_coding | 0.61 | 2.82E-05 |
| AC138430.1 | HCN1 | protein_coding | 0.68 | 1.17E-06 |
| AC138430.1 | VTI1B | protein_coding | 0.57 | 8.78E-05 |
| AC138430.1 | FOXN4 | protein_coding | 0.71 | 1.81E-07 |
| AC138430.1 | B4GALNT4 | protein_coding | -0.75 | 1.89E-08 |
| AC138430.1 | CCN6 | protein_coding | 0.59 | 5.19E-05 |
| AC138430.1 | IFT81 | protein_coding | -0.57 | 0.000109236 |
| AC138430.1 | NRF1 | protein_coding | -0.70 | 3.38E-07 |
| AC138430.1 | SLC25A13 | protein_coding | 0.83 | 2.97E-11 |
| AC138430.1 | UQCRQ | protein_coding | 0.59 | 5.33E-05 |
| AC138430.1 | PCK1 | protein_coding | 0.68 | 8.96E-07 |
| AC138430.1 | ZNRF1 | protein_coding | 0.59 | 5.11E-05 |
| AC138430.1 | REN | protein_coding | 0.72 | 1.12E-07 |
| AC138430.1 | CCL15-CCL14 | protein_coding | 0.58 | 8.37E-05 |
| AC138430.1 | R3HDM4 | protein_coding | -0.57 | 0.000117776 |
| AC138430.1 | PCNX2 | protein_coding | -0.78 | 1.45E-09 |
| AC138430.1 | LNPEP | protein_coding | -0.66 | 2.23E-06 |
| AC138430.1 | CENPF | protein_coding | -0.68 | 1.24E-06 |
| AC138430.1 | SH2D5 | protein_coding | -0.59 | 5.52E-05 |
| AC138430.1 | CNTFR | protein_coding | 0.78 | 2.57E-09 |
| AC138430.1 | HPCA | protein_coding | -0.61 | 1.98E-05 |
| AC138430.1 | SYVN1 | protein_coding | 0.67 | 1.34E-06 |
| AC138430.1 | CEP41 | protein_coding | -0.80 | 5.33E-10 |
| AC138430.1 | RECQL5 | protein_coding | -0.54 | 0.000250141 |
| AC138430.1 | GK | protein_coding | 0.60 | 2.93E-05 |
| AC138430.1 | SHISA2 | protein_coding | -0.56 | 0.000166945 |
| AC138430.1 | PGRMC2 | protein_coding | 0.61 | 2.13E-05 |
| AC138430.1 | RNASEH2A | protein_coding | -0.51 | 0.000592048 |
| AC138430.1 | SLC35A1 | protein_coding | 0.54 | 0.000310837 |
| AC138430.1 | FDPS | protein_coding | 0.51 | 0.000687382 |
| AC138430.1 | NPEPL1 | protein_coding | -0.62 | 1.86E-05 |
| AC138430.1 | WASHC2A | protein_coding | -0.61 | 2.83E-05 |
| AC138430.1 | ENO4 | protein_coding | -0.57 | 0.000104401 |
| AC138430.1 | IZUMO4 | protein_coding | 0.59 | 4.28E-05 |
| AC138430.1 | AAGAB | protein_coding | -0.52 | 0.000473404 |
| AC138430.1 | NCAPG | protein_coding | -0.71 | 1.64E-07 |
| AC138430.1 | C8G | protein_coding | 0.51 | 0.000582486 |
| AC138430.1 | PIANP | protein_coding | -0.57 | 0.000116093 |
| AC138430.1 | TNFRSF4 | protein_coding | -0.63 | 1.08E-05 |
| AC138430.1 | FBXL19 | protein_coding | -0.84 | 3.97E-12 |
| AC138430.1 | RBMS2 | protein_coding | -0.70 | 2.82E-07 |
| AC138430.1 | HBD | protein_coding | 0.56 | 0.000149466 |
| AC138430.1 | CEACAM1 | protein_coding | 0.56 | 0.000128077 |
| AC138430.1 | FAM9B | protein_coding | 0.54 | 0.000302551 |
| AC138430.1 | OVOL3 | protein_coding | -0.54 | 0.000297833 |
| AC138430.1 | ISY1 | protein_coding | -0.51 | 0.000705143 |
| AC138430.1 | FER1L5 | protein_coding | -0.63 | 1.16E-05 |
| AC138430.1 | CKAP2L | protein_coding | -0.59 | 5.00E-05 |
| AC138430.1 | SIK2 | protein_coding | 0.67 | 1.45E-06 |
| AC138430.1 | MARCKSL1 | protein_coding | -0.54 | 0.000310403 |
| AC138430.1 | SFXN2 | protein_coding | 0.70 | 2.95E-07 |
| AC138430.1 | MRPL53 | protein_coding | -0.56 | 0.000159991 |
| AC138430.1 | 43891 | protein_coding | 0.66 | 2.33E-06 |
| AC138430.1 | BAIAP2L2 | protein_coding | -0.65 | 5.06E-06 |
| AC138430.1 | RNF123 | protein_coding | 0.76 | 1.02E-08 |
| AC138430.1 | CYP8B1 | protein_coding | 0.64 | 7.22E-06 |
| AC138430.1 | SLC35A3 | protein_coding | 0.63 | 9.58E-06 |
| AC138430.1 | RNF227 | protein_coding | -0.65 | 3.93E-06 |
| AC138430.1 | FOXN3 | protein_coding | 0.65 | 3.74E-06 |
| AC138430.1 | RABL2B | protein_coding | -0.69 | 6.67E-07 |
| AC138430.1 | MUL1 | protein_coding | 0.51 | 0.00057965 |
| AC138430.1 | ADAMTS10 | protein_coding | -0.52 | 0.000552671 |
| AC138430.1 | HMGN5 | protein_coding | 0.70 | 4.72E-07 |
| AC138430.1 | CENPI | protein_coding | -0.70 | 4.68E-07 |
| AC138430.1 | TCAIM | protein_coding | 0.78 | 1.34E-09 |
| AC138430.1 | LHPP | protein_coding | 0.66 | 2.54E-06 |
| AC138430.1 | EIF4A3 | protein_coding | -0.59 | 4.30E-05 |
| AC138430.1 | SEC24A | protein_coding | 0.60 | 3.78E-05 |
| AC138430.1 | PCDH9 | protein_coding | 0.51 | 0.000613269 |
| AC138430.1 | FOXS1 | protein_coding | -0.58 | 6.03E-05 |
| AC138430.1 | PALB2 | protein_coding | -0.58 | 7.13E-05 |
| AC138430.1 | OIT3 | protein_coding | 0.68 | 1.02E-06 |
| AC138430.1 | PNKP | protein_coding | -0.55 | 0.000205557 |
| AC138430.1 | MMS19 | protein_coding | -0.51 | 0.000583195 |
| AC138430.1 | CCDC152 | protein_coding | 0.73 | 7.67E-08 |
| AC138430.1 | CRMP1 | protein_coding | -0.73 | 7.64E-08 |
| AC138430.1 | SF3A2 | protein_coding | -0.71 | 2.09E-07 |
| AC138430.1 | C2orf50 | protein_coding | -0.74 | 3.22E-08 |
| AC138430.1 | EHBP1 | protein_coding | 0.66 | 2.51E-06 |
| AC138430.1 | UBASH3B | protein_coding | -0.75 | 2.20E-08 |
| AC138430.1 | RPIA | protein_coding | -0.62 | 1.61E-05 |
| AC138430.1 | KCND3 | protein_coding | 0.52 | 0.000574068 |
| AC138430.1 | C7orf31 | protein_coding | -0.55 | 0.000197356 |
| AC138430.1 | HSD17B10 | protein_coding | 0.67 | 1.51E-06 |
| AC138430.1 | CTR9 | protein_coding | 0.58 | 7.04E-05 |
| AC138430.1 | SLC25A25 | protein_coding | 0.71 | 2.47E-07 |
| AC138430.1 | SCN3B | protein_coding | -0.53 | 0.000388313 |
| AC138430.1 | LPCAT4 | protein_coding | -0.77 | 5.92E-09 |
| AC138430.1 | PFN3 | protein_coding | 0.64 | 5.98E-06 |
| AC138430.1 | LNP1 | protein_coding | 0.57 | 0.000120392 |
| AC138430.1 | OXER1 | protein_coding | 0.66 | 2.47E-06 |
| AC138430.1 | AL121845.2 | protein_coding | 0.80 | 4.52E-10 |
| AC138430.1 | CYP26A1 | protein_coding | 0.58 | 6.02E-05 |
| AC138430.1 | CBS | protein_coding | 0.72 | 1.20E-07 |
| AC138430.1 | SERPINA7 | protein_coding | 0.60 | 2.92E-05 |
| AC138430.1 | ABCB4 | protein_coding | 0.70 | 4.11E-07 |
| AC138430.1 | DNASE1L3 | protein_coding | 0.58 | 6.30E-05 |
| AC138430.1 | SH3RF3 | protein_coding | -0.52 | 0.000470515 |
[truncated: 924,178 more chars]
